# Supplementary material for: Effect of Important Food Sources of Fructose-Containing Sugars on Inflammatory Biomarkers: A Systematic Review and Meta-Analysis of Controlled Feeding Trials
Source: Nutrients. 2022 Sep 26;14(19):3986. doi: 10.3390/nu14193986 (PMC9572084; doi:10.3390/nu14193986)
Supplement: Supplementary file 1 [file nutrients-14-03986-s001.zip › nutrients-1922621-supplementary.pdf]

Online supplement to "Effect of important sources of fructose-containing sugars on biomarkers of inflammation: a systematic review and meta-analysis of controlled trials"

Table of Contents

Supplemental Tables 8

Supplemental Table S1: PRISMA Checklist 8

Supplemental Table S2: Search strategy for controlled trials assessing the effect of important food sources of fructose-containing sugars on biomarkers of inflammation 11

Supplemental Table S3: PICOTS framework of the search strategy 13

Supplemental Table S4: Food source definitions 14

Supplemental Table S5: Trial characteristics 15

Supplemental Table S6: Sensitivity analyses of the use of correlation coefficients of 0·25 and 0·75 for crossover trials in the primary analysis of the effect of important food sources of fructose-containing sugars on biomarkers of inflammation outcomes 35

Supplemental Table S7: GRADE certainty of evidence assessment* for the effect of fructose-containing sugars on biomarkers of inflammation by energy control 39

Supplemental Table S8: GRADE certainty of evidence assessment* for the effect of fructose-containing sugars on biomarkers of inflammation by important food source of fructose-containing sugars 42

Supplemental Figure S1: Risk of bias proportion graph for the effect of important food sources of fructose-containing sugars on CRP (mg/L) in substitution trials 47

Supplemental Figure S2: Risk of bias proportion graph for the effect of important food sources of fructose-containing sugars on CRP (mg/L) in addition trials 47

Supplemental Figure S3: Risk of bias proportion graph for the effect of important food sources of fructose-containing sugars on CRP (mg/L) in subtraction trials 48

Supplemental Figure S4: Risk of bias proportion graph for the effect of important food sources of fructose-containing sugars on CRP (mg/L) in ad libitum trials 48

Supplemental Figure S5: Risk of bias proportion graph for the effect of important food sources of fructose-containing sugars on TNF- α (pg/mL) trials in substitution trials 49

Supplemental Figure S6: Risk of bias proportion graph for the effect of important food sources of fructose-containing sugars on TNF-α (pg/mL) in addition trials 49

Supplemental Figure S7: Risk of bias proportion graph for the effect of important food sources of fructose-containing sugars on IL-6 (pg/mL) in substitution trials 50

Supplemental Figure S8: Risk of bias proportion graph for the effect of important food sources of fructose-containing sugars on IL-6 (pg/mL) in addition trials 50

Supplemental Figure S9: Forest plot of controlled trials of the effect of important food sources of fructose-containing sugars on CRP (mg/L) in substitution trials 51

Supplemental Figure S10: Forest plot of controlled trials of the effect of important food sources of fructose-containing sugars on CRP (mg/L) in addition trials 53

Supplemental Figure S11: Forest plot of controlled trials of the effect of important food sources of fructose-containing sugars on CRP (mg/L) in subtraction trials 55
Supplemental Figure S12: Forest plot of controlled trials of the effect of important food sources of fructose-containing sugars on CRP (mg/L) *ad libitum* trials.. 56
Supplemental Figure S13: Forest plot of controlled trials of the effect of important food sources of fructose-containing sugars on TNF-α (pg/mL) trials in substitution trials.............................. 57
Supplemental Figure S14: Forest plot of controlled trials of the effect of important food sources of fructose-containing sugars on TNF-α (pg/mL) trials in addition trials 59
Supplemental Figure S15: Forest plot of controlled trials of the effect of important food sources of fructose-containing sugars on IL-6 (pg/mL) in substitution trials... 61
Supplemental Figure S16: Forest plot of controlled trials of the effect of important food sources of fructose-containing sugars on IL-6 in addition trials... 63
Supplemental Figure S17: Sensitivity analysis of the systematic removal of each trial for the effect of important food sources of fructose-containing sugars on CRP (mg/L) in substitution trials.. 65
Supplemental Figure S18: Sensitivity analysis of the systematic removal of each trial for the effect of important food sources of fructose-containing sugars on CRP (mg/L) in addition trials 66
Supplemental Figure S19: Sensitivity analysis of the systematic removal of each trial in the primary analysis of the effect of important food sources of fructose-containing sugars on CRP (mg/L) in subtraction trials 67
Supplemental Figure S20: Sensitivity analysis of the systematic removal of each trial for the effect of important food sources of fructose-containing sugars on CRP (mg/L) in *ad libitum* trials 67
Supplemental Figure S21: Sensitivity analysis of the systematic removal of each trial for the effect of SSB on CRP (mg/L) in substitution trials 68
Supplemental Figure S22: Sensitivity analysis of the systematic removal of each trial for the effect of sweetened dairy on CRP (mg/L) in substitution trials... 68
Supplemental Figure S23: Sensitivity analysis of the systematic removal of each trial for the effect of 100% fruit juice on CRP (mg/L) in substitution trials 69
Supplemental Figure S24: Sensitivity analysis of the systematic removal of each trial for the effect of fruit on CRP (mg/L) in substitution trials... 69
Supplemental Figure S25: Sensitivity analysis of the systematic removal of each trial for the effect of dried fruit on CRP (mg/L) in substitution trials 70
Supplemental Figure S26: Sensitivity analysis of the systematic removal of each trial for the effect of mixed sources (with SSBs) on CRP (mg/L) in substitution trials................................. 70
Supplemental Figure S27: Sensitivity analysis of the systematic removal of each trial for the effect of mixed sources (without SSBs) on CRP (mg/L) in substitution trials............................ 71
Supplemental Figure S28: Sensitivity analysis of the systematic removal of each trial for the effect of SSB on CRP (mg/L) in addition trials.. 71

Supplemental Figure S29: Sensitivity analysis of the systematic removal of each trial for the effect of sweetened dairy alternatives (soy) on CRP (mg/L) in addition trials 72
Supplemental Figure S30: Sensitivity analysis of the systematic removal of each trial for the effect of 100% fruit juice on CRP (mg/L) in addition trials 72
Supplemental Figure S31: Sensitivity analysis of the systematic removal of each trial for the effect of fruit on CRP (mg/L) in addition trials 73
Supplemental Figure S32: Sensitivity analysis of the systematic removal of each trial for the effect of dried fruit on CRP (mg/L) in addition trials 73
Supplemental Figure S33: Sensitivity analysis of the systematic removal of each trial for the effect of sweets and desserts on CRP (mg/L) in addition trials 74
Supplemental Figure S34: Sensitivity analysis of the systematic removal of each trial for the effect of important food sources of fructose-containing sugars on TNF-α (pg/mL) in substitution trials 74
Supplemental Figure S35: Sensitivity analysis of the systematic removal of each trial for the effect of important food sources of fructose-containing sugars on TNF-α (pg/mL) in addition trials 75
Supplemental Figure S36: Sensitivity analysis of the systematic removal of each trial for the effect of SSB on TNF-a (pg/mL) in addition trials 76
Supplemental Figure S37: Sensitivity analysis of the systematic removal of each trial for the effect of sweetened dairy on TNF-a (pg/mL) in addition trials 76
Supplemental Figure S38: Sensitivity analysis of the systematic removal of each trial for the effect of 100% fruit juice on TNF-a (pg/mL) in addition trials 76
Supplemental Figure S39: Sensitivity analysis of the systematic removal of each trial for the effect of fruit on TNF-a (pg/mL) in addition trials 77
Supplemental Figure S40: Sensitivity analysis of the systematic removal of each trial for the effect of addition sweets and desserts on TNF-a (pg/mL) in addition trials 77
Supplemental Figure S41: Sensitivity analysis of the systematic removal of each trial for the effect of added nutritive (caloric) sweeteners on TNF-a (pg/mL) in addition trials 77
Supplemental Figure S42: Sensitivity analysis of the systematic removal of each trial for the effect of important food sources of fructose-containing sugars on IL-6 (pg/mL) in substitution trials 78
Supplemental Figure S43: Sensitivity analysis of the systematic removal of each trial for the effect of important food sources of fructose-containing sugars on IL-6 (pg/mL) on addition trials 79
Supplemental Figure S44: Sensitivity analysis of the systematic removal of each trial for the effect of SSB on IL-6 (pg/mL) in addition trials 80
Supplemental Figure S45: Sensitivity analysis of the systematic removal of each trial for the effect of sweetened dairy on IL-6 (pg/mL) in addition trials 80
Supplemental Figure S46: Sensitivity analysis of the systematic removal of each trial for the effect of 100% fruit juice on IL-6 (pg/mL) in addition trials 81

Supplemental Figure S47: Sensitivity analysis of the systematic removal of each trial for the effect of fruit on IL-6 (pg/mL) in addition trials 81

Supplemental Figure S48 (part 1 of 3): Subgroup analyses for the effect of important food sources of fructose-containing sugars on CRP (mg/L) in substitution trials 82

Supplemental Figure S48 (part 2 of 3): Subgroup analyses for the effect of important food sources of fructose-containing sugars on CRP (mg/L) in substitution trials 84

Supplemental Figure S48 (part 3 of 3): Subgroup analyses for the effect of important food sources of fructose-containing sugars on CRP (mg/L) in substitution trials 85

Supplemental Figure S49: Risk of bias (using The Cochrane Collaboration Tool) subgroup analysis for the effect of important food sources of fructose-containing sugars on CRP (mg/L) in substitution comparisons 86

Supplemental Figure S50 (part 1 of 3): Subgroup analyses for the effect of important food sources of fructose-containing sugars on CRP (mg/L) in addition trials 87

Supplemental Figure S50 (part 2 of 3): Subgroup analyses for the effect of important food sources of fructose-containing sugars on CRP (mg/L) in addition comparisons 88

Supplemental Figure S50 (part 3 of 3): Subgroup analyses for the effect of important food sources of fructose-containing sugars on CRP (mg/L) in addition comparisons 89

Supplemental Figure S51: Risk of bias (using The Cochrane Collaboration Tool) subgroup analysis for the effect of important food sources of fructose-containing sugars on CRP (mg/L) in addition trials 90

Supplemental Figure S52: Continuous meta-regression analysis for the effect of important food sources of fructose-containing sugars on CRP (mg/L) in substitution trials 91

Supplemental Figure S53: Continuous meta-regression analysis for the effect of important food sources of fructose-containing sugars on CRP (mg/L) in addition trials 91

Supplemental Figure S54 (part 1 of 3): Subgroup analyses for the effect of SSB on CRP (mg/L) in substitution trials 92

Supplemental Figure S54 (part 2 of 3): Subgroup analyses for the effect of SSB on CRP (mg/L) in substitution trials 93

Supplemental Figure S54 (part 3 of 3): Subgroup analyses for the effect of SSB on CRP (mg/L) in substitution trials 94

Supplemental Figure S55: Risk of bias (using The Cochrane Collaboration Tool) subgroup analysis for the effect of SSB on CRP (mg/L) in substitution trials 95

Supplemental Figure S56 (part 1 of 3): Subgroup analyses for the effect of 100% fruit juice on CRP (mg/L) in addition trials 96

Supplemental Figure S56 (part 2 of 3): Subgroup analyses for the effect of 100% fruit juice on CRP (mg/L) in addition trials 97

Supplemental Figure S56 (part 3 of 3): Subgroup analyses for the effect of 100% fruit juice on CRP (mg/L) in addition trials 98

Supplemental Figure S57: Risk of bias (using The Cochrane Collaboration Tool) subgroup analysis for the effect of 100% fruit juice on CRP (mg/L) in addition trials 99

Supplemental Figure S58: Continuous meta-regression analysis for the effect SSB on CRP (mg/L) in substitution comparisons 100

Supplemental Figure S59: Continuous meta-regression analysis for the effect of 100% fruit juice on CRP (mg/L) in addition comparisons 100

Supplemental Figure S60 (part 1 of 3): Subgroup analyses for the effect of important food sources of fructose-containing sugars on TNF-α (pg/mL) in substitution trials........ 101

Supplemental Figure S60 (part 2 of 3): Subgroup analyses for the effect of important food sources of fructose-containing sugars on TNF-α (pg/mL) in substitution trials........ 102

Supplemental Figure S60 (part 3 of 3): Subgroup analyses for the effect of important food sources of fructose-containing sugars on TNF-α (pg/mL) in substitution trials........ 103

Supplemental Figure S61: Risk of bias (using The Cochrane Collaboration Tool) subgroup analysis for the effect of important food sources of fructose-containing sugars on TNF-α (pg/mL) in substitution trials........ 104

Supplemental Figure S62 (part 1 of 3): Subgroup analyses for the effect of important food sources of fructose-containing sugars on TNF-α (pg/mL) in addition trials 105

Supplemental Figure S62 (part 2 of 3): Subgroup analyses for the effect of important food sources of fructose-containing sugars on TNF-α (pg/mL) in addition trials 106

Supplemental Figure S62 (part 3 of 3): Subgroup analyses for the effect of important food sources of fructose-containing sugars on TNF-α (pg/mL) in addition trials 107

Supplemental Figure S63: Risk of bias (using The Cochrane Collaboration Tool) subgroup analysis for the effect of important food sources of fructose-containing sugars on TNF-α (pg/mL) in addition trials 108

Supplemental Figure S64 (part 1 of 3): Subgroup analyses for the effect of important food sources of fructose-containing sugars on IL-6 (pg/mL) in substitution trials........ 109

Supplemental Figure S64 (part 2 of 3): Subgroup analyses for the effect of important food sources of fructose-containing sugars on IL-6 (pg/mL) in substitution trials........ 110

Supplemental Figure S64 (part 3 of 3): Subgroup analyses for the effect of important food sources of fructose-containing sugars on IL-6 (pg/mL) in substitution trials........ 111

Supplemental Figure S65: Risk of bias (using The Cochrane Collaboration Tool) subgroup analysis for the effect of important food sources of fructose-containing sugars on IL-6 (pg/mL) in substitution trials........ 112

Supplemental Figure S66 (part 1 of 3): Subgroup analyses for the effect of important food sources of fructose-containing sugars on IL-6 (pg/mL) in addition trials 113

Supplemental Figure S66 (part 2 of 3): Subgroup analyses for the effect of important food sources of fructose-containing sugars on IL-6 (pg/mL) in addition trials 114

Supplemental Figure S66 (part 3 of 3): Subgroup analyses for the effect of important food sources of fructose-containing sugars on IL-6 (pg/mL) in addition trials 115

Supplemental Figure S67: Risk of bias (using The Cochrane Collaboration Tool) subgroup analysis for the effect of important food sources of fructose-containing sugars on IL-6 (pg/mL) in addition trials 116

Supplemental Figure S68: Continuous meta-regression analysis for the effect of important food sources of fructose-containing sugars on TNF-α (pg/mL) in substitution trials. 117

Supplemental Figure S69: Continuous meta-regression analysis for the effect of important food sources of fructose-containing sugars on TNF-α (pg/mL) in addition trials 117

Supplemental Figure S70: Continuous meta-regression analysis for the effect of important food sources of fructose-containing sugars on IL-6 (pg/mL) in substitution trials 118

Supplemental Figure S71: Continuous meta-regression analysis for the effect of important food sources of fructose-containing sugars on IL-6 (pg/mL) in addition comparisons 118

Supplemental Figure S72: Linear and non-linear meta-regression analyses for effect of important food sources of fructose-containing sugars on CRP (mg/L) in substitution trials 119

Supplemental Figure S73: Linear and non-linear meta-regression analysis for the effect of important food sources of fructose-containing sugars on CRP (mg/L) in addition comparisons120

Supplemental Figure S74: Linear and non-linear meta-regression analyses for the effect of individual food sources of fructose-containing sugars dose on CRP (mg/L) in substitution trials 121

Supplemental Figure S75: Linear and non-linear meta-regression analyses for the effect of individual food sources of fructose-containing sugars dose on CRP (mg/L) in addition trials .. 122

Supplemental Figure S76: Non-linear dose-response analysis using public thresholds of 5% (panel A), 10% (panel B), and 25% (panel C) of energy for the effect of total food sources of fructose-containing sugars on CRP (mg/L) in substitution trials 124

Supplemental Figure S77: Non-linear dose-response analysis using public threshold of of 5% (panel A), 10% (panel B), and 25% (panel C) energy for the effect of important food sources of fructose-containing sugars on CRP (mg/L) in addition trials 126

Supplemental Figure S78: Non-linear dose-response analysis using public thresholds of 10% (panel A) of energy for the effect of the effect of SSBs on CRP (mg/L) in substitution trials .. 128

Supplemental Figure S79: Non-linear dose-response analysis using public thresholds of 5%, 10% , and 25% of energy for the effect of the effect of SSBs on CRP (mg/L) in addition trials 129

Supplemental Figure S80: Non-linear dose-response analysis using public thresholds of 5%, 10% , and 25% of energy for the effect of 100% fruit juice on CRP (mg/L) in addition trials 130

Supplemental Figure S81: Non-linear dose-response analysis using public thresholds of 5%, 10%, and 25% of energy for the effect of fruit on CRP (mg/L) in addition trials 132

Supplemental Figure S82: Linear and non-linear meta-regression analyses for the effect of important food sources of fructose-containing sugars on TNF-α (pg/mL) in substitution trials.134

Supplemental Figure S83: Linear and non-linear meta-regression analyses for the effect of important food sources of fructose-containing sugars on TNF-α (pg/mL) in addition trials 135

Supplemental Figure S84: Linear and non-linear meta-regression analyses for the effect of important food sources of fructose-containing sugars on IL-6 (pg/mL) in substitution trials. ... 136

Supplemental Figure S85: Linear and non-linear meta-regression analyses for the effect of important food sources of fructose-containing sugars on IL-6 (pg/mL) in addition trials 137

Supplemental Figure S86: Non-linear dose-response analysis using public thresholds of 5% (panel A), 10% (panel B), and 25% (panel C) of energy for the effect of important food sources of fructose-containing sugars on TNF-α (pg/mL) in substitution trials..................................... 138

Supplemental Figure S87: Non-linear dose-response analysis using public thresholds of 5% (panel A), 10% (panel B), and 25% (panel C) of energy for the effect of important food sources of fructose-containing sugars on TNF-α (pg/mL) in addition trials 140

Supplemental Figure S88: Non-linear dose-response analysis using public thresholds of 5% (panel A), 10% (panel B), and 25% (panel C) of energy for the effect of important food sources of fructose-containing sugars on IL-6 (pg/mL) in substitution trials... 142

Supplemental Figure S89: Non-linear dose-response analysis using public thresholds of 5% (panel A), 10% (panel B), and 25% (panel C) of energy for the effect of important food sources of fructose-containing sugars on IL-6 (pg/mL) addition trials 144

Supplemental Figure S90: Publication bias funnel plots for the effect of important food sources of fructose-containing sugars on CRP (mg/L) in substitution trials 146

Supplemental Figure S91: Publication bias funnel plots for the effect of important food sources of fructose-containing sugars on CRP (mg/L) in addition trials.. 147

Supplemental Figure S92: Publication bias funnel plots for the effect of SSBs on CRP (mg/L) in substitution trials.. 148

Supplemental Figure S93: Publication bias funnel plots for the effect of 100% fruit juice on CRP (mg/L) in addition trials.. 149

Supplemental Figure S94: Publication bias funnel plots for the effect of important food sources of fructose-containing sugars on TNF-α (pg/mL) in substitution trials..................................... 150

Supplemental Figure S95: Publication bias funnel plots for the effect of important food sources of fructose-containing sugars on TNF-α (pg/mL) in addition trials 151

Supplemental Figure S96: Publication bias funnel plots for the effect of important food sources of fructose-containing sugars on IL-6 (pg/mL) in substitution trials... 152

Supplemental Figure S97: Publication bias funnel plots for the effect of important food sources of fructose-containing sugars on IL-6 (pg/mL) in addition trials 153

Supplemental Figure S98: Trim and Fill funnel plot for the effect of important food sources of fructose-containing sugars IL-6 (pg/mL) in substitution trials 154

Supplemental Figure S99: Trim and Fill funnel plot for the effect of important food sources of fructose-containing sugars IL-6 (pg/mL) in addition trials... 155

Supplemental Tables

Supplemental Table S1: PRISMA Checklist

Section and Topic	Item #	Checklist item	Location where item is reported
**TITLE**			
Title	1	Identify the report as a systematic review.	Page 1
**ABSTRACT**			
Abstract	2	See the PRISMA 2020 for Abstracts checklist.	Page 2-3
**INTRODUCTION**			
Rationale	3	Describe the rationale for the review in the context of existing knowledge.	Page 4-5
Objectives	4	Provide an explicit statement of the objective(s) or question(s) the review addresses.	Page 5
**METHODS**			
Eligibility criteria	5	Specify the inclusion and exclusion criteria for the review and how studies were grouped for the syntheses.	Page 5-6
Information sources	6	Specify all databases, registers, websites, organisations, reference lists and other sources searched or consulted to identify studies. Specify the date when each source was last searched or consulted.	Page 5
Search strategy	7	Present the full search strategies for all databases, registers and websites, including any filters and limits used.	Page 5, Supplemental Tables S2-S3
Selection process	8	Specify the methods used to decide whether a study met the inclusion criteria of the review, including how many reviewers screened each record and each report retrieved, whether they worked independently, and if applicable, details of automation tools used in the process.	Page 5-7
Data collection process	9	Specify the methods used to collect data from reports, including how many reviewers collected data from each report, whether they worked independently, any processes for obtaining or confirming data from study investigators, and if applicable, details of automation tools used in the process.	Page 7-9
Data items	10a	List and define all outcomes for which data were sought. Specify whether all results that were compatible with each outcome domain in each study were sought (e.g. for all measures, time points, analyses), and if not, the methods used to decide which results to collect.	Page 8-9
	10b	List and define all other variables for which data were sought (e.g. participant and intervention characteristics, funding sources). Describe any assumptions made about any missing or unclear information.	Page 7-9
Study risk of bias assessment	11	Specify the methods used to assess risk of bias in the included studies, including details of the tool(s) used, how many reviewers assessed each study and whether they worked independently, and if applicable, details of automation tools used in the process.	Page 7-8

Section and Topic	Item #	Checklist item	Location where item is reported
Effect measures	12	Specify for each outcome the effect measure(s) (e.g. risk ratio, mean difference) used in the synthesis or presentation of results.	Page 8
Synthesis methods	13a	Describe the processes used to decide which studies were eligible for each synthesis (e.g. tabulating the study intervention characteristics and comparing against the planned groups for each synthesis (item #5)).	Page 8-10
	13b	Describe any methods required to prepare the data for presentation or synthesis, such as handling of missing summary statistics, or data conversions.	Page 8-10
	13c	Describe any methods used to tabulate or visually display results of individual studies and syntheses.	Page 8-10
	13d	Describe any methods used to synthesize results and provide a rationale for the choice(s). If meta-analysis was performed, describe the model(s), method(s) to identify the presence and extent of statistical heterogeneity, and software package(s) used.	Page 8-10
	13e	Describe any methods used to explore possible causes of heterogeneity among study results (e.g. subgroup analysis, meta-regression).	Page 8-10
	13f	Describe any sensitivity analyses conducted to assess robustness of the synthesized results.	Page 8-10
Reporting bias assessment	14	Describe any methods used to assess risk of bias due to missing results in a synthesis (arising from reporting biases).	Page 7
Certainty assessment	15	Describe any methods used to assess certainty (or confidence) in the body of evidence for an outcome.	Page 10-11
**RESULTS**			
Study selection	16a	Describe the results of the search and selection process, from the number of records identified in the search to the number of studies included in the review, ideally using a flow diagram.	Page 11-12, Figure 1
	16b	Cite studies that might appear to meet the inclusion criteria, but which were excluded, and explain why they were excluded.	Page 11-12, Figure 1
Study characteristics	17	Cite each included study and present its characteristics.	Page 12, Table 1, Supplemental Table S5
Risk of bias in studies	18	Present assessments of risk of bias for each included study.	Page 13, Supplemental Figures S1-S8
Results of individual studies	19	For all outcomes, present, for each study: (a) summary statistics for each group (where appropriate) and (b) an effect estimate and its precision (e.g. confidence/credible interval), ideally using structured tables or plots.	Page 14-15, Figures 2-4, Supplemental Figures S9-S16
	20a	For each synthesis, briefly summarise the characteristics and risk of bias among contributing studies.	Page 12-13

Section and Topic	Item #	Checklist item	Location where item is reported
Results of syntheses	20b	Present results of all statistical syntheses conducted. If meta-analysis was done, present for each the summary estimate and its precision (e.g. confidence/credible interval) and measures of statistical heterogeneity. If comparing groups, describe the direction of the effect.	Page 14-15, Figures 2-4, Supplemental Figures S9-S16
	20c	Present results of all investigations of possible causes of heterogeneity among study results.	Page 15-18
	20d	Present results of all sensitivity analyses conducted to assess the robustness of the synthesized results.	Page 15-20
Reporting biases	21	Present assessments of risk of bias due to missing results (arising from reporting biases) for each synthesis assessed.	Page 13, 15-18
Certainty of evidence	22	Present assessments of certainty (or confidence) in the body of evidence for each outcome assessed.	Page 20-21
**DISCUSSION**			
Discussion	23a	Provide a general interpretation of the results in the context of other evidence.	Page 21-25
	23b	Discuss any limitations of the evidence included in the review.	Page 27-28
	23c	Discuss any limitations of the review processes used.	Page 27-28
	23d	Discuss implications of the results for practice, policy, and future research.	Page 28-29
**OTHER INFORMATION**			
Registration and protocol	24a	Provide registration information for the review, including register name and registration number, or state that the review was not registered.	Page 5
	24b	Indicate where the review protocol can be accessed, or state that a protocol was not prepared.	Page 5
	24c	Describe and explain any amendments to information provided at registration or in the protocol.	Page 5
Support	25	Describe sources of financial or non-financial support for the review, and the role of the funders or sponsors in the review.	Page 30-31
Competing interests	26	Declare any competing interests of review authors.	Page 31-37
Availability of data, code and other materials	27	Report which of the following are publicly available and where they can be found: template data collection forms; data extracted from included studies; data used for all analyses; analytic code; any other materials used in the review.	Page 31

N/A=not applicable.
Table obtained from Page et al. 2021.[1]

1. Page MJ, Moher D, Bossuyt PM, et al. PRISMA 2020 explanation and elaboration: updated guidance and exemplars for reporting systematic reviews. BMJ 2021;372:n160. doi: 10.1136/bmj.n160 [published Online First: 2021/03/31]

Supplemental Table S2: Search strategy for controlled trials assessing the effect of important food sources of fructose-containing sugars on biomarkers of inflammation

Database and search terms					
**MEDLINE**		**EMBASE**		**The Cochrane Library of Controlled Trials**	
1	exp fructose/	1	exp fructose/	1	exp fructose/
2	fructose.mp.	2	fructose.mp.	2	fructose.mp.
3	exp sucrose/	3	exp sucrose/	3	exp sucrose/
4	sucrose.mp.	4	sucrose.mp.	4	sucrose.mp.
5	sugar*.mp.	5	sugar*.mp.	5	sugar*.mp.
6	SSB.mp.	6	SSB.mp.	6	SSB.mp.
7	sweetened.mp.	7	sweetened.mp.	7	sweetened.mp.
8	exp soft drink/	8	exp soft drink/	8	exp soft drink/
9	soft drink*.mp.	9	soft drink*.mp.	9	soft drink*.mp.
10	cola.mp.	10	cola.mp.	10	cola.mp.
11	exp Honey/	11	exp Honey/	11	exp honey/
12	honey.mp.	12	honey.mp.	12	honey.mp.
13	exp fruit/	13	exp fruit/	13	exp fruit/
14	fruit.mp.	14	fruit.mp.	14	fruit.mp.
15	exp carbonated beverage/	15	exp carbonated beverage/	15	exp carbonated beverages/
16	carbonated beverage*.mp.	16	carbonated beverage*.mp.	16	carbonated beverage*.mp.
17	exp energy drink/	17	exp energy drink/	17	exp energy drinks/
18	energy drink*.mp.	18	energy drink*.mp.	18	energy drink*.mp.
19	HFCS.mp	19	HFCS.mp	19	HFCS.mp.
20	sugar* sweetened beverage*.mp.	20	sugar* sweetened beverage*.mp.	20	sugar* sweetened beverage*.mp.
21	1 or 2 or 3 or 4 or 5 or 6 or 7 or 8 or 9 or 10 or 11 or 12 or 13 or 14 or 15 or 16 or 17 or 18 or 19 or 20	21	1 or 2 or 3 or 4 or 5 or 6 or 7 or 8 or 9 or 10 or 11 or 12 or 13 or 14 or 15 or 16 or 17 or 18 or 19 or 20	21	1 or 2 or 3 or 4 or 5 or 6 or 7 or 8 or 9 or 10 or 11 or 12 or 13 or 14 or 15 or 16 or 17 or 18 or 19 or 20
22	exp interleukins/	22	exp interleukins/	22	exp interleukins/
23	interleukin-6.mp.	23	interleukin-6.mp.	23	interleukin-6.mp.
24	IL-6.mp.	24	IL-6.mp.	24	IL-6.mp.
25	C-reactive protein.mp.	25	C-reactive protein.mp.	25	C-reactive protein.mp.
26	CRP.mp	26	CRP.mp.	26	CRP.mp.
27	Tumor Necrosis Factor-alpha.mp.	27	Tumor Necrosis Factor-alpha.mp.	27	Tumor Necrosis Factor-alpha.mp.
28	tnf-alpha.mp.	28	tnf-alpha.mp.	28	tnf-alpha.mp.
29	22 or 23 or 24 or 25 or 26 or 27 or 28	29	22 or 23 or 24 or 25 or 26 or 27 or 28	29	22 or 23 or 24 or 25 or 26 or 27 or 28
30	21 and 29	30	21 and 29	30	21 and 29
31	exp cohort studies/	31	exp cohort analysis/		
32	cohort$.mp.	32	exp longitudinal study/		
33	epidemiologic methods/	33	exp prospective study/		

34 exp case-control studies/<br>35 (case$ and control$).mp.<br>36 (case$ and series).mp.<br>37 case reports.pt.<br>38 (case$ adj2 report$).mp.<br>39 (case$ adj2 stud$).mp.<br>40 31 or 32 or 33 or 34 or 35 or 36 or 37 or 38 or 39<br>41 30 not 40<br>42 Limit 41 to animals<br>43 41 not 42<br>44 random:.tw.<br>45 clinical trial:.mp.<br>46 exp health care quality/<br>47 44 or 45 or 46<br>48 43 and 47	34 exp follow up/<br>35 cohort$.mp.<br>36 exp case control study/<br>37 (case$ and control$).mp.<br>38 case report/<br>39 (case$ adj2 report$).mp.<br>40 (case$ adj2 stud$).mp.<br>41 exp case study/<br>42 (case$ and series).mp.<br>43 31 or 32 or 33 or 34 or 35 or 36 or 37 or 38 or 39 or 40 or 41 or 42<br>44 30 not 43<br>45 Limit 44 to animal studies<br>46 44 not 45<br>47 random:.tw.<br>48 clinical trial:.mp.<br>49 exp health care quality/<br>50 47 or 48 or 49<br>51 46 and 50	

CRP=C reactive protein; IL-6=Interleukin-6; TNF-alpha= tumour necrosis factor alpha; SSB= sugar sweetened beverages; HFCS= high fructose corn syrup

Supplemental Table S3: PICOTS framework of the search strategy

Participants	Interventions	Comparators	Outcomes	Time	Study design
Individuals of all ages and health backgrounds	Food sources of fructose-containing sugars	Diets and foods free or lower (minimum 5g sugar difference) in fructose-containing sugars	CRP, TNF-α, and IL-6, mean difference and 95% confidence intervals	≥7 days	Controlled trials done in humans

CRP = C-reactive protein; IL-6 = Interleukin 6; PICOTS=participants, interventions, comparators, outcomes, time and study design; TNF-α = Tumour necrosis factor-alpha

Supplemental Table S4: Food source definitions

Food source of fructose-containing sugars	Definition
SSB	Carbonated or non-carbonated beverages where all or the majority of sugars are added sugars. This also includes interventions where sugars were provided to participants as crystalline packages and where they are instructed to add or incorporate into beverages.
Sweetened dairy	Animal dairy products sweetened with added sugars and where the control includes non-dairy products. These would contain both added and naturally occurring sugars.
Sweetened dairy alternatives (soy)	Soy-based dairy products sweetened with added sugars and where the control includes non-soy-based dairy products.
Sweetened dairy alternative (other)	Other plant-based dairy products sweetened with added sugars and where the control includes non-plant-based dairy products.
Fruit drink	Fruit drinks which are derived from fruit juices or fruit flavouring with added sugars. These must contain added and may also contain naturally occurring sugars.
100% Fruit juice	Fruit juice which is derived 100% from fruits with no added sugar. The one exception was cranberry juice, in which a small amount of added sugars was added for palatability.
Fruit	Includes whole fruit, freeze-dried powdered fruit, smoothies in which the only difference between intervention groups is the fruit present. The dose of the sugars under investigation is naturally occurring coming from fruit.
Dried fruit	Includes unsweetened and sweetened dried fruit. Sugars can be naturally occurring, or both naturally occurring and added.
Mixed fruit forms	Interventions include two or more of the food sources of fruit sugars (i.e., fruit, dried fruit, 100% fruit juice). Sugars are naturally occurring coming from fruit.
Sweetened cereal grains and bars	Includes sweetened dried cereal, nut bars and fruit and nut bars. Sugars are added.
Sweets and desserts	Includes cookies, cakes, muffins, confectionaries, fondant, etc. Sugars are added.
Honey	Honey was provided to participants.
Added nutritive (caloric) sweetener	Sugars provided to participants as crystalline packages, where they are instructed to add or incorporate it to various foods. Sugars are added regulatory designations.
Mixed sources (with SSBs)	Interventions where fructose-containing sugars were consumed in the form of SSBs in addition to other food sources. Examples include whole dietary interventions. Sugars can be added, or both naturally occurring and added.
Mixed sources (without SSBs)	Interventions include two or more of the above food sources of fructose-containing sugars with the exception of SSBs. Sugars can be added, or both naturally occurring and added.

SSB; Sugar sweetened beverages

Supplemental Table S5: Trial characteristics

Study, Year	Participants (M, W)	Age Mean (SD or range) (years)	Setting	BW Mean (SD or range) (kg)	BMI Mean (SD or range) (kg/m^2)	CRP Mean (SD or range) (mg/L)	IL-6 Mean (SD) (pg/mL)	TNF-α Mean (SD) (pg/mL)	Design	Feeding Control[a]	Randomization	Sugars and Control Source	Intervention or Comparator	Fructose-containing sugars dose (g/d)(%E)[b]	Diet (% C:F:P)c	Energy Balance[d]	Follow-up	Funding Sources[e]	Inflam Med use
**Substitution Trials**																			
**Food Source:**																			
**SSB**																			
**Aeberli et al. 2011**	29 H (29M, 0W)	26 (7)	OP, Switzerland	73.7 (8.8)	22.4 (1.9)	0.2 (0.4)	NR	NR	C	Supp	Y					Neutral	3 wk	A, I	N
Intervention	29 H (29M, 0W)											Fructose	600mL/d SSB	~80 (13)	55:32:13				
Intervention	29 H (29M, 0W)											Sucrose	600mL/d SSB	~80 (12)	55:32:13				
Intervention	29 H (29M, 0W)											Fructose	600mL/d SSB	~40 (7)	51:35:14				
Control	29 H (29M, 0W)											Glucose	600mL/d SSB						
**Chiu et al. 2020**	30 OW/OB children (30B, 0G)	15 (2)	OP, USA	86.8 (15.7)	1.8 (0.5)	NR	NR	NR	C	Supp	Y				49:36:16	Neutral	3 wk	A, I	N
Intervention												HFCS	HFCS-sweetened beverage	~110 (22)					
Control												Milk	2% milk						
**Cox et al. 2012**	30 H	54	IP/OP, USA	85.9[f]	29.3[f]			NR	P	Met, Supp	N				55:30:15	Neutral	10 wk	A	N
Intervention	16 H	53				3.7 (3.2)	3.5 (2.8)					Fructose	Fructose-sweetened beverage	~125 (25)					
Control	14 H	55				5.7 (9.3)	3.3 (2.7)					Glucose	Glucose-sweetened beverage						
**Jin et al. 2014**	21 OW (11M, 10W)	14(3)	OP, USA		NR		NR	NR	P	Supp	Y				NR	Neutral	4 wk	A	N

Intervention	9 OW (3M, 6W)	14(3)		82.3(16.9)		6.8(9.5)						Fructose	Fructose-sweetened beverage	~99 (20)					
Control	12 OW (8M, 4W)	13(3)		82(14.8)		5.21(4.6)						Glucose	Glucose-sweetened beverage						
**Johnston et al. 2013**	32 OW/OB (32M, 0W)		OP, UK	95.3 (5.7)					P	Positive EB: Supp; Neutral EB: Met	Y				55:30:15	Neutral and positive	2 wk	A	NR
Intervention	15 OW/OB (15M, 0W)	34 (10)		96.8 (7.4)	30 (1.4)	1.0 (1.1)	3.6 (4.8)	1.9 (0.5)				Fructose	Fructose-sweetened beverage	~221 (25)					
Intervention	15 OW/OB (15M, 0W)	35 (11)		96.8 (7.4)	30 (1.4)	1.0 (1.1)	3.6 (4.8)	1.9 (0.5)				Fructose	Fructose-sweetened beverage	~221 (25)					
Control	17 OW/OB (17M, 0W)	33 (9)		93.9 (8.7)	28.9 (1.7)	1.4 (1.5)	5.0 (13.9)	2.0 (0.3)				Glucose	Glucose-sweetened beverage						
**Kuzma et al. 2016**	24 H (15M, 9W)	36 (12)	OP, USA	NR	27.4 (4.8)	1.8 (1.5)	NR	NR	C	Supp	Y					Positive	1 wk	A	NR
Intervention												Fructose	Fructose-sweetened beverage	~125 (25)	43:12:13				
Intervention												HFCS	HFCS-sweetened beverage	~125 (25)	43:12:13				
Control												Glucose	Glucose-sweetened beverage		42:24:14				
**Sweetened Dairy**																			
**Angelopoulos et al. 2016**	267 H	38(12)	OP, USA	73.6 (12.4)	26.3 (3.3)	1.6 (1.8)	NR	NR	P	Supp	Y				NR	Neutral	10 wk	I	N
Intervention	61 H	36(1)										HFCS	18% HFCS in milk	~86.8 (18)					
Intervention	64 H	40(13)										Sucrose	18% Sucrose in milk	~93.1 (18)					
Intervention	65 H	39(12)										Fructose	9% Fructose in milk	~46.5 (9)					

Control	77 H	36(2)										Glucose	9% Glucose in milk						
**Sweetened Dairy Alternatives (Soy)**																			
**Eslami et al. 2019**	64 OW/OB (19M, 45W)	46(10)	OP, Iran				NR	NR	P	Supp	Y				55:30:15	Negative	8 wk	A, I	N
Intervention	32 OW/OB (10M, 22W)	46(11)		83.8 (9.8)	30.9(3.6)	3.7 (1.8)						Sucrose	Sweetened soy beverage	~5(1)					
Control	32 OW/OB (9M, 23W)	45(10)		84.5(14)	31.4(3.7)	3.5 (1.4)						Mixed Diet	Grains/starches/fats						
**100% Fruit Juice**																			
**Ponce et al. 2019**	72 MetS (23M, 49W)	48(9)	OP, Brazil	95(16)	34.6 (4.1)	8.1(0.6)	NR	NR	P	Supp	Y					Neutral	12 wk	A	NR
Intervention	36 MetS (12M, 24W)	49(9)		96(16)	34 (4.2)							Fruit	500 mL orange juice/d	~44 (~12.2)	49:27:24				
Control	36 MetS (11M, 25W)	46(9)		95(15)	35.1 (4.1)							Fat	Energy equivalent nuts		48:28:24				
**Ribeiro et al 2017**	78 OB (24M, 54W)	36(1)	OP, Brazil	97.5(12)	33(3)		NR	NR	P	Supp	Y				~60:35:15	Negative	12 wk	A, I	N
Intervention	39 OB	37(1)		97(12)	33(3)	0.5 (0.6)						Fruit juice	500 mL orange juice/d	~44 (~8.8)					
Control	39 OB	33(1)		98(12)	35(4)	0.5 (0.6)						Mixed Diet	Energy equivalent food item						
**Fruit**																			
**Du et al. 2019**	49 OA (14M, 35W)		OP, USA	NR	32.2 (6.2)	NR			P	Supp	Y				NR	Positive	16 wk	I	N
Intervention	27 OA (9M, 18W)	56(10)					1.5 (0.5)	2.2 (0.3)				Fruit	Freeze dried whole blueberry	~3.4 (0.7)					
Control	22 OA (5M, 17W)	55(8)					1.3 (0.5)	2.0 (0.3)				Maltodextrin	Placebo powder						
**Fridell et al. 2018**	30H (18M, 12W)	24 (4)	OP, Sweden		22.3 (1.9)				P	DA	Y				NR	Positive	8 wk	A	N

Intervention	15H (7M, 8W)			66.5 (8.7)	22.2 (1.6)	1.2 (1.5)	26.4 (10)	6.8 (2.6)				Fruit	Moderate fructose from fruit	~96 (14.6)					
Control	15H (11M, 4W)			73.6 (9)	22.5 (2.3)	0.6 (0.7)	28.4 (15)	6.8 (2.9)				Fat	Nuts						
**Kolehmainen et al. 2012**		52(7)	OP, Finland					NR	P	Supp	Y					Neutral	8 wk	A	N
Intervention	13 OW	53(6)		85.4 (12.1)	31.4 (4.7)	2.9 (1.7)	4.1 (1.6)					Fruit	Moderate fructose from bilberry	~33.6 (6.7)	54:29:15[g]				
Control	11 OW	50(7)		93.1 (10.8)	32.9 (3.4)	2.8(2.8)	3.7 (1.5)					Starch	Low fructose						
**Lehtonen et al. 2011**	80 OW/OB (0M, 80W)	44(6)	OP, Finland	81.6 (8.5)	29.6 (2.1)	NR	NR	NR	C	Supp	Y				NR	Neutral	5 wk	A,I	Y
Intervention												Fruit	Bilberries	~8.4 (1.7)					
Control												Mixed Diet	Diet alone						
**Moazen et al. 2013**	36 T2D (13M, 23W)	52(11)	OP, Iran					NR	P	Supp	Y				NR	Neutral	6 wk	A,I	N
Intervention	19 T2D	52(8)		75.8 (9.3)	27.3 (3.3)	5.5 (1.1)	2.5 (2.0)					Fruit	Moderate fructose from fruit (strawberry powder)	~24.5 (5.6)					
Control	17 T2D	51(14)		73 (11.8)	28.8 (4.2)	5.5 (1.1)	3.2 (5.1)					Lactose	Low fructose						
**Navaei et al. 2019**	40 MetS (10M, 30W)	59(5)	OP, USA		33.2 (4.9)			NR	C	Supp	Y					Positive	12 wk	I	Y
Intervention						5.5 (1.1)	5.3 (4.7)					Fruit	Moderate fructose from fruit (pears)	~16 (3.2)	48:37:16				
Control						5.5 (1.1)	5.1 (4.2)					Maltodextrin	Low fructose						
**Dried Fruit**																			
**Kanellos et al. 2017**	33 H		OP, Greece				NR	NR	P	Supp	Y					Neutral	4 wk	A, I	N
Intervention	20 H	31 (8)[h]		78.1 (12.4)	24.4 (2.7)	1.9 (1.3)						Fruit	90g raisins replacing snacks	~60 (~12)	41:27:13				
Control	13 H	30 (5)[h]		77.5 (13.3)	24.4 (2.9)	1.5 (2.2)						Mixed Diet	Usual diet		43:33:17				

**Kanellos et al. 2014**	48 T2D (25M, 23W)		OP, Greece				NR	NR	P	Supp	Y				NR	Neutral	24 wk	A, I	N
Intervention	26 T2D (15M, 11W)	64(6)		83.4 (13.8)	30.5 (4.4)	2.2 (0.9)						Fruit	36g/d of Corinthian raisins replacing snacks	~24 (4.9)					
Control	22 T2D (10M, 12W)	63(9)		81.2 (14.3)	30.4 (5.5)	3.1 (1.5)						Mixed Diet	Usual diet, no grapes/raisins						
**Kaliora et al. 2016**	44 NAFLD	NR	OP, Greece						P	Supp	Y				50:30:20	Negative	24 wk	A, I	N
Intervention	23 NAFLD			85.7 (14.3)	29.7 (22.2)	2.1 (1.9)	1.6 (1.4)	0.9 (1.1)				Fruit	36g/d of Corinthian raisins replacing snacks	~24 (~4.98)					
Control	21 NAFLD			82 (3)	29.1 (21.8)	2.4 (3.1)	1.7 (3.2	1.3 (1)				Mixed Diet	Usual diet, no grapes/raisins						
**Lehtonen et al. 2011**	80 OW/OB (0M, 80W)	44(6)	OP, Finland	81.6 (8.5)	29.6 (2.1)	NR	NR	NR	C	Supp	Y				NR	Neutral	5 wk	A,I	Y
Intervention												Dried Fruit	Sea Buckthorn berry	~8.4 (1.7)					
Control												Mixed Diet	Diet alone						
**Puglisi et al. 2008**	22 H (11 M, 11 W)	56 (5)	OP, USA	78.6 (16.0)	27.7 (3.8)	NR	NR	4.39 (2.6)	P	Supp	Y					Positive	6 wk	I	N
Intervention	10 H (5 M, 5 W)	58 (5)		78.4 (15.9)	27.5 (3.8)							Dried fruit	Walking + 1 cup raisins/d	~86 (17.2)	56:29:15				
Control	12 H (6 M, 6 W)	55 (4)		78.7 (16.8)	27.9 (3.9)							Diet alone	Walking						
**Mixed Fruit Forms**																			
**Lehtonen et al. 2010**		43(6)	OP, Finland	81.6(8.5)				1 (1)	P	Supp	Y					Neutral	20 wk	A,I	N
Intervention	28OW(0M, 28W)				29.3(2.2)	2.2 (2.1)	1.0 (1.1)	1.0 (1.1)				Fruit	Fresh berries	~14.7(3.3)	50:32:17				
Control	22OW(0M, 22W)				29.5(1.8)	2.3(2.1)	1.0 (1.1)	1.0 (1.0)				Mixed Diet	Mixed snacks						
**Added Nutritive (caloric) Sweetener**																			

**Sadeghi et al. 2020**	42 T2D (18M, 24W)	58(10)	OP, Iran	72 (11.9)	27.8 (3.6)		NR	NR	C	Supp	Y					Neutral	8 wk	A	N
Intervention						0.7 (0.1)						Honey	50g/d honey	41(8)	66:23:11				
Control						0.6 (0.2)						Mixed diet alone	No honey						
**Mixed sources (with SSBs)**																			
**Brymora et al. 2012**	28 CKD (17M, 11W)	59(15)	OP, Poland	85.8 (11.5)	29.9 (4.2)		NR		C	DA	N				55:30:15	Neutral		A	N
Intervention						3.3 (4.5)		2.4 (1.7)				Fructose, sucrose	Regular basal diet. Including SSBs	~59 (10.2)			2 wk		
Intervention						3.3 (4.5)		2.4 (1.7)				Fructose, sucrose	Regular basal diet resumed. Including SSBs	~53 (9.1)			6 wk		
Control						4.3 (4.9)		2.7 (2.5)				Starch	Low-fructose diet - restrict SSB, NSB and fruits				6 wk		
**Jalilvand et al. 2020**	40 T2D (16M, 24W)		OP, Iran				NR	NR	P	DA	Y				55:30:15	Neutral	8 wk	A	N
Intervention	20 T2D (8M, 12W)	53 (7)		63.3 (6)	24.2 (1.6)	2.8 (1.7)						Mixed fructose types	Conventional diabetic diet from ADA 2017 guideline	25 (5)					
Control	20 T2D (8M, 12W)	53 (8)		63.6 (5.4)	24.6 (1.4)	2.7 (1.5)						Mixed comparator	Restrict sucrose-sweetened and artificially sweetened drinks and foods	8.7 (1.8)					

**Khodami et al. 2022**	43 NAFLD (32M, 11W)		OP, Iran				NR		P	DA	Y		Both groups received advice on a balanced diet (emphasizing lean meats, low-fat dairy, whole grains, legumes, fruits, and vegetables) and moderate physical activity	NR		Neutral	12 wk	A	N
Intervention	21 NAFLD (17 M, 4W)	46 (11)		96.5 (18.5)	32.3 (4.3)	3.8 (0.8)		4.9 (2.1)				Mixed	Usual diet		58:33:14				
Control	22 NAFLD (15M, 7W)	41 (11)		91.7 (14.7)	31.4 (3.8)	3.8 (1.1)		4.6 (1.5)				Mixed	Low sugar diet (<10%E from free sugars)		63:33:13				
**Nilholm et al. 2021**	105 IBS (23M, 82W)		OP, Sweden		NR				P	DA	Y					Neutral	4wk	A	N
Intervention	25 IBS (3 M, 22 W)	35 (29-50)		68 (57-75)		1.5 (2.6)	0.7 (0.4)	2.9 (3.3)				Sucrose	Regular diet	20 (6.3)	42:38:17				
Control	80 IBS (20 M, 60 W)	48 (37-57)		72 (64-85)		2.4 (4)	0.9 (1.5)	4.1 (14)				Mixed	Starch or sucrose reduced diet (kept energy the same)		25:47:21				
**Souto et al. 2013**	33 DM1 (21 M, 12 W)	22 (5)	OP, Brazil	NR			NR	NR	P	DA	N					Neutral	12 wk	NR	N
Intervention	15 DM1 (8 M, 7 W)				24.0 (2.6)	0.38 (0.2)						Sucrose	encouraged to select Sucrose containing foods from an exchange list	162 (7)	58:26:20				
Control	18 DM1 (12 M, 6 W)				22.4 (2.7)	0.27 (0.2)						Starch	Isocaloric exchange of sucrose for sucrose-free foods based on an exchange list		53:24:20				
**Mixed sources (without SSBs)**																			

**Goss et al. 2020**	34 OW/OB (12M, 22W)		OP, USA						P	Supp, DA	Y					Negative	8wk	A	N
Intervention	15 OW/OB (5M, 10 W)	70 (3)		96.8 (30.9)	34.7 (3.8)	3.7 (3.7)	1.6 (0.9)	1.8 (0.9)				Mixed	Low-fat diet, breakfast bars	78 (20.3)	55:20:25				
Control	19 OW/OB (7M, 12W)	70 (4)		93.7 (15.1)	34.0 (3.4)	3.2 (2.3)	2.2 (1.1)	1.5 (1)				Fat	Eggs, high-fat diet						
**Maki et al. 2020**	30 MetS (11M, 19W)	54 (10)	OP, USA	89.6 (13.1)	31.9 (3.8)	2.1 (2.4)	NR	NR	C	Supp	Y					Neutral	4 wk	I	N
Intervention												Mixed	Breakfasts included ready-to-eat cereals with milk, waffles with syrup, granola bar, fresh and dried fruits	25 (4.7)	~60:31:12				
Control												Protein	Rotation of 3 egg-based breakfasts each providing 2 eggs/d, 6 d/wk		~41:32:26				
**Palacios et al. 2020**	66 pre-DM (17M, 16W)	48 (13)	OP, USA	89 (17)	31 (4)			NR	C	Supp	Y					Neutral	6 wk	I	N
Intervention						2.1 (0.4)	1.9 (0.4)					Mixed	20%E from free sugars provided as candy and SSB	36 (6.4)	62:19:15				
Control						2.2 (0.4)	1.9 (0.4)					Fat	20% E from saturated fat		35:46:14				
**Van Mejil et al. 2010**	35 H (10M, 25W)	50 (13)	OP, Netherlands	NR	32.0 (3.8)	NR	NR	NR	C	Supp	Y					Positive	8 wk	I	N
Intervention												Sucrose	43 g fruit biscuits/d	~70.2 (45)	53:30:16				
Control												Dairy	Yogurt		46:33:19				

**Addition Trials**																			
**Food Source:**																			
**SSB**																			
**Aeberli et al. 2011**	29 H (29M, 0W)	26 (7)	OP, Switzerland	73.7 (8.8)	22.4 (1.9)	0.2 (0.4)	NR	NR	C	Supp	Y					Positive	3wk	A, I	N
Intervention	29 H (29M, 0W)											Fructose	600mL/d SSB	~80 (13)	55:32:13				
Intervention	29 H (29M, 0W)											Sucrose	600mL/d SSB	~80 (12)	55:32:13				
Intervention	29 H (29M, 0W)											Fructose	600mL/d SSB	~40 (7)	51:35:14				
Control	29 H (29M, 0W)											Mixed	Reduce free fructose intake		46:38:16				
**Johnston et al. 2013**	32 OW/OB (32M, 0W)	34 (10)	OP, UK						P		N				55:15:30	Positive	2 wk	A	NR
Intervention	15 OW/OB (15M, 0W)	35 (11)		96.8 (7.4)	30 (1.4)	1.0 (1.1)	3.6 (4.8)	1.9 (0.5)		Supp		Fructose	Hypercaloric fructose-sweetened beverage	~125 (25)					
Control	17 OW/OB (17M, 0W)	33 (9)		93.9 (8.7)	28.9 (1.7)	1.4 (1.5)	5.0 (13.9)	2.0 (0.3)		Met		Glucose	Isocaloric glucose-sweetened beverage						
**Njike et al. 2011**	39 OW/OB (6 M, 33 W)		OP, USA			1.0 (0.1-12.7)	NR	NR	C	Supp	Y					Positive	6 wk	A,I	N
Intervention	39 OW/OB (6 M, 33 W)	52 (11)		179.5 (23.2)	30.3 (3.4)							Sucrose	Sugar-sweetened hot cocoa beverage	~91(18.2)	~55:29:15				
Control	38 OW/OB (6 M, 32 W)	53 (10)		178.9 (23.9)	30.2 (3.4)							NNS	Sugar free hot cocoa beverage		~48:35:17				
**Sanchez-Delgado et al. 2021**	38H (11M, 27W)		OP, Mexico			NR			P	Supp	Y					Positive	6 wk	NR	NR
Intervention	12 H (3M, 9W)	22(4)		59.4 (9.1)	22.5 (2.4)		37.6 (29.9)	50.4 (3.8)				Sucrose	Sucrose 8x5g/day	40 (9)	49:31:18				

Control	13 H (3M, 10W)	22(4)		57.4 (8.1)	22.2 (1.8)		32.5 (8.3)	50.4 (2.9)				NNS	Sucralose 4x1g/day		45:35:20				
Control	13 H (5M, 8W)	24(5)		59.1 (6.6)	21.8 (1.9)		30.7 (17.3)	49.4 (5)				NNS	Steviol glycosides 4x1g/day		45:33:22				
**Vaz et al. 2011**	192 H		OP, India		NR	0.3 (0-0.8)	NR	NR	P	Supp	Y					Positiv e	17 wk	I	NR
Intervention	95 H	8 (1.0)		21.9 (3.4)								Sucrose	Fortified choco-malt beverage	~28 (5.6)	58:17:9				
Intervention	95 H	8 (1.0)		21.2 (3)								Sucrose	Unfortified choco-malt beverage	~28 (5.6)	67:22:11				
Control	97 H	8 (1.0)		21.5 (3)								Diet alone	No beverage		66:23:11				
**Zafrilla et al. 2021**	136 OW (80M, 56W)		OP, Spain			NR		NR	P	Supp	Y		Each group received citris juices and maqui extract but with diff sweeteners		NR	Positiv e	9 wk	A	N
Intervention	45 OW (26M, 19W)	42 (7)		84.8 (12.4)	29.1 (3)		0.8 (0.4)					Sucrose	7.5g/100mL and drank 330 mL/day	24.8 (5)					
Control	46 OW (27M, 19W)	44 (7)		82.9 (10.8)	27.9 (2.9)		0.8 (0.5)					Stevia	4mg/100mL and drank 330 mL/day						
Control	45 OW (27M, 18W)	42 (8)		82.4 (10.8)	27.8 (2.3)		0.5 (0.6)					Sucralose	4mg/100mL and drank 330 mL/day						
**Sweetened Dairy**																			
**Ellis et al. 2011**	12 OW/OB	51 (15)	OP, USA	86.6 (12.9)	29.2 (2.3)	3.0 (0.2)	2.1 (0.0)	1.2 (0.1)	C	Supp	N				NR	Positiv e		A, I	N
Intervention												Sucrose	Freeze dried strawberry beverage (swe etened milk)	~2.9(0. 6)			6 wk		

Intervention												Sucrose	Freeze dried strawberry beverage (sweetened milk)	~2.9(0.6)			6 wk		
Control												Diet alone	No beverage				1 wk		
**100% Fruit Juice**																			
**Aghababaee et al. 2015**	72 hyperlipidemia (54M, 18W)		OP, Iran				NR	NR	P	Supp	Y					Positive	8 wk	A	N
Intervention	36 hyperlipidemia	45 (8)		85.3 (16.7)	29.2 (4.5)	1.0 (0.6)						Fruit	300 mL blackberry juice with pulp	~24.4 (3.6)	58:28:14				
Control	36 hyperlipidemia	46 (9)		78.9 (9.8)	28.0 (3.4)	1.3 (0.7)						Diet alone	No blackberry juice		58:29:13				
**Asgary et al. 2014**	21 hypertensive (6 M, 15 W)		OP, Iran					NR	P	Supp	N				NR	Positive	2 wk	A	N
Intervention	11 hypertensive (3 M, 8 W)	59 (5)		68.0 (10.1)	26.8 (3.5)	1.8 (1.6)	7.5 (4.8)					Fruit	150 mL/d natural pomegranate juice	~19.9(4)					
Control	10 hypertensive (3 M, 7 W)	47(12)		73.5 (6.0)	28.0 (4.1)	1.0 (0.5)	6.7 (3.8)					Water	150 mL/d water						
**Castilla et al. 2008**	16 hypolipidemic	NR	OP, Spain	NR	NR		NR	NR	P	Supp	Y				50:35:15	Positive	2 wk	A	N
Intervention	8 hypolipidemic					6.0 (5.1)						Fruit	100 mL/d red grape juice	~7.9(1.6)					
Control	8 hypolipidemic					10.4 (9.6)						Diet alone	No red grape juice						
**Castilla et al. 2008** – Vitamin E	16 hypolipidemic	NR	OP, Spain	NR	NR		NR	NR	P	Supp	Y				50:35:15	Positive	2 wk	A	N
Intervention	8 hypolipidemic					12.7 (10.7)						Fruit	100 mL/d red grape juice with Vitamin E	~7.9(1.6)					

Control	8 hypolipidemic					13.0 (9.6)						Diet alone	No red grape juice, plus Vitamin E						
**Fatel et al. 2021**	41 RA (9M, 32 W)		OP, Brazil					NR	P	Supp	Y				NR	Positive	13 wk	A, I	N
Intervention	20 RA (5M, 15W)	57 (14)		66.3 (22.1)	27.7 (7.5)	4.4 (4.6)	3.3 (3.6)					Fruit	500 ml/d reduced calorie cranberry juice + 3g/d fish oil	12.5 (2.5)					
Control	21 RA (4M, 17W)	56 (14)		67.0 (19.6)	27.6 (6.8)	3.7 (2.9)	7.9 (10.0)					Diet alone	3 g/d fish oil						
**Karlsen et al. 2010**	62 H but at elevated risk for CVD		OP, Norway						P	Supp	Y				NR	Positive	4 wk	A	N
Intervention	31 H (21M, 10W)[h]	53 (34-68)		79.4 (58-105)	25.6 (19.9-31.7)	1.3 (1.3)	5.6 (57.8)	21.2 (63.2)				Fruit	330 mL/d bilberry juice	~39(7.8)					
Control	31 H	53 (30-68)		81.3 (56-112)	25.5 (17.8-31.5)	1.0 (0.9)	5.6 (33.5)	11.5 (28.6)				Water	1 L water/d[j]						
**Leelarungrayub et al. 2016**	29H (20M, 9W)	72 (9)	OP, Thailand	52.4 (8.2)	NR	NR	NR		C	Supp	N				NR	Positive		A	N
Intervention								8.0 (1.2)				Fruit	200g/d star fruit juice	8.0 (1.6)			4wk		
Control								7.7 (0.2)				Diet alone	No star fruit juice				2wk		
**Ravn-Haren et al. 2013**	23 H (9 M, 14 W)	36 (18)	OP, Denmark	NR	22.3 (2.6)	NR	NR	NR	C	Supp	Y				NR	Positive	4wk	A	N
Intervention												Fruit	500 mL/d polyphenolic and pectin restricted diet with clear apple juice	63 (~12.6)					
Intervention												Fruit	500 mL/d polyphenolic and pectin restricted diet with cloudy apple juice	51(~11.8)					

Control												Fruit	22g/d polyphenolic and pectin restricted diet with apple pomance	2.1(~0.0)					
Control												Diet alone	Polyphenolic and pectin restricted diet						
**Simao et al. 2013**	56 MetS (14M, 42W)		OP, Brazil	NR					P	Supp	N				NR	Positive	9 wk	A, I	N
Intervention	20 MetS (6M, 14W)	51 (42-53)			30.9 (26.3-38.4)	56.9 (74.5)	2.3 (0.7)	1.9 (1.9)				Fruit	0.7 L/d cranberry juice (reduced energy)	~29.2 (5.8)[i]					
Control	36 MetS (8M, 28W)	49 (45-56)			34.0 (31.3-36.9)	54.7 (57.1)	2.3 (0.7)	2.4 (2.6)				Diet alone	No cranberry juice	~17.5 (3.5)					
**Thimoteo et al. 2019**	38 RA (0M, 38W)		OP, Brazil	NR		4.9 (5.9)	NR	NR	P	Supp	Y				NR	Positive	13 wk	A, I	Y
Intervention	20 RA (0M, 20W)	55 (51-65)			26 (23-30)							Fruit	Reduced-calorie cranberry juice 500mL/day	~12.5 (2.5)					
Control	18 RA (0M, 18W)	50.5 (40-60)			30 (22-32)							Diet alone	No cranberry juice						
**Fruit**																			
**Basu et al. 2010**	48 MetS (4 M, 44 W)	50 (21)	OP, USA	NR	37.8 (15.9)	NR	NR	NR	P	Supp	Y				NR	Positive	8 wk	A, I	N
Intervention	25 MetS (2 M, 23 W)	52 (15)			38.1 (7.5)							Fruit	50 g/d free-dried blueberries	~30 (~6)					
Control	23 MetS (2 M, 21 W)	48 (16)			37.5 (14.4)							Water	Water						
**Blum et al. 2007**	103 H (35M, 68W)		OP, Israel	NR			NR	NR	P	DA	Y				NR	Positive	4 wk	I	NR
Intervention	50 H (17M, 33W)	46 (14)			28.1 (3.2)	0.4 (0.6)						Fruit	300 g/d tomatoes	~7.9(1.6)					

Control	53 H (18M, 35W)	45 (14)			30.1 (1.5)	0.5 (0.5)						Diet alone	No tomatoes						
**Dow et al. 2013**	69 OW/OB (21 M, 48 W)	42 (11)	OP, USA	91.5 (13.9)	32.1 (4.1)		NR	NR	P	Supp	Y					Positive	6 wk	A	N
Intervention	37 OW/OB (12 M, 25 W)[k]	41 (11)		92.1 (15.0)	32.8 (4.2)	2.2 (1.5)						Fruit	0.5 fresh red grapefruit/d 3 times a day (so 1.5 grapefruits)	~24 (4.8)	~50:34:16				
Control	32 OW/OB (9 M, 23 W)[m]	43 (11)		90.8 (12.8)	31.4 (3.8)	2.4 (2.0)						Diet alone	No grapefruit		~48:37:16				
**Franck et al. 2020**	48 risk of MetS (16M, 32W)		OP, Canada	NR			NR	NR	P	Supp	Y					Positive	8 wk	I	N
Intervention	24 (7M, 17W)	33 (10)			30.4 (5)	2.7 (2.3)						Fruit	280g frozen raspberries (2 cups)/d	12.4 (2.4)	48:35:17				
Control	24 (9M, 15W)	32 (8)			29.4 (3.9)	2.7 (2.8)						Diet alone	Usual diet, no supplementation		42:40:18				
**Kelley et al. 2013**	18 H (2 M, 16 W)	50 (45-61)	OP, USA		26.3 (3.8)	NR	NR	NR	C	Supp	N			NR		Neutral		A, I	NR
Intervention												Fruit	Sweet bing cherries		50:33:16		4 wk		
Control												Diet alone	No cherries		55:32:14		1 wk		
**Liddle et al. 2021**	44 OW/OB (14M, 30W)		OP, Canada						P	Supp	Y					Positive	6 wk	A, I	N
Intervention	22 OW/OW (7 M, 15 W)	43 (19-69)		94.9 (23)	33.2 (6.1)	3.0 (0.9)	1.4 (0.4)	6.7 (1.3)				Fruit	3 whole small Gala apples (200g edible parts)	17.5 (3.2)	49:34:16				
Control	22 OW/OB (7 M, 15 W)	48 (24-69)		96.6 (18.8)	33.3 (5.6)	3.1 (1.2)	1.2 (0.6)	6.2 (1.3)				Diet alone	No apples		43:40:17				

**Ravn-Haren et al. 2013**	23 H (9 M, 14 W)	36 (18)	OP, Denmark		22.3 (2.5)	NR	NR	NR	C	Supp	Y				NR	Positive	4 wk	A	N
Intervention												Fruit	Polyphenolic and pectin restricted diet with 550 g whole apples/d	~51 (10)					
Control												Fruit	Polyphenolic and pectin restricted diet with 22 g apple pomace/d	2.1 (~0.0)					
Control												Diet alone	Polyphenolic and pectin restricted diet with apple pomace						
**Schell et al. 2019**	22 T2D	54 (21)	OP, USA	104 (55)	35.3 (10)				C	Supp	Y					Positive	4 wk	A, I	Y
Intervention						5.1 (5.6)	12.5 (31.0)	5.5 (7.0)				Fruit	250 g frozen raspberries per day (pureed)	~11.1 (2.2)	48:34:17				
Control						5.3 (5.6)	8.5 (19.2)	4.4 (11.7)				Diet alone	Continue typical diet		45:37:16				
**Dried Fruit**																			
**Hooshmand et al. 2016**		71 (3)	OP, USA	65.0 (10.6)	25.0 (4.3)		NR	NR	P	Supp	Y					Positive	24 wk	A, I	N
Intervention	13 osteopenic (0 M, 13W)					1.7 (0.4)						Fruit	100 g/d dried plum	~38.1 (8.6)	53:37:16				
Intervention	13 osteopenic (0 M, 13W)					1.8 (0.4)						Fruit	50 g/d dried plum	~19.1 (3.8)	48:34:14				
Control	16 osteopenic (0 M, 16 W)					1.6 (0.2)						Diet alone	No dried plums		51:32:15				
**Irannejad et al. 2020**	72 T2D (21 M, 51 W)		OP, Iran				NR	NR	P	Supp	Y					Positive	12 wk	A	N

Intervention	36 T2D (8 M, 28 W)	53 (8)		76 (13)	29 (4)	32(16.6)						Fruit	30g/d z. vulgaris	14.3(3.8)	70:18:16				
Control	36 T2D (13 M, 23 W)	57 (6)		75 (11)	28 (4)	26.6(14)						Diet alone	Diet alone		69:17:15				
**Sweetened Cereal Grains and Bars**																			
**Mietus-Snyder et al. 2012**	25 H (10M, 15W)	45 (16)	OP, USA	NR	25.7 (14.3)	1.8 (2.2)	NR	NR	C	Supp	N				NR	Positive	2 wk	A, I	N
Intervention												Mixed type	2 servings/d nutrition bars (blueberry, cranberry, red grape, dried plum concentrates and chocolate)	~15.2 (3)					
Control												Diet alone	No nutrition bar						
**Sweets and Desserts**																			
**Alavinejad et al. 2015**	42 NAFLD (35M, 9W)		OP, Iran				NR	NR	P	Supp	Y				NR	Positive	12 wk	A, I	N
Intervention	21 NAFLD (19M, 3W)	38 (11)		88.6 (13.2)	30.3 (3.6)	1.4 (4.7)						Sucrose	Dark Chocolate	~6.1 (1.2)					
Control	21 NAFLD (16M, 6W)	38 (10)		84.9 (20.6)	29.7 (5.8)	2.0 (9.2)						Fat	White Chocolate						
**Jafarirad et al. 2018**	44 T2D (17M, 27W)	52 (6)	OP, Iran	NR	NR				P	Supp	Y					Positive	8 wk	A	N
Intervention	21 T2D (7M, 14W)	51 (8)				4.2 (1.3)	6.6 (3.8)	34.3 (21.8)				Sucrose	84% dark chocolate	5.5 (1.1)	56:27:18				
Control	23 T2D (10M, 13W)	51 (8)				3.2 (1.3)	4.2 (2.2)	24.6 (10.2)				Diet alone	no chocolate		58:23:19				
**Martini et al. 2020**	21 NW (10M, 11W)	23 (2)	OP, Italy	67 (12.4)	22.3 (7.8)	NR	NR	NR	C	Supp	Y					Positive	4 wk	I	N

Intervention												Sucrose	1 cup espresso + 2 cocoa-based products containing coffee/d	43.4 (8.2)	47:37:16				
Control												Water	1 cup espresso/d		49:38:13				
**Added Sweeteners**																			
**Ghazali et al. 2017**	64 smokers	38 (8)	OP, Malaysia	NR	NR				P	Supp	Y				NR	Positiv e	12 wk	A	NR
Intervention						2.3 (1.8)	1.6 (1.1)	2.6 (8.2)				Honey	20 g/d Malaysian Tualang honey	~16.4 (3.3)					
Control						2.9 (13.5)	2.0 (1.5)	3.0 (0.7)				Diet alone	No honey						
**Pothasak et al. 2020**	60 COPD (31M, 29W)		OP, Thailand			NR	NR		P	Supp	Y				NR	Positiv e	4 wk	A	N
Intervention	20 COPD (8M, 12W)	71 (27)		50.7 (12.4)	21.7 (6.1)			10 (1.5)				Honey	10g/d starfruit honey mixed in 150mL water	8.2 (1.6)					
Intervention	15 COPD (8M, 7W)	64 (6)		51.6 (13.5)	20.9 (4.6)			10.5 (5.3)				Honey (Ex)	10g/d starfruit honey mixed in 150mL water with 3x 30min/wk walking exercise	8.2 (1.6)					
Control	10 COPD (5M, 5W)	69 (4)		56.5 (11.8)	23.5 (7.2)			10.3 (1.6)				Diet alone	No honey						
Control	15 COPD (10M, 5W)	61 (13)		58.4 (17.1)	20.5 (4.4)			11.4 (2.8)				Diet alone (Ex)	No honey with 3x 30min/wk walking exercise						
**Subtraction Trials**																			
**Food Source:**																			
**SSB**																			
**Campos et al. 2015 (IHCL**	12 OW/OB	28(7)	OP, Switzerl and				NR	NR	P	Supp	Y					Negati ve	12 wk	A	NR

**<60mmol/L)**																			
Intervention	6 OW/OB			85.6 (11.3)	30.1(4. 9)	2.3(1.1)						NNS	Replace SSB with NSB	~86.8 (15)	46:38:16				
Control	6 OW/OB			78.5 (7.1)	26.9(1. 2)	4.4(4.2)						Sucrose	Habitual SSB intake (SSB and sugar sweetened tea)		51:34:15				
**Campos et al. 2015 (IHCL >60mmol/L)**	15 OW/OB	29 (7)	OP, Switzerland				NR	NR	P	Supp	Y					Negative	12 wk	A	NR
Intervention	8 OW/OB			102.2 (12.2)	32.5 (4.5)	4.8 (4.4)						NNS	Replace SSB with NSB	~86.8 (15)	46:38:16				
Control	7 OW/OB			100.0 (11.6)	33.8 (5.6)	2.1 (0.7)						Sucrose	Habitual SSB intake (SSB and sugar sweetened tea)		51:34:15				
**Ebbeling et al. 2020**	120 MW (72M, 48W)[h]		OP, USA				NR	NR	P	Supp	Y				NR	Negative	48 wk	A, I	N
Intervention	60 MW (36M, 24W)[h]	27 (6)[f]		76.8 (16.7)[f]	26.1 (5.2)[f]	1.2 (1.5)						NNS	substitute all SSB with ASB						
Intervention	66 MW (39M, 27W)[h]	28 (6)[f]		77.5 (16.1)[f]	26.6 (4.6)[f]	0.9 (0.7)						water	substitute all SSB with water						
Control	60 MW (36M, 24W)[h]	26 (5)[f]		75.5 (15.6)[f]	25.8 (4.7)[f]	1.2 (1.5)						HFCS	SSB (usual intake)	84.5 (15.3)					
**Ad libitum Trials**																			
**Food Source:**																			
**Mixed Sources (with SSBs)**																			
**Markey et al. 2016**	50 H (16M, 34W)	32 (10)	OP, UK	69.8 (11.4)	24.0 (3.3)		NR	NR	C	Supp	Y					Neutral	8 wk	I	N

Intervention	22 H (7M, 15F)			70.1 (11.3)	24.1 (3.3)	1.1 (1.4)						Sucrose	Exchange ≥1 food portion and ≥1 beverage per day from habitual diet with sugar containing products	~30 (6)	54:30:14				
Control	28 H (9M, 19F)			69.8 (11.5)	24.0 (3.4)	0.9 (0.9)						Sweetener	Exchange ≥1 food portion and ≥1 beverage per day from habitual diet with sugar reformulated products		48:33:15				
**Munsters et al. 2010**	29 OW/OB (14M, 15W)	39 (2)	OP, Netherlands			3.0 (2.9)	NR	NR	P	Supp	Y					Neutral	24 wk	A	NR
Intervention	14 OW/OB (7M, 7W)	40 (2)		85.3 (9.4)	28.6 (1.5)							Mixed type	Simple carbohydrate diet	~126 (19)	42:40:15				
Control	15 OW/OB (7M, 8W)	39 (2)		83.2 (9.3)	28.3 (1.5)							Mixed comparator	Complex carbohydrates		45:39:13				
Control	18 OW/OB (10M, 8W)	37 (2)		88.5 (11.5)	29.9 (3.0)							Mixed comparator	Control diet		45:39:14				

%C=percent carbohydrate; %E=percentage of total energy intake; %F=percent fat; %P=percent protein; A=agency funding; B=boy; BMI=body mass index; BW=body weight; C=crossover trial; Ca=calcium; CKD=chronic kidney disease; COPD=chronic obstructive pulmonary disease; CVD=cardiovascular disease; d=day; DA=dietary advice; EB=energy balance; F=female; G=girl; H=healthy (mixed weight); HFCS=high-fructose corn syrup; HI=hyperinsulinemic; HTN=hypertension; I=industry funding; IBW=ideal body weight; IGT=impaired glucose tolerance; IHCL=intrahepatocellular lipid; IP=in-patient setting; M=male; Met=metabolic diet; mo=month; N=no; NAFLD=non-alcoholic fatty liver disease; NNS=non-nutritive sweetener; NR=not reported; NSB=non-nutritive sweetened beverage; OffT2D=offspring of type 2 diabetic parents; OP=out-patient setting; OW/OB= overweight/obese body mass index; P=parallel trial; RA= Rheumatoid Arthritis; SD=standard deviation; SF=stone former; SSB=sugar-sweetened beverage; Supp=supplemented diet; T1DM=type 1 diabetes mellitus; T2D=type 2 diabetes mellitus; UK=United Kingdom; USA=United States of America; wk(s)=week(s); Y=yes ; yr=year; CRP= C reactive protein, TNF-α = tumour necrosis factor alpha, IL-6= interleukin 6.

[a]Metabolic feeding control included provision of all study foods, supplement feeding control included provision of study supplements only, and dietary advice included dietary counselling without the provision of any dietary foods or supplements.

[b]Dose preceded by "~" represent approximates calculated on the basis of average energy intake reported by participants. In the absence of this data, an average of 2000 kcal/d was assumed.

[c]Total energy intake in the form of carbohydrate:fat:protein

[d]Positive energy balance included interventions designed to consume excess calories on top of a baseline diet. Negative energy balance included interventions designed to create a calorie deficit compared to the baseline diet. Neutral energy balance included interventions designed to continue habitual caloric intake.

[e]Agency funding included government, not-for-profit health agencies or University sources.

[f]Data measured in N=32 participants at baseline.
[g]Measured using values taken from : https://fineli.fi/fineli/en/elintarvikkeet/442?q=bilberr&foodType=ANY&portionUnit=G&portionSize=100&sortByColumn=points&sortOrder=asc&component=2331
[h]18 participants out of the intervention group were evaluated for CRP and included in the present analysis
[i]Fructose-containing sugars dose estimated from: https://www.oceanspray.com/Products/Juices/By-Type/Classic-Juice-Drinks/Cranberry-Juice-Cocktail.
[j]Fructose-containing sugars dose estimated from: https://www.pomefresh.com/products/pomefresh-100-organic-bilberry-juice-1l
[k/m]14/15 participants respectively in intervention and control were diagnosed with metabolic syndrome

Supplemental Table S6: Sensitivity analyses of the use of correlation coefficients of 0·25 and 0·75 for crossover trials in the primary analysis of the effect of important food sources of fructose-containing sugars on biomarkers of inflammation outcomes

**Inflammation Outcome**	**MD [95% CI], P_{MD}<br>I^2, P_Q**		
	**Correlation Coefficient used in the Primary Analysis**	**Correlation Coefficient used in Sensitivity Analyses**	
**Energy Design and Food Source (N crossover trials/total)**	**0·5**	**0·25**	**0·75**
**CRP (mg/L)**			
**Substitution (15/37)**	**0.074[-0.077, 0.224], P_{MD}=0.336 I^2=53.70%, P_Q<0.001**	**0.080[-0.053, 0.214], P_{MD}=0.24 I^2=53.76%, P_Q<0.001**	**0.055[-0.06, 0.167], P_{MD}=0.34 I^2=54.65%, P_Q<0.001**
SSB (6/10)	0.07 [-0.16, 0.30] , P_{MD}=0.543 I^2=0.00%, P_Q=0.450	0.096 [-0.17, 0.36] , P_{MD}=0.47 I^2=0.00%, P_Q=0.468	0.045 [-0.12, 0.212] , P_{MD}=0.59 I^2=0.79%, P_Q=0.431
Sweetened dairy (0/3)	-0.06 [-0.51, 0.39] , P_{MD}=0.802 I^2= 0.00%, P_Q=0.63	NA	NA
Sweetened dairy alternative (soy) (0/1)	-0.96 [-1.67, -0.25] , P_{MD}=0·008 I^2= .%, P_Q= .	NA	NA
100% Fruit juice (0/2)	-1.09 [-2.01, -0.17] , P_{MD}=0·021 I^2= 0.00%, P_Q=0.591	NA	NA
Fruit (2/5)	-0.43 [-0.87, 0.01] , P_{MD}=0.055 I^2=34.3%, P_Q=0.193	-0.43 [-0.89, 0.02] , P_{MD}=0.060 I^2=34.31%, P_Q=0.193	-0.43 [-0.85, -0.01] , **P_{MD}=0.045** I^2=34.33%, P_Q=0.192
Dried fruit (1/4)*	0.21 [-0.14, 0.55] , P_{MD}=0.240 I^2=0.00%, P_Q=0.683	NA	NA
Mixed fruit forms (0/1)	-0.10 [-1.20, 1.00] , P_{MD}=0.859 I^2= .%, P_Q=.	NA	NA
Added nutritive (caloric) sweeteners (1/1)	-0.03 [-0.11, 0.05] , P_{MD}=0.464 I^2= .%, $P_{Q=.}$	-0.03 [-0.12, 0.06] , P_{MD}=0.523 I^2= .%, $P_{Q=.}$	-0.03 [-0.095, 0.035] , P_{MD}=0.363 I^2= .%, $P_{Q=.}$
Mixed sources (with SSBs) (2/6)*	0.643 [0.118, 1.168] , P_{MD}=0.016 I^2=82.91%, P_Q<0.001	NA	NA
Mixed sources (without SSBs) (3/4)	0.29 [-0.21, 0.78] , P_{MD}=0.26 I^2=0.00%, P_Q=0.78	0.19 [-0.24, 0.63] , P_{MD}=0.38 I^2=0.00%, P_Q=0.66	0.063 [-0.204, 0.33] , P_{MD}=0.64 I^2=0.00%, P_Q=0.52
**Addition (13/37)**	**-0.18 [-0.33, -0.028], P_{MD}= 0.020 I^2=43.67%, P_Q=0.003**	**-0.202 [-0.36, -0.045], P_{MD}= 0.012 I^2=41.88%, P_Q=0.0046**	**-0.15 [-0.29, -0.003], P_{MD}=0.045 I^2=47.69%, P_Q=0.0008**

SSB (4/7)	0.02 [-0.15, 0.19] , P_{MD}=0.79 I^2=30.88%, P_Q=0.19	0.013 [-0.16, 0.19] , P_{MD}=0.88 I^2=28.38%, P_Q=0.222	0.04 [-0.11, 0.2] , P_{MD}=0.59 I^2=36.70%, P_Q=0.15
Sweetened dairy alternative (soy) (0/2)	0.20 [-0.78, 1.18] , P_{MD}=0.689 I^2=52.0%, P_Q=0.149	NA	NA
100% Fruit juice (4/12)	-0.12 [-0.53, 0.295] , P_{MD}=0.58 I^2=49.29%, P_Q=0.03	-0.15 [-0.59, 0.285] , P_{MD}=0.5 I^2=46.41%, P_Q=0.039	-0.071 [-0.45, 0.305] , P_{MD}=0.71 I^2=54.62%, P_Q=0.012
Fruit (4/9)	-0.50 [-0.75, -0.25] , P_{MD}=0.000 I^2=0.00%, P_Q=0.960	-0.52 [-0.77, -0.27] , P_{MD}=0.000 I^2=0.00%, P_Q=0.978	-0.45 [-0.68, -0.23] , P_{MD}=0.000 I^2=0.00%, P_Q=0.892
Dried fruit (0/3)	0.02 [-0.75, 0.79] , P_{MD}=0.962 I^2=0.00%, P_Q=0.575	NA	NA
Sweetened cereal grains & bars (1/1)*	-0.30 [-0.79, 0.19] , P_{MD}=0.228 I^2= .%, P_Q=.	NA	NA
Sweets and desserts (0/2)	-0.67 [-1.85, 0.50] , P_{MD}=0.26 I^2=86.8%, P_Q=0.006	NA	NA
Added nutritive (caloric) sweetener (0/1)	0.56 [-4.19, 5.31] , P_{MD}=0.817 I^2= .%, P_Q=.	NA	NA
**Subtraction (0/4)**	**0.14 [-0.29, 0.56], P_{MD}=0.522 I^2=0·00%, P_Q=0.877**	NA	NA
SSB (0/4)	0.14 [-0.29, 0.56], P_{MD}=0.522 I^2=0·00%, P_Q=0.877	NA	NA
**Ad Libitum (1/3)***	**-0.09 [-0.44, 0.25], P_{MD}=0.604 I^2=0·00%, P_Q=0.910**	NA	NA
SSBs (and other food sources) (1/3)*	-0.09 [-0.44, 0.25] , P_{MD}=0.604 I^2=0·00%, P_Q=0.910	NA	NA
**TNF-α (pg/mL)**			
**Substitution (5/17)**	**0.04 [-0.11, 0.19], P_{MD}=0.61 I^2=52.55%, P_Q=0.006**	**0.042 [-0.107, 0.192], P_{MD}=0.58 I^2=51.51%, P_Q=0.007**	**0.033 [-0.12, 0.185], P_{MD}=0.67 I^2=55.32%, P_Q=0.003**
SSB (0/2)	-0.028 [-0.18, 0.13] , P_{MD}=0.72 I^2=45.68%, P_Q=0.175	NA	NA
Fruit (1/3)*	-0.15 [-0.39, 0.08] , P_{MD}=0.206 I^2=0·00%, P_Q=0.455	NA	NA
Dried Fruit (1/5)*	0.03 [-0.49, 0.55] , P_{MD}=0.920 I^2=65.5%, P_Q=0.021	NA	NA
Mixed fruit forms (0/1)	0.20 [-0.34, 0.74] , P_{MD}=0.467	NA	NA

	I^2= .%, P_Q=.		
Mixed sources (with SBBs) (2/4)	0.35 [-0.69, 1.39] , P_{MD}=0.51 I^2=69.16%, P_Q=0.02	0.382 [-0.66, 1.42] , P_{MD}=0.47 I^2=61.63%, P_Q=0.05	0.32 [-0.7, 1.33] , P_{MD}=0.54 I^2=77.59%, P_Q=0.004
Mixed sources (without SSBs) (1/2)*	0.15 [-0.01, 0.32] , P_{MD}=0.059 I^2=0·00%, P_Q=0.83	NA	NA
**Addition (5/16)**	**-0.48 [-0.99, 0.04], P_{MD}=0.069 I^2=89.6%, P_Q<0.001**	**-0.47 [-0.90, -0.03], P_{MD}=0.04 I^2=83.38%, P_Q<0.001**	**-0.46 [-1.10, 0.18], P_{MD}=0.16 I^2=94.76%, P_Q<0.001**
SSB (0/3)	0.79 [-1.23, 2.80] , P_{MD}=0.444 I^2=58.6%, P_Q=0.089	NA	NA
Sweetened dairy (0/2)	-0.10 [-0.30, 0.10] , P_{MD}=0.336 I^2=0·00%, P_Q=1.000	NA	NA
100% Fruit juice (1/3)	-0.65 [-2.57, 1.27] , P_{MD}=0.507 I^2=95.6%, P_Q=0.000	-0.65 [-2.58, 1.27] , P_{MD}=0.508 I^2=95.72%, P_Q=0.000	-0.66 [-2.73, 1.41] , P_{MD}=0.532 I^2=97.45%, P_Q=0.000
Fruit (2/3)*	-0.89 [-1.58, -0.20] , P_{MD}=0.012 I^2=14.3%, P_Q=0.311	NA	NA
Sweets and desserts (1/2)	-4.66 [-16.21, 6.90] , P_{MD}=0.43 I^2=77.3%, P_Q=0.040	-4.68 [-16.24, 6.88] , P_{MD}=0.43 I^2=76.94%, P_Q=0.04	-4.63 [-16.18, 6.91] , P_{MD}=0.43 I^2=77.71%, P_Q=0.03
Added nutritive (caloric) sweetener (0/3)	-0.91 [-2.18, 0.36] , P_{MD}=0.162 I^2= 2.00%, P_Q=0.361	NA	NA
**IL-6 (pg/mL)**			
**Substitution (6/16)**	**-0·043 [-0.24, 0.15], P_{MD}=0.663 I^2=22.17%, P_Q=0.202**	**-0·035 [-0.23, 0.16], P_{MD}=0.726 I^2=22.49%, P_Q=0.198**	**-0·04 [-0.215, 0.13], P_{MD}=0.64 I^2=23.64%, P_Q=0.19**
SSB (2/5)	-0.096 [-0.49, 0.3] , P_{MD}=0.634 I^2=0.0%, P_Q=0.798	-0.1 [-0.575, 0.375] , P_{MD}=0.68 I^2=0.0%, P_Q=0.8	-0.09 [-0.375, 0.19] , P_{MD}=0.53 I^2=0.0%, P_Q=0.8
Fruit (1/4)*	0.16 [-0.83, 1.16] , P_{MD}=0.748 I^2=46.1%, P_Q=0.135	NA	NA
Dried fruit (1/3)*	0.30 [-0.73, 1.34] , P_{MD}=0.566 I^2=71.9%, P_Q=0.028	NA	NA
Mixed (with SSBs) (0/1)	0.02 [-0.301, 0.34] , P_{MD}=0.903 I^2=.%, P_Q=.	NA	NA
Mixed (without SSBs) (2/3)	0.18 [-0.201, 0.57] , P_{MD}=0.35 I^2=0.0%, P_Q=0.66	0.17 [-0.19, 0.525] , P_{MD}=0.35 I^2=0.0%, P_Q=0.66	0.15 [-0.16, 0.47] , P_{MD}=0.346 I^2=0.0%, P_Q=0.646
**Addition (1/16)***	**-0·15 [-0.45, 0.16], P_{MD}=0.352 I^2=82.90%, P_Q=0.000**	NA	NA
SSB (0/5)	0.06 [-0.34, 0.46] , P_{MD}=0.776	NA	NA

	I^2=29.0%, P_Q=0.229		
Sweetened dairy (0/2)	-0.52 [-1.12, 0.08] , P_{MD}=0.087 I^2=46.6%, P_Q=0.171	NA	NA
100% Fruit juice (0/4)	-3.014 [-6.91, 0.88] , P_{MD}=0.13 I^2=74.08%, P_Q=0.009	NA	NA
Fruit (1/3)*	-0.19 [-0.59, 0.21] , P_{MD}=0.352 I^2=86.5%, P_Q=0.001	NA	NA
Sweets and desserts (0/1)	-8.79 [-14.26, -3.32] , P_{MD}=0.002 I^2= .%, P_Q= .	NA	NA
Added nutritive (caloric) sweetener (0/1)	0.40 [-0.22, 1.02] , P_{MD}=0.207 I^2= .%, P_Q= .	NA	NA

CRP=C reactive protein; IL-6=Interleukin-6; NA, not applicable; TNF-α= tumour necrosis factor-alpha; SSB=sugar sweetened beverages

*For one or more crossover trial a correlation coefficient was not used since a p-value was provided for the mean difference between treatment changes.

Supplemental Table S7: GRADE certainty of evidence assessment* for the effect of fructose-containing sugars on biomarkers of inflammation by energy control

Outcome and trial (N)	Design	GRADE assessment: Downgrades: Risk of bias (ROB)	Inconsistency	Indirectness	Imprecision	Publication bias	Upgrades: Dose response	Effect (MD [95% CI], P_{MD})		Certainty of Evidence[a]	Interpretation of magnitude of effect[b]
**CRP (mg/L)**											
Substitution (37)	Randomized and non-randomized trials	Not serious	Not serious[1]	Very serious[2]	Not serious	None	None	↔	0.07 [-0.08 to 0.22], P=0.336	⊕⊕◯◯ Low	No effect
Addition (37)	Randomized and non-randomized trials	Not serious	Not serious	Very serious[3]	Not serious	None	None	↓	-0.18 [-0.33 to -0.03], P=0.020	⊕⊕◯◯ Low	Trivial
Subtraction (4)	Randomized trials	Not serious	Not serious	Very serious[4]	Serious[5]	None[6]	None[7]	↔	0.14 [-0.29 to 0.56], P=0.522	⊕◯◯◯ Very low	No effect
Ad libitum (3)	Randomized trials	Not serious	Not serious	Very serious[4]	Not serious	None[6]	None[7]	↔	-0.09 [-0.44 to 0.25], P=0.604	⊕⊕◯◯ Low	No effect
**TNF-α (pg/mL)**											
Substitution (17)	Randomized and non-randomized trials	Not serious	Not serious[8]	Not serious	Not serious	None	None	↔	0.04 [-0.11 to 0.19], P=0.610	⊕⊕⊕⊕ High	No effect
Addition (16)	Randomized and non-randomized trials	Not serious	Not serious[9]	Very serious[3]	Serious[10]	None	None	↔	-0.48 [-0.99 to 0.04], P=0.069	⊕◯◯◯ Very low	No effect
**IL-6 (pg/mL)**											
Substitution (16)	Randomized and non-randomized trials	Not serious	Not serious	Not serious	Serious[11]	None[12]	None	↔	-0.04 [-0.24 to 0.15], P=0.663	⊕⊕⊕◯ Moderate	No effect
Addition (16)	Randomized and non-randomized trials	Not serious	Serious[13]	Very serious[2]	Serious[11]	None[14]	None	↔	-0.15 [-0.45 to 0.16], P=0.352	⊕◯◯◯ Very low	No effect

[a] Since all included trials were randomized or non-randomized controlled trials, the certainty of the evidence was graded as high for all outcomes by default and then downgraded or upgraded based on pre-specified criteria. Criteria for downgrades included risk of bias (ROB) (downgraded if the majority of trials were considered to be at high ROB); inconsistency (downgraded if there was substantial

unexplained heterogeneity [$I^2 \geq 50\%$, $P_Q < 0.10$]; indirectness (downgraded if there were factors absent or present relating to the participants, interventions, or outcomes that limited the generalizability of the results); imprecision (downgraded if the 95% confidence interval crossed the minimally important difference [MID] for harm or benefit set at 0.5 mg/L for CRP (Reynolds Risk Score. Available at: http://www.reynoldsriskscore.org/Default.aspx [Accessed March 14, 2018]; Ridker, P.M. et al., 2008. Circulation, 118(22), pp.2243–51; Ridker, P.M. et al., 2007. JAMA 297(6), pp.611–619.), 0.28 pg/mL for TNF-α (Mayoclinic. Tumour Necrosis Factor (TNF), Plasma. Available from: https://www.mayocliniclabs.com/test-catalog/Clinical+and+Interpretive/63022), 0.18 pg/mL for IL-6 (Mayoclinic. Interleukin 6, Plasma. Available from: https://www.mayocliniclabs.com/test-catalog/Clinical+and+Interpretive/63020); and publication bias (downgraded if there is evidence of publication bias based on funnel plot asymmetry and/or significant Egger's or Begg's tests (P<0.10) with confirmation by adjustment by Duval and Tweedie trim-and-fill analysis). Criteria for upgrades included a significant dose-response gradient.

[b] For the interpretation of the magnitude, we used the MIDs (see a above) to assess the importance of magnitude of our pooled estimates using the effect size categories according to new GRADE guidance. We then used the MIDs to assess the importance of the magnitude of our point estimates using the effect size categories according GRADE guidance (Santesso et al. 2020, Schunemann et al. 2013, Balshem et al. 2011) as follows: large effect (≥5x MID); moderate effect (≥2x MID); small important effect (≥1x MID); and trivial/unimportant effect (<1 MID).

CI, confidence interval; CRP, C-reactive protein; IL-6, interleukin-6; MD, mean difference; MID, minimally important difference; TNF-α, tumour necrosis factor-alpha; ROB, risk of bias

[1]No downgrade for serious inconsistency. The evidence for substantial heterogeneity was driven by food source (P=0.010 for interaction) for which a double downgrade was already made. The significant interaction of food source also altered the evidence for substantial heterogeneity to non-substantial heterogeneity (Original: I^2=53.7%, P_Q<0.001; residual I^2=44.4%, P_Q=0.01).

[2] Double downgrade for very serious indirectness as there was a significant interaction by food source (P<0.1) indicating that there is biological plausibility of differences in behaviour of foods due to the food matrices.

[3] Double downgrade for very serious indirectness for the effect of total fructose-containing sugars on CRP in addition trials as the effect is driven by one food source (fruit). We double downgraded for very serious indirectness for addition trials of TNF-α as well due to the similar significant effect of fruit.

[4] Double downgrade for very serious indirectness as there was only one food source available for analyses, thus limiting the ability to assess differences in food sources.

[5] Downgrade for serious imprecision as the 95% confidence interval overlaps the MID of clinically important harm for CRP (0.5mg/L).

[6] No downgrade for publication bias, as publication bias could not be assessed due to lack of power for assessing funnel plot asymmetry and small study effects (<10 trial comparisons included in the meta-analysis).

[7] No upgrade for dose-response, as dose-response could not be assessed as <6 trials were available.

[8] Although there was substantial heterogeneity in the analysis of the effect of total fructose containing sugars on TNF- α in substitution trials, we did not downgrade for serious inconsistency, since it was partially explained when the study by Kaliora et al. 2016 or Van Meijl et al. 2011 was removed as part of a priori sensitivity analyses (Original: I^2=53%, P_Q=0.006; after Kaliora et al. 2016 removed: I^2=42%, P_Q=0.038; after Van Meijl et al. 2011 removed: I^2=49%, P_Q=0.014).

[9] Although there was substantial heterogeneity in the analysis of the effect of total fructose containing sugars on TNF- α in addition trials, we did not downgrade for serious inconsistency, since it was partially explained when the study by Leelarungrayub et al. 2016 was removed as part of a priori sensitivity analyses (Original: I^2=87%, P_Q<0.001; after removal: I^2=49%, P_Q=0.017).

[10] Downgrade for serious imprecision as the 95% confidence interval overlaps the MID of clinically important benefit for TNF- α (0.28pg/mL). There was also a gain of significance in sensitivity analyses with the removal of Karlsen et al. 2010 (recalculated MD: -0.61pg/mL; 95% CI: -1.15 to -0.08; P_{MD}=0.027) or Sanchez-Delgado et al. 2021 (ST) (recalculated MD: -0.57pg/mL; 95% CI: -1.09 to -0.05; P_{MD}=0.030) and with the use of a correlation coefficient of 0.25 (recalculated MD: -0.47pg/mL; 95% CI: -0.90 to -0.03; P_{MD}=0.04).

[11] Downgrade for serious imprecision as the 95% confidence interval overlaps the MID of clinically important benefit for IL-6 (0.18pg/mL).

[12] Although a significant publication bias was detected at P=0.004 in Egger’s test, we did not downgrade for publication bias as the imputation of 4 trials from trim-and-fill analyses did not change the significance, magnitude or direction of the overall effect (Original MD: -0.04pg/mL; 95% CI: -0.24 to 0.15, P=0.664; imputed MD: -0.05pg/mL; 95% CI: -0.28 to 0.18, P=0.677).

[13] Downgrade for serious inconsistency, due to substantial unexplained heterogeneity I^2=83%, P_Q <0.001.

[14] Although a significant publication bias was detected at P=0.015 in Egger’s test, we did not downgrade for publication bias as the imputation of 4 trials from trim-and-fill analyses did not change the significance, magnitude or direction of the overall effect (Original MD: -0.15pg/mL; 95% CI: -0.45 to 0.16, P=0.349; imputed MD: -0.06pg/mL; 95% CI: -0.39 to 0.27, P=0.718).

Supplemental Table S8: GRADE certainty of evidence assessment* for the effect of fructose-containing sugars on biomarkers of inflammation by important food source of fructose-containing sugars

		GRADE assessment									
		Downgrades					Upgrades				
Outcome and trial (N)	Design	Risk of bias	Inconsistency	Indirectness	Imprecision	Publication bias	Dose response		Effect (MD [95%CI], P_{MD})	Certainty of Evidence[a]	Interpretation of magnitude of effect[b]
**CRP in substitution trials (mg/L)**											
SSB (10)	Randomized and non-randomized trials	Not serious	Not serious	Not serious	Not serious	None	None	↔	0.07 [-0.16 to 0.30], P=0.543	⊕⊕⊕⊕ High	No effect
Sweetened dairy (3)	Randomized trials	Not serious	Not serious	Serious[1]	Serious[2]	None[3]	None[4]	↔	-0.06 [-0.51 to 0.39], P=0.802	⊕⊕◯◯ Low	No effect
Sweetened dairy alternative (soy) (1)	Randomized trial	Not serious	Not serious	Serious[1]	Serious[2]	None[3]	None[4]	↓	-0.96 [-1.67 to -0.25], P=0.008	⊕⊕◯◯ Low	Small important
100% Fruit juice (2)	Randomized trials	Not serious	Not serious	Serious[1]	Serious[2]	None[3]	None[4]	↓	-1.09 [-2.01 to -0.17], P=0.021	⊕⊕◯◯ Low	Moderate
Fruit (5)	Randomized trials	Not serious	Not serious	Not serious	Serious[2]	None[3]	None[4]	↔	-0.43 [-0.87 to 0.01], P=0.055	⊕⊕⊕◯ Moderate	No effect
Dried fruit (4)	Randomized trials	Not serious	Not serious	Not serious	Serious[2]	None[3]	None[4]	↔	0.21 [-0.14 to 0.55], P=0.240	⊕⊕⊕◯ Moderate	No effect
Mixed fruit forms (1)	Randomized trial	Not serious	Not serious	Serious[1]	Serious[2]	None[3]	None[4]	↔	-0.10 [-1.20 to 1.00], P=0.859	⊕⊕◯◯ Low	No effect
Added nutritive (caloric) sweetener (1)	Randomized trial	Not serious	Not serious	Serious[1]	Not serious	None[3]	None[4]	↔	-0.03 [-0.11 to 0.05], P=0.464	⊕⊕⊕◯ Moderate	No effect
Mixed sources (with SSBs) (6)	Randomized and non-randomized trials	Not serious[5]	Not serious[6]	Not serious	Serious[2]	None[3]	None[4]	↑	0.64 [0.12 to 1.17], P=0.016	⊕⊕⊕◯ Moderate	Small important
Mixed sources (without SSBs) (4)	Randomized trials	Not serious	Not serious	Not serious	Serious[2]	None[3]	None[4]	↔	0.28 [-0.21 to 0.78], P=0.260	⊕⊕⊕◯ Moderate	No effect
**CRP in addition trials (mg/L)**											
SSB (7)	Randomized and non-	Not serious	Not serious	Not serious	Not serious	None[3]	None	↔	0.02 [-0.15 to 0.19], P=0.790	⊕⊕⊕⊕ High	No effect

	randomized trials										
Sweetened dairy alternative (soy) (2)	Randomized trials	Not serious	Not serious	Serious[1]	Serious[2]	None[3]	None[4]	↔	0.20 [-0.78 to 1.18], P=0.689	⊕⊕◯◯ Low	No effect
100% Fruit juice (12)	Randomized and non-randomized trials	Not serious	Not serious	Not serious	Serious[2]	None[3]	None	↔	-0.12 [-0.53 to 0.30], P=0.580	⊕⊕⊕◯ Moderate	No effect
Fruit (9)	Randomized and non-randomized trials	Not serious	Not serious	Not serious	Serious[2]	None[3]	None	↓	-0.50 [-0.75 to -0.25], P<0.001	⊕⊕⊕◯ Moderate	Small important
Dried fruit (3)	Randomized trials	Not serious	Not serious	Serious[1]	Serious[2]	None[3]	None[4]	↔	0.02 [-0.75 to 0.79], P=0.962	⊕⊕◯◯ Low	No effect
Sweetened cereal grains and bars (1)	Non-randomized trial	Serious[7]	Not serious	Serious[1]	Serious[2]	None[3]	None[4]	↔	-0.30 [-0.79 to 0.19], P=0.228	⊕◯◯◯ Very low	No effect
Sweets and desserts (2)	Randomized trials	Not serious	Serious[8]	Serious[1]	Serious[2]	None[3]	None[4]	↔	-0.67 [-1.85 to 0.50], P=0.260	⊕◯◯◯ Very low	No effect
Added nutritive (caloric) sweetener (1)	Randomized trial	Not serious	Not serious	Serious[1]	Serious[2]	None[3]	None[4]	↔	0.56 [-4.19 to 5.31], P=0.817	⊕⊕◯◯ Low	No effect
**CRP in subtraction trials (mg/L)**											
SSB (4)	Randomized trials	Not serious	Not serious	Serious[1]	Serious[2]	None[3]	None[4]	↔	0.14 [-0.29 to 0.56], P=0.522	⊕⊕◯◯ Low	No effect
**CRP in *ad libitum* trials (mg/L)**											
Mixed sources (with SSBs) (3)	Randomized trials	Not serious	Not serious	Serious[1]	Not serious	None[3]	None[4]	↔	-0.09 [-0.44 to 0.25], P=0.604	⊕⊕⊕◯ Moderate	No effect
**TNF-α in addition trials (pg/mL)**											
SSB (3)	Randomized trials	Not serious	Not serious[9]	Serious[1]	Serious[2]	None[3]	None[4]	↔	0.79 [-1.23 to 2.80], P=0.444	⊕⊕◯◯ Low	No effect
Sweetened dairy (2)	Randomized trials	Not serious	Not serious	Serious[1]	Serious[2]	None[3]	None[4]	↔	-0.10 [-0.30 to 0.10], P=0.336	⊕⊕◯◯ Low	No effect

100% Fruit juice (3)	Randomized and non-randomized trials	Not serious	Serious[10]	Not serious	Serious[2]	None[3]	None[4]	↔	-0.65 [-2.57 to 1.27], P=0.507	⊕⊕○○ Low	No effect
Fruit (3)	Randomized and non-randomized trials	Not serious	Not serious	Not serious	Serious[2]	None[3]	None[4]	↓	-0.89 [-1.58 to -0.20], P=0.012	⊕⊕⊕○ Moderate	Small important
Sweets and desserts (2)	Randomized trials	Not serious	Serious[11]	Serious[1]	Serious[2]	None[3]	None[4]	↔	-4.66 [-16.21 to 6.90], P=0.429	⊕○○○ Very low	No effect
Added nutritive (caloric) sweetener (3)	Randomized trials	Not serious	Not serious	Serious[1]	Serious[2]	None[3]	None[4]	↔	-0.91 [-2.18 to 0.36], P=0.162	⊕⊕○○ Low	No effect
**IL-6 in addition trials (pg/mL)**											
SSB (5)	Randomized trials	Not serious	Not serious	Not serious	Serious[2]	None[3]	None[4]	↔	0.06 [-0.34 to 0.46], P=0.776	⊕⊕⊕○ Moderate	No effect
Sweetened dairy (2)	Randomized trials	Not serious	Not serious	Serious[1]	Serious[2]	None[3]	None[4]	↔	-0.52 [-1.12 to 0.08], P=0.087	⊕⊕○○ Low	No effect
100% Fruit juice (4)	Randomized and non-randomized trials	Not serious	Not serious[12]	Not serious	Serious[2]	None[3]	None[4]	↔	-3.01 [-6.91 to 0.88], P=0.130	⊕⊕⊕○ Moderate	No effect
Fruit (3)	Randomized and non-randomized trials	Not serious	Serious[13]	Not serious	Serious[2]	None[3]	None[4]	↔	-0.19 [-0.59 to 0.21], P=0.352	⊕⊕○○ Low	No effect
Sweets and desserts (dark chocolate) (1)	Randomized trial	Not serious	Not serious	Serious[1]	Not serious	None[3]	None[4]	↓	-8.79 [-14.26 to -3.32], P=0.002	⊕⊕⊕○ Moderate	Large
Added nutritive (caloric) sweetener (1)	Randomized trial	Not serious	Not serious	Serious[1]	Serious[2]	None[3]	None[4]	↔	0.40 [-0.22 to 1.02], P=0.207	⊕⊕○○ Low	No effect

[a] Since all included trials were randomized or non-randomized controlled trials, the certainty of the evidence was graded as high for all outcomes by default and then downgraded or upgraded based on pre-specified criteria. Criteria for downgrades included risk of bias (ROB) (downgraded if the majority of trials were considered to be at high ROB); inconsistency (downgraded if there was substantial unexplained heterogeneity [$I^2 \geq 50\%$, $P_Q < 0.10$]; indirectness (downgraded if there were factors absent or present relating to the participants, interventions, or outcomes that limited the generalizability of the results); imprecision (downgraded if the 95% confidence interval crossed the minimally important difference [MID] for harm or benefit set at 0.5 mg/L for CRP (Reynolds Risk Score. Available at: http://www.reynoldsriskscore.org/Default.aspx [Accessed March 14, 2018]; Ridker, P.M. et al., 2008. Circulation,

118(22), pp.2243–51; Ridker, P.M. et al., 2007. JAMA 297(6), pp.611–619.), 0.28 pg/mL for TNF-α (Mayoclinic. Tumour Necrosis Factor (TNF), Plasma. Available from: https://www.mayocliniclabs.com/test-catalog/Clinical+and+Interpretive/63022), 0.18 pg/mL for IL-6 (Mayoclinic. Interleukin 6, Plasma. Available from: https://www.mayocliniclabs.com/test-catalog/Clinical+and+Interpretive/63020); and publication bias (downgraded if there is evidence of publication bias based on funnel plot asymmetry and/or significant Egger's or Begg's tests (P<0.10) with confirmation by adjustment by Duval and Tweedie trim-and-fill analysis). Criteria for upgrades included a significant dose-response gradient.

[b] For the interpretation of the magnitude, we used the MIDs (see a above) to assess the importance of magnitude of our pooled estimates using the effect size categories according to new GRADE guidance. We then used the MIDs to assess the importance of the magnitude of our point estimates using the effect size categories according GRADE guidance (Santesso et al. 2020, Schunemann et al. 2013, Balshem et al. 2011) as follows: large effect (≥5x MID); moderate effect (≥2x MID); small important effect (≥1x MID); and trivial/unimportant effect (< 1 MID).

CI, confidence interval; CRP, C-reactive protein; IL-6, interleukin-6; MD, mean difference; MID, minimally important difference; TNF-α, tumour necrosis factor-alpha; ROB, risk of bias

1 Downgrade for serious indirectness as all trial comparisons come from one or two studies, which leads to lack of reproducibility and poor applicability of the results to the general adult population.

[2] Downgrade for serious imprecision as the 95% confidence interval overlaps the MID of clinically important harm and/or benefit for CRP (0.5mg/L), TNF-α (0.28pg/mL) or IL-6 (0.18pg/mL). Further, in sensitivity analyses, the removal of the following individual trials resulted in a loss of significance: Jalilvand et al. 2020 or Khodami et al. 2022 for mixed sources with SSBs on CRP in substitution trials; Ribeiro et al. 2017 for 100% fruit juice on CRP in substitution trials; gain of significance in sensitivity analyses with the removal of Lehtonen et al. 2011 for fruit on CRP in substitution trials; Alavinejad et al. 2015 for sweets and desserts on CRP in addition trials; Ellis et al. 2011 (SB) for sweetened dairy on IL-6 in addition trials; Simao et al. 2013 for 100% fruit juice on IL-6 in addition trials; and with the use of a correlation coefficient of 0.75 for crossover trials for fruit on CRP in substitution trials (recalculated MD: -043mg/L; 95% CI: -0.85 to -0.01; P_{MD}=0.045).

[3] No downgrade for publication bias, as publication bias could not be assessed due to lack of power for assessing funnel plot asymmetry and small study effects (<10 trial comparisons included in the meta-analysis).

[4] No upgrade for dose-response, as dose-response could not be assessed as <6 trials were available with dose data.

[5] No downgrade for serious risk of bias since although half of trial comparisons (3 out of 6) were at high risk of bias for sequence generation and allocation concealment, there was no effect in sensitivity analyses between the effect of each set of 3 trials.

[6] Although there was substantial heterogeneity in the analysis of the effect of mixed sources with SSBs on CRP in substitution trials, we did not downgrade for serious inconsistency, since it was partially explained when the study by Souto et al. 2013 was removed as part of a priori sensitivity analyses (Original: I^2=83%, P_Q<0.001; after removal: I^2=0%, P_Q=0.865).

[7] Downgrade for serious risk of bias since the one trial was non-randomized study and thus sequence generation and allocation concealment were high risk of bias.
[8] Downgrade for serious inconsistency, due to substantial unexplained heterogeneity I^2=87%, $P_{Q=}$0.006.
[9] Although there was substantial heterogeneity in the analysis of the effect of SSB on TNF-α in addition trials, we did not downgrade for serious inconsistency, since it was partially explained when the study by Sanchez-Delgado et al. 2021 (ST) was removed as part of a priori sensitivity analyses (Original: I_2=59%, P_Q=0.089; after study removed: I_2=0%, P_Q=0.979).
[10] Downgrade for serious inconsistency, due to substantial unexplained heterogeneity I^2=96%, $P_Q<0.001$.
[11] Downgrade for serious inconsistency, due to substantial unexplained heterogeneity I^2=77%, $P_{Q=}$0.04.
[12] Although there was substantial heterogeneity in the analysis of the effect of 100% fruit juice on IL-6 in addition trials, we did not downgrade for serious inconsistency, since it was partially explained when the study by Simao et al. 2013 was removed as part of a priori sensitivity analyses (Original: I^2=74%, P_Q=0.009; after removal: I^2=46%, P_Q=0.160).
[13] Downgrade for serious inconsistency, due to substantial unexplained heterogeneity I^2=86%, $P_Q<0.001$.

Supplemental Figures

Supplemental Figure S1: Risk of bias proportion graph for the effect of important food sources of fructose-containing sugars on CRP (mg/L) in substitution trials

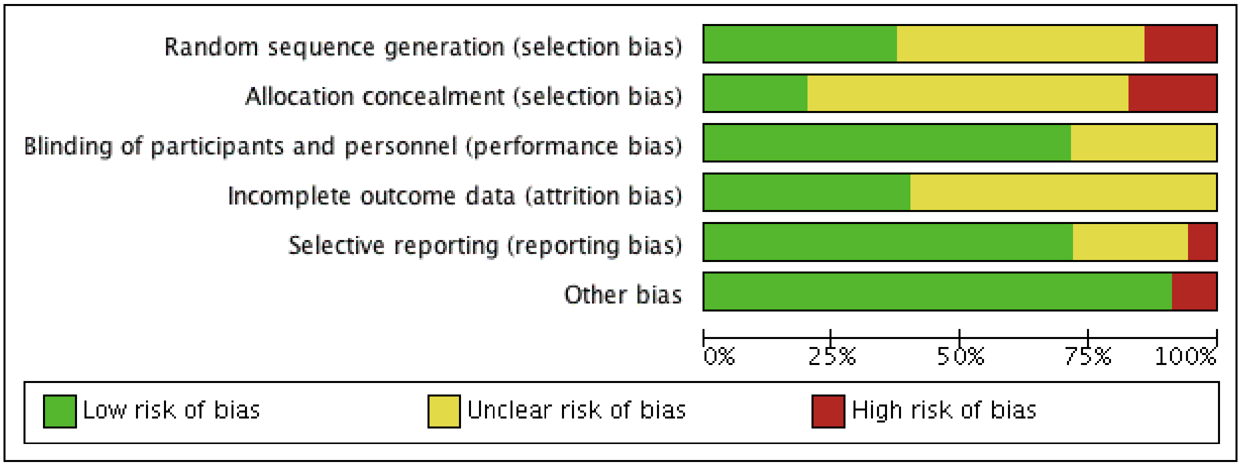

Colored bars represent the proportion of trials assessed as low (green), unclear (yellow) or high (red) risk of bias for the six domains of bias above according to criteria set by the Cochrane Risk of Bias tool in the 35 included controlled trial comparisons.
High other risk of bias (carry-over effect) was given to crossover trials which had no washout between interventions. Trials which did not have this characteristic were rated as Low.

Supplemental Figure S2: Risk of bias proportion graph for the effect of important food sources of fructose-containing sugars on CRP (mg/L) in addition trials

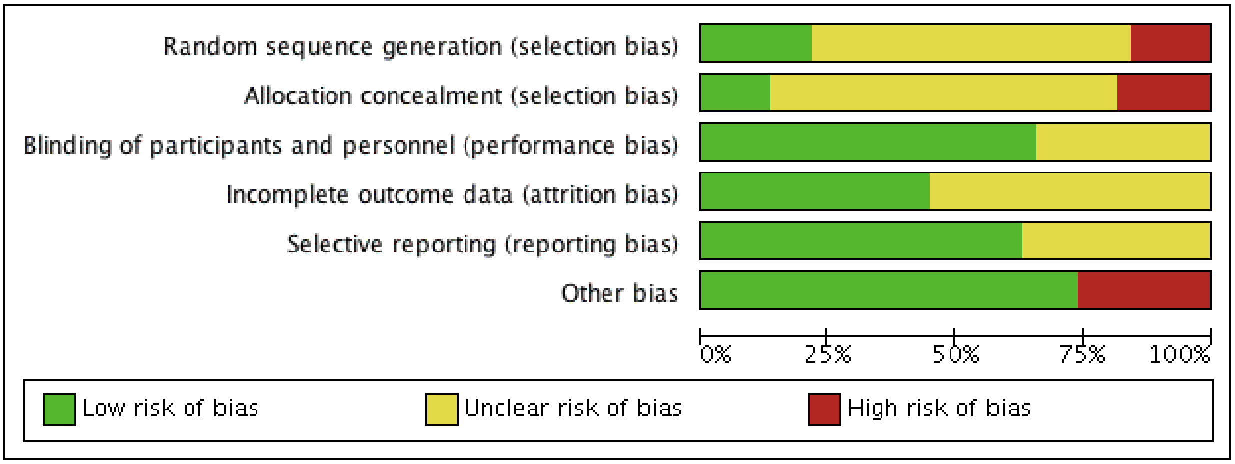

Colored bars represent the proportion of trials assessed as low (green), unclear (yellow) or high (red) risk of bias for the six domains of bias above according to criteria set by the Cochrane Risk of Bias tool in the 38 included controlled trial comparisons.
High other risk of bias (carry-over effect) was given to crossover trials which had no washout between interventions. Trials which did not have this characteristic were rated as Low.

Supplemental Figure S3: Risk of bias proportion graph for the effect of important food sources of fructose-containing sugars on CRP (mg/L) in subtraction trials

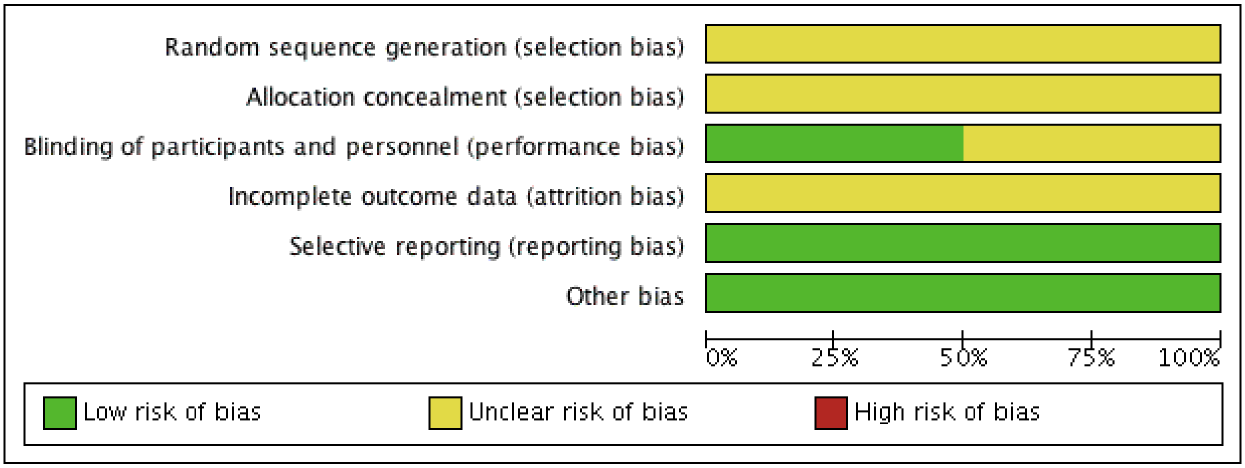

Colored bars represent the proportion of trials assessed as low (green), unclear (yellow) or high (red) risk of bias for the six domains of bias above according to criteria set by the Cochrane Risk of Bias tool in the 4 included controlled trial comparisons.
High other risk of bias (carry-over effect) was given to crossover trials which had no washout between interventions. Trials which did not have this characteristic were rated as Low.

Supplemental Figure S4: Risk of bias proportion graph for the effect of important food sources of fructose-containing sugars on CRP (mg/L) in ad libitum trials

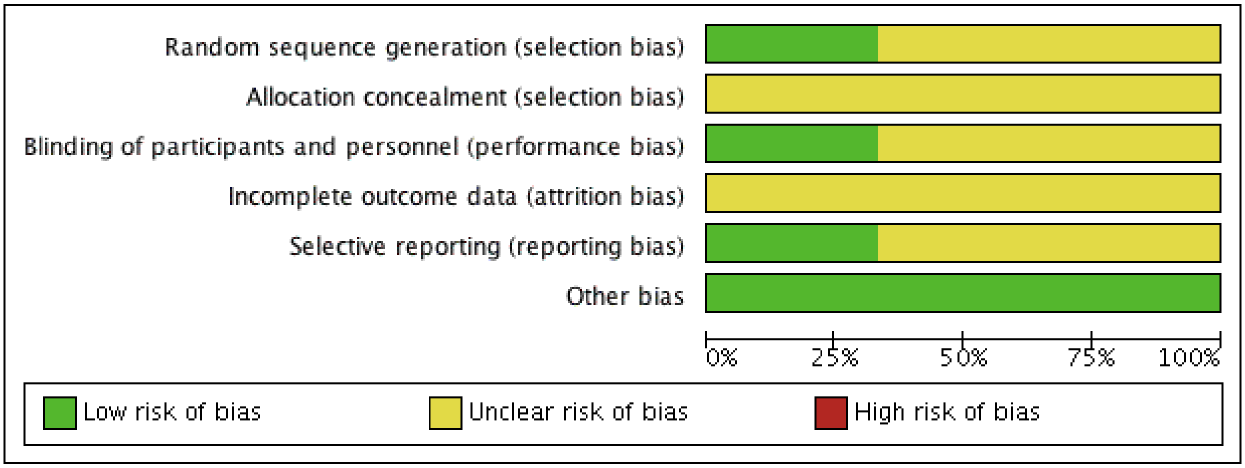

Colored bars represent the proportion of trials assessed as low (green), unclear (yellow) or high (red) risk of bias for the six domains of bias above according to criteria set by the Cochrane Risk of Bias tool in the 3 included controlled trial comparisons.
High other risk of bias (carry-over effect) was given to crossover trials which had no washout between interventions. Trials which did not have this characteristic were rated as Low.

Supplemental Figure S5: Risk of bias proportion graph for the effect of important food sources of fructose-containing sugars on TNF- α (pg/mL) trials in substitution trials

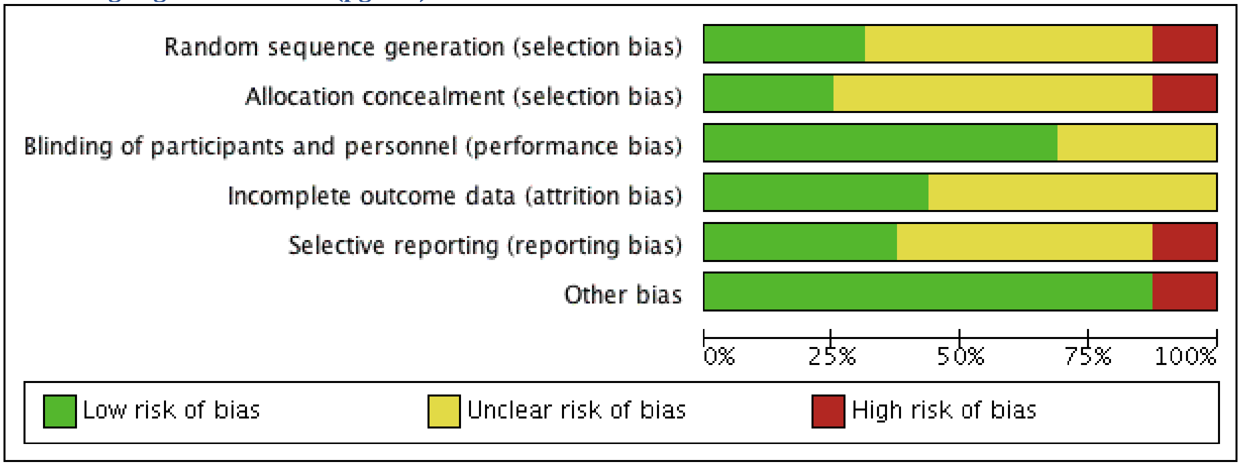

Colored bars represent the proportion of trials assessed as low (green), unclear (yellow) or high (red) risk of bias for the six domains of bias above according to criteria set by the Cochrane Risk of Bias tool in the 16 included controlled trial comparisons.
High other risk of bias (carry-over effect) was given to crossover trials which had no washout between interventions. Trials which did not have this characteristic were rated as Low.

Supplemental Figure S6: Risk of bias proportion graph for the effect of important food sources of fructose-containing sugars on TNF-α (pg/mL) in addition trials

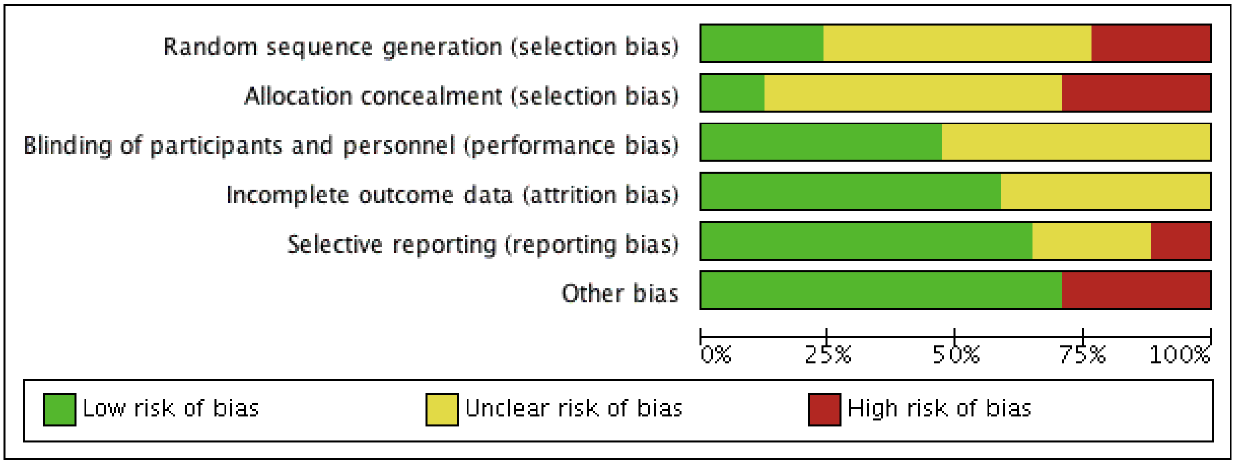

Colored bars represent the proportion of trials assessed as low (green), unclear (yellow) or high (red) risk of bias for the six domains of bias above according to criteria set by the Cochrane Risk of Bias tool in the 17 included controlled trial comparisons.
High other risk of bias (carry-over effect) was given to crossover trials which had no washout between interventions. Trials which did not have this characteristic were rated as Low.

Supplemental Figure S7: Risk of bias proportion graph for the effect of important food sources of fructose-containing sugars on IL-6 (pg/mL) in substitution trials

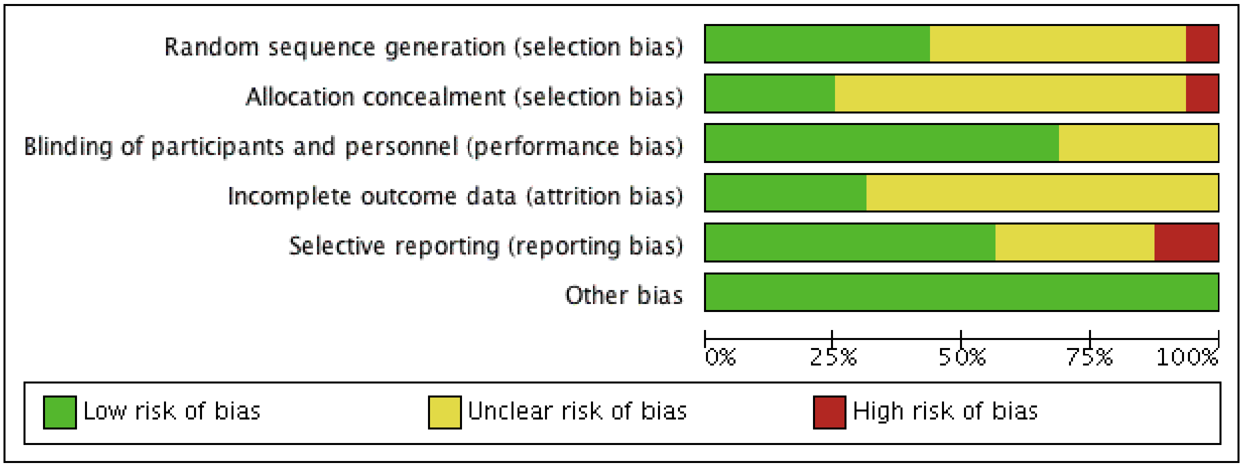

Colored bars represent the proportion of trials assessed as low (green), unclear (yellow) or high (red) risk of bias for the six domains of bias above according to criteria set by the Cochrane Risk of Bias tool in the 16 included controlled trial comparisons.
High other risk of bias (carry-over effect) was given to crossover trials which had no washout between interventions. Trials which did not have this characteristic were rated as Low.

Supplemental Figure S8: Risk of bias proportion graph for the effect of important food sources of fructose-containing sugars on IL-6 (pg/mL) in addition trials

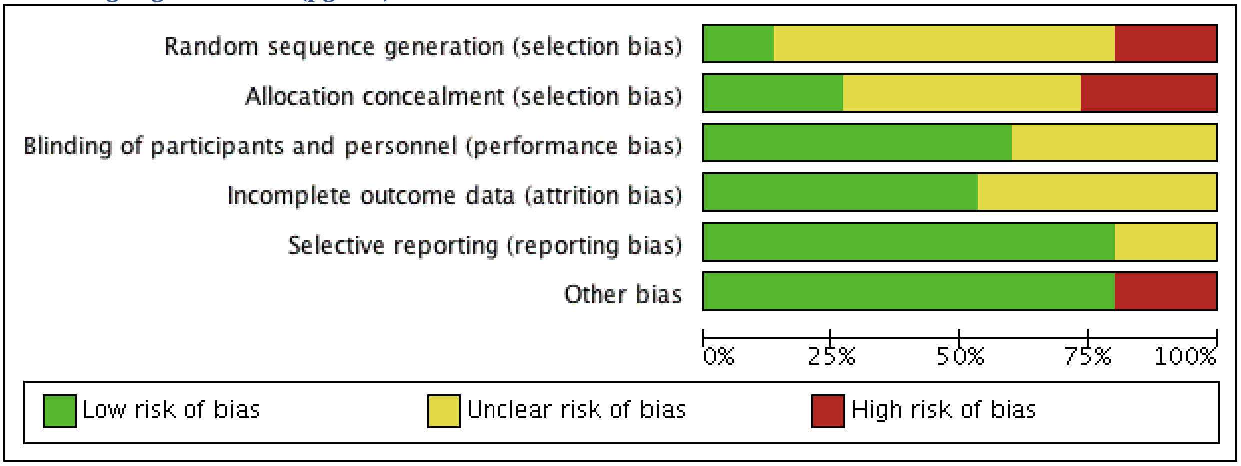

Colored bars represent the proportion of trials assessed as low (green), unclear (yellow) or high (red) risk of bias for the six domains of bias above according to criteria set by the Cochrane Risk of Bias tool in the 15 included controlled trial comparisons.
High other risk of bias (carry-over effect) was given to crossover trials which had no washout between interventions. Trials which did not have this characteristic were rated as Low.

Supplemental Figure S9: Forest plot of controlled trials of the effect of important food sources of fructose-containing sugars on CRP (mg/L) in substitution trials

CRP (mg/L)
Substitution trials

Trial or subgroup	N Control	N Intervention	Mean difference with 95% CI	Weight (%)	A	B	C	D	E	F
**1. SSB**										
Aeberli et al. 2011 (HF vs HG)	29	29	0.04 [-0.47 to 0.55]	4.48	U	U	L	L	L	L
Aeberli et al. 2011 (HS vs HG)	29	29	0.03 [-0.47 to 0.53]	4.54	U	U	L	L	L	L
Aeberli et al. 2011 (MF vs MG)	29	29	-0.02 [-0.49 to 0.45]	4.79	U	U	L	L	L	L
Chiu et al. 2020	30	30	0.10 [-0.53 to 0.74]	3.44	L	L	L	L	L	L
Cox et al. 2011	15	16	0.70 [-0.69 to 2.09]	1.04	H	H	U	U	U	L
Jin et al. 2014	12	9	1.50 [0.35 to 2.65]	1.45	U	U	L	L	L	L
Johnston et al. 2013 (T1 Neutral E balance)	17	15	-0.38 [-1.31 to 0.55]	2.03	L	L	L	L	L	L
Johnston et al. 2013 (T2 Positive E balance)	17	15	-1.46 [-4.60 to 1.68]	0.22	L	L	L	L	L	L
Kuzma et al. 2016 (Frc)	24	24	0.08 [-0.95 to 1.11]	1.73	L	U	L	U	L	L
Kuzma et al. 2016 (HFCS)	24	24	-0.05 [-1.11 to 1.01]	1.65	L	U	L	U	L	L
Heterogeneity: $\tau^2 = 0.00$, $I^2 = 0.00\%$, $H^2 = 1.00$			0.07 [-0.16 to 0.30]							
Test of $\theta_i = \theta_j$: Q(9) = 8.81, p = 0.45										
**2. Sweetened Dairy**										
Angelopoulos et al. 2016 (FRC)	77	65	0.25 [-0.52 to 1.02]	2.68	U	U	L	U	L	L
Angelopoulos et al. 2016 (HFCS)	77	65	-0.23 [-1.04 to 0.58]	2.50	U	U	L	U	L	L
Angelopoulos et al. 2016 (SUC)	77	61	-0.20 [-0.95 to 0.55]	2.77	U	U	L	U	L	L
Heterogeneity: $\tau^2 = 0.00$, $I^2 = 0.00\%$, $H^2 = 1.00$			-0.06 [-0.51 to 0.39]							
Test of $\theta_i = \theta_j$: Q(2) = 0.92, p = 0.63										
**3. Sweetened dairy alternatives (soy)**										
Eslami et al. 2019	32	32	-0.96 [-1.67 to -0.25]	3.02	L	H	L	U	L	L
Heterogeneity: $\tau^2 = 0.00$, $I^2 = .\%$, $H^2 = .$			-0.96 [-1.67 to -0.25]							
Test of $\theta_i = \theta_j$: Q(0) = 0.00, p = .										
**4. 100% Fruit juice**										
Ponce et al. 2019	36	36	-1.80 [-4.54 to 0.94]	0.29	L	L	U	U	L	L
Ribeiro et al. 2017	39	39	-1.00 [-1.98 to -0.02]	1.87	L	U	U	L	L	L
Heterogeneity: $\tau^2 = 0.00$, $I^2 = 0.00\%$, $H^2 = 1.00$			-1.09 [-2.01 to -0.17]							
Test of $\theta_i = \theta_j$: Q(1) = 0.29, p = 0.59										
**5. Fruit**										
Fridell et al. 2018	15	15	-0.31 [-1.04 to 0.42]	2.91	L	L	L	L	L	L
Kolehmainen et al. 2012	11	13	-0.77 [-1.51 to -0.03]	2.83	U	U	U	U	U	L
Lehtonen et al. 2011 (BB)	80	80	0.20 [-0.48 to 0.88]	3.18	U	U	U	L	H	L
Moazen et al. 2013	17	19	-0.98 [-1.77 to -0.19]	2.61	U	U	L	U	L	L
Navaei et al. 2019	40	40	-0.39 [-1.66 to 0.88]	1.21	L	U	L	U	L	L
Heterogeneity: $\tau^2 = 0.09$, $I^2 = 34.32\%$, $H^2 = 1.52$			-0.43 [-0.87 to 0.01]							
Test of $\theta_i = \theta_j$: Q(4) = 6.09, p = 0.19										
**6. Dried fruit**										
Kaliora et al. 2016	21	23	0.25 [-1.20 to 1.70]	0.96	L	U	L	U	U	L
Kanellos et al. 2014	22	26	0.70 [-0.18 to 1.58]	2.20	L	U	L	U	L	L
Kanellos et al. 2017	13	20	0.30 [-1.55 to 2.15]	0.62	L	U	L	U	L	L
Lehtonen et al. 2011 (SB)	80	80	0.10 [-0.29 to 0.49]	5.60	U	U	U	L	H	L
Heterogeneity: $\tau^2 = 0.00$, $I^2 = 0.00\%$, $H^2 = 1.00$			0.21 [-0.14 to 0.55]							
Test of $\theta_i = \theta_j$: Q(3) = 1.50, p = 0.68										
**7. Mixed fruit forms**										
Lehtonen et al. 2010	22	28	-0.10 [-1.20 to 1.00]	1.55	U	U	U	U	U	L
Heterogeneity: $\tau^2 = 0.00$, $I^2 = .\%$, $H^2 = .$			-0.10 [-1.20 to 1.00]							
Test of $\theta_i = \theta_j$: Q(0) = 0.00, p = .										
**8. Added nutritive (caloric) sweeteners**										
Sadeghi et al. 2020	42	42	-0.03 [-0.11 to 0.05]	8.86	U	L	L	L	L	L
Heterogeneity: $\tau^2 = 0.00$, $I^2 = .\%$, $H^2 = .$			-0.03 [-0.11 to 0.05]							
Test of $\theta_i = \theta_j$: Q(0) = 0.00, p = .										
**9. Mixed sources (with SSBs)**										
Brymora et al. 2012 (T1)	28	28	1.00 [0.00 to 2.00]	1.81	H	H	H	U	U	H
Brymora et al. 2012 (T2)	28	28	0.90 [-0.31 to 2.11]	1.32	H	H	H	U	U	H
Jalilvand et al. 2020	20	20	0.84 [0.46 to 1.22]	5.77	L	L	L	U	L	L
Khodami et al. 2022	22	21	1.02 [0.46 to 1.58]	3.99	L	L	U	U	L	L
Niholm et al. 2021	80	25	0.30 [-0.87 to 1.47]	1.40	U	U	L	U	L	L
Souto et al 2013	18	15	0.02 [-0.12 to 0.16]	8.43	H	H	U	U	U	L
Heterogeneity: $\tau^2 = 0.29$, $I^2 = 82.91\%$, $H^2 = 5.85$			0.64 [0.12 to 1.17]							
Test of $\theta_i = \theta_j$: Q(5) = 29.26, p = 0.00										
**10. Mixed sources (no SSBs)**										
Goss et al. 2020	19	15	-0.50 [-3.31 to 2.31]	0.28	U	L	L	U	L	L
Maki et al. 2020	30	30	0.04 [-0.81 to 0.88]	2.35	U	U	L	U	L	L
Palacios et al. 2020	33	33	-0.80 [-6.68 to 5.08]	0.06	U	U	U	U	L	L
Van Meijl et al. 2011	35	35	0.47 [-0.15 to 1.09]	3.56	U	U	L	U	U	L
Heterogeneity: $\tau^2 = 0.00$, $I^2 = 0.00\%$, $H^2 = 1.00$			0.28 [-0.21 to 0.78]							
Test of $\theta_i = \theta_j$: Q(3) = 1.11, p = 0.78										
**Overall**			0.07 [-0.08 to 0.22]							
Heterogeneity: $\tau^2 = 0.06$, $I^2 = 53.70\%$, $H^2 = 2.16$										
Test of $\theta_i = \theta_j$: Q(36) = 77.75, p = 0.00										
Test of group differences: p = 0.01, $I^2 = 44.40\%$, $p_{het} = 0.01$										
Test of $\theta = 0$: z = 0.963, p = 0.336										

Risk of bias

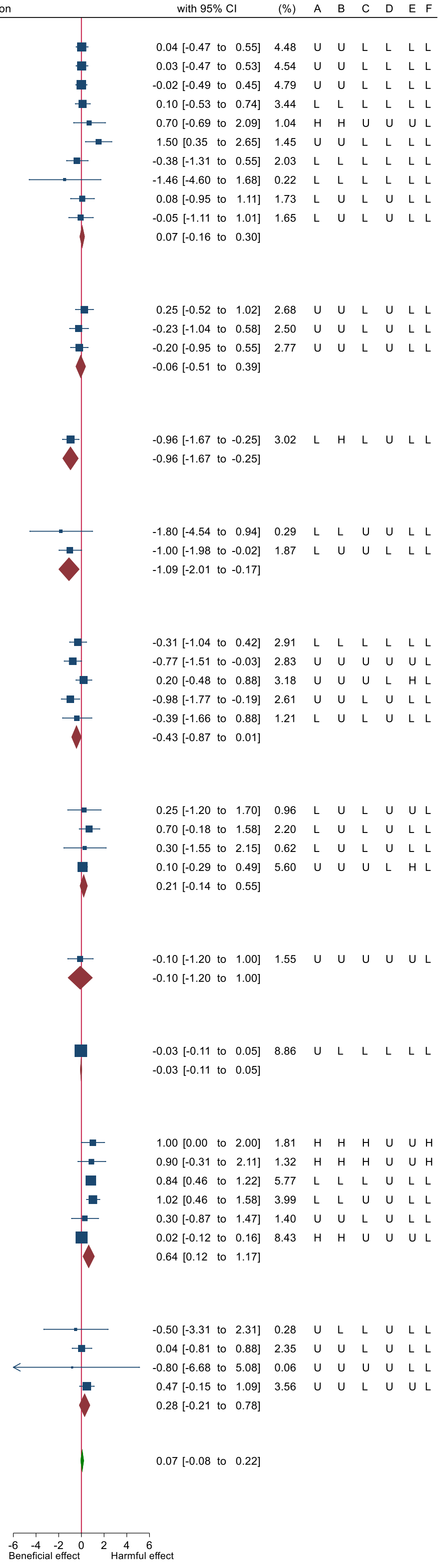

CI, confidence interval; CRP= C reactive protein; BB= Bilberries; FRC= fructose; HG=high glucose; HS=high sucrose; HF=high fructose; MG=medium glucose; MF= medium fructose; HFCS=high-fructose corn syrup; SB= sea buckthorn berries; SSB=sugar-sweetened beverage; SUC= Sucrose; T1= Test 1;T2= Test 2

Pooled effect estimates for each subgroup and overall effect are represented by the diamonds. Data are expressed as weighted mean differences with 95% confidence intervals using the generic inverse-variance method and random effects DerSimonian-Laird model. Paired analyses were applied to all crossover trials. Inter-study heterogeneity was assessed using the Cochrane Q statistic and quantified using the I^2 statistic, with significance set at $p<0.100$ and $I^2 \geq 50\%$ considered to be evidence of substantial heterogeneity.

Risk of Bias Legend: (H) High Risk; (L) Low Risk; (U) Unclear. The letters represent the following risk of bias domains: A, random sequence generation (selection bias); B, allocation concealment (selection bias); C, blinding of participants and personnel and outcome assessors (performance bias); D, incomplete outcome data (attrition bias); E, selective reporting (reporting bias); and F, other bias. High other risk of bias (carry-over effect) was given to crossover trials which had no washout between interventions. Trials which did not have this characteristic were rated as Low.

Pooled effect summary calculated with the χ^2 test. Test for group differences calculated with meta-regression, which uses the Wald test.

Supplemental Figure S10: Forest plot of controlled trials of the effect of important food sources of fructose-containing sugars on CRP (mg/L) in addition trials

CRP (mg/L)

Addition trials

Trial or subgroup	N Control	N Intervention	Mean difference with 95% CI	Weight (%)	Risk of bias A	B	C	D	E	F
**1. SSB**										
Aeberli et al. 2011 (HF)	29	29	0.10 [-0.43 to 0.64]	4.34	U	U	L	L	L	L
Aeberli et al. 2011 (HS)	29	29	0.09 [-0.44 to 0.62]	4.39	U	U	L	L	L	L
Aeberli et al. 2011 (MF)	29	29	0.05 [-0.46 to 0.55]	4.60	U	U	L	L	L	L
Johnston et al. 2013	17	15	-1.06 [-1.90 to -0.22]	2.44	H	H	L	L	L	H
Njike et al. 2011	37	37	0.39 [-0.36 to 1.13]	2.88	L	U	L	U	L	L
Vaz et al. 2011 (Fort)	97	95	0.11 [-0.07 to 0.28]	8.36	L	L	L	U	L	L
Vaz et al. 2011 (Unfort)	97	95	-0.04 [-0.23 to 0.16]	8.17	L	L	L	U	L	L
Heterogeneity: $\tau^2 = 0.01$, $I^2 = 30.88\%$, $H^2 = 1.45$			0.02 [-0.15 to 0.19]							
Test of $\theta_i = \theta_j$: Q(6) = 8.68, p = 0.19										
**2. Sweetened dairy alternatives (soy)**										
Ellis et al. 2011 (PL)	12	12	0.70 [-0.26 to 1.66]	1.98	U	U	U	L	L	H
Ellis et al. 2011 (SB)	12	12	-0.30 [-1.26 to 0.66]	1.98	U	U	U	L	L	H
Heterogeneity: $\tau^2 = 0.26$, $I^2 = 51.98\%$, $H^2 = 2.08$			0.20 [-0.78 to 1.18]							
Test of $\theta_i = \theta_j$: Q(1) = 2.08, p = 0.15										
**3. 100% Fruit juice**										
Aghababaee et al. 2015	36	36	-0.10 [-0.39 to 0.20]	6.95	L	U	U	L	U	L
Asgary et al. 2014	10	11	-0.32 [-5.87 to 5.23]	0.07	H	H	L	L	L	L
Castilla et al. 2008	8	8	1.70 [-9.51 to 12.91]	0.02	U	U	U	L	U	L
Castilla et al. 2008 Vit E	8	8	2.00 [-12.11 to 16.11]	0.01	U	U	U	L	U	L
Fatel et al. 2021	21	20	-5.32 [-7.93 to -2.71]	0.33	U	U	L	U	L	L
Karlsen et al. 2010	31	31	-0.45 [-0.84 to -0.06]	5.82	U	L	U	L	L	L
Ravn-Haren et al. 2013 (Cld vs Ctrl)	23	23	0.04 [-0.94 to 1.02]	1.91	U	U	L	U	U	H
Ravn-Haren et al. 2013 (Cld vs Pom)	23	23	0.23 [-0.42 to 0.88]	3.46	U	U	L	U	U	H
Ravn-Haren et al. 2013 (Clr vs Ctrl)	23	23	0.18 [-0.84 to 1.20]	1.79	U	U	L	U	U	H
Ravn-Haren et al. 2013 (Clr vs Pom)	23	23	0.37 [-0.42 to 1.16]	2.66	U	U	L	U	U	H
Simao et al. 2013	36	20	1.24 [-3.06 to 5.53]	0.12	H	H	L	U	U	L
Thimoteo et al. 2019	18	20	0.27 [-4.05 to 4.58]	0.12	U	U	L	U	U	L
Heterogeneity: $\tau^2 = 0.17$, $I^2 = 49.29\%$, $H^2 = 1.97$			-0.12 [-0.53 to 0.30]							
Test of $\theta_i = \theta_j$: Q(11) = 21.69, p = 0.03										
**4. Fruit**										
Basu et al. 2010	23	25	-0.20 [-3.30 to 2.90]	0.23	U	U	L	U	L	L
Blum et al. 2007	53	50	-0.20 [-2.30 to 1.90]	0.50	U	U	U	L	U	L
Dow et al. 2013	32	37	-0.50 [-1.07 to 0.07]	4.04	U	U	U	L	L	L
Franck et al. 2020	24	23	-0.94 [-2.08 to 0.20]	1.50	U	U	U	U	L	L
Kelley et al. 2013	11	11	-0.71 [-1.25 to -0.17]	4.27	H	H	L	U	U	L
Liddle et al. 2021	22	22	-0.51 [-0.93 to -0.09]	5.49	L	L	U	L	L	L
Ravn-Haren et al. 2013 (Clr)	23	23	-0.32 [-1.30 to 0.66]	1.90	U	U	L	U	U	H
Ravn-Haren et al. 2013 (Pom)	23	23	-0.13 [-0.81 to 0.55]	3.28	U	U	L	U	U	H
Schell et al. 2019	22	22	-0.50 [-4.26 to 3.26]	0.16	U	U	L	L	L	L
Heterogeneity: $\tau^2 = 0.00$, $I^2 = 0.00\%$, $H^2 = 1.00$			-0.50 [-0.75 to -0.25]							
Test of $\theta_i = \theta_j$: Q(8) = 2.55, p = 0.96										
**5. Dried fruit**										
Hooshmand et al. 2016 (HD)	16	13	0.10 [-0.97 to 1.17]	1.66	U	U	U	L	L	L
Hooshmand et al. 2016 (LD)	16	13	0.02 [-1.10 to 1.14]	1.53	U	U	U	L	L	L
Irannejad et al. 2020	36	36	-3.90 [-11.27 to 3.47]	0.04	L	U	L	U	L	L
Heterogeneity: $\tau^2 = 0.00$, $I^2 = 0.00\%$, $H^2 = 1.00$			0.02 [-0.75 to 0.79]							
Test of $\theta_i = \theta_j$: Q(2) = 1.11, p = 0.57										
**6. Sweetened cereal grains and bars**										
Mietus-Snyder et al. 2012	25	25	-0.30 [-0.79 to 0.19]	4.77	H	H	U	U	U	H
Heterogeneity: $\tau^2 = 0.00$, $I^2 = .\%$, $H^2 = .$			-0.30 [-0.79 to 0.19]							
Test of $\theta_i = \theta_j$: Q(0) = 0.00, p = .										
**7. Sweets and desserts**										
Alavinejad et al. 2015	21	21	-0.11 [-0.56 to 0.34]	5.14	U	L	L	U	L	L
Jafariard e al. 2018	23	21	-1.31 [-2.04 to -0.58]	2.99	U	U	L	U	L	L
Heterogeneity: $\tau^2 = 0.63$, $I^2 = 86.82\%$, $H^2 = 7.59$			-0.67 [-1.85 to 0.50]							
Test of $\theta_i = \theta_j$: Q(1) = 7.59, p = 0.01										
**8. Added nutritive (caloric) sweeteners**										
Ghazali et al. 2017	32	32	0.56 [-4.19 to 5.31]	0.10	L	H	L	U	L	L
Heterogeneity: $\tau^2 = 0.00$, $I^2 = .\%$, $H^2 = .$			0.56 [-4.19 to 5.31]							
Test of $\theta_i = \theta_j$: Q(0) = -0.00, p = .										
**Overall**			-0.18 [-0.33 to -0.03]							
Heterogeneity: $\tau^2 = 0.06$, $I^2 = 43.67\%$, $H^2 = 1.78$										
Test of $\theta_i = \theta_j$: Q(36) = 63.90, p = 0.00										
Test of group differences: p = 0.18, $I^2 = 33.96\%$, $p_{het} = 0.04$										
Test of $\theta = 0$: z = -2.324, p = 0.020										

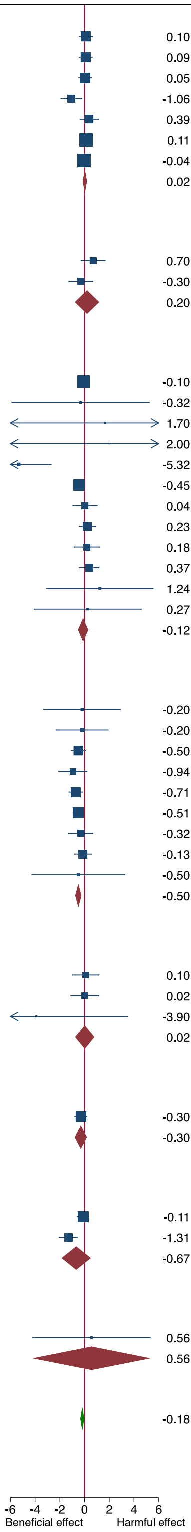

CI, confidence interval; CRP= C reactive protein; Cld=Cloudy; Ctrl=Control; Clr=Clear; Pom=Pomace; Frc= fructose; Fort= fortified; HS=high sucrose; HF=high fructose; MF= medium fructose; HD=higher dose; LD=lower dose; HFCS=high-fructose corn syrup; PB=placebo; SB= strawberries; SSB=sugar-sweetened beverage; T1= Test 1; T2= Test 2: Unfort= unfortified; Vit E= vitamin E.

Pooled effect estimates for each subgroup and overall effect are represented by the diamonds. Data are expressed as weighted mean differences with 95% confidence intervals using the generic inverse-variance method and random effects DerSimonian-Laird model. Paired analyses were applied to all crossover trials. Inter-study heterogeneity was assessed using the Cochrane Q statistic and quantified using the I^2 statistic, with significance set at $p<0.100$ and $I^2 \geq 50\%$ considered to be evidence of substantial heterogeneity.

Risk of Bias Legend: (H) High Risk; (L) Low Risk; (U) Unclear. The letters represent the following risk of bias domains: A, random sequence generation (selection bias); B, allocation concealment (selection bias); C, blinding of participants and personnel and outcome assessors (performance bias); D, incomplete outcome data (attrition bias); E, selective reporting (reporting bias); and F, other bias. High other risk of bias (carry-over effect) was given to crossover trials which had no washout between interventions. Trials which did not have this characteristic were rated as Low.

Pooled effect summary calculated with the χ^2 test. Test for group differences calculated with meta-regression, which uses the Wald test.

Supplemental Figure S11: Forest plot of controlled trials of the effect of important food sources of fructose-containing sugars on CRP (mg/L) in subtraction trials

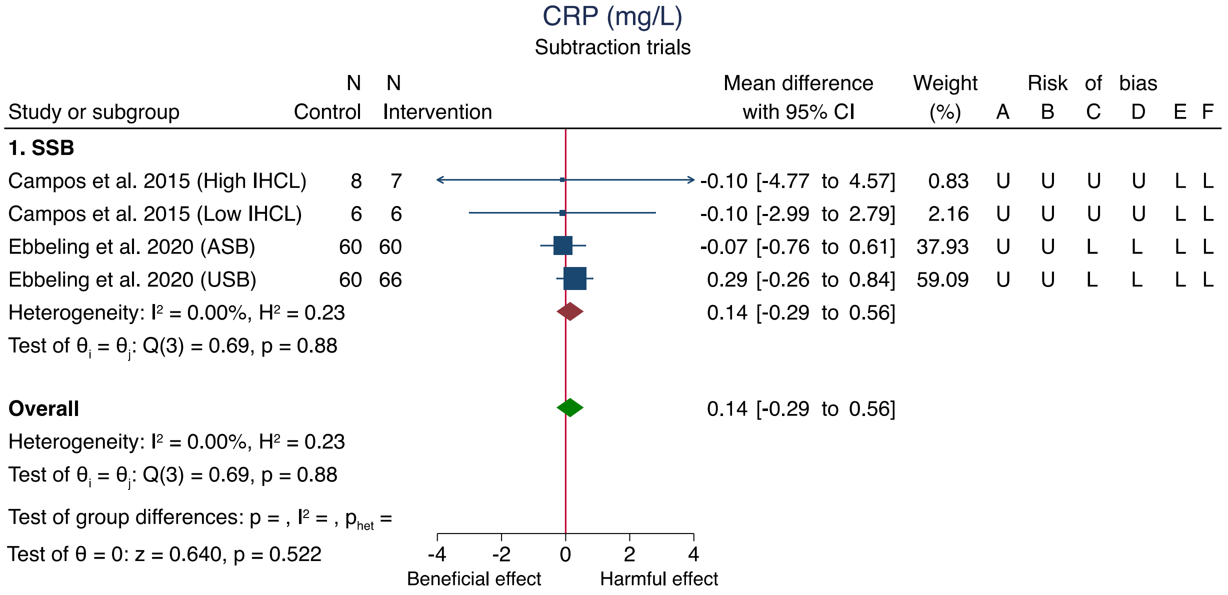

CI, confidence interval; ASB= artificially sweetened beverage; CRP= C reactive protein; IHCL= intrahepatocellular lipid; SSB=sugar-sweetened beverage; USB = unsweetened beverage.

Pooled effect estimates for each subgroup and overall effect are represented by the diamonds. Data are expressed as weighted mean differences with 95% confidence intervals using the generic inverse-variance method and random effects DerSimonian-Laird model. Paired analyses were applied to all crossover trials. Inter-study heterogeneity was assessed using the Cochrane Q statistic and quantified using the I^2 statistic, with significance set at $p<0.100$ and $I^2 \geq 50\%$ considered to be evidence of substantial heterogeneity.

Risk of Bias Legend: (H) High Risk; (L) Low Risk; (U) Unclear. The letters represent the following risk of bias domains: A, random sequence generation (selection bias); B, allocation concealment (selection bias); C, blinding of participants and personnel and outcome assessors (performance bias); D, incomplete outcome data (attrition bias); E, selective reporting (reporting bias); and F, other bias. High other risk of bias (carry-over effect) was given to crossover trials which had no washout between interventions. Trials which did not have this characteristic were rated as Low.

Pooled effect summary calculated with the χ^2 test. Test for group differences calculated with meta-regression, which uses the Wald test.

Supplemental Figure S12: Forest plot of controlled trials of the effect of important food sources of fructose-containing sugars on CRP (mg/L) *ad libitum* trials

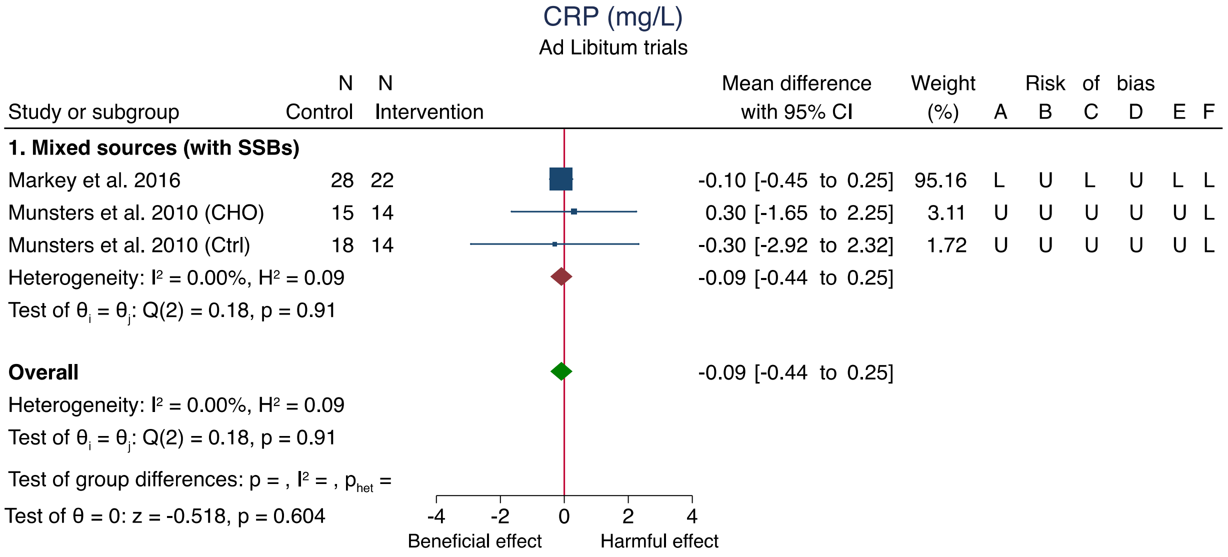

CI, confidence interval; CRP= C reactive protein; CHO= Carbohydrate; Ctrl= Control; SSB= sugar sweetened beverage.

Pooled effect estimates for each subgroup and overall effect are represented by the diamonds. Data are expressed as weighted mean differences with 95% confidence intervals using the generic inverse-variance method and random effects DerSimonian-Laird model. Paired analyses were applied to all crossover trials. Inter-study heterogeneity was assessed using the Cochrane Q statistic and quantified using the I^2 statistic, with significance set at $p<0.100$ and $I^2 \geq 50\%$ considered to be evidence of substantial heterogeneity.

Risk of Bias Legend: (H) High Risk; (L) Low Risk; (U) Unclear. The letters represent the following risk of bias domains: A, random sequence generation (selection bias); B, allocation concealment (selection bias); C, blinding of participants and personnel and outcome assessors (performance bias); D, incomplete outcome data (attrition bias); E, selective reporting (reporting bias); and F, other bias. High other risk of bias (carry-over effect) was given to crossover trials which had no washout between interventions. Trials which did not have this characteristic were rated as Low.

Pooled effect summary calculated with the χ^2 test. Test for group differences calculated with meta-regression, which uses the Wald test.

Supplemental Figure S13: Forest plot of controlled trials of the effect of important food sources of fructose-containing sugars on TNF-α (pg/mL) trials in substitution trials

TNF-a (pg/mL)

Substitution trials

Trial or subgroup	N Control	N Intervention	Mean difference with 95% CI	Weight (%)	Risk of bias A	B	C	D	E	F
**1. SSB**										
Johnston et al. 2013 (T1 Neutral E balance)	17	15	0.06 [-0.12 to 0.24]	13.96	L	L	L	L	L	L
Johnston et al. 2013 (T2 Positive E balance)	17	15	-0.10 [-0.25 to 0.05]	14.89	L	L	L	L	L	L
Heterogeneity: $\tau^2 = 0.01$, $I^2 = 45.68\%$, $H^2 = 1.84$			-0.03 [-0.18 to 0.13]							
Test of $\theta_i = \theta_j$: Q(1) = 1.84, p = 0.17										
**2. Fruit**										
Du et al. 2019	22	27	0.12 [-0.76 to 1.00]	2.52	U	U	L	U	U	L
Fridell et al. 2018	15	15	0.65 [-0.87 to 2.17]	0.92	L	L	L	L	L	L
Lehtonen et al. 2011 (BB)	80	80	-0.20 [-0.45 to 0.05]	11.65	U	U	U	L	H	L
Heterogeneity: $\tau^2 = 0.00$, $I^2 = 0.00\%$, $H^2 = 1.00$			-0.15 [-0.39 to 0.08]							
Test of $\theta_i = \theta_j$: Q(2) = 1.57, p = 0.46										
**3. Dried fruit**										
Kaliora et al. 2016	21	23	0.90 [0.26 to 1.54]	4.20	L	U	L	U	U	L
Kanellos et al. 2014	22	26	-0.20 [-0.66 to 0.26]	6.60	L	U	L	U	L	L
Lehtonen et al. 2011 (SB)	80	80	-0.20 [-0.44 to 0.04]	12.07	U	U	U	L	H	L
Puglisi et al. 2008 R	12	10	-1.51 [-3.91 to 0.89]	0.38	U	U	L	L	U	L
Puglisi et al. 2008 R+W	12	10	0.01 [-3.32 to 3.34]	0.20	U	U	L	L	U	L
Heterogeneity: $\tau^2 = 0.17$, $I^2 = 65.50\%$, $H^2 = 2.90$			0.03 [-0.49 to 0.55]							
Test of $\theta_i = \theta_j$: Q(4) = 11.60, p = 0.02										
**4. Mixed fruit forms**										
Lehtonen et al. 2010	22	28	0.20 [-0.34 to 0.74]	5.37	U	U	U	U	U	L
Heterogeneity: $\tau^2 = 0.00$, $I^2 = .\%$, $H^2 = .$			0.20 [-0.34 to 0.74]							
Test of $\theta_i = \theta_j$: Q(0) = -0.00, p = .										
**5. Mixed sources (with SSBs)**										
Brymora et al. 2012 (T1)	28	28	0.30 [-0.86 to 1.46]	1.54	H	H	H	U	U	H
Brymora et al. 2012 (T2)	28	28	-0.60 [-1.44 to 0.24]	2.69	H	H	H	U	U	H
Khodami et al. 2022	22	21	1.25 [0.45 to 2.05]	2.91	L	L	U	U	L	L
Niholm et al. 2021	80	25	0.59 [-2.45 to 3.63]	0.24	U	U	L	U	L	L
Heterogeneity: $\tau^2 = 0.70$, $I^2 = 69.16\%$, $H^2 = 3.24$			0.35 [-0.69 to 1.39]							
Test of $\theta_i = \theta_j$: Q(3) = 9.73, p = 0.02										
**6. Mixed sources (no SSBs)**										
Goss et al. 2020	19	15	0.10 [-0.42 to 0.62]	5.60	U	L	L	U	L	L
Van Meijl et al. 2011	35	35	0.16 [-0.01 to 0.33]	14.26	U	U	L	U	U	L
Heterogeneity: $\tau^2 = 0.00$, $I^2 = 0.00\%$, $H^2 = 1.00$			0.15 [-0.01 to 0.31]							
Test of $\theta_i = \theta_j$: Q(1) = 0.05, p = 0.83										
**Overall**			0.04 [-0.11 to 0.19]							
Heterogeneity: $\tau^2 = 0.03$, $I^2 = 52.55\%$, $H^2 = 2.11$										
Test of $\theta_i = \theta_j$: Q(16) = 33.72, p = 0.01										

Test of group differences: p = 0.83, $I^2 = 56.97\%$, $p_{het} = 0.01$

Test of $\theta = 0$: z = 0.513, p = 0.608

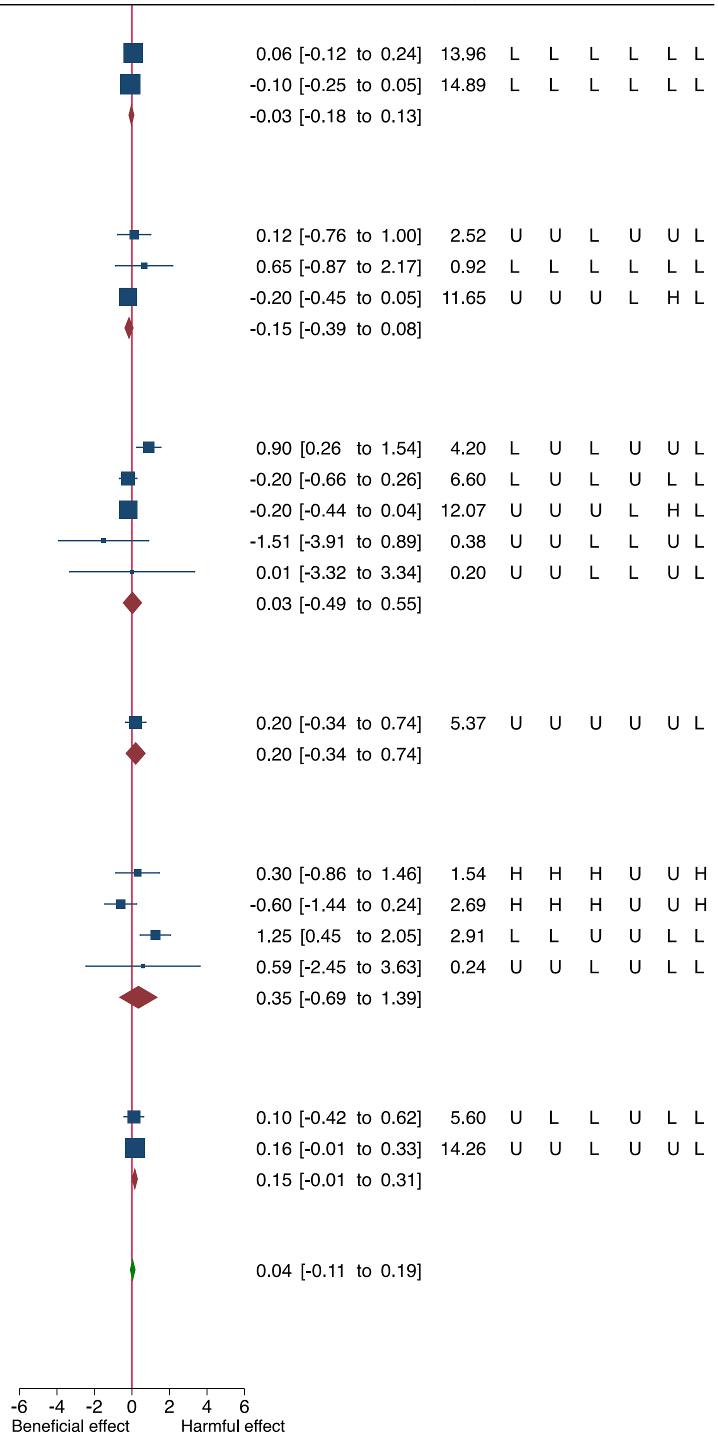

CI, confidence interval; BB= Bilberries; SB= sea buckthorn berries; SSB=sugar-sweetened beverage; TNF-a= Tumour necrosis factor-alpha; R= Raisin; R+W= Raisin + Walk; T1 = Test 1; T2= Test 2

Pooled effect estimates for each subgroup and overall effect are represented by the diamonds. Data are expressed as weighted mean differences with 95% confidence intervals using the generic inverse-variance method and random effects DerSimonian-Laird model. Paired analyses were applied to all crossover trials. Inter-study heterogeneity was assessed using the Cochrane Q statistic and quantified using the I^2 statistic, with significance set at $p<0.100$ and $I^2 \geq 50\%$ considered to be evidence of substantial heterogeneity.

Risk of Bias Legend: (H) High Risk; (L) Low Risk; (U) Unclear. The letters represent the following risk of bias domains: A, random sequence generation (selection bias); B, allocation concealment (selection bias); C, blinding of participants and personnel and outcome assessors (performance bias); D, incomplete outcome data (attrition bias); E, selective reporting (reporting bias); and F, other bias. High other risk of bias (carry-over effect) was given to crossover trials which had no washout between interventions. Trials which did not have this characteristic were rated as Low.

Pooled effect summary calculated with the χ^2 test. Test for group differences calculated with meta-regression, which uses the Wald test.

Supplemental Figure S14: Forest plot of controlled trials of the effect of important food sources of fructose-containing sugars on TNF-α (pg/mL) trials in addition trials

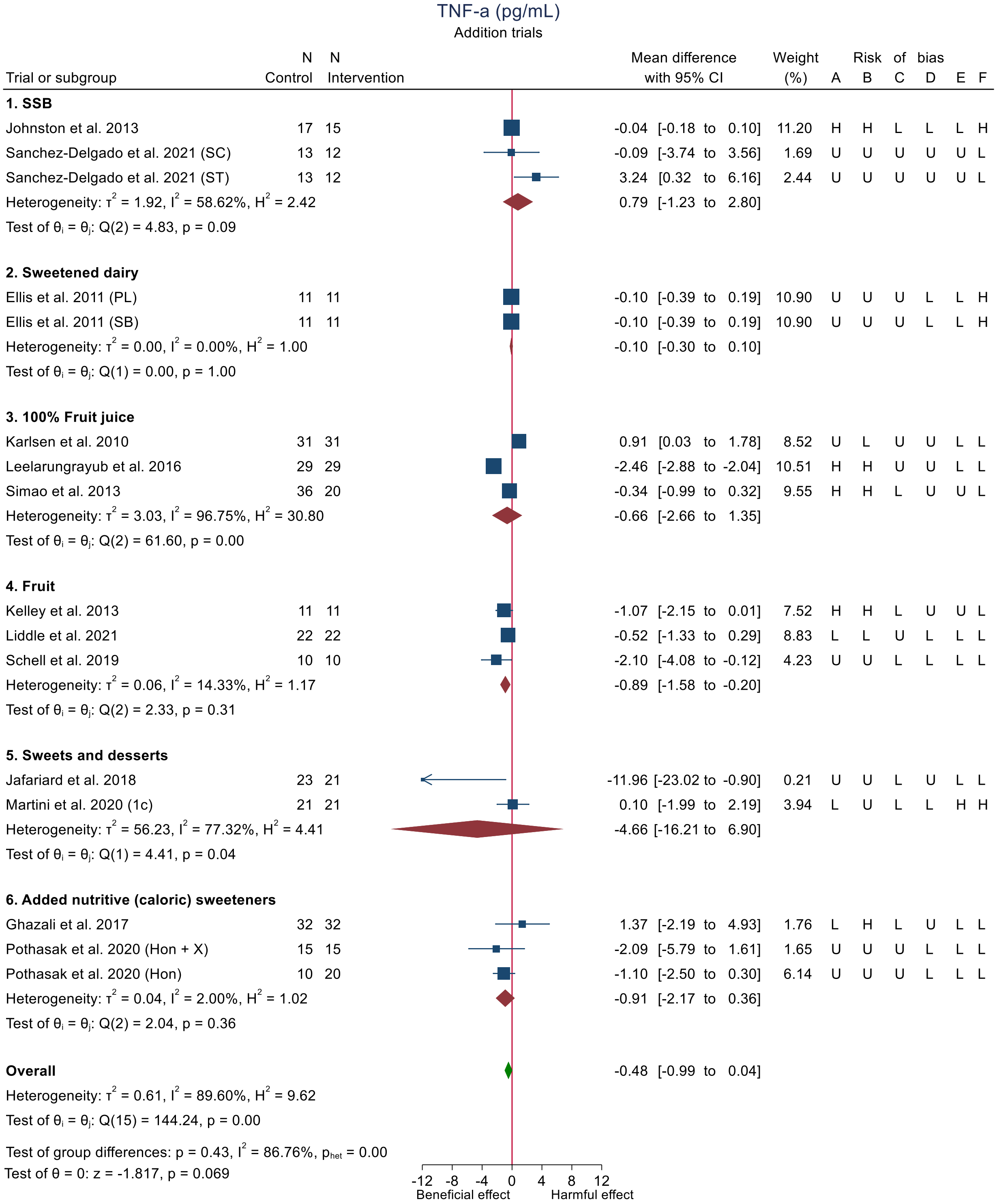

CI, confidence interval; Hon= Honey; Hon + X= Honey + exercise; PL= Placebo; SB= Strawberry; SC= Sucralose; SSB=sugar-sweetened beverage; ST= Steviol; TNF= Tumour necrosis factor-alpha; 1c= 1 cup.
Pooled effect estimates for each subgroup and overall effect are represented by the diamonds. Data are expressed as weighted mean differences with 95% confidence intervals using the generic inverse-variance method and random effects DerSimonian-Laird model. Paired analyses were applied to all crossover trials. Inter-study heterogeneity was assessed using the Cochrane Q statistic and quantified using the I^2 statistic, with significance set at $p<0.100$ and $I^2 \geq 50\%$ considered to be evidence of substantial heterogeneity.
Risk of Bias Legend: (H) High Risk; (L) Low Risk; (U) Unclear. The letters represent the following risk of bias domains: A, random sequence generation (selection bias); B, allocation concealment (selection bias); C, blinding of participants and personnel and outcome assessors (performance bias); D, incomplete outcome data (attrition bias); E, selective reporting (reporting bias); and F, other bias. High other risk of bias (carry-over effect) was given to crossover trials which had no washout between interventions. Trials which did not have this characteristic were rated as Low.
Pooled effect summary calculated with the χ^2 test. Test for group differences calculated with meta-regression, which uses the Wald test.

Supplemental Figure S15: Forest plot of controlled trials of the effect of important food sources of fructose-containing sugars on IL-6 (pg/mL) in substitution trials

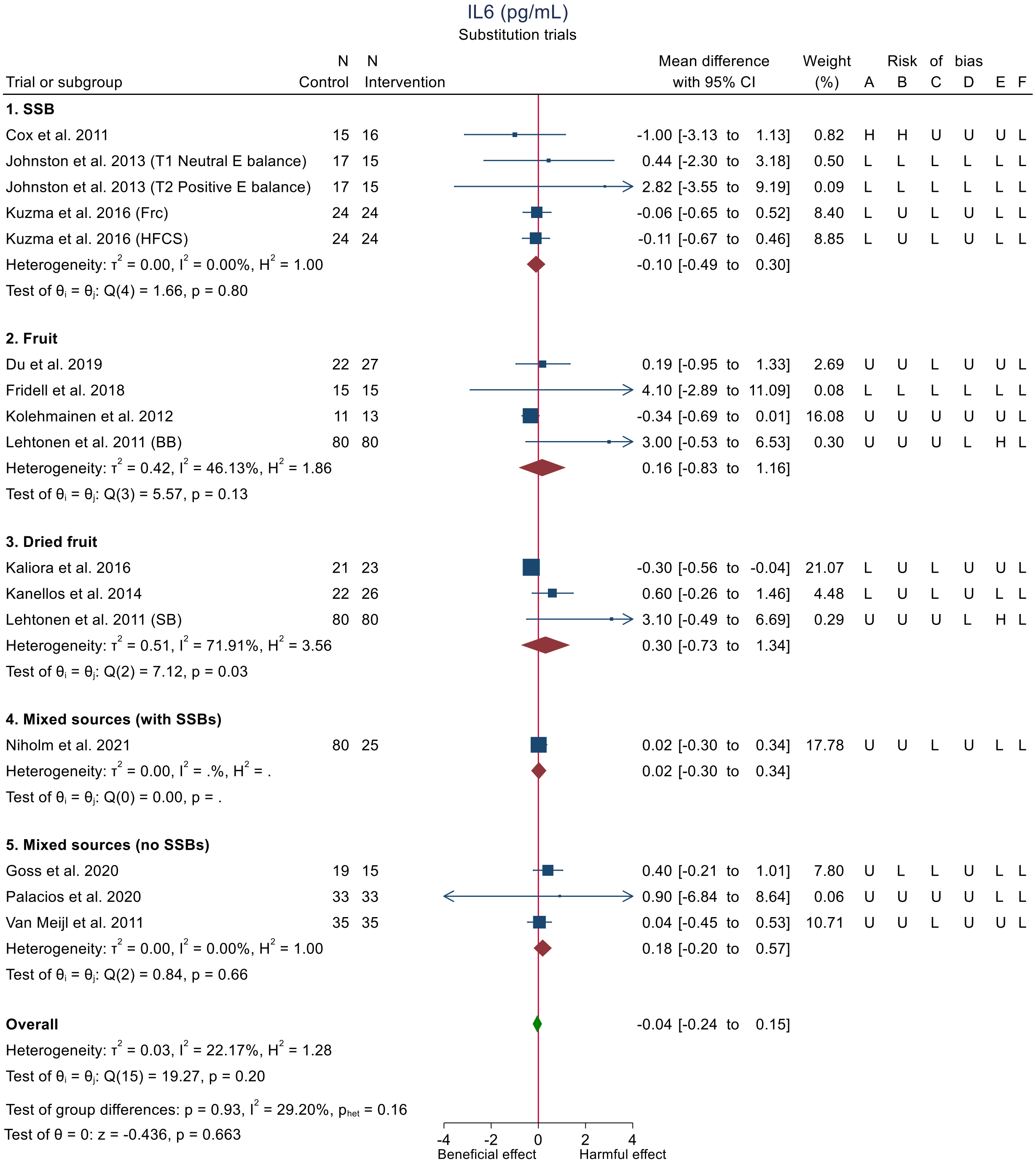

CI, confidence interval; IL6= Interleukin 6; BB= Bilberries; Frc= Fructose; HFCS= High fructose corn syrup; SB= sea buckthorn berries; SSB=sugar-sweetened beverage; T1= Test 1; T2= Test 2

Pooled effect estimates for each subgroup and overall effect are represented by the diamonds. Data are expressed as weighted mean differences with 95% confidence intervals using the generic inverse-variance method and random effects DerSimonian-Laird model. Paired analyses were applied to all crossover trials. Inter-study heterogeneity was assessed using the Cochrane Q statistic and quantified using the I^2 statistic, with significance set at $p<0.100$ and $I^2 \geq 50\%$ considered to be evidence of substantial heterogeneity.

Risk of Bias Legend: (H) High Risk; (L) Low Risk; (U) Unclear. The letters represent the following risk of bias domains: A, random sequence generation (selection bias); B, allocation concealment (selection bias); C, blinding of participants and personnel and outcome assessors (performance bias); D, incomplete outcome data (attrition bias); E, selective reporting (reporting bias); and F, other bias. High other risk of bias (carry-over effect) was given to crossover trials which had no washout between interventions. Trials which did not have this characteristic were rated as Low.
Pooled effect summary calculated with the χ^2 test. Test for group differences calculated with meta-regression, which uses the Wald test.

Supplemental Figure S16: Forest plot of controlled trials of the effect of important food sources of fructose-containing sugars on IL-6 in addition trials

IL-6 (pg/mL)

Addition trials

Trial or subgroup	N Control	N Intervention	Mean difference with 95% CI	Weight (%)	Risk of bias A	B	C	D	E	F
**1. SSB**										
Johnston et al. 2013	12	10	1.14 [-0.93 to 3.21]	1.93	H	H	L	L	L	H
Sanchez-Delgado et al. 2021 (SC)	13	12	0.97 [-1.11 to 3.04]	1.93	U	U	U	U	U	L
Sanchez-Delgado et al. 2021 (ST)	13	12	1.16 [-0.82 to 3.14]	2.10	U	U	U	U	U	L
Zafrilla et al. 2021 (SC)	45	45	0.13 [-0.25 to 0.51]	13.00	U	L	L	L	L	L
Zafrilla et al. 2021 (ST)	46	45	-0.27 [-0.66 to 0.12]	12.86	U	L	L	L	L	L
Heterogeneity: $\tau^2 = 0.05$, $I^2 = 28.95\%$, $H^2 = 1.41$			0.06 [-0.34 to 0.46]							
Test of $\theta_i = \theta_j$: Q(4) = 5.63, p = 0.23										
**2. Sweetened dairy**										
Ellis et al. 2011 (PL)	11	11	-0.70 [-0.90 to -0.50]	15.15	U	U	U	L	L	H
Ellis et al. 2011 (SB)	11	11	0.00 [-0.98 to 0.98]	6.10	U	U	U	L	L	H
Heterogeneity: $\tau^2 = 0.11$, $I^2 = 46.57\%$, $H^2 = 1.87$			-0.52 [-1.12 to 0.08]							
Test of $\theta_i = \theta_j$: Q(1) = 1.87, p = 0.17										
**3. 100% Fruit juice**										
Asgary et al. 2014	10	11	-2.47 [-5.89 to 0.95]	0.77	H	H	L	L	L	L
Fatel et al. 2021	21	20	-5.72 [-10.78 to -0.67]	0.36	U	U	L	U	L	L
Karlsen et al. 2010	31	31	-14.53 [-27.71 to -1.36]	0.05	U	L	U	U	L	L
Simao et al. 2013	36	20	0.40 [-0.89 to 1.69]	4.22	H	H	L	U	U	L
Heterogeneity: $\tau^2 = 9.89$, $I^2 = 74.08\%$, $H^2 = 3.86$			-3.01 [-6.91 to 0.88]							
Test of $\theta_i = \theta_j$: Q(3) = 11.57, p = 0.01										
**4. Fruit**										
Basu et al. 2010	23	25	0.00 [-0.03 to 0.03]	16.22	U	U	L	U	L	L
Liddle et al. 2021	22	22	-0.35 [-0.57 to -0.14]	15.05	L	L	U	L	L	L
Schell et al. 2019	10	10	-7.30 [-14.18 to -0.42]	0.20	U	U	L	L	L	L
Heterogeneity: $\tau^2 = 0.08$, $I^2 = 86.45\%$, $H^2 = 7.38$			-0.19 [-0.59 to 0.21]							
Test of $\theta_i = \theta_j$: Q(2) = 14.76, p = 0.00										
**5. Sweets and desserts**										
Jafariard e al. 2018	23	21	-8.79 [-14.26 to -3.32]	0.31	U	U	L	U	L	L
Heterogeneity: $\tau^2 = 0.00$, $I^2 = .\%$, $H^2 = .$			-8.79 [-14.26 to -3.32]							
Test of $\theta_i = \theta_j$: Q(0) = 0.00, p = .										
**6. Added nutritive (caloric) sweetener**										
Ghazali et al. 2017	32	32	0.40 [-0.22 to 1.02]	9.75	L	H	L	U	L	L
Heterogeneity: $\tau^2 = 0.00$, $I^2 = .\%$, $H^2 = .$			0.40 [-0.22 to 1.02]							
Test of $\theta_i = \theta_j$: Q(0) = 0.00, p = .										
**Overall**			-0.15 [-0.45 to 0.16]							
Heterogeneity: $\tau^2 = 0.15$, $I^2 = 82.90\%$, $H^2 = 5.85$										
Test of $\theta_i = \theta_j$: Q(15) = 87.72, p = 0.00										

Test of group differences: p = 0.02, $I^2 = 79.67\%$, $p_{het} = 0.00$

Test of θ = 0: z = -0.931, p = 0.352

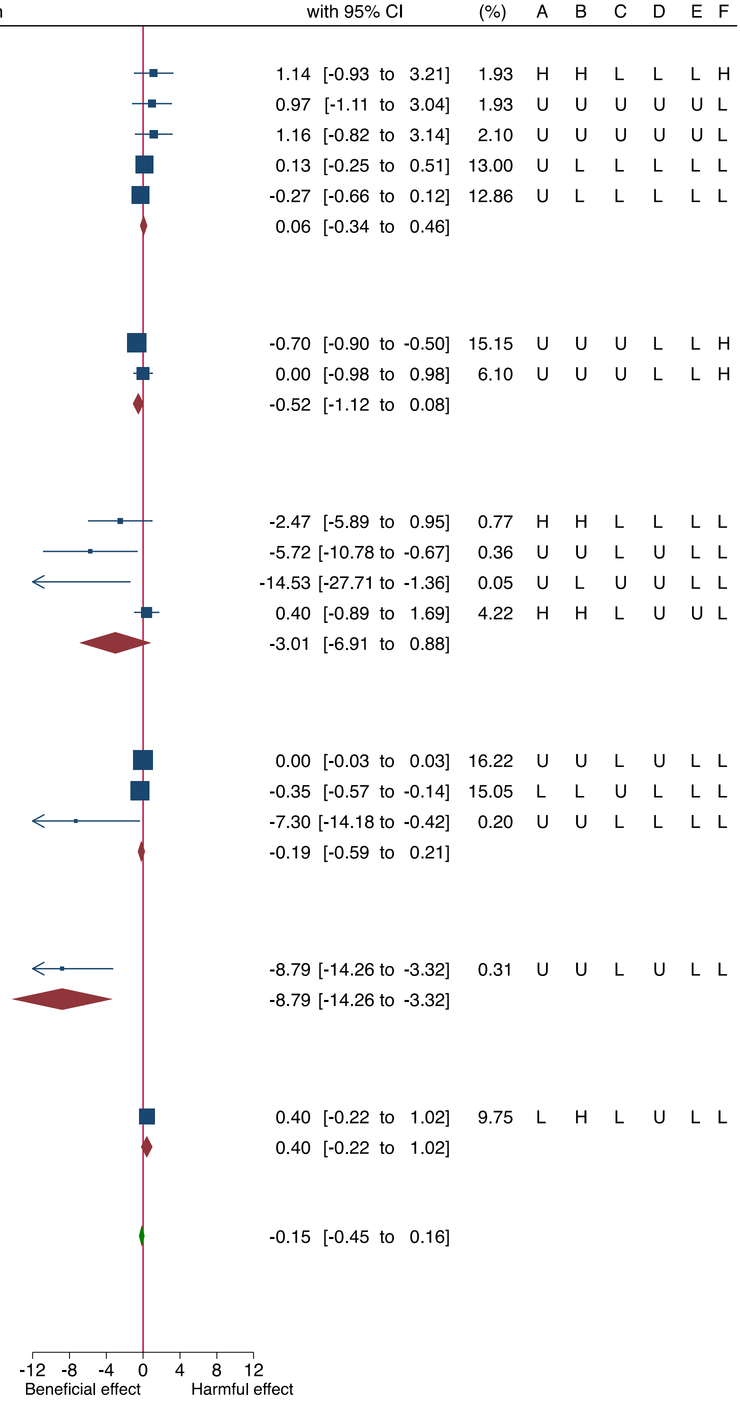

CI, confidence interval; IL-6= Interleukin 6; PL= Placebo; SB= Strawberries; SC= Sucralose; SSB=sugar-sweetened beverage; ST= Steviol

Pooled effect estimates for each subgroup and overall effect are represented by the diamonds. Data are expressed as weighted mean differences with 95% confidence intervals using the generic inverse-variance method and random effects DerSimonian-Laird model. Paired analyses were applied to all crossover trials. Inter-study heterogeneity was assessed using the Cochrane Q statistic and quantified using the I^2 statistic, with significance set at $p<0.100$ and $I^2 \geq 50\%$ considered to be evidence of substantial heterogeneity.

Risk of Bias Legend: (H) High Risk; (L) Low Risk; (U) Unclear. The letters represent the following risk of bias domains: A, random sequence generation (selection bias); B, allocation concealment (selection bias); C, blinding of participants and personnel and outcome assessors (performance bias); D, incomplete outcome data (attrition bias); E, selective reporting (reporting bias); and F, other bias. High other risk of bias (carry-over effect) was given to crossover trials which had no washout between interventions. Trials which did not have this characteristic were rated as Low.

Pooled effect summary calculated with the χ^2 test. Test for group differences calculated with meta-regression, which uses the Wald test.

Supplemental Figure S17: Sensitivity analysis of the systematic removal of each trial for the effect of important food sources of fructose-containing sugars on CRP (mg/L) in substitution trials

Influence analysis

CRP (mg/L) in substitution trials

Trial removed	Mean difference with 95% CI	P_{effect}	I^2 (%)	P_Q
**Overall**	0.07 [-0.08 to 0.22]	0.336	54	< 0.001
Aeberli et al. 2011 (HF vs HG)	0.07 [-0.09 to 0.23]	0.346	55	< 0.001
Aeberli et al. 2011 (HS vs HG)	0.08 [-0.08 to 0.23]	0.344	55	< 0.001
Aeberli et al. 2011 (MF vs MG)	0.08 [-0.08 to 0.23]	0.328	55	< 0.001
Angelopoulos et al. 2016 (FRC)	0.07 [-0.08 to 0.22]	0.380	55	< 0.001
Angelopoulos et al. 2016 (HFCS)	0.08 [-0.07 to 0.23]	0.298	55	< 0.001
Angelopoulos et al. 2016 (SUC)	0.08 [-0.07 to 0.23]	0.299	55	< 0.001
Brymora et al. 2012 (T1)	0.06 [-0.09 to 0.21]	0.451	53	< 0.001
Brymora et al. 2012 (T2)	0.06 [-0.09 to 0.21]	0.412	54	< 0.001
Chiu et al. 2020	0.07 [-0.09 to 0.23]	0.359	55	< 0.001
Cox et al. 2011	0.07 [-0.08 to 0.22]	0.384	54	< 0.001
Eslami et al. 2019	0.11 [-0.04 to 0.25]	0.157	50	< 0.001
Fridell et al. 2018	0.09 [-0.06 to 0.25]	0.276	55	< 0.001
Goss et al. 2020	0.08 [-0.08 to 0.23]	0.330	55	< 0.001
Jalilvand et al. 2020	0.03 [-0.11 to 0.17]	0.709	41	0.007
Jin et al. 2014	0.05 [-0.10 to 0.20]	0.465	51	< 0.001
Johnston et al. 2013 (T1 Neutral E balance)	0.08 [-0.07 to 0.23]	0.285	55	< 0.001
Johnston et al. 2013 (T2 Positive E balance)	0.08 [-0.07 to 0.23]	0.315	54	< 0.001
Kaliora et al. 2016	0.07 [-0.08 to 0.22]	0.353	55	< 0.001
Kanellos et al. 2014	0.06 [-0.09 to 0.21]	0.437	54	< 0.001
Kanellos et al. 2017	0.07 [-0.08 to 0.22]	0.351	55	< 0.001
Khodami et al. 2022	0.04 [-0.11 to 0.19]	0.612	47	0.001
Kolehmainen et al. 2012	0.10 [-0.05 to 0.25]	0.197	52	< 0.001
Kuzma et al. 2016 (Frc)	0.07 [-0.09 to 0.23]	0.345	55	< 0.001
Kuzma et al. 2016 (HFCS)	0.08 [-0.08 to 0.23]	0.331	55	< 0.001
Lehtonen et al. 2010	0.08 [-0.08 to 0.23]	0.327	55	< 0.001
Lehtonen et al. 2011 (BB)	0.07 [-0.08 to 0.22]	0.377	55	< 0.001
Lehtonen et al. 2011 (SB)	0.07 [-0.09 to 0.23]	0.372	55	< 0.001
Maki et al. 2020	0.07 [-0.09 to 0.23]	0.341	55	< 0.001
Moazen et al. 2013	0.10 [-0.05 to 0.25]	0.176	51	< 0.001
Navaei et al. 2019	0.08 [-0.07 to 0.23]	0.306	55	< 0.001
Niholm et al. 2021	0.07 [-0.08 to 0.22]	0.365	55	< 0.001
Palacios et al. 2020	0.07 [-0.09 to 0.23]	0.336	55	< 0.001
Ponce et al. 2019	0.08 [-0.07 to 0.23]	0.300	54	< 0.001
Ribeiro et al. 2017	0.09 [-0.05 to 0.24]	0.215	52	< 0.001
Sadeghi et al. 2020	0.08 [-0.10 to 0.26]	0.413	53	< 0.001
Souto et al 2013	0.07 [-0.11 to 0.25]	0.417	55	< 0.001
Van Meijl et al. 2011	0.06 [-0.09 to 0.21]	0.448	54	< 0.001

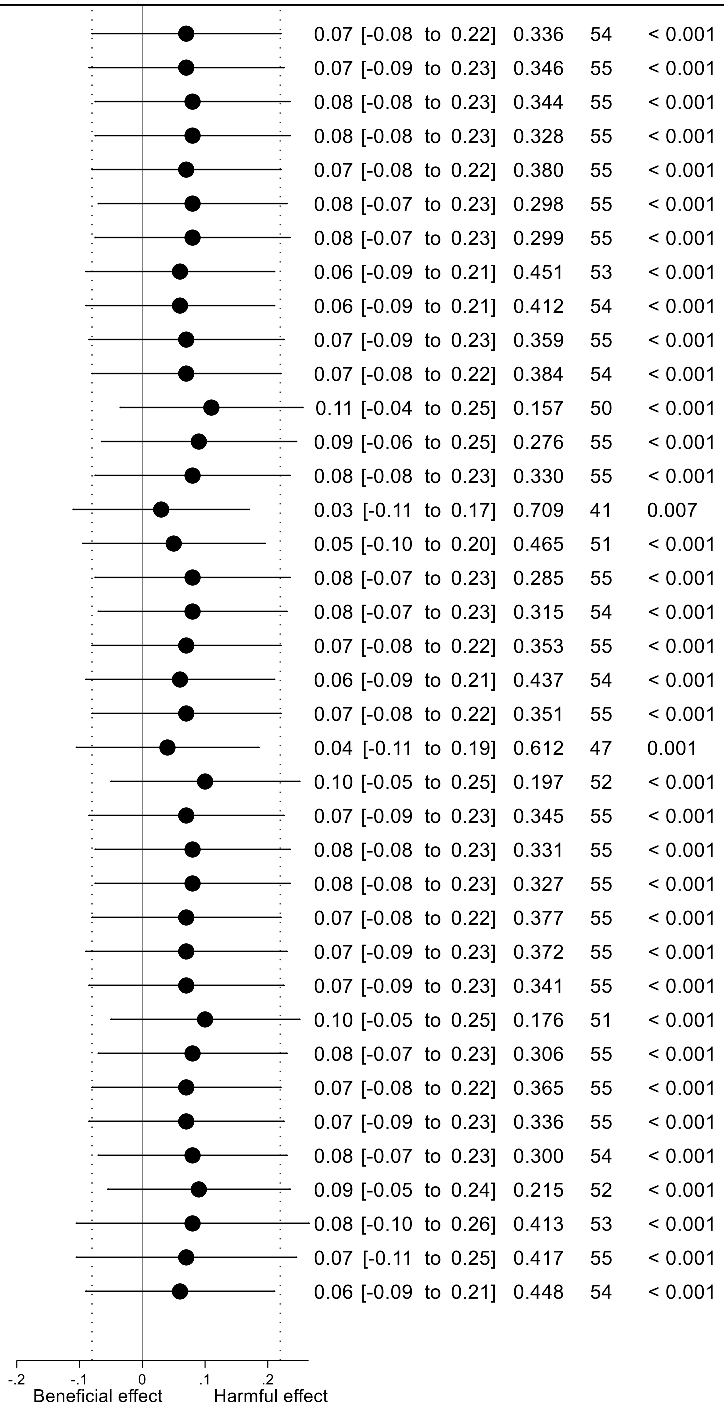

Influence analysis: removal of each trial, one at a time and recalculation of the overall effect and heterogeneity

CI, confidence interval; CRP= C reactive protein; BB= Bilberries; FRC= fructose; HG=high glucose; HS=high sucrose; HF=high fructose; MG=medium glucose; MF= medium fructose; HFCS=high-fructose corn syrup; SB= sea buckthorn berries; SSB=sugar-sweetened beverage; SUC= sucrose; T1= test 1; T2= Test 2

Supplemental Figure S18: Sensitivity analysis of the systematic removal of each trial for the effect of important food sources of fructose-containing sugars on CRP (mg/L) in addition trials

Influence analysis

CRP (mg/L) in addition trials

Trial removed	Mean difference with 95% CI	P_{effect}	I^2 (%)	P_Q
**Overall**	-0.15 [-0.28 to -0.02]	0.020	28	0.064
Aeberli et al. 2011 (HF)	-0.17 [-0.31 to -0.03]	0.015	29	0.057
Aeberli et al. 2011 (HS)	-0.17 [-0.31 to -0.03]	0.015	29	0.056
Aeberli et al. 2011 (MF)	-0.16 [-0.30 to -0.02]	0.017	29	0.054
Aghababaee et al. 2015	-0.16 [-0.30 to -0.02]	0.023	30	0.051
Alavinejad et al. 2015	-0.16 [-0.29 to -0.03]	0.023	30	0.051
Asgary et al. 2014	-0.15 [-0.29 to -0.02]	0.021	30	0.051
Basu et al. 2010	-0.15 [-0.29 to -0.02]	0.021	30	0.051
Blum et al. 2007	-0.15 [-0.29 to -0.02]	0.021	30	0.051
Castilla et al. 2008	-0.15 [-0.29 to -0.02]	0.020	30	0.052
Castilla et al. 2008 Vit E	-0.15 [-0.29 to -0.02]	0.020	30	0.052
Dow et al. 2013	-0.14 [-0.27 to -0.01]	0.036	27	0.075
Ellis et al. 2011 (PL)	-0.16 [-0.28 to -0.04]	0.011	26	0.085
Ellis et al. 2011 (SB)	-0.15 [-0.28 to -0.02]	0.024	30	0.053
Franck et al. 2020	-0.14 [-0.27 to -0.01]	0.028	27	0.077
Ghazali et al. 2017	-0.16 [-0.29 to -0.03]	0.020	30	0.052
Hooshmand et al. 2016 (HD)	-0.16 [-0.29 to -0.03]	0.019	30	0.053
Hooshmand et al. 2016 (LD)	-0.16 [-0.29 to -0.03]	0.020	30	0.052
Irannejad et al. 2020	-0.15 [-0.28 to -0.02]	0.021	28	0.062
Jafariard e al. 2018	-0.10 [-0.20 to 0.00]	0.055	9	0.311
Johnston et al. 2013	-0.13 [-0.25 to -0.01]	0.037	21	0.132
Karlsen et al. 2010	-0.13 [-0.26 to 0.00]	0.047	25	0.097
Kelley et al. 2013	-0.12 [-0.24 to 0.01]	0.048	22	0.130
Liddle et al. 2021	-0.13 [-0.26 to 0.00]	0.049	24	0.107
Mietus-Snyder et al. 2012	-0.15 [-0.29 to -0.02]	0.031	29	0.059
Njike et al. 2011	-0.17 [-0.30 to -0.04]	0.012	27	0.070
Ravn-Haren et al. 2013 (Cld vs Ctrl)	-0.16 [-0.29 to -0.03]	0.019	30	0.052
Ravn-Haren et al. 2013 (Cld vs Pom)	-0.17 [-0.31 to -0.03]	0.013	28	0.062
Ravn-Haren et al. 2013 (Clr vs Ctrl)	-0.16 [-0.29 to -0.03]	0.018	29	0.054
Ravn-Haren et al. 2013 (Clr vs Pom)	-0.17 [-0.30 to -0.04]	0.013	28	0.066
Ravn-Haren et al. 2013 (Clr)	-0.15 [-0.28 to -0.02]	0.024	30	0.053
Ravn-Haren et al. 2013 (Pom)	-0.16 [-0.29 to -0.03]	0.022	30	0.051
Schell et al. 2019	-0.15 [-0.29 to -0.02]	0.021	30	0.052
Simao et al. 2013	-0.16 [-0.29 to -0.03]	0.019	29	0.055
Thimoteo et al. 2019	-0.16 [-0.29 to -0.03]	0.020	30	0.051
Vaz et al. 2011 (Fort)	-0.18 [-0.32 to -0.05]	0.006	19	0.170
Vaz et al. 2011 (Unfort)	-0.17 [-0.31 to -0.03]	0.020	29	0.056

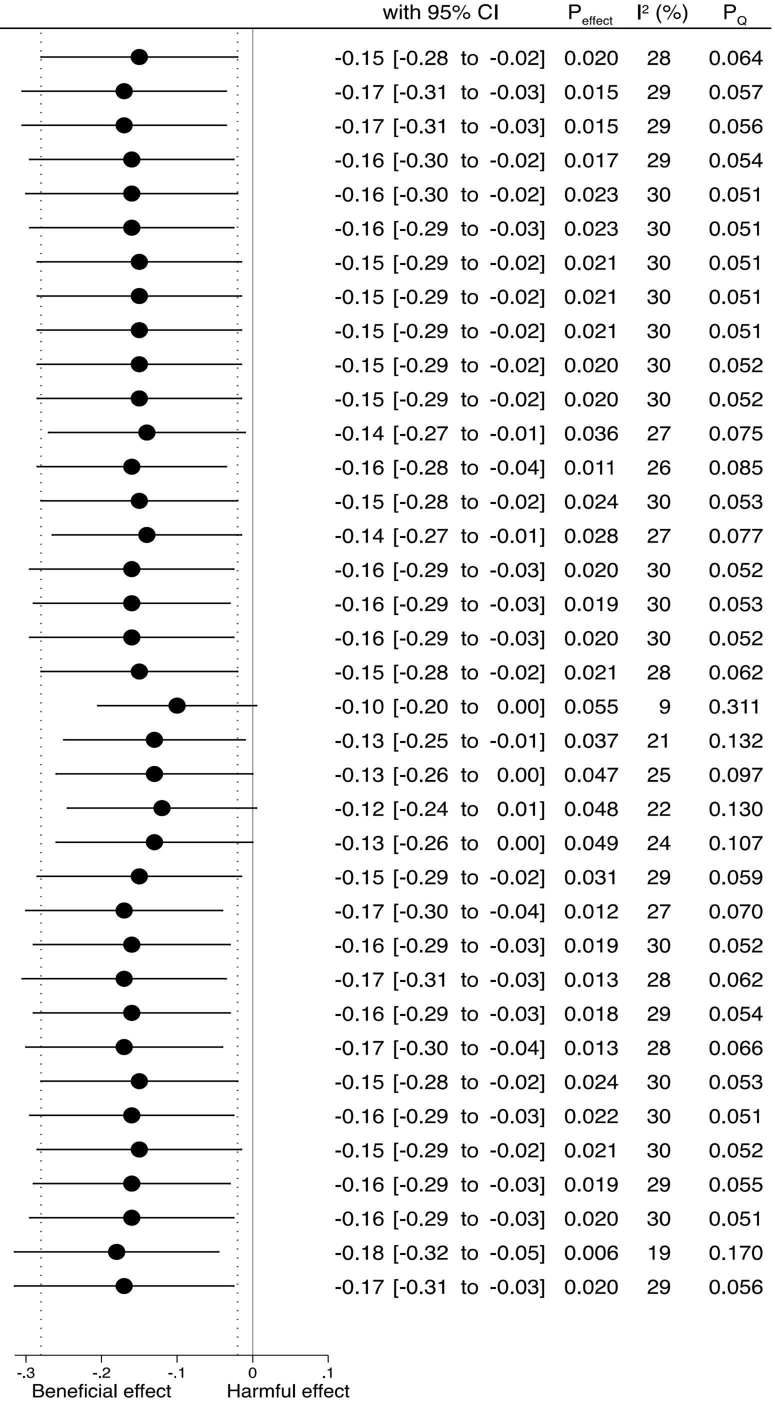

Influence analysis: removal of each trial, one at a time and recalculation of the overall effect and heterogeneity

CI, confidence interval; CRP= C reactive protein; Cld=Cloudy; Ctrl=Control; Clr=Clear; Pom=Pomace; Frc= fructose; fort= fortified; HS=high sucrose; HF=high fructose; MF= medium fructose; HD=higher dose; LD=lower dose; HFCS=high-fructose corn syrup; PB=placebo; SB= strawberries; SSB=sugar-sweetened beverage; unfort= unfortified; Vit E= vitamin E

Supplemental Figure S19: Sensitivity analysis of the systematic removal of each trial in the primary analysis of the effect of important food sources of fructose-containing sugars on CRP (mg/L) in subtraction trials

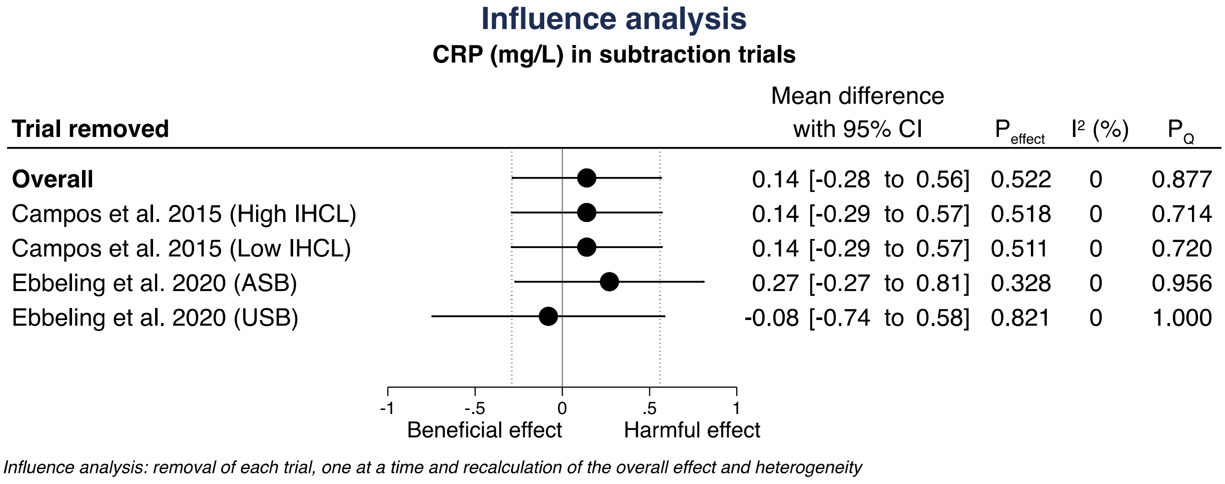

CI, confidence interval; ASB= artificially sweetened beverage; CRP= C reactive protein; IHCL=intrahepatocellular lipid; USB= unsweetened beverage

Supplemental Figure S20: Sensitivity analysis of the systematic removal of each trial for the effect of important food sources of fructose-containing sugars on CRP (mg/L) in *ad libitum* trials

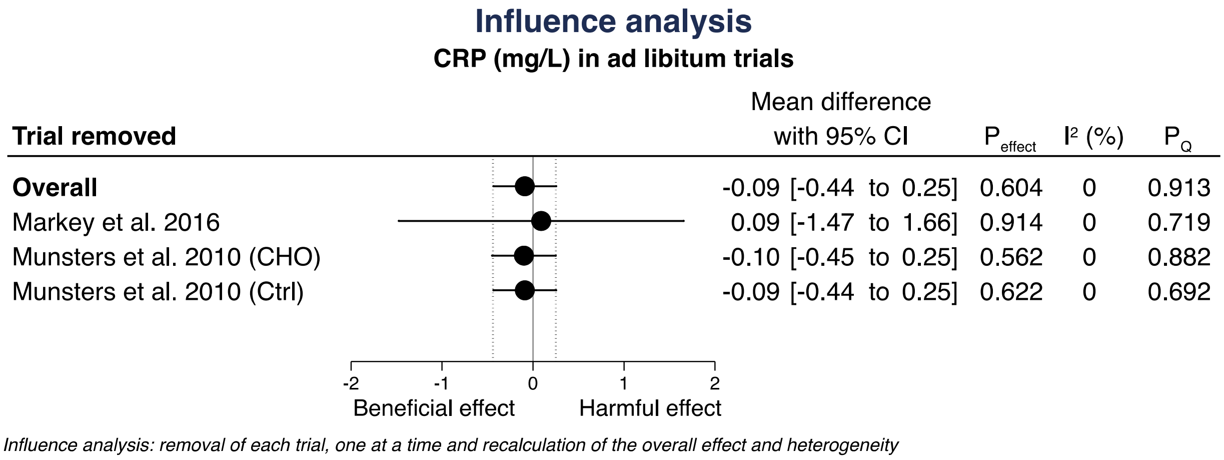

CI, confidence interval; CRP= C reactive protein; CHO= Carbohydrate; Ctrl= Control

Supplemental Figure S21: Sensitivity analysis of the systematic removal of each trial for the effect of SSB on CRP (mg/L) in substitution trials

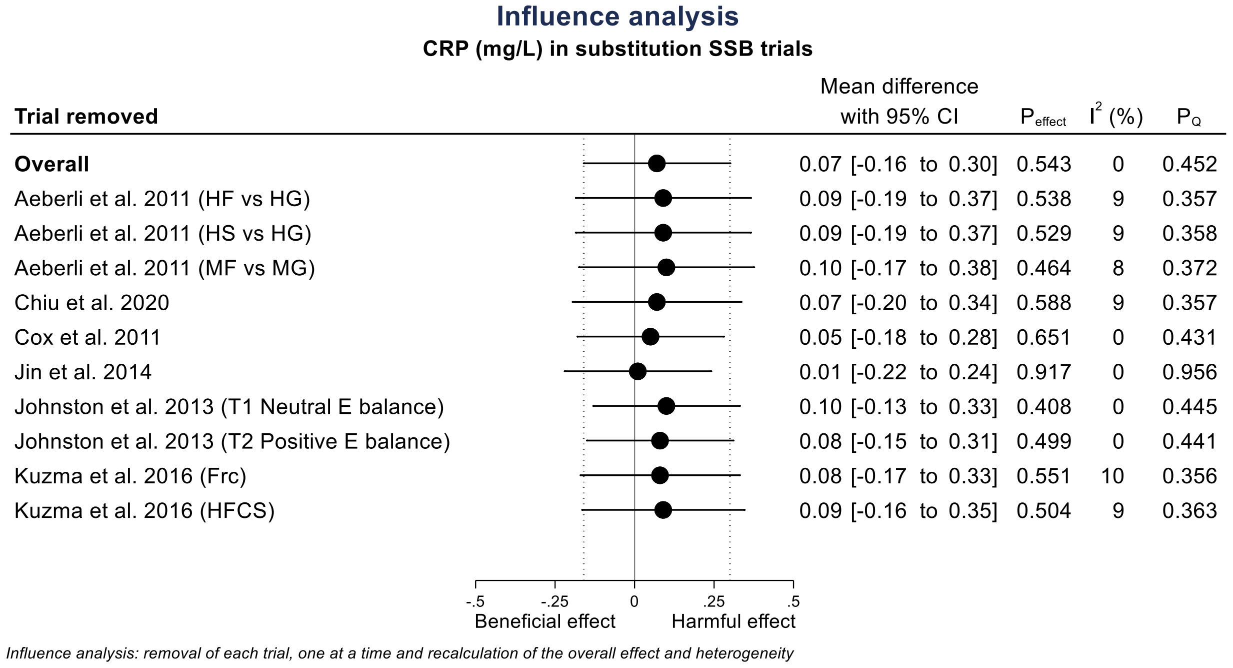

CI, confidence interval; CRP= C reactive protein; BB= Bilberries; Frc= fructose; HG=high glucose; HS=high sucrose; HF=high fructose; MG=medium glucose; MF= medium fructose; HFCS=high-fructose corn syrup; SB= sea buckthorn berries; SSB=sugar-sweetened beverage; T1= Test 1; T2= Test 2

Supplemental Figure S22: Sensitivity analysis of the systematic removal of each trial for the effect of sweetened dairy on CRP (mg/L) in substitution trials

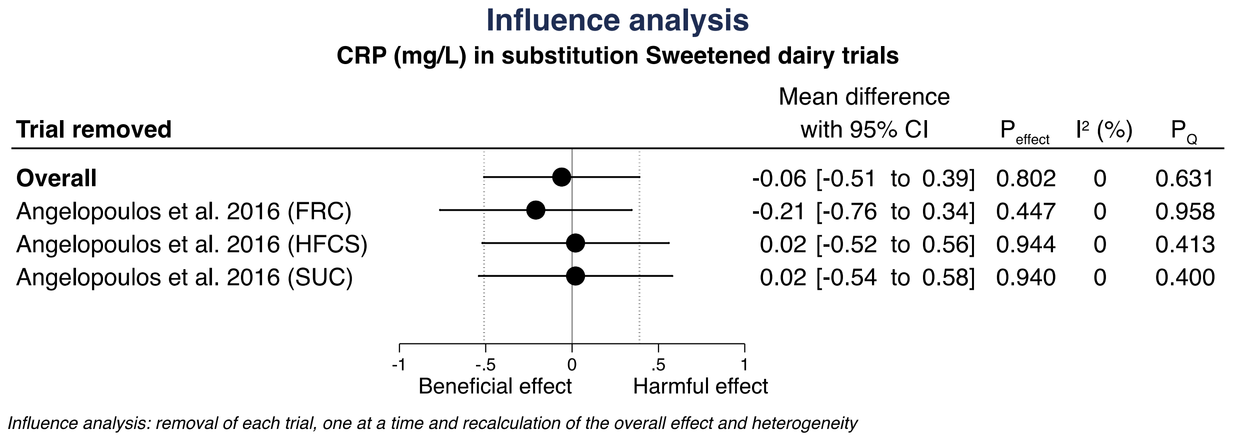

CI, confidence interval; CRP= C reactive protein; FRC= Fructose; HFCS= High fructose corn syrup; SUC= sucrose

Supplemental Figure S23: Sensitivity analysis of the systematic removal of each trial for the effect of 100% fruit juice on CRP (mg/L) in substitution trials

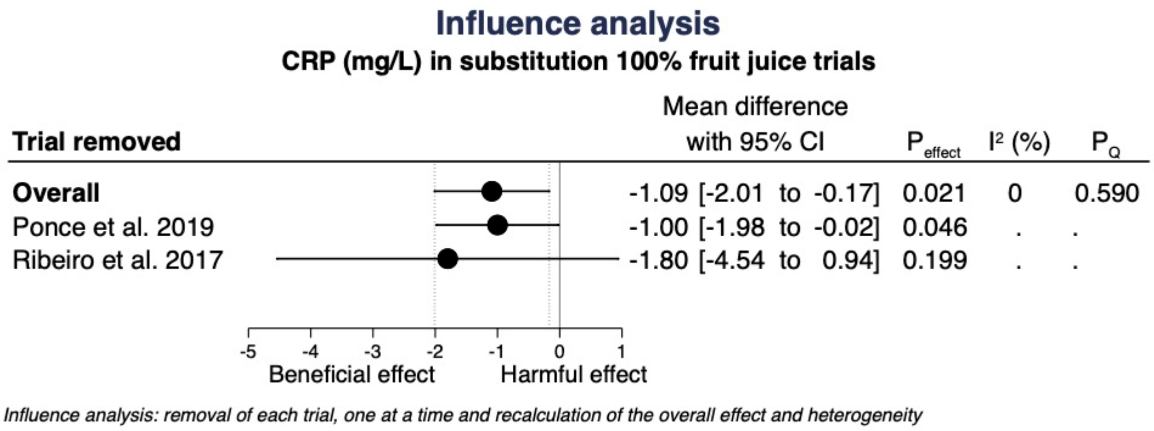

CI, confidence interval; CRP= C reactive protein

Supplemental Figure S24: Sensitivity analysis of the systematic removal of each trial for the effect of fruit on CRP (mg/L) in substitution trials

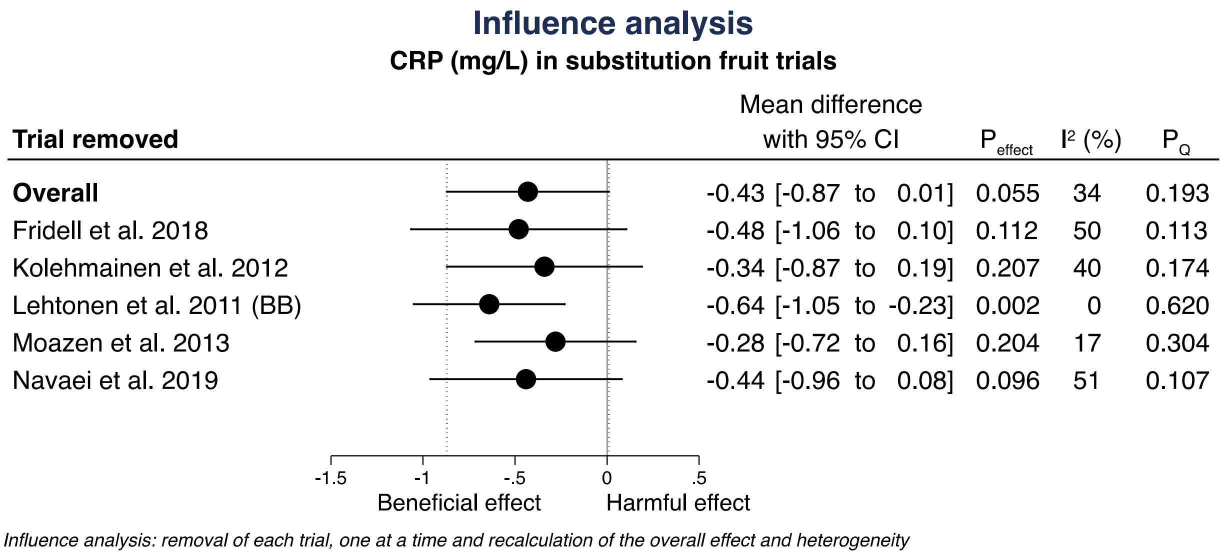

CI, confidence interval; CRP= C reactive protein; BB= Bilberries

Supplemental Figure S25: Sensitivity analysis of the systematic removal of each trial for the effect of dried fruit on CRP (mg/L) in substitution trials

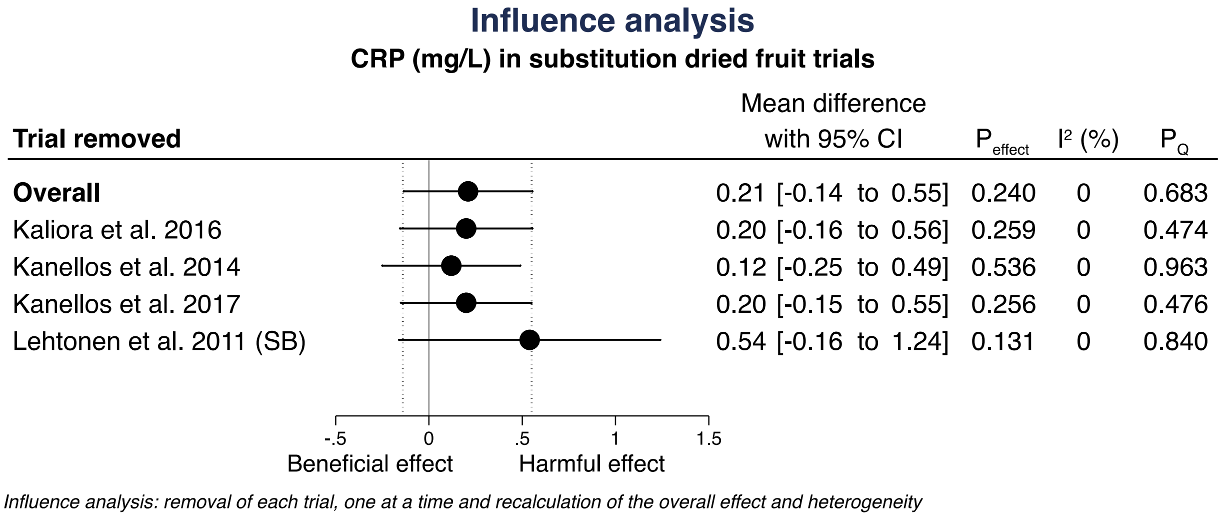

CI, confidence interval; CRP= C reactive protein; SB= seabuckthorn berries

Supplemental Figure S26: Sensitivity analysis of the systematic removal of each trial for the effect of mixed sources (with SSBs) on CRP (mg/L) in substitution trials

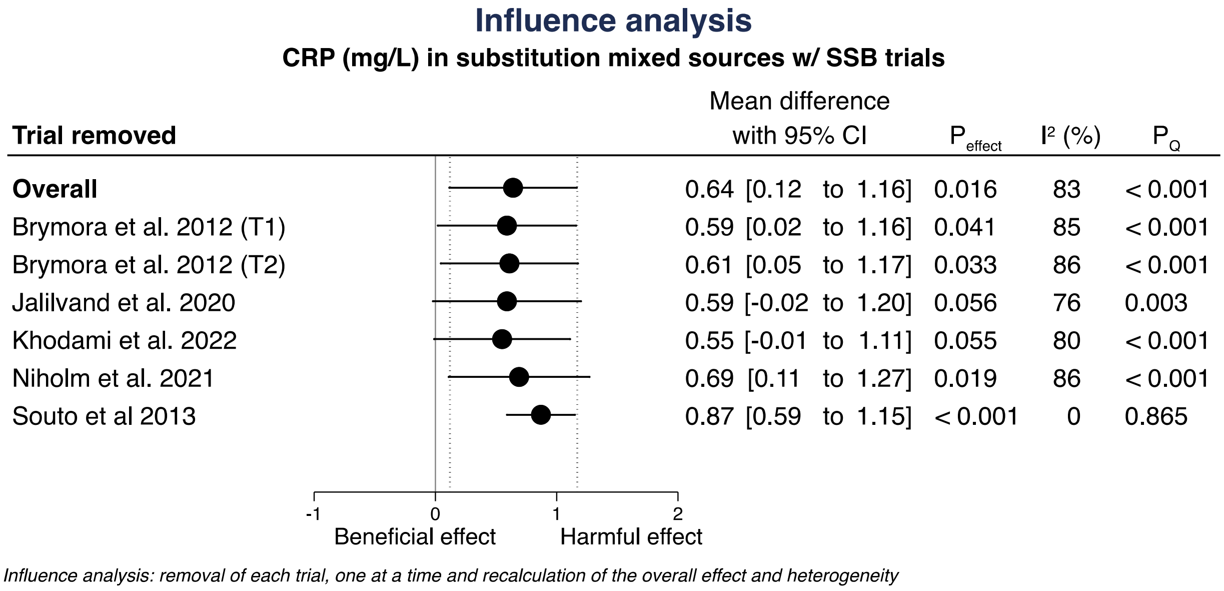

CI, confidence interval; CRP= C reactive protein; SSB=sugar-sweetened beverage; T1= Test 1; T2= Test 2

Supplemental Figure S27: Sensitivity analysis of the systematic removal of each trial for the effect of mixed sources (without SSBs) on CRP (mg/L) in substitution trials

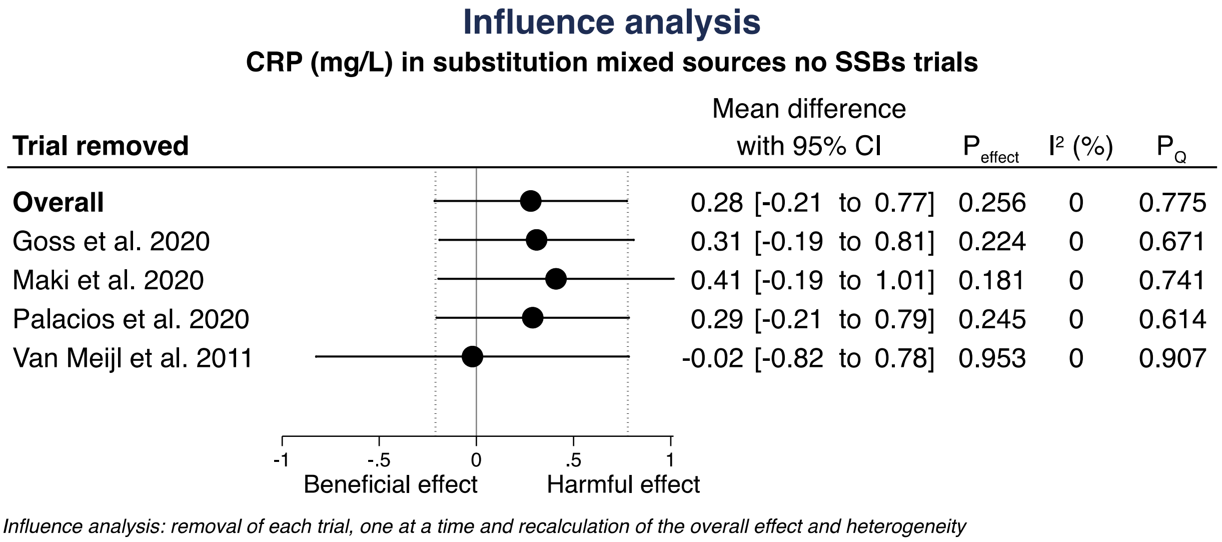

CI, confidence interval; CRP= C reactive protein; SSB= sugar sweetened beverage

Supplemental Figure S28: Sensitivity analysis of the systematic removal of each trial for the effect of SSB on CRP (mg/L) in addition trials

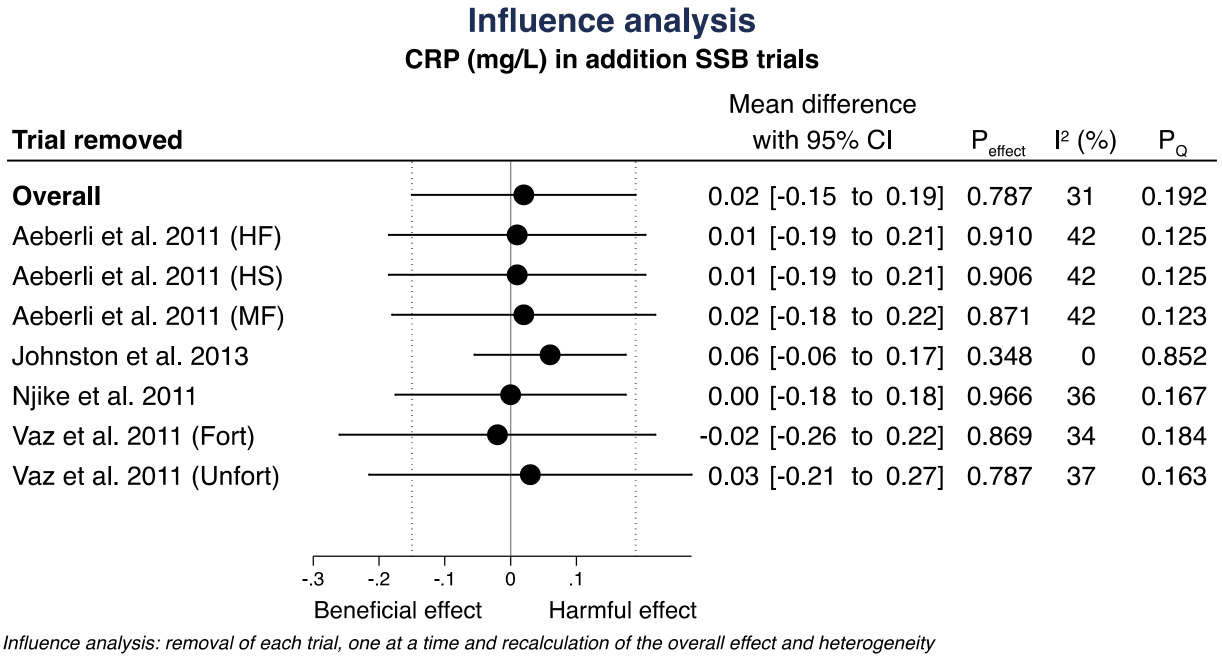

CI, confidence interval; CRP= C reactive protein; fort= fortified; HS=high sucrose; HF=high fructose; MF= medium fructose; SSB=sugar-sweetened beverage; T1= test 1; T2= test 2; unfort= unfortified

Supplemental Figure S29: Sensitivity analysis of the systematic removal of each trial for the effect of sweetened dairy alternatives (soy) on CRP (mg/L) in addition trials

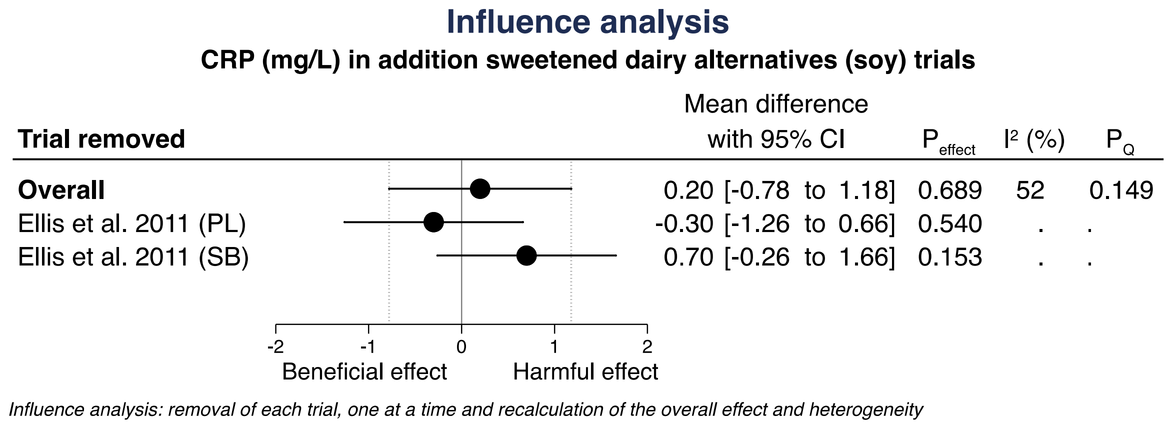

CI, confidence interval; CRP= C reactive protein; PL= Placebo; SB= Strawberries

Supplemental Figure S30: Sensitivity analysis of the systematic removal of each trial for the effect of 100% fruit juice on CRP (mg/L) in addition trials

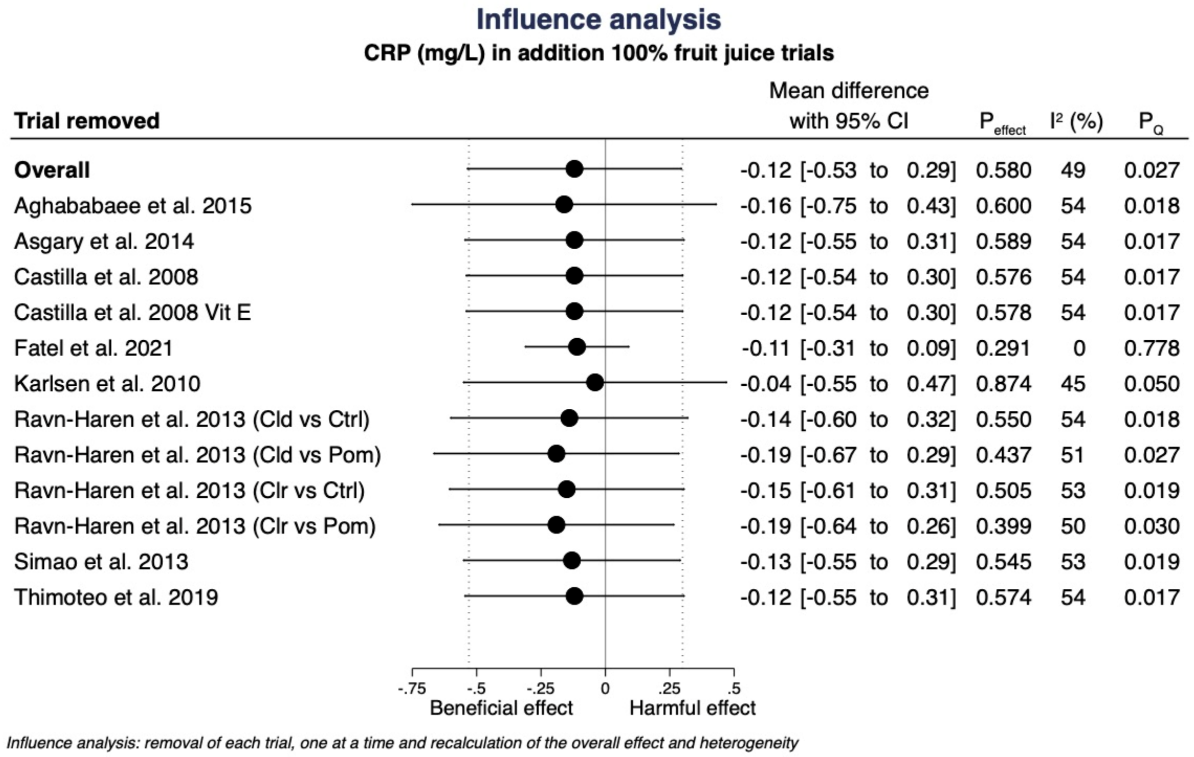

CI, confidence interval; CRP= C reactive protein; Cld=Cloudy; Ctrl=Control; Clr=Clear; Pom=Pomace; Vit E= Vitamin E

Supplemental Figure S31: Sensitivity analysis of the systematic removal of each trial for the effect of fruit on CRP (mg/L) in addition trials

Influence analysis

CRP (mg/L) in addition fruit trials

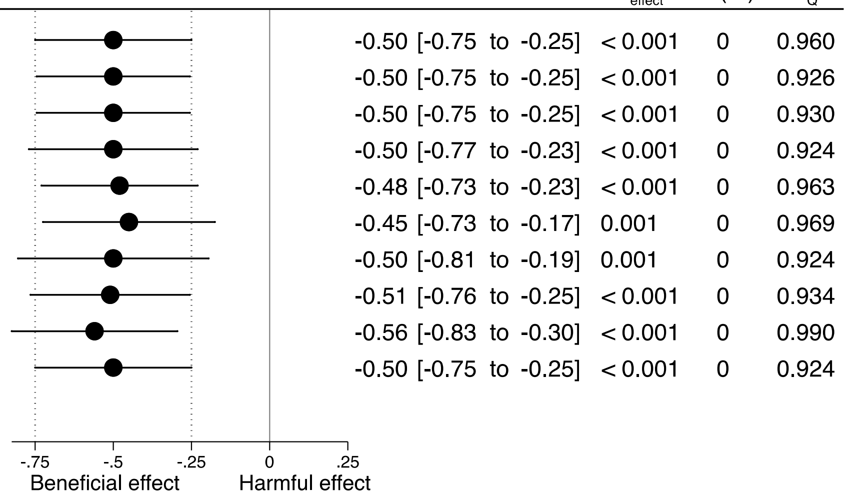

Trial removed	Mean difference with 95% CI	P_{effect}	I^2 (%)	P_Q
**Overall**	-0.50 [-0.75 to -0.25]	< 0.001	0	0.960
Basu et al. 2010	-0.50 [-0.75 to -0.25]	< 0.001	0	0.926
Blum et al. 2007	-0.50 [-0.75 to -0.25]	< 0.001	0	0.930
Dow et al. 2013	-0.50 [-0.77 to -0.23]	< 0.001	0	0.924
Franck et al. 2020	-0.48 [-0.73 to -0.23]	< 0.001	0	0.963
Kelley et al. 2013	-0.45 [-0.73 to -0.17]	0.001	0	0.969
Liddle et al. 2021	-0.50 [-0.81 to -0.19]	0.001	0	0.924
Ravn-Haren et al. 2013 (Clr)	-0.51 [-0.76 to -0.25]	< 0.001	0	0.934
Ravn-Haren et al. 2013 (Pom)	-0.56 [-0.83 to -0.30]	< 0.001	0	0.990
Schell et al. 2019	-0.50 [-0.75 to -0.25]	< 0.001	0	0.924

Influence analysis: removal of each trial, one at a time and recalculation of the overall effect and heterogeneity

CI, confidence interval; CRP= C reactive protein; Clr=Clear; Pom=Pomace

Supplemental Figure S32: Sensitivity analysis of the systematic removal of each trial for the effect of dried fruit on CRP (mg/L) in addition trials

Influence analysis

CRP (mg/L) in addition dried fruit trials

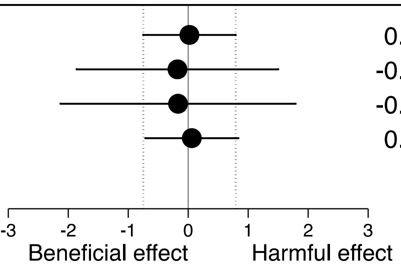

Trial removed	Mean difference with 95% CI	P_{effect}	I^2 (%)	P_Q
**Overall**	0.02 [-0.75 to 0.79]	0.962	0	0.575
Hooshmand et al. 2016 (HD)	-0.18 [-1.86 to 1.50]	0.836	6	0.303
Hooshmand et al. 2016 (LD)	-0.17 [-2.13 to 1.79]	0.866	10	0.293
Irannejad et al. 2020	0.06 [-0.71 to 0.83]	0.876	0	0.919

Influence analysis: removal of each trial, one at a time and recalculation of the overall effect and heterogeneity

CI, confidence interval; CRP= C reactive protein; HD= higher dose; LD= lower dose

Supplemental Figure S33: Sensitivity analysis of the systematic removal of each trial for the effect of sweets and desserts on CRP (mg/L) in addition trials

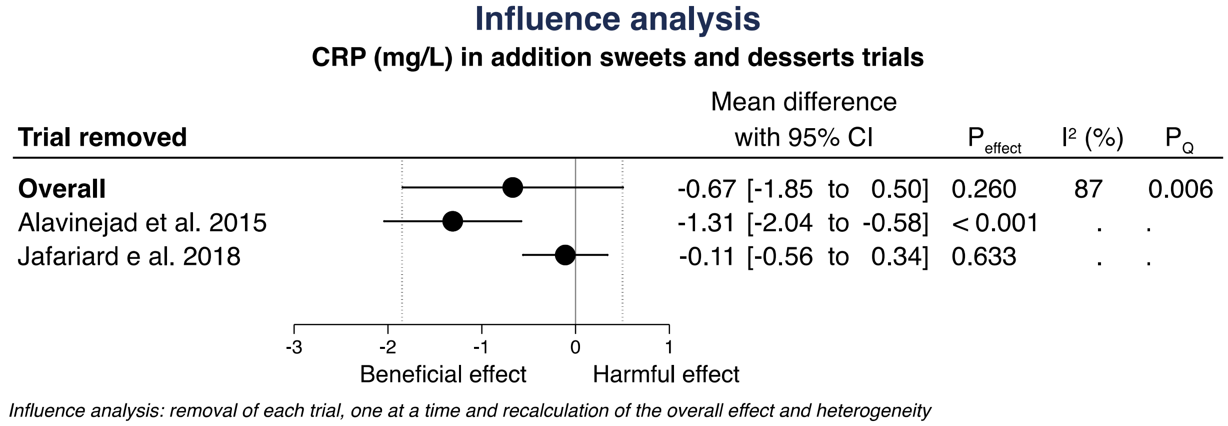

CI, confidence interval; CRP= C reactive protein

Supplemental Figure S34: Sensitivity analysis of the systematic removal of each trial for the effect of important food sources of fructose-containing sugars on TNF-α (pg/mL) in substitution trials

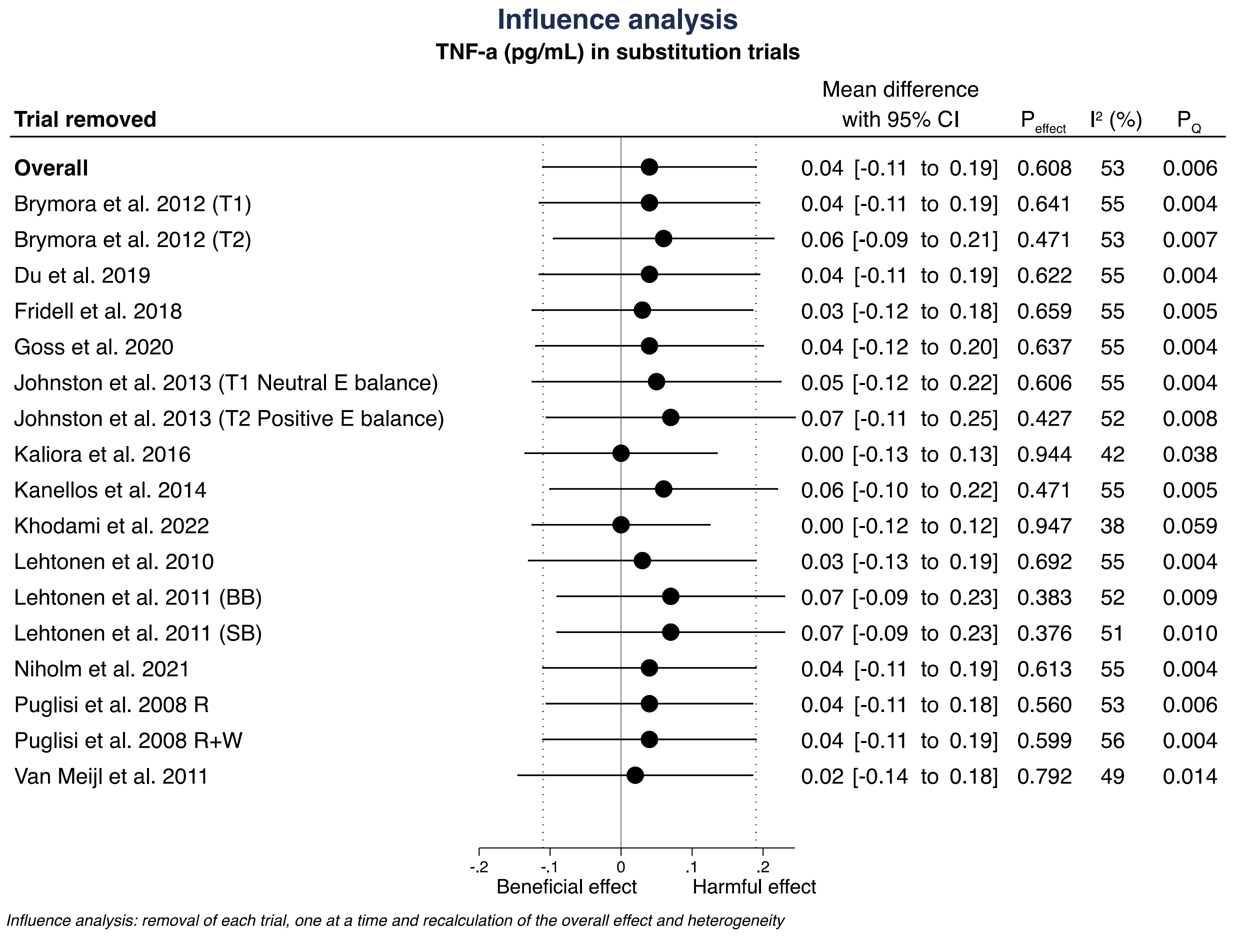

CI, confidence interval; BB= Bilberries; SB= sea buckthorn berries; R= Raisin; R+W= Raisin + Walk; T1= Test 1; T2= Test 2; TNF-a= tumour necrosis factor alpha

Supplemental Figure S35: Sensitivity analysis of the systematic removal of each trial for the effect of important food sources of fructose-containing sugars on TNF-α (pg/mL) in addition trials

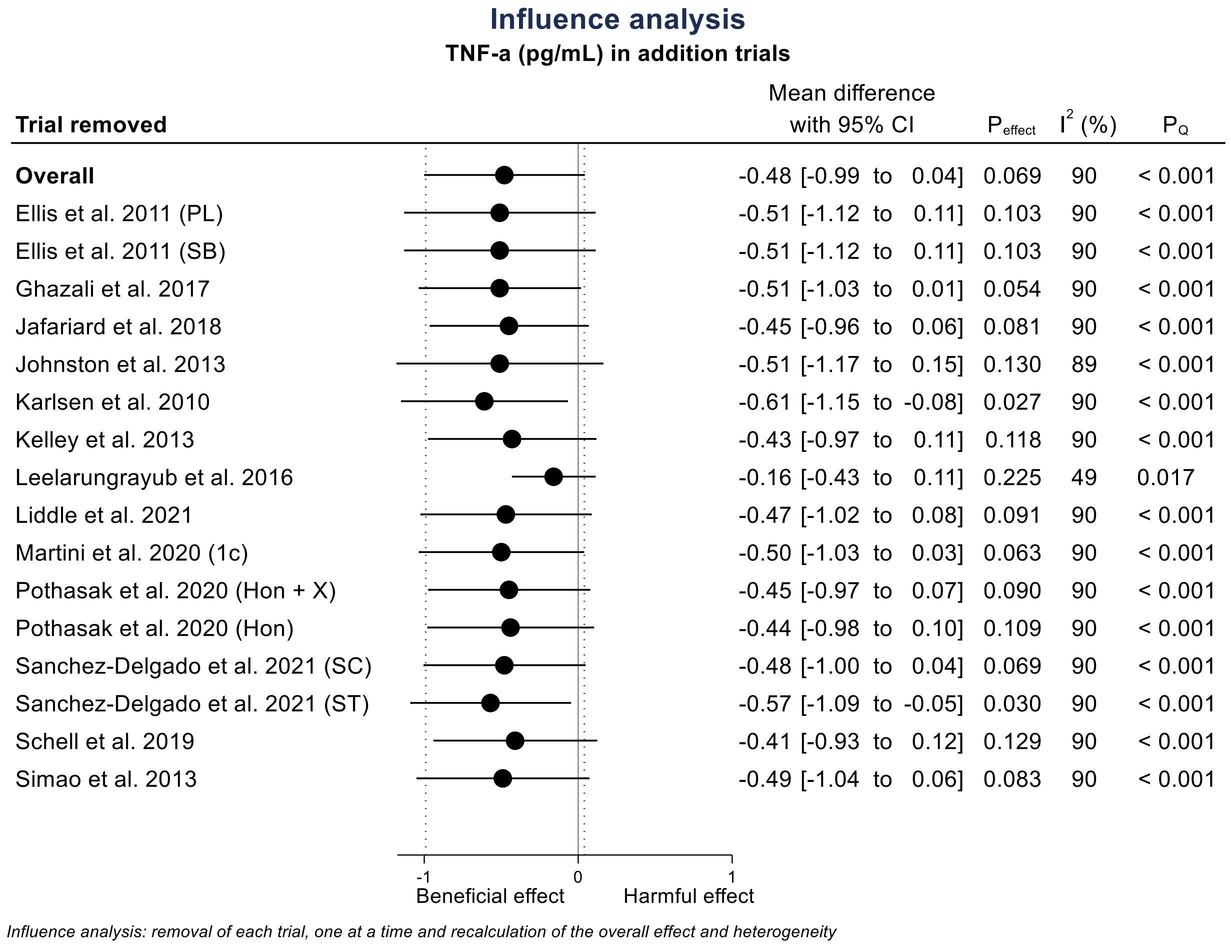

CI, confidence interval; Hon= Honey; Hon + X = Honey + exercise; PL= Placebo; SB= Strawberries; SC= sucralose; ST= steviol; TNF-a= Tumour necrosis factor-alpha; 1c= 1 cup

Supplemental Figure S36: Sensitivity analysis of the systematic removal of each trial for the effect of SSB on TNF-a (pg/mL) in addition trials

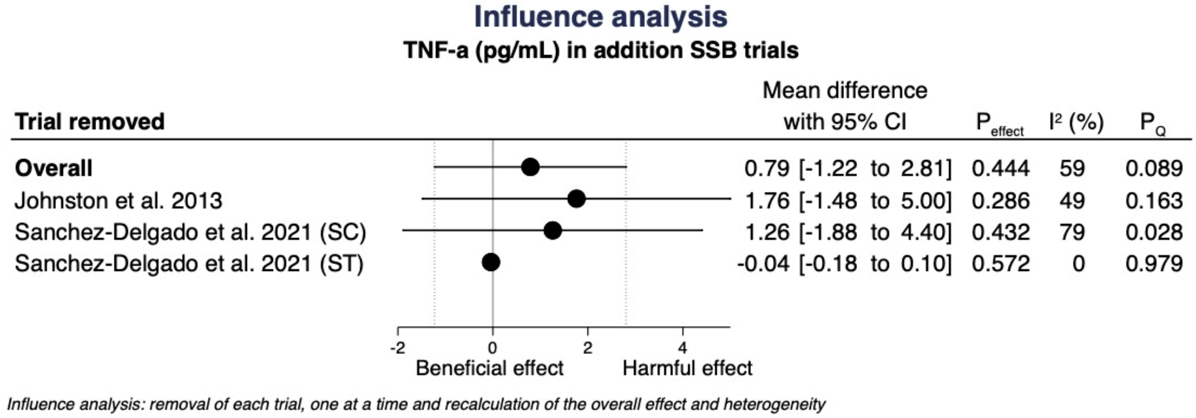

CI, confidence interval; TNF-a = tumour necrosis factor-alpha

Supplemental Figure S37: Sensitivity analysis of the systematic removal of each trial for the effect of sweetened dairy on TNF-a (pg/mL) in addition trials

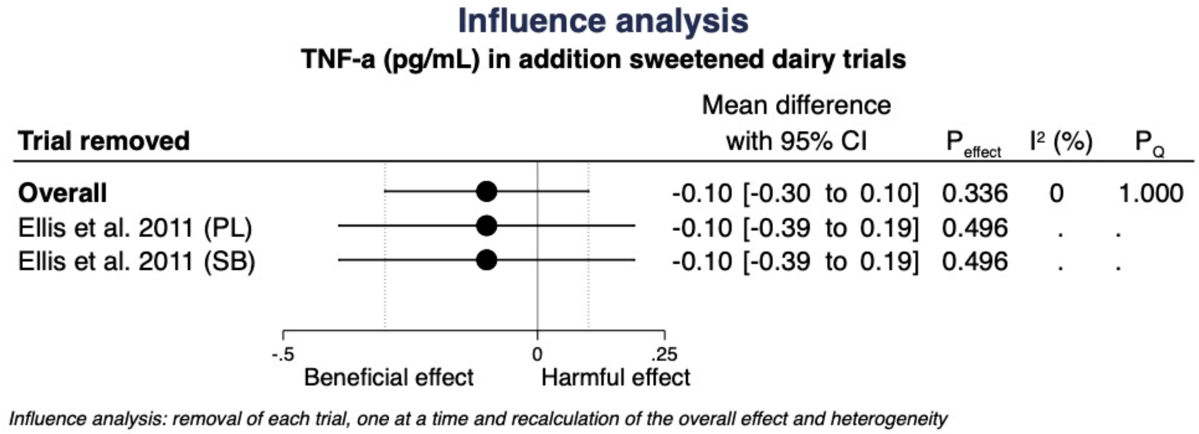

CI, confidence interval; TNF-a = tumour necrosis factor-alpha

Supplemental Figure S38: Sensitivity analysis of the systematic removal of each trial for the effect of 100% fruit juice on TNF-a (pg/mL) in addition trials

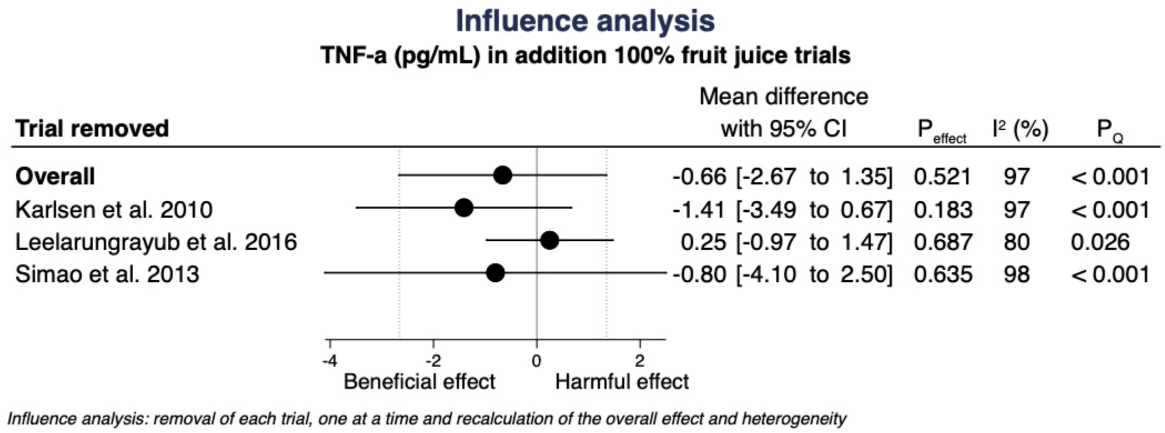

CI, confidence interval; TNF-a = tumour necrosis factor-alpha

Supplemental Figure S39: Sensitivity analysis of the systematic removal of each trial for the effect of fruit on TNF-a (pg/mL) in addition trials

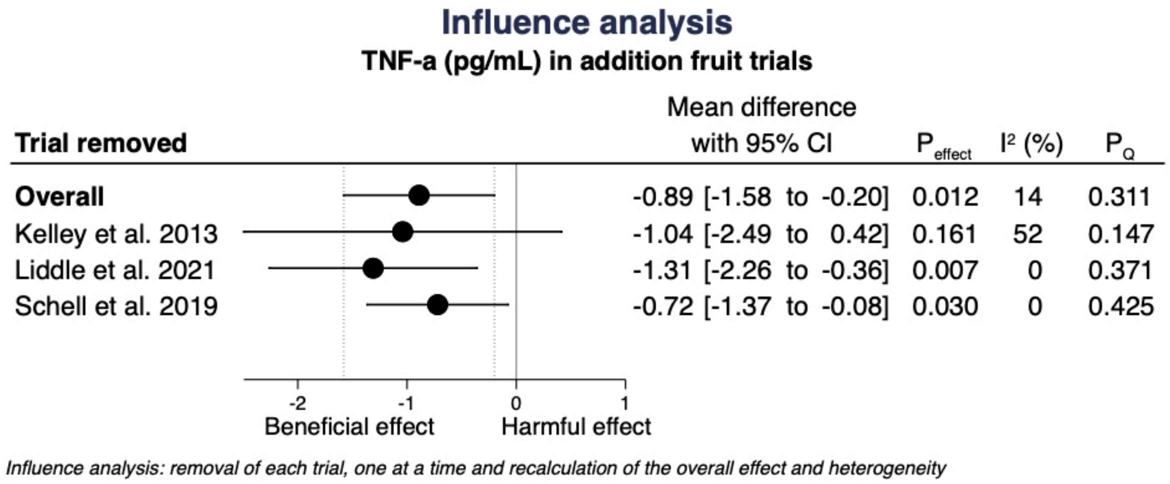

CI, confidence interval; TNF-a = tumour necrosis factor-alpha

Supplemental Figure S40: Sensitivity analysis of the systematic removal of each trial for the effect of addition sweets and desserts on TNF-a (pg/mL) in addition trials

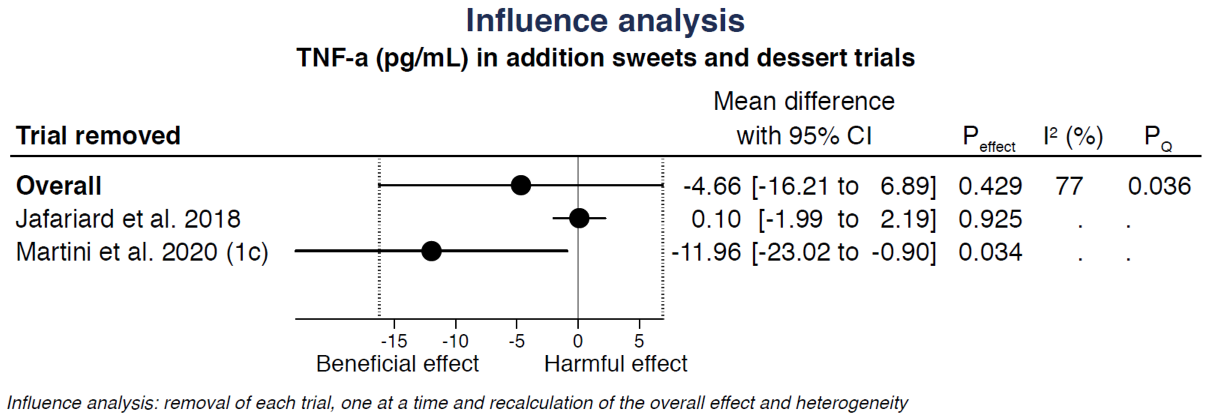

CI, confidence interval; TNF-a = tumour necrosis factor-alpha; 1c = 1 cup

Supplemental Figure S41: Sensitivity analysis of the systematic removal of each trial for the effect of added nutritive (caloric) sweeteners on TNF-a (pg/mL) in addition trials

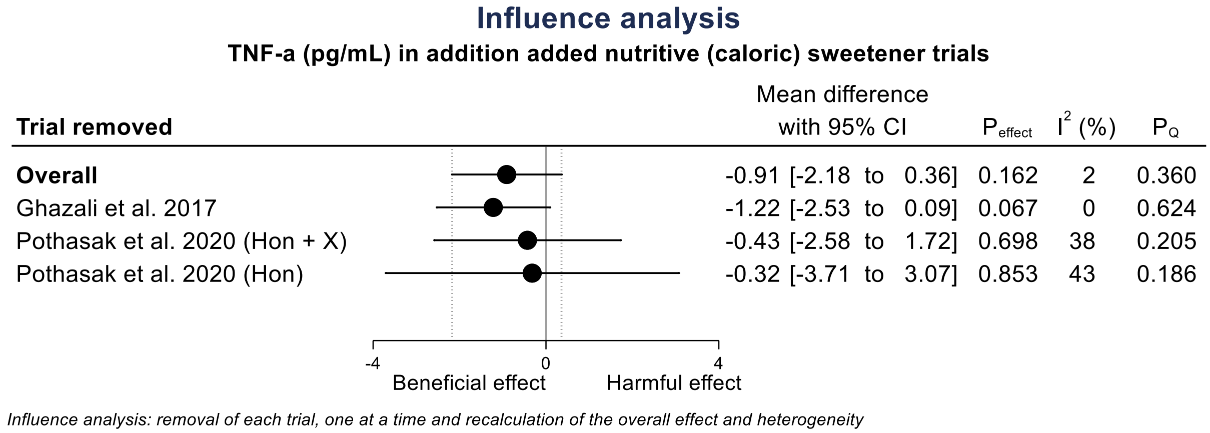

CI, confidence interval; TNF-a = tumour necrosis factor-alpha

Supplemental Figure S42: Sensitivity analysis of the systematic removal of each trial for the effect of important food sources of fructose-containing sugars on IL-6 (pg/mL) in substitution trials.

Influence analysis

IL-6 (pg/mL) in substitution trials

Trial removed	Mean difference with 95% CI	P_{effect}	I^2 (%)	P_Q
**Overall**	-0.04 [-0.23 to 0.16]	0.663	22	0.202
Cox et al. 2011	-0.03 [-0.23 to 0.17]	0.750	25	0.182
Du et al. 2019	-0.04 [-0.24 to 0.16]	0.682	26	0.164
Fridell et al. 2018	-0.05 [-0.24 to 0.14]	0.610	22	0.212
Goss et al. 2020	-0.09 [-0.28 to 0.09]	0.331	16	0.279
Johnston et al. 2013 (T1 Neutral E balance)	-0.04 [-0.24 to 0.16]	0.708	27	0.160
Johnston et al. 2013 (T2 Positive E balance)	-0.04 [-0.23 to 0.16]	0.658	24	0.186
Kaliora et al. 2016	0.02 [-0.18 to 0.22]	0.878	13	0.309
Kanellos et al. 2014	-0.09 [-0.28 to 0.10]	0.364	16	0.275
Kolehmainen et al. 2012	0.01 [-0.20 to 0.22]	0.922	18	0.250
Kuzma et al. 2016 (Frc)	-0.03 [-0.25 to 0.19]	0.795	27	0.155
Kuzma et al. 2016 (HFCS)	-0.02 [-0.23 to 0.19]	0.832	27	0.155
Lehtonen et al. 2011 (BB)	-0.07 [-0.25 to 0.11]	0.432	14	0.295
Lehtonen et al. 2011 (SB)	-0.07 [-0.25 to 0.11]	0.425	14	0.300
Niholm et al. 2021	-0.04 [-0.27 to 0.19]	0.738	25	0.180
Palacios et al. 2020	-0.04 [-0.24 to 0.16]	0.726	27	0.157
Van Meijl et al. 2011	-0.04 [-0.26 to 0.18]	0.707	26	0.167

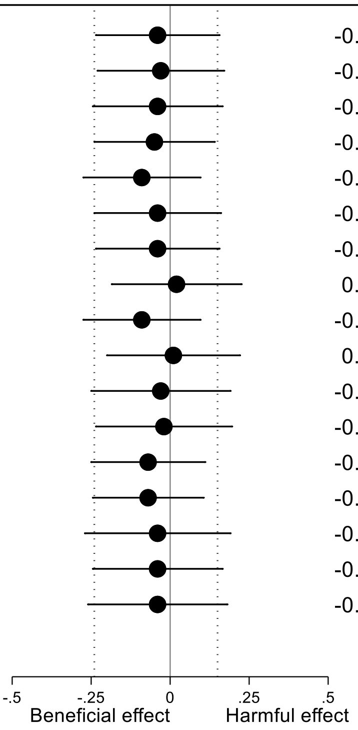

Influence analysis: removal of each trial, one at a time and recalculation of the overall effect and heterogeneity

CI=confidence interval; IL-6= Interleukin-6; Frc= fructose; HFCS= High fructose corn syrup; BB= Bilberries; SB= Sea buckthorn berries; T1= Test 1; T2= Test 2

Supplemental Figure S43: Sensitivity analysis of the systematic removal of each trial for the effect of important food sources of fructose-containing sugars on IL-6 (pg/mL) on addition trials

Influence analysis

IL-6 (pg/mL) in addition trials

Trial removed	Mean difference with 95% CI	P_{effect}	I^2 (%)	P_Q
**Overall**	-0.15 [-0.45 to 0.15]	0.352	83	< 0.001
Asgary et al. 2014	-0.13 [-0.44 to 0.18]	0.413	84	< 0.001
Basu et al. 2010	-0.17 [-0.56 to 0.22]	0.400	74	< 0.001
Ellis et al. 2011 (PL)	-0.05 [-0.34 to 0.24]	0.730	68	< 0.001
Ellis et al. 2011 (SB)	-0.15 [-0.48 to 0.17]	0.344	84	< 0.001
Fatel et al. 2021	-0.13 [-0.43 to 0.17]	0.407	83	< 0.001
Ghazali et al. 2017	-0.20 [-0.52 to 0.12]	0.220	84	< 0.001
Jafariard e al. 2018	-0.12 [-0.41 to 0.17]	0.406	82	< 0.001
Johnston et al. 2013	-0.17 [-0.48 to 0.14]	0.280	84	< 0.001
Karlsen et al. 2010	-0.14 [-0.44 to 0.16]	0.365	83	< 0.001
Liddle et al. 2021	-0.11 [-0.47 to 0.25]	0.573	82	< 0.001
Sanchez-Delgado et al. 2021 (SC)	-0.17 [-0.48 to 0.14]	0.290	84	< 0.001
Sanchez-Delgado et al. 2021 (ST)	-0.17 [-0.48 to 0.14]	0.272	84	< 0.001
Schell et al. 2019	-0.13 [-0.43 to 0.17]	0.388	83	< 0.001
Simao et al. 2013	-0.17 [-0.49 to 0.15]	0.292	84	< 0.001
Zafrilla et al. 2021 (SC)	-0.18 [-0.52 to 0.16]	0.293	84	< 0.001
Zafrilla et al. 2021 (ST)	-0.13 [-0.47 to 0.22]	0.472	84	< 0.001

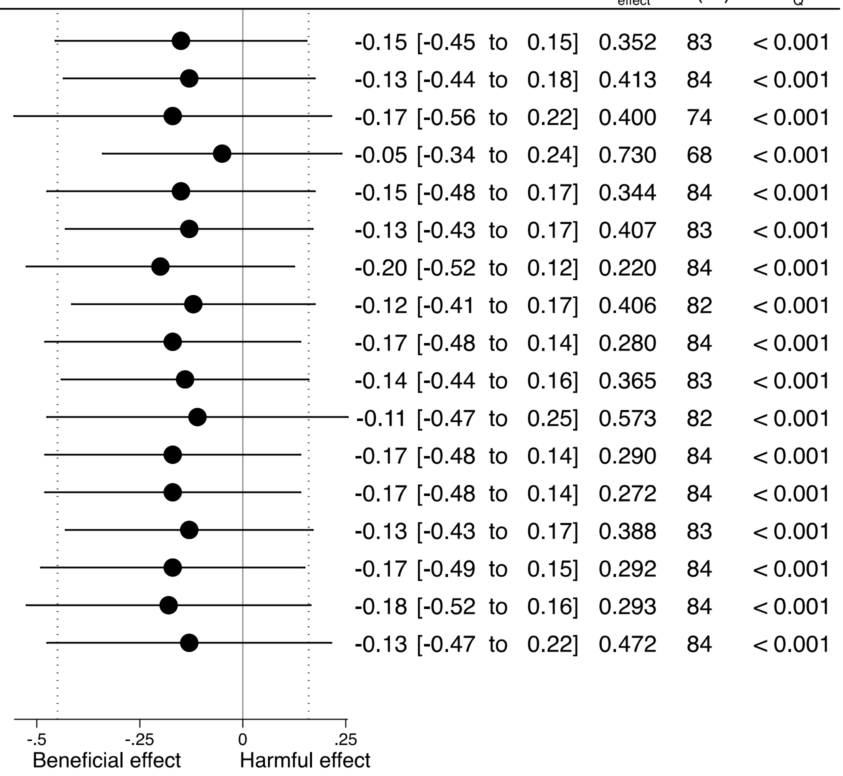

Influence analysis: removal of each trial, one at a time and recalculation of the overall effect and heterogeneity

CI=confidence interval; IL-6= Interleukin-6; PL= Placebo; SB= Strawberries; SC= sucralose; ST= steviol

Supplemental Figure S44: Sensitivity analysis of the systematic removal of each trial for the effect of SSB on IL-6 (pg/mL) in addition trials

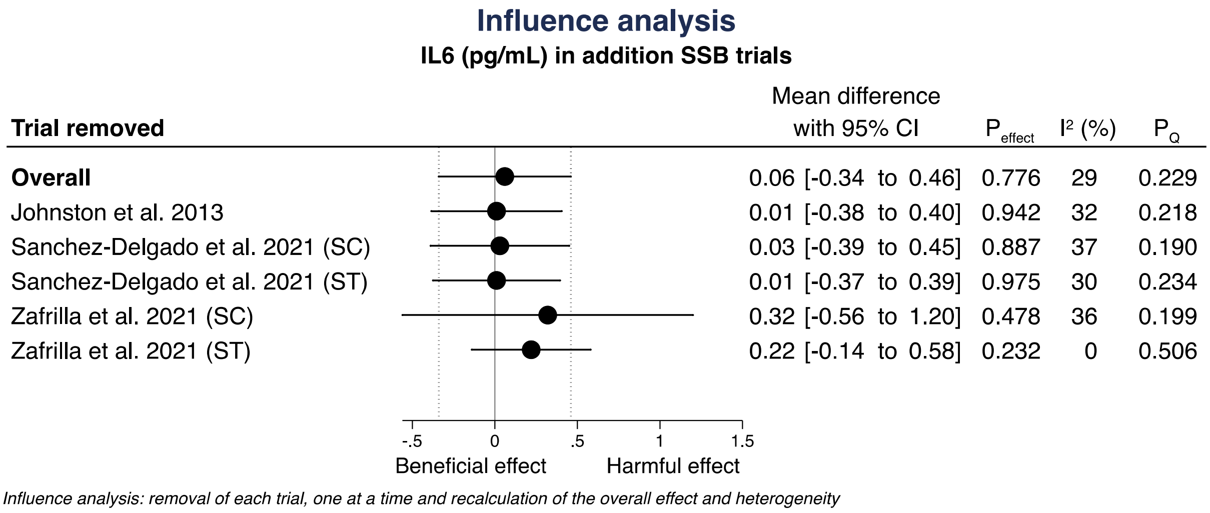

CI=confidence interval; IL6= interleukin-6; SC= sucralose; ST= steviol; SSB= sugar sweetened beverage

Supplemental Figure S45: Sensitivity analysis of the systematic removal of each trial for the effect of sweetened dairy on IL-6 (pg/mL) in addition trials

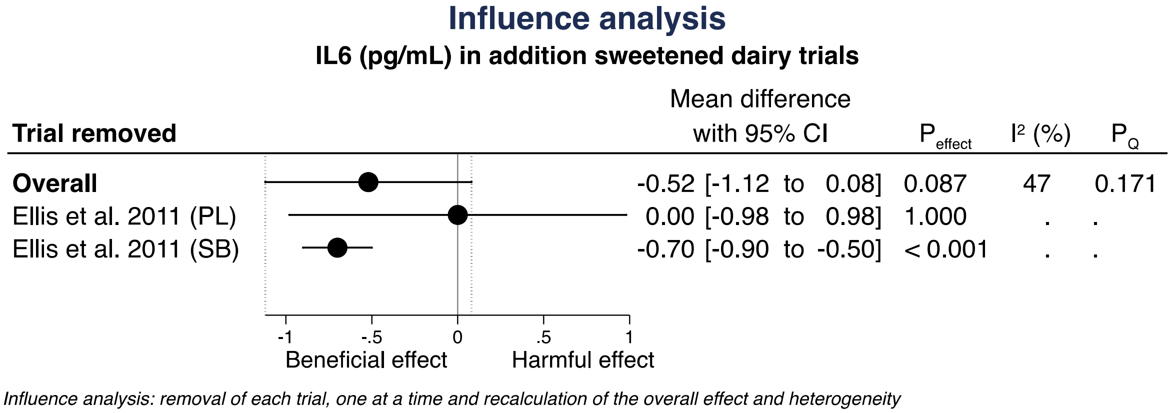

CI=confidence interval; IL6= interleukin-6; PL= placebo; SB= strawberries

Supplemental Figure S46: Sensitivity analysis of the systematic removal of each trial for the effect of 100% fruit juice on IL-6 (pg/mL) in addition trials

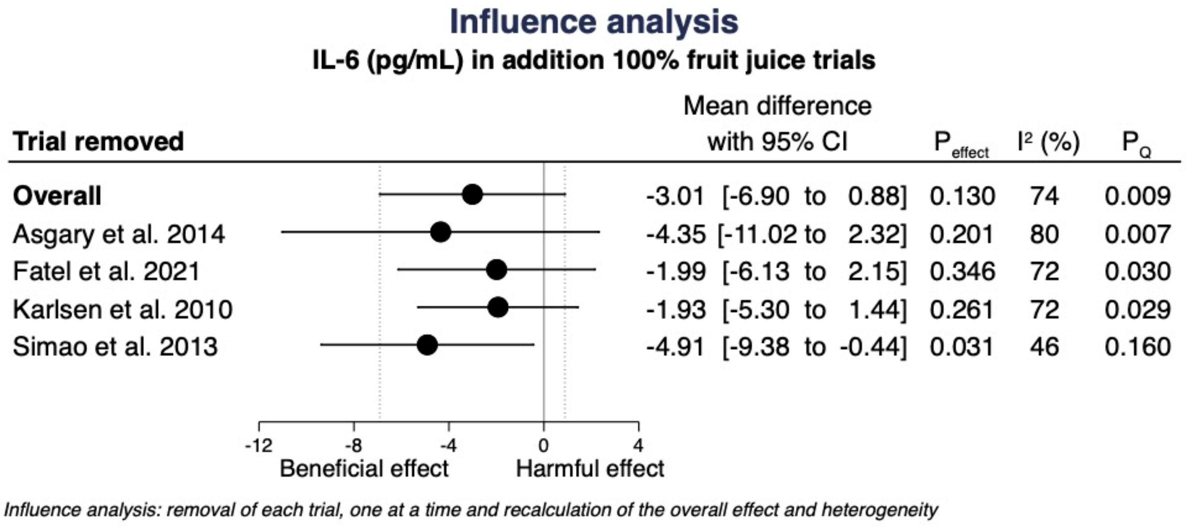

CI=confidence interval; IL-6= interleukin-6

Supplemental Figure S47: Sensitivity analysis of the systematic removal of each trial for the effect of fruit on IL-6 (pg/mL) in addition trials

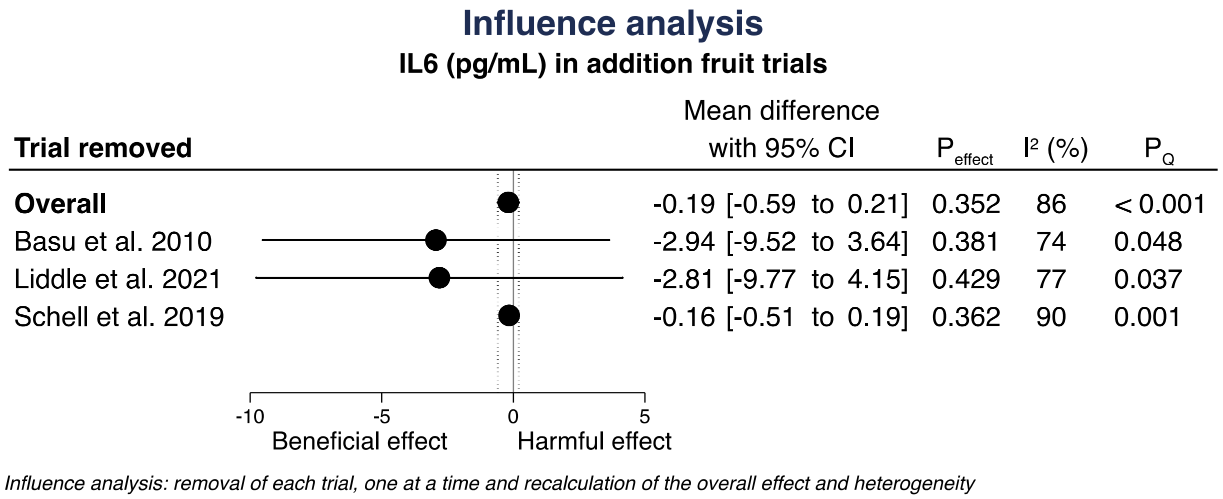

CI=confidence interval; IL6= interleukin 6

Supplemental Figure S48 (part 1 of 3): Subgroup analyses for the effect of important food sources of fructose-containing sugars on CRP (mg/L) in substitution trials

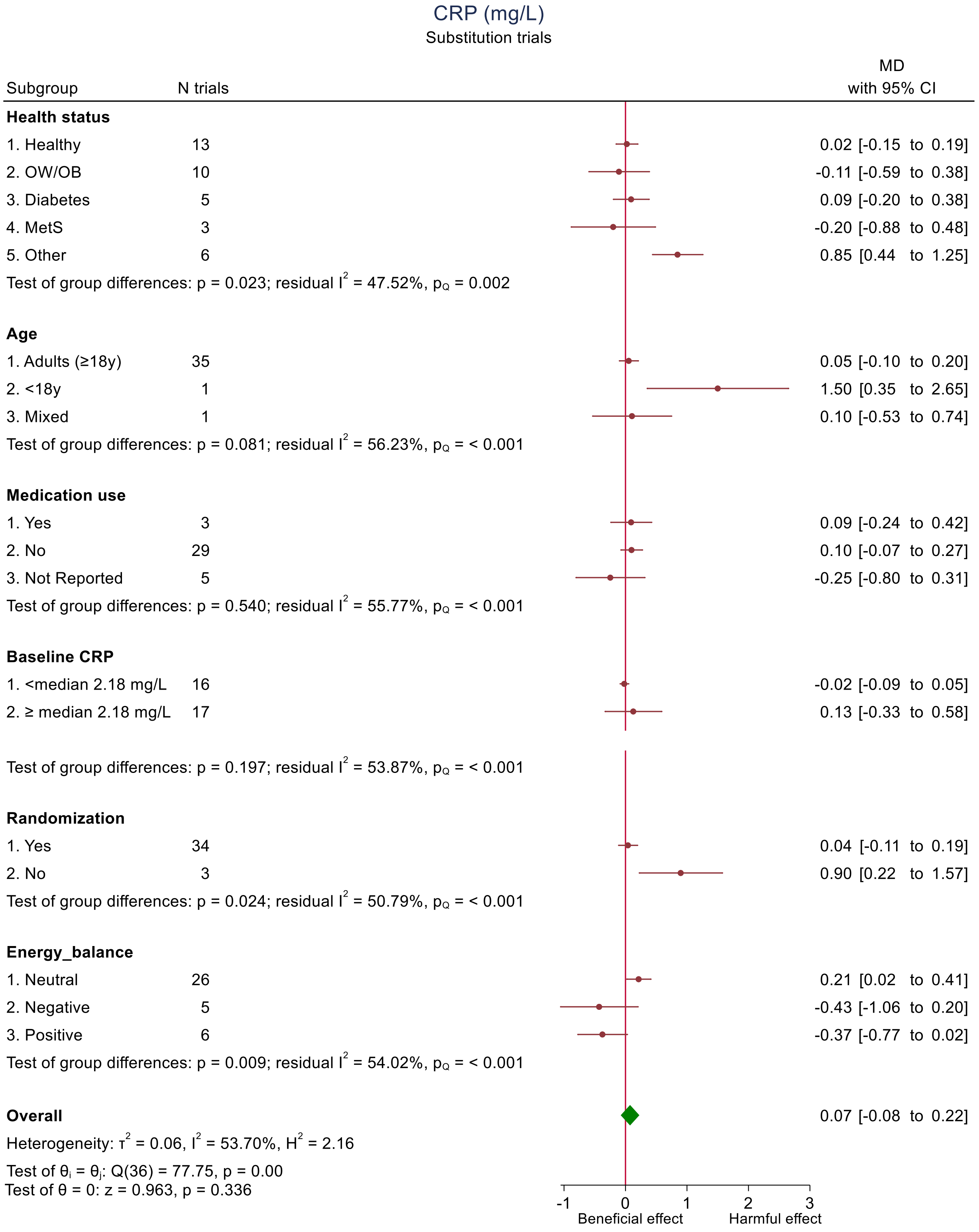

CI=confidence interval; CRP = C reactive protein ; het=heterogeneity; MD= mean difference; MetS= metabolic syndrome; OW/OB=overweight or obese; y=years

*N=4 trials missing data for baseline CRP

The green diamond represents the pooled estimate for the overall primary analysis of food sources of fructose-containing sugars and CRP. Within subgroup mean differences are the pooled effect estimates represented by a red circle. 95% confidence intervals are represented by the line through the circle. Data are expressed as mean differences with 95% confidence intervals using the generic inverse-variance method and random effects DerSimonian-Laird model. Inter-study heterogeneity was assessed using the Cochrane Q statistic and quantified using the I^2 statistic, with significance set at $P_Q<0{\cdot}100$ and $I^2\geq50\%$ considered to be evidence of substantial heterogeneity. $P<0{\cdot}050$ indicates that the effect size differed between levels of the subgroup.

Supplemental Figure S48 (part 2 of 3): Subgroup analyses for the effect of important food sources of fructose-containing sugars on CRP (mg/L) in substitution trials

CRP (mg/L)

Substitution trials

Subgroup	N trials	MD with 95% CI
**Fructose type**		
1. Fructose	8	0.14 [-0.19 to 0.47]
2. Sucrose	6	-0.04 [-0.35 to 0.26]
3. Honey	1	-0.03 [-0.11 to 0.05]
4. Fruit	12	-0.23 [-0.56 to 0.11]
5. HFCS	3	-0.03 [-0.48 to 0.42]
6. Mixed type	7	0.80 [0.52 to 1.07]
Test of group differences: p = 0.002; residual I^2 = 27.73%, p_Q = 0.076		
**Comparator**		
1. Starch	4	0.16 [-0.48 to 0.80]
2. Glucose	12	0.04 [-0.18 to 0.25]
3. Fat	4	-0.42 [-1.10 to 0.26]
4. Lactose	3	-0.10 [-0.90 to 0.69]
5. Protein	1	0.04 [-0.81 to 0.88]
6. Malto-dextrin	1	-0.39 [-1.66 to 0.88]
7. Mixed	12	0.17 [-0.17 to 0.50]
Test of group differences: p = 0.851; residual I^2 = 60.37%, p_Q = < 0.001		
**Design**		
1. Parallel	22	-0.01 [-0.31 to 0.28]
2. Crossover	15	-0.00 [-0.08 to 0.07]
Test of group differences: p = 0.498; residual I^2 = 54.43%, p_Q = < 0.001		
**Follow up**		
1. ≤ 8 weeks	25	0.06 [-0.14 to 0.25]
2. >8 weeks	12	0.10 [-0.23 to 0.42]
Test of group differences: p = 0.800; residual I^2 = 54.67%, p_Q = < 0.001		
**Feeding control**		
1. Supplemented	27	-0.02 [-0.17 to 0.13]
2. Dietary advice	7	0.50 [0.04 to 0.97]
3. Metabolic	2	-0.62 [-1.20 to -0.04]
4. Metabolic and Supplemented	1	0.70 [-0.69 to 2.09]
Test of group differences: p = 0.007; residual I^2 = 54.26%, p_Q = < 0.001		
**Overall**		0.07 [-0.08 to 0.22]
Heterogeneity: τ^2 = 0.06, I^2 = 53.70%, H^2 = 2.16		
Test of $\theta_i = \theta_j$: Q(36) = 77.75, p = 0.00		
Test of θ = 0: z = 0.963, p = 0.336		

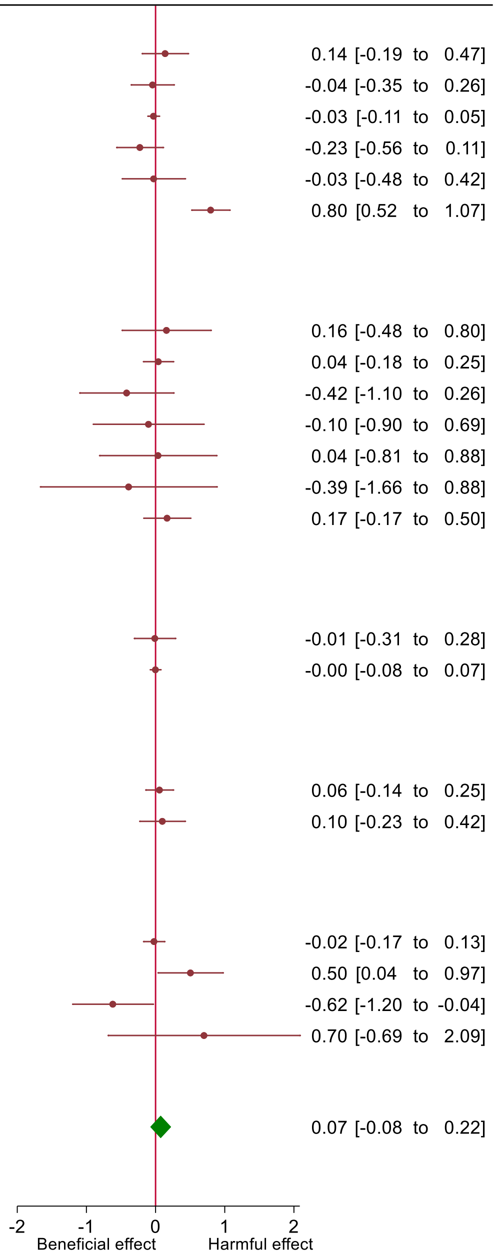

CI=confidence interval; CRP = C reactive protein; HFCS = High fructose corn syrup; MD= mean difference

The green diamond represents the pooled estimate for the overall primary analysis of food sources of fructose-containing sugars and CRP. Within subgroup mean differences are the pooled effect estimates represented by a red circle. 95% confidence intervals are represented by the line through the circle. Data are expressed as mean differences with 95% confidence intervals using the generic inverse-variance method and random effects DerSimonian-Laird model. Inter-study heterogeneity was assessed using the Cochrane Q statistic and quantified using the I^2 statistic, with significance set at $P_Q<0·100$ and $I^2≥50\%$ considered to be evidence of substantial heterogeneity. P<0·050 indicates that the effect size differed between levels of the subgroup.

Supplemental Figure S48 (part 3 of 3): Subgroup analyses for the effect of important food sources of fructose-containing sugars on CRP (mg/L) in substitution trials

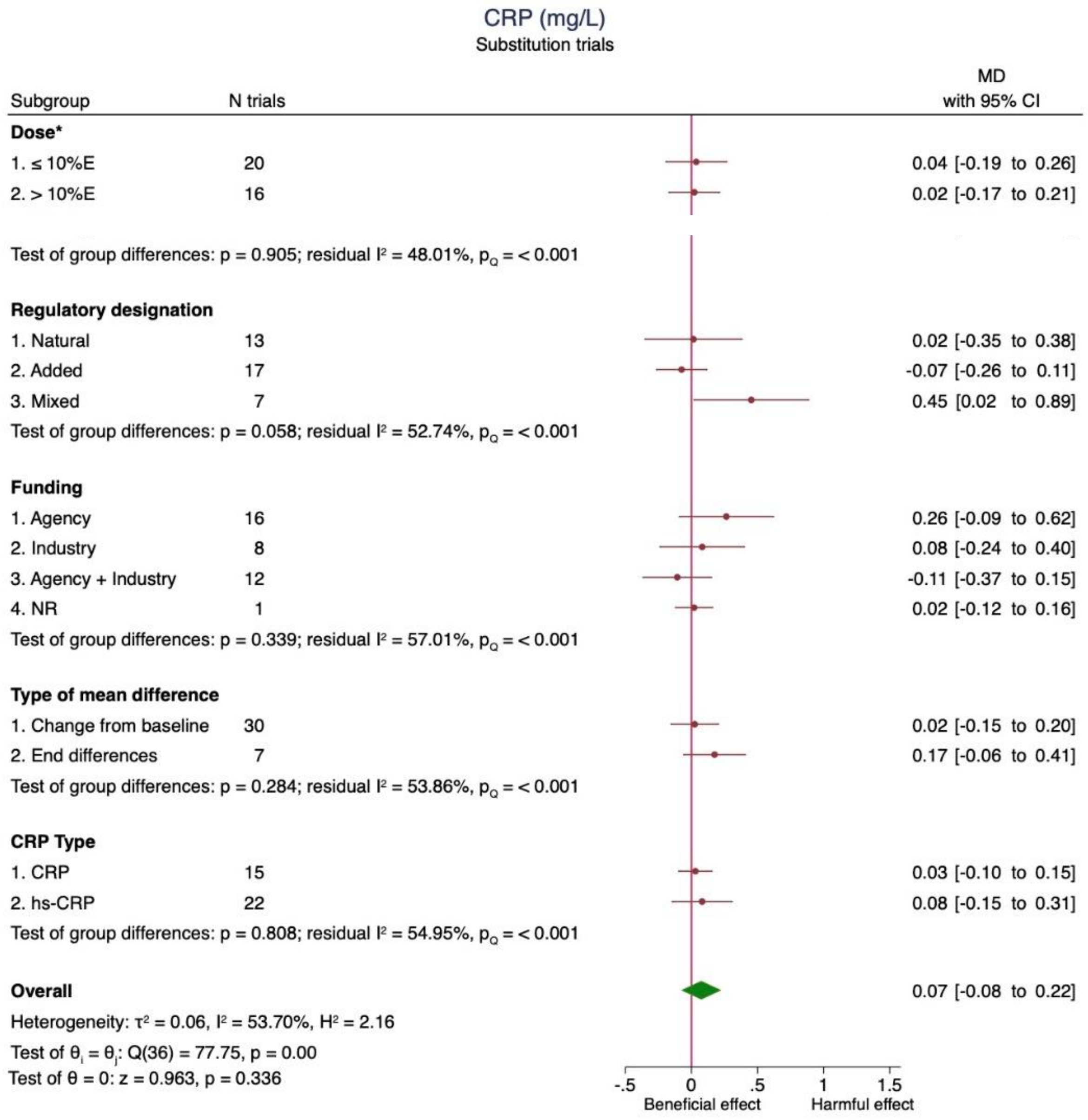

CI=confidence interval; CRP = C reactive protein; E= Energy; MD= mean difference; NR= not reported

The green diamond represents the pooled estimate for the overall primary analysis of food sources of fructose-containing sugars and CRP. Within subgroup mean differences are the pooled effect estimates represented by a red circle. 95% confidence intervals are represented by the line through the circle. Data are expressed as mean differences with 95% confidence intervals using the generic inverse-variance method and random effects DerSimonian-Laird model. Inter-study heterogeneity was assessed using the Cochrane Q statistic and quantified using the I^2 statistic, with significance set at $P_Q<0{\cdot}100$ and $I^2 \geq 50\%$ considered to be evidence of substantial heterogeneity. P<0·050 indicates that the effect size differed between levels of the subgroup.

Supplemental Figure S49: Risk of bias (using The Cochrane Collaboration Tool) subgroup analysis for the effect of important food sources of fructose-containing sugars on CRP (mg/L) in substitution comparisons

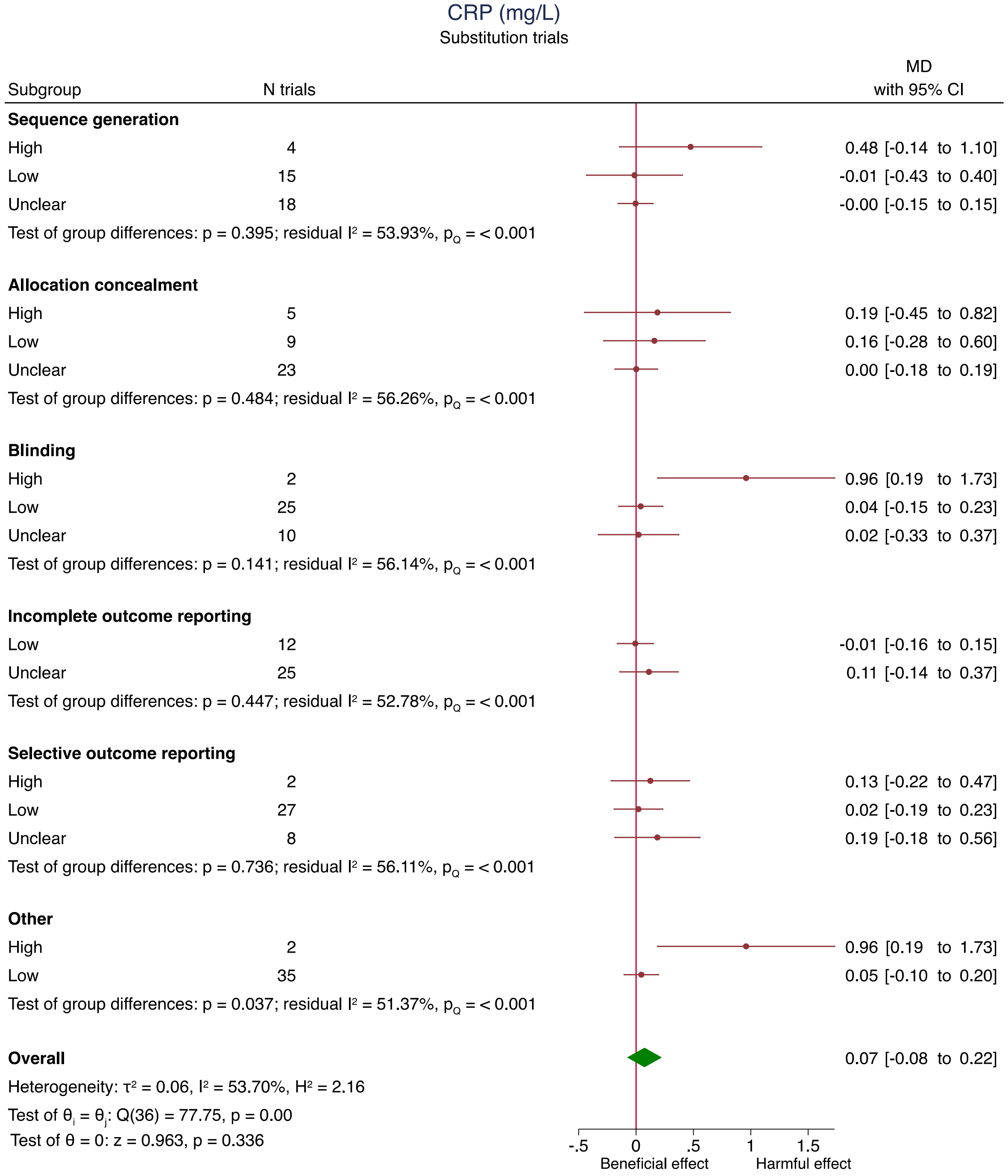

CI=confidence interval; CRP = C reactive protein; MD= mean difference
The green diamond represents the pooled estimate for the overall primary analysis of food sources of fructose-containing sugars and CRP. Within subgroup mean differences are the pooled effect estimates represented by a red circle. 95% confidence intervals are represented by the line through the circle. Data are expressed as mean differences with 95% confidence intervals using the generic inverse-variance method and random effects DerSimonian-Laird model. Inter-study heterogeneity was assessed using the Cochrane Q statistic and quantified using the I^2 statistic, with significance set at $P_Q<0{\cdot}100$ and $I^2\geq50\%$ considered to be evidence of substantial heterogeneity. P<0·050 indicates that the effect size differed between levels of the subgroup.

Supplemental Figure S50 (part 1 of 3): Subgroup analyses for the effect of important food sources of fructose-containing sugars on CRP (mg/L) in addition trials

CRP (mg/L)

Addition trials

Subgroup	N trials	MD with 95% CI
**Health status**		
1. Healthy	17	-0.08 [-0.21 to 0.05]
2. OW/OB	6	-0.28 [-0.80 to 0.23]
3. Diabetes	2	-1.33 [-2.06 to -0.61]
4. Mets	2	0.29 [-2.22 to 2.80]
5. Other	10	-0.23 [-0.75 to 0.29]
Test of group differences: p = 0.068; residual I^2 = 38.59%, p_Q = 0.013		
**Age**		
1. Adults (≥18y)	33	-0.22 [-0.40 to -0.05]
2. <18y	2	0.04 [-0.10 to 0.18]
		]
Test of group differences: p = 0.203; residual I^2 = 40.11%, p_Q = 0.009		
**Medication use**		
1. Yes	2	-0.17 [-3.00 to 2.66]
2. No	29	-0.17 [-0.35 to 0.01]
3. Not Reported	6	-0.21 [-0.53 to 0.10]
Test of group differences: p = 0.961; residual I^2 = 44.14%, p_Q = 0.003		
**Baseline CRP**		
1. <median 1.51 mg/L	17	-0.01 [-0.12 to 0.09]
2. ≥ median 1.51 mg/L	17	-0.41 [-0.77 to -0.06]
Test of group differences: p = 0.016; residual I^2 = 32.94%, p_Q = 0.036		
**Randomization**		
1. Yes	32	-0.13 [-0.29 to 0.03]
2. No	5	-0.49 [-0.83 to -0.16]
Test of group differences: p = 0.091; residual I^2 = 39.91%, p_Q = 0.008		
**Energy balance**		
2. Positive	37	-0.18 [-0.33 to -0.03]
Test of group differences: p = ; residual I^2 = %, p_Q =		
**Overall**		-0.18 [-0.33 to -0.03]
Heterogeneity: τ^2 = 0.06, I^2 = 43.67%, H^2 = 1.78		
Test of $\theta_i = \theta_j$: Q(36) = 63.90, p = 0.00		
Test of θ = 0: z = -2.324, p = 0.020		

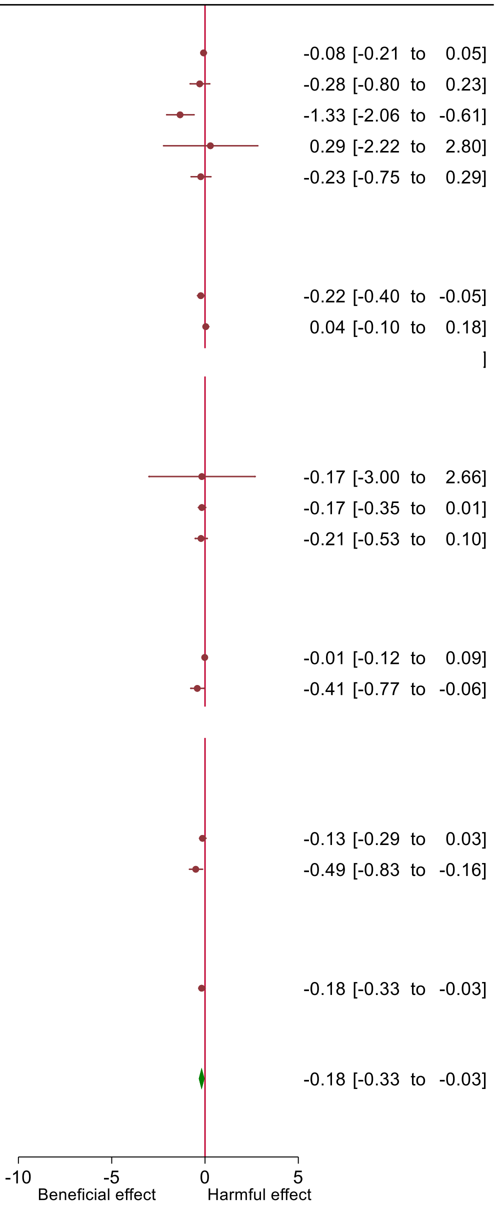

CI=confidence interval; CRP = C reactive protein; OW/OB=overweight or obese BMI; MD= mean difference
*N= 2 trials missing data for age in addition CRP trials
**N=4 trials missing data for baseline CRP
The green diamond represents the pooled estimate for the overall primary analysis of food sources of fructose-containing sugars and CRP. Within subgroup mean differences are the pooled effect estimates represented by a red circle. 95% confidence intervals are represented by the line through the circle. Data are expressed as mean differences with 95% confidence intervals using the generic inverse-variance method and random effects DerSimonian-Laird model. Inter-study heterogeneity was assessed using the Cochrane Q statistic and quantified using the I^2 statistic, with significance set at P_Q<0·100 and I^2≥50% considered to be evidence of substantial heterogeneity. P<0·050 indicates that the effect size differed between levels of the subgroup.

Supplemental Figure S50 (part 2 of 3): Subgroup analyses for the effect of important food sources of fructose-containing sugars on CRP (mg/L) in addition comparisons

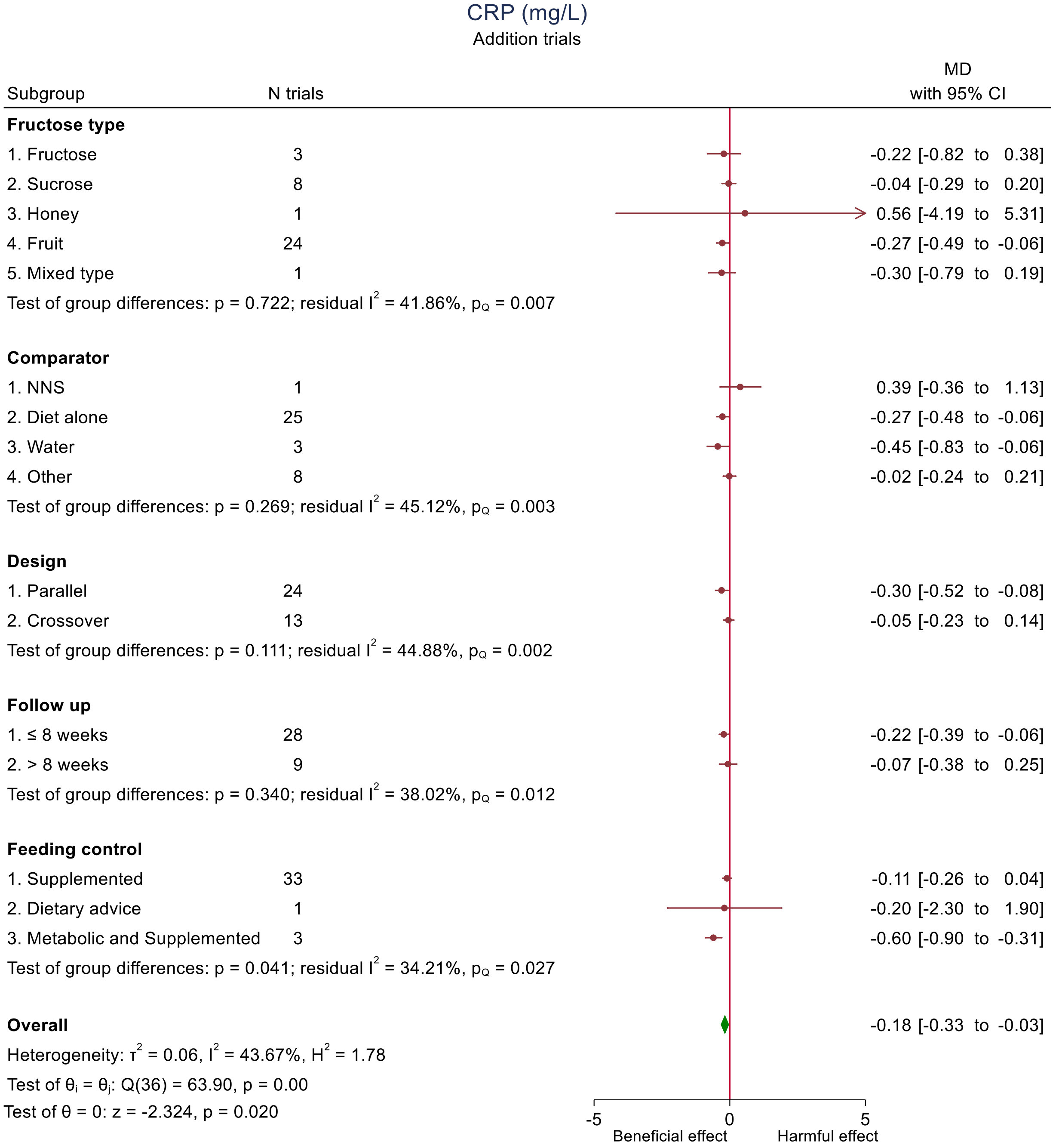

CI=confidence interval; CRP = C reactive protein ; MD= mean difference; NNS= non nutritive sweetener;
The green diamond represents the pooled estimate for the overall primary analysis of food sources of fructose-containing sugars and CRP. Within subgroup mean differences are the pooled effect estimates represented by a red circle. 95% confidence intervals are represented by the line through the circle. Data are expressed as mean differences with 95% confidence intervals using the generic inverse-variance method and random effects DerSimonian-Laird model. Inter-study heterogeneity was assessed using the Cochrane Q statistic and quantified using the I^2 statistic, with significance set at $P_Q<0{\cdot}100$ and $I^2 \geq 50\%$ considered to be evidence of substantial heterogeneity. $P<0{\cdot}050$ indicates that the effect size differed between levels of the subgroup.

Supplemental Figure S50 (part 3 of 3): Subgroup analyses for the effect of important food sources of fructose-containing sugars on CRP (mg/L) in addition comparisons

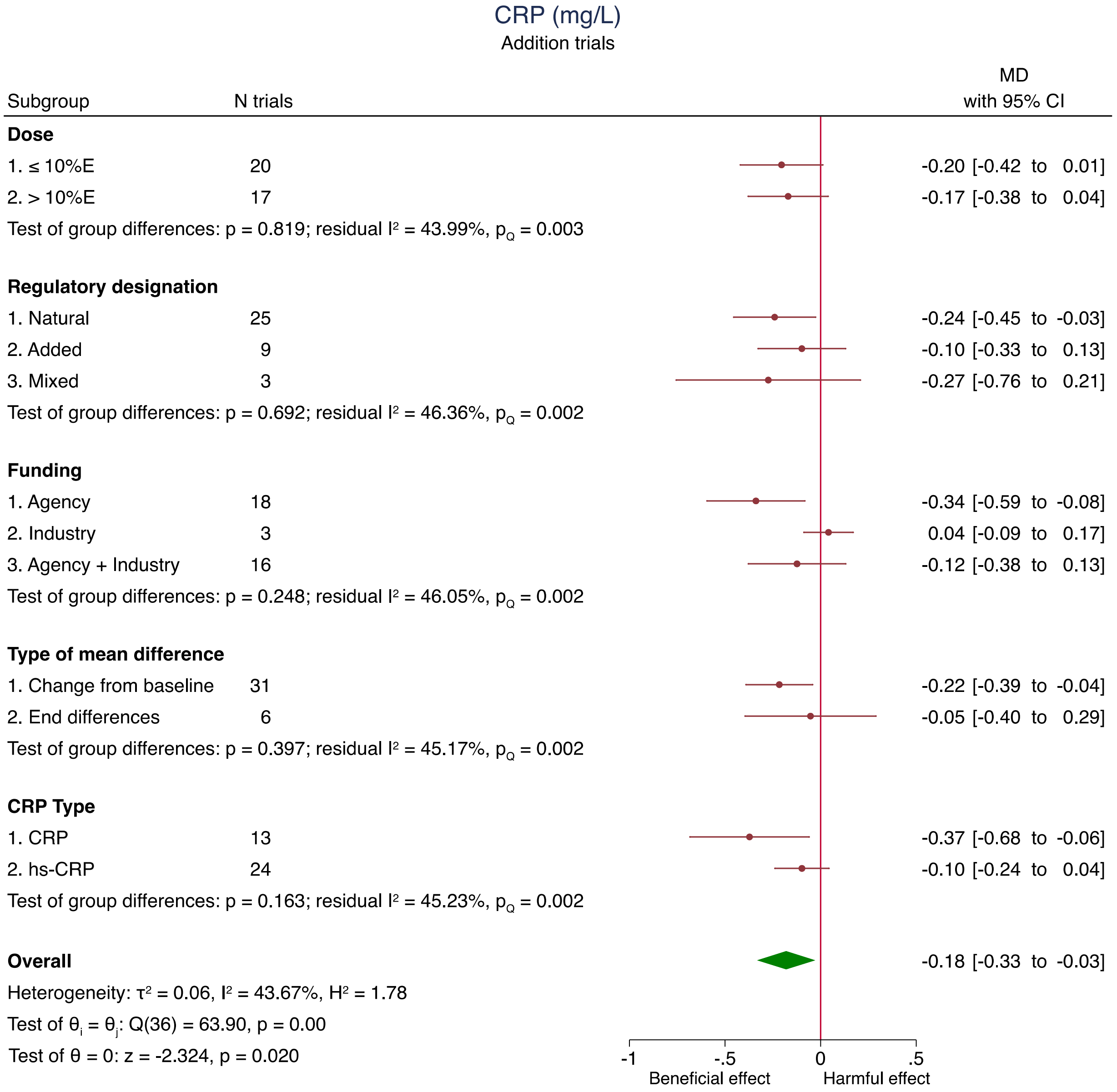

CI=confidence interval; CRP= C reactive protein; E= energy; MD= mean difference

The green diamond represents the pooled estimate for the overall primary analysis of food sources of fructose-containing sugars and CRP. Within subgroup mean differences are the pooled effect estimates represented by a red circle. 95% confidence intervals are represented by the line through the circle. Data are expressed as mean differences with 95% confidence intervals using the generic inverse-variance method and random effects DerSimonian-Laird model. Inter-study heterogeneity was assessed using the Cochrane Q statistic and quantified using the I^2 statistic, with significance set at P_Q<0·100 and I^2≥50% considered to be evidence of substantial heterogeneity. P<0·050 indicates that the effect size differed between levels of the subgroup.

Supplemental Figure S51: Risk of bias (using The Cochrane Collaboration Tool) subgroup analysis for the effect of important food sources of fructose-containing sugars on CRP (mg/L) in addition trials

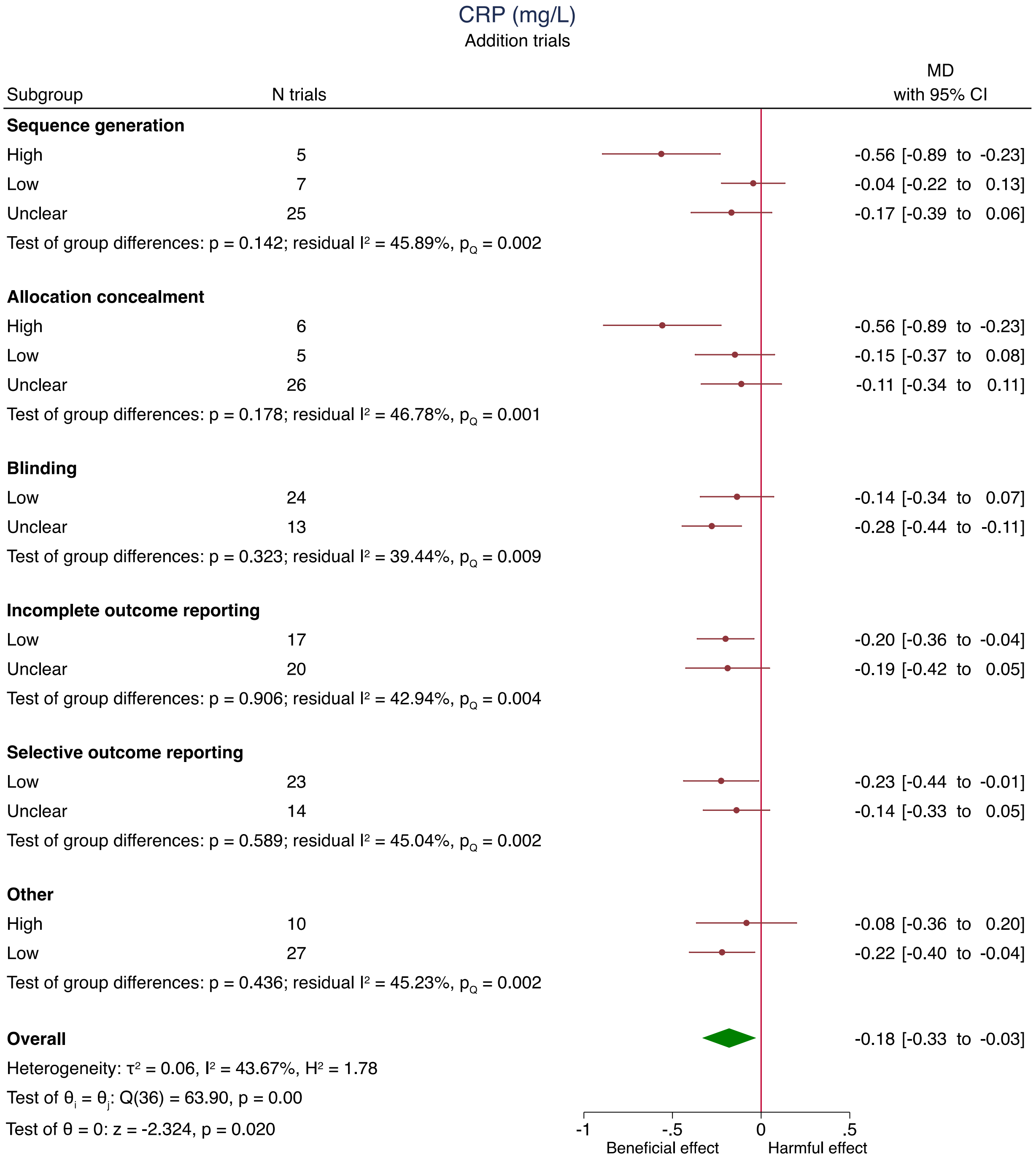

CI=confidence interval; CRP = C reactive protein; MD= mean difference

The green diamond represents the pooled estimate for the overall primary analysis of food sources of fructose-containing sugars and CRP. Within subgroup mean differences are the pooled effect estimates represented by a red circle. 95% confidence intervals are represented by the line through the circle. Data are expressed as mean differences with 95% confidence intervals using the generic inverse-variance method and random effects DerSimonian-Laird model. Inter-study heterogeneity was assessed using the Cochrane Q statistic and quantified using the I^2 statistic, with significance set at $P_Q<0{\cdot}100$ and $I^2 \geq 50\%$ considered to be evidence of substantial heterogeneity. P<0·050 indicates that the effect size differed between levels of the subgroup.

Supplemental Figure S52: Continuous meta-regression analysis for the effect of important food sources of fructose-containing sugars on CRP (mg/L) in substitution trials

Continuous meta-regression
in CRP (mg/L) substitution trials

Subgroup	Range	Trials	Beta [95% CI]	P_{MD}	Residual I^2 (%)	P_Q
Baseline CRP (mg/L)*	0.206—8.100	33	0.00 [-0.11 to 0.11]	0.719	51	0.001
Follow-up (weeks)	1.000—24.000	37	0.00 [-0.03 to 0.03]	0.701	48	0.001
Food source of fructose containing sugars dose (%E)	1.000—45.000	37	0.00 [-0.01 to 0.01]	0.971	48	0.001
Mean age (years)	13.500—70.212	37	0.00 [-0.01 to 0.01]	0.311	48	0.001

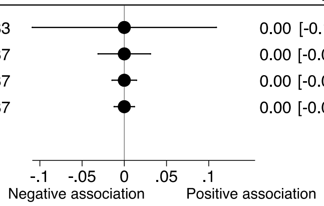

CI=confidence interval; CRP= C reactive protein; %E=percentage of total energy intake;
Data is presented as between group mean difference (95% confidence intervals) for a 1-unit change in the predictor variable. ß–coefficients were estimated using continuous meta-regression analysis. A positive ß-coefficient implies an increase in CRP with the food source of fructose-containing sugars intervention as the subgroup variable increases, and a negative ß-coefficient implies a decrease in uric acid. Residual I^2 reports inter-study heterogeneity not explained by the subgroup and was estimated using the Cochrane Q statistic.
*N=4 trials missing data for baseline CRP

Supplemental Figure S53: Continuous meta-regression analysis for the effect of important food sources of fructose-containing sugars on CRP (mg/L) in addition trials

Continuous meta-regression
in CRP (mg/L) addition trials

Subgroup	Range	Trials	Beta [95% CI]	P_{MD}	Residual I^2 (%)	P_Q
Baseline CRP (mg/L)*	0.206—55.474	35	0.00 [-0.07 to 0.07]	0.284	27	0.075
Follow-up (weeks)	1.000—24.000	37	0.00 [-0.02 to 0.02]	0.107	23	0.107
Food source of fructose containing sugars dose (%E)	1.100—35.000	37	0.00 [-0.01 to 0.01]	0.338	30	0.050
Mean age (years)**	8.250—70.700	36	0.00 [-0.01 to 0.01]	0.671	33	0.032

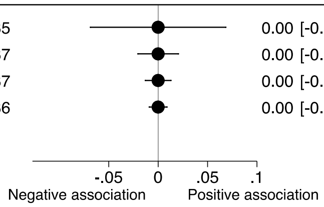

CI=confidence interval; CRP= C reactive protein; %E=percentage of total energy intake;
Data is presented as between group mean difference (95% confidence intervals) for a 1-unit change in the predictor variable. ß–coefficients were estimated using continuous meta-regression analysis. A positive ß-coefficient implies an increase in CRP with the food source of fructose-containing sugars intervention as the subgroup variable increases, and a negative ß-coefficient implies a decrease in uric acid. Residual I^2 reports inter-study heterogeneity not explained by the subgroup and was estimated using the Cochrane Q statistic.
* N=4 trials missing data for baseline CRP
**N=2 trials missing data for age in addition CRP trials

Supplemental Figure S54 (part 1 of 3): Subgroup analyses for the effect of SSB on CRP (mg/L) in substitution trials

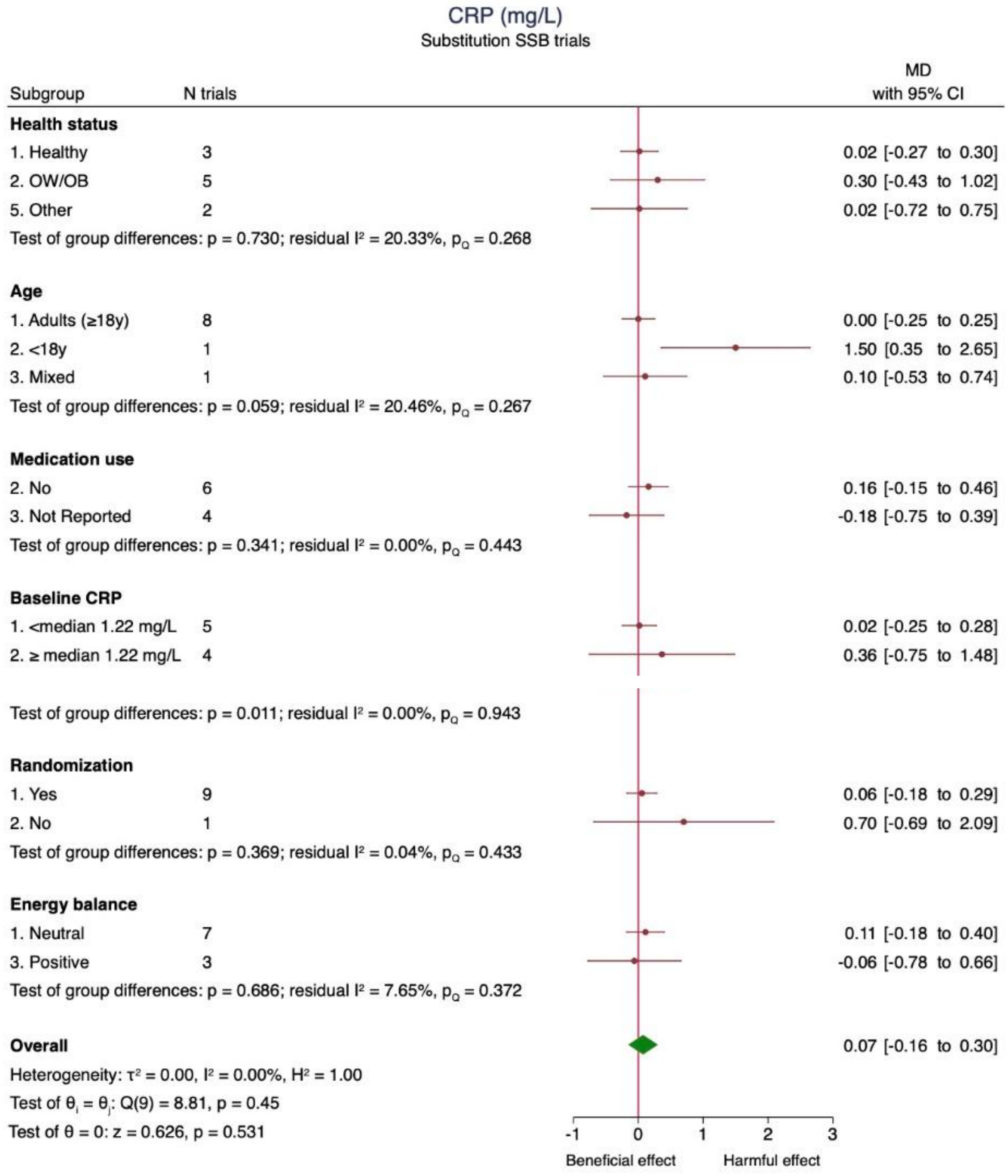

CI=confidence interval; CRP = C reactive protein; OW/OB=overweight or obese; MD= mean difference; y=years
*N=1 trials missing data for baseline CRP
The green diamond represents the pooled estimate for the overall primary analysis of food sources of fructose-containing sugars and CRP. Within subgroup mean differences are the pooled effect estimates represented by a red circle. 95% confidence intervals are represented by the line through the circle. Data are expressed as mean differences with 95% confidence intervals using the generic inverse-variance method and random effects DerSimonian-Laird model. Inter-study heterogeneity was assessed using the Cochrane Q statistic and quantified using the I^2 statistic, with significance set at $P_Q<0{\cdot}100$ and $I^2 \geq 50\%$ considered to be evidence of substantial heterogeneity. P<0·050 indicates that the effect size differed between levels of the subgroup.

Supplemental Figure S54 (part 2 of 3): Subgroup analyses for the effect of SSB on CRP (mg/L) in substitution trials

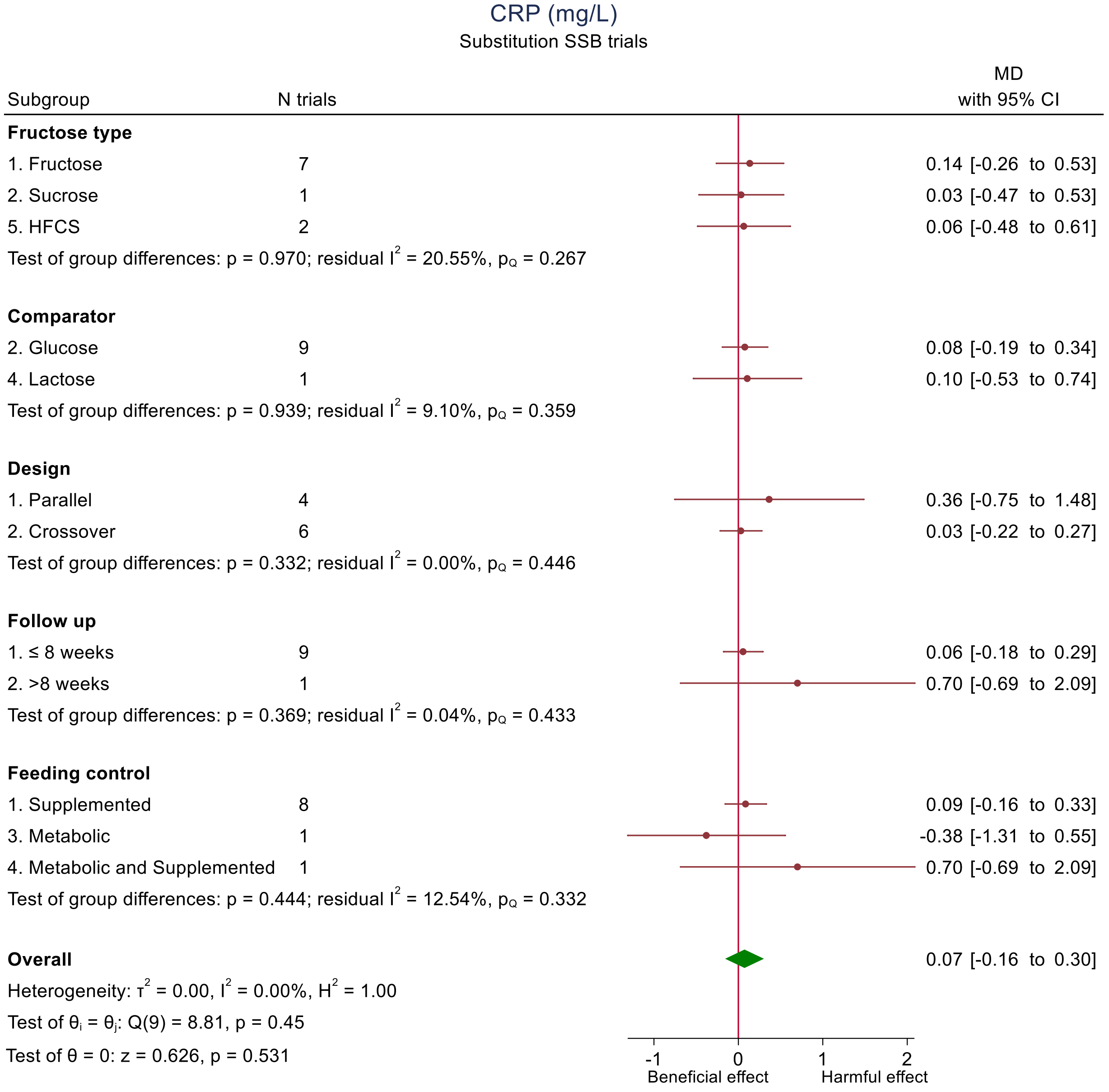

CI=confidence interval; CRP = C reactive protein ; HFCS = High fructose corn syrup; MD= mean difference
The green diamond represents the pooled estimate for the overall primary analysis of food sources of fructose-containing sugars and CRP. Within subgroup mean differences are the pooled effect estimates represented by a red circle. 95% confidence intervals are represented by the line through the circle. Data are expressed as mean differences with 95% confidence intervals using the generic inverse-variance method and random effects DerSimonian-Laird model. Inter-study heterogeneity was assessed using the Cochrane Q statistic and quantified using the I^2 statistic, with significance set at $P_Q<0·100$ and $I^2 \geq 50\%$ considered to be evidence of substantial heterogeneity. P<0·050 indicates that the effect size differed between levels of the subgroup.

Supplemental Figure S54 (part 3 of 3): Subgroup analyses for the effect of SSB on CRP (mg/L) in substitution trials

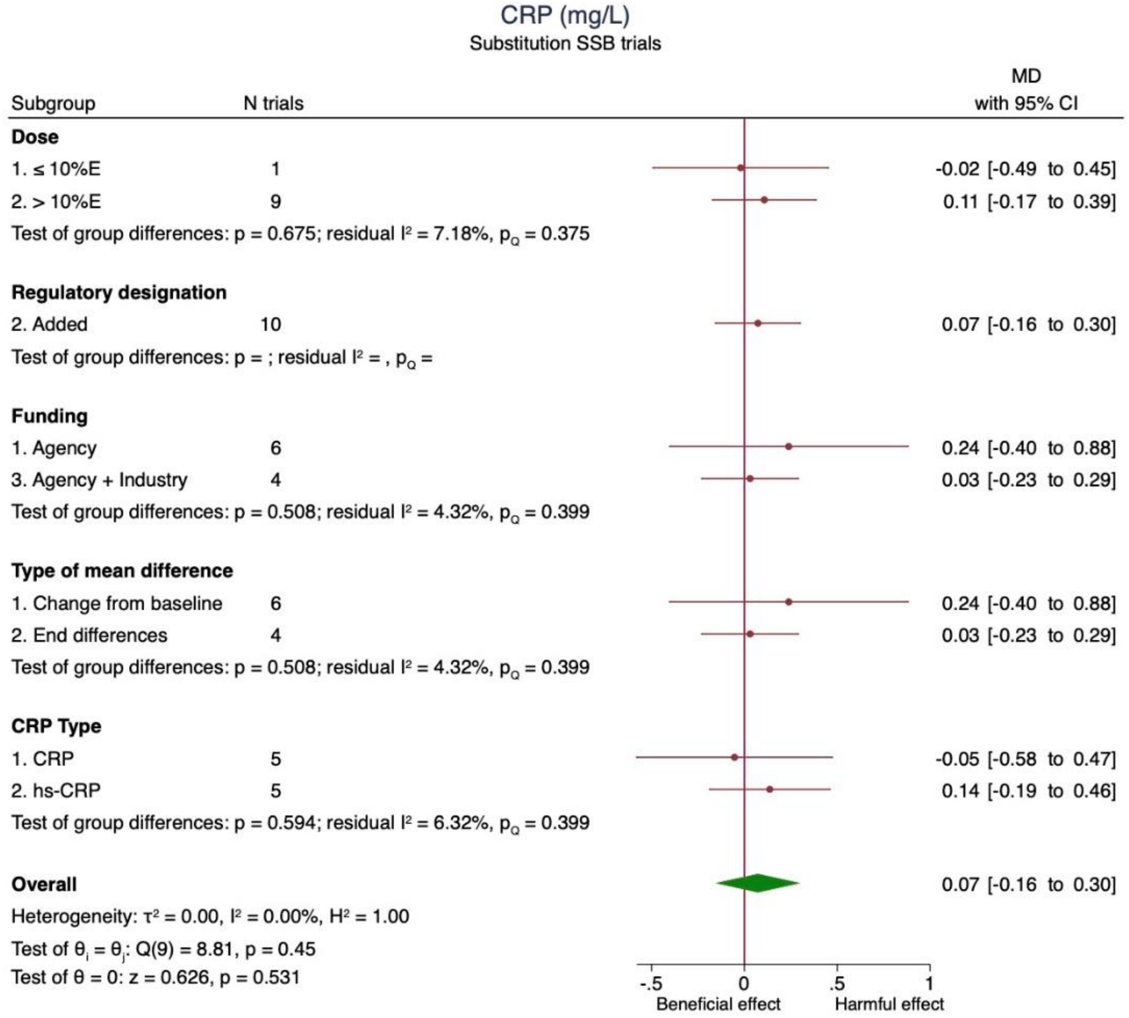

CI=confidence interval; CRP = C reactive protein ; E= energy; MD= mean difference
The green diamond represents the pooled estimate for the overall primary analysis of food sources of fructose-containing sugars and CRP. Within subgroup mean differences are the pooled effect estimates represented by a red circle. 95% confidence intervals are represented by the line through the circle. Data are expressed as mean differences with 95% confidence intervals using the generic inverse-variance method and random effects DerSimonian-Laird model. Inter-study heterogeneity was assessed using the Cochrane Q statistic and quantified using the I^2 statistic, with significance set at $P_Q<0{\cdot}100$ and $I^2\geq50\%$ considered to be evidence of substantial heterogeneity. P<0·050 indicates that the effect size differed between levels of the subgroup.

Supplemental Figure S55: Risk of bias (using The Cochrane Collaboration Tool) subgroup analysis for the effect of SSB on CRP (mg/L) in substitution trials

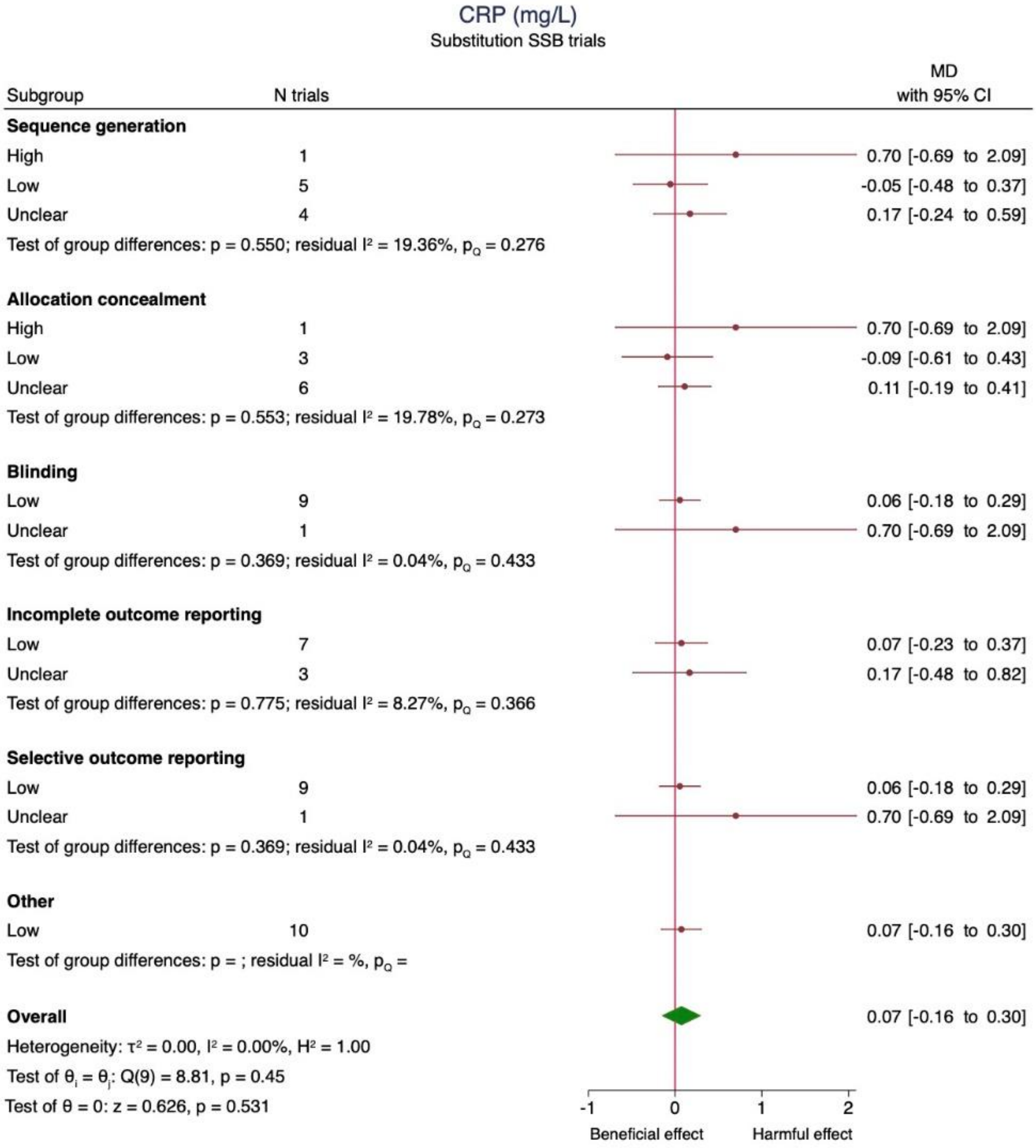

CI=confidence interval; CRP = C reactive protein ; MD= mean difference

The green diamond represents the pooled estimate for the overall primary analysis of food sources of fructose-containing sugars and CRP. Within subgroup mean differences are the pooled effect estimates represented by a red circle. 95% confidence intervals are represented by the line through the circle. Data are expressed as mean differences with 95% confidence intervals using the generic inverse-variance method and random effects DerSimonian-Laird model. Inter-study heterogeneity was assessed using the Cochrane Q statistic and quantified using the I^2 statistic, with significance set at $P_Q<0{\cdot}100$ and $I^2\geq50\%$ considered to be evidence of substantial heterogeneity. P<0·050 indicates that the effect size differed between levels of the subgroup.

Supplemental Figure S56 (part 1 of 3): Subgroup analyses for the effect of 100% fruit juice on CRP (mg/L) in addition trials

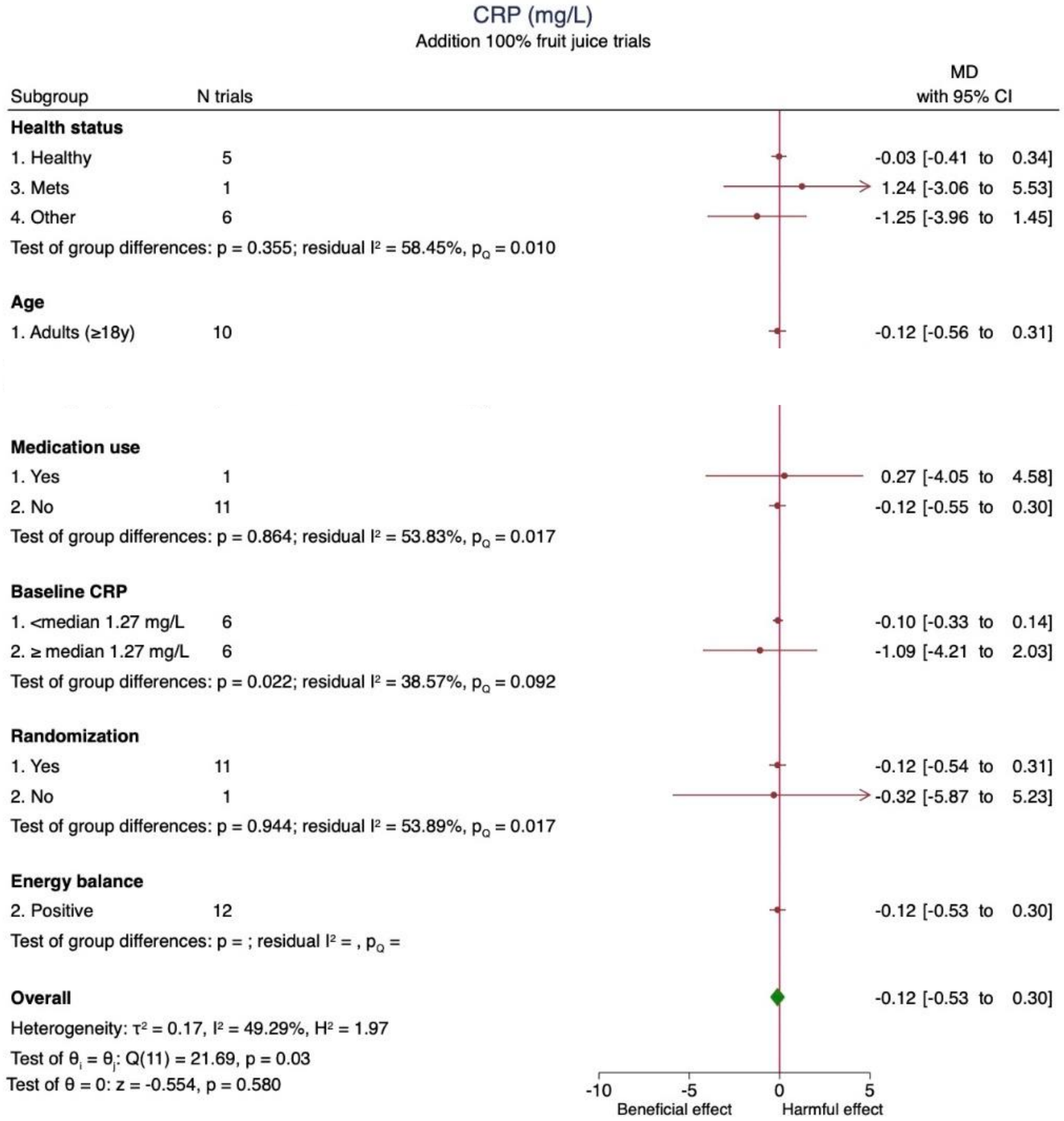

CI=confidence interval; CRP = C reactive protein; MD= mean difference; MetS= metabolic syndrome; y=years
*N=2 trials missing data for age in addition 100% fruit juice trials
**N=1 trial missing for baseline CRP
The green diamond represents the pooled estimate for the overall primary analysis of food sources of fructose-containing sugars and CRP. Within subgroup mean differences are the pooled effect estimates represented by a red circle. 95% confidence intervals are represented by the line through the circle. Data are expressed as mean differences with 95% confidence intervals using the generic inverse-variance method and random effects DerSimonian-Laird model. Inter-study heterogeneity was assessed using the Cochrane Q statistic and quantified using the I^2 statistic, with significance set at $P_Q<0{\cdot}100$ and $I^2\geq50\%$ considered to be evidence of substantial heterogeneity. P<0·050 indicates that the effect size differed between levels of the subgroup.

Supplemental Figure S56 (part 2 of 3): Subgroup analyses for the effect of 100% fruit juice on CRP (mg/L) in addition trials

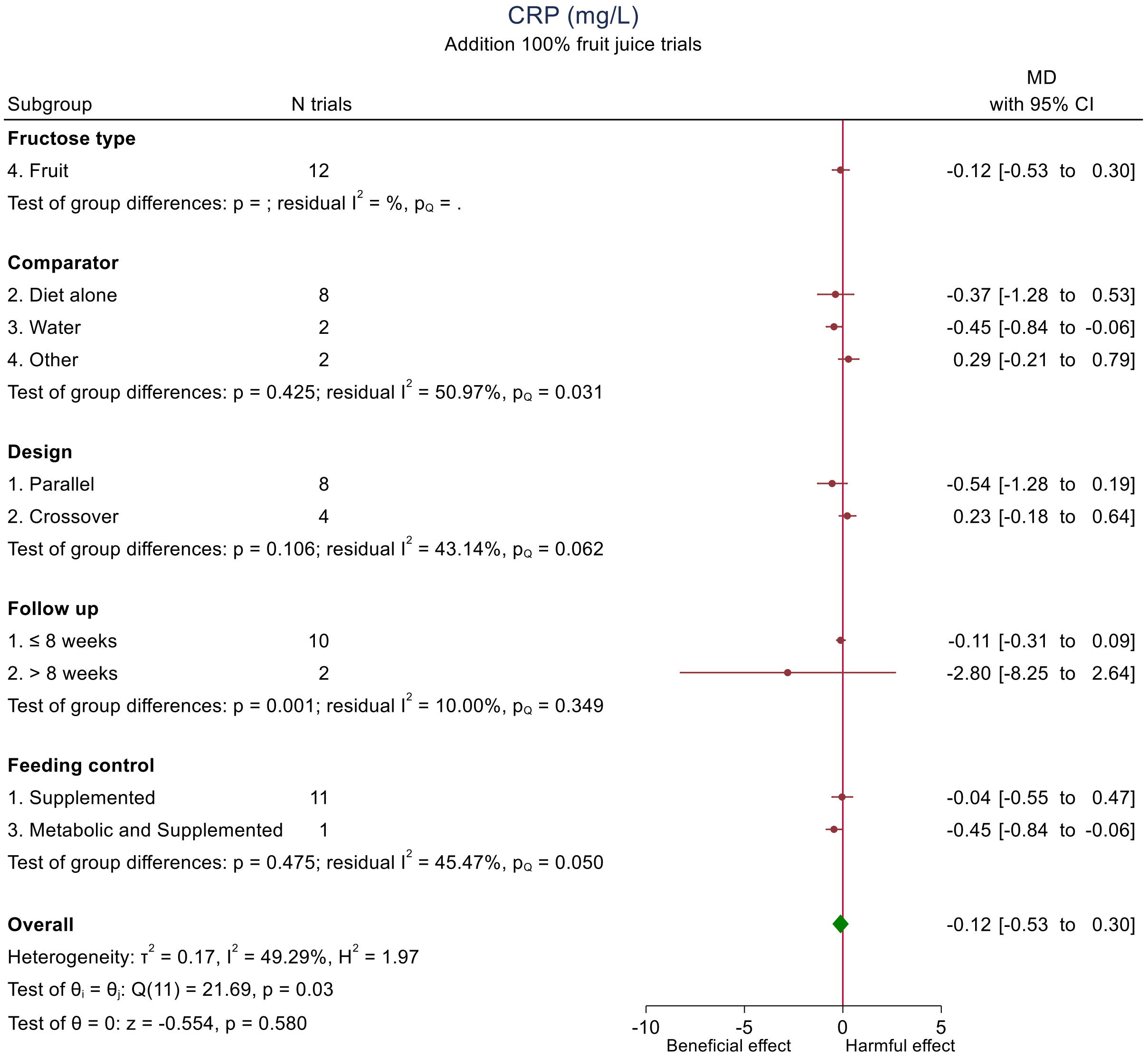

CI=confidence interval; CRP = C reactive protein; MD= mean difference;
The green diamond represents the pooled estimate for the overall primary analysis of food sources of fructose-containing sugars and CRP. Within subgroup mean differences are the pooled effect estimates represented by a red circle. 95% confidence intervals are represented by the line through the circle. Data are expressed as mean differences with 95% confidence intervals using the generic inverse-variance method and random effects DerSimonian-Laird model. Inter-study heterogeneity was assessed using the Cochrane Q statistic and quantified using the I^2 statistic, with significance set at $P_Q<0{\cdot}100$ and $I^2 \geq 50\%$ considered to be evidence of substantial heterogeneity. P<0·050 indicates that the effect size differed between levels of the subgroup.

Supplemental Figure S56 (part 3 of 3): Subgroup analyses for the effect of 100% fruit juice on CRP (mg/L) in addition trials

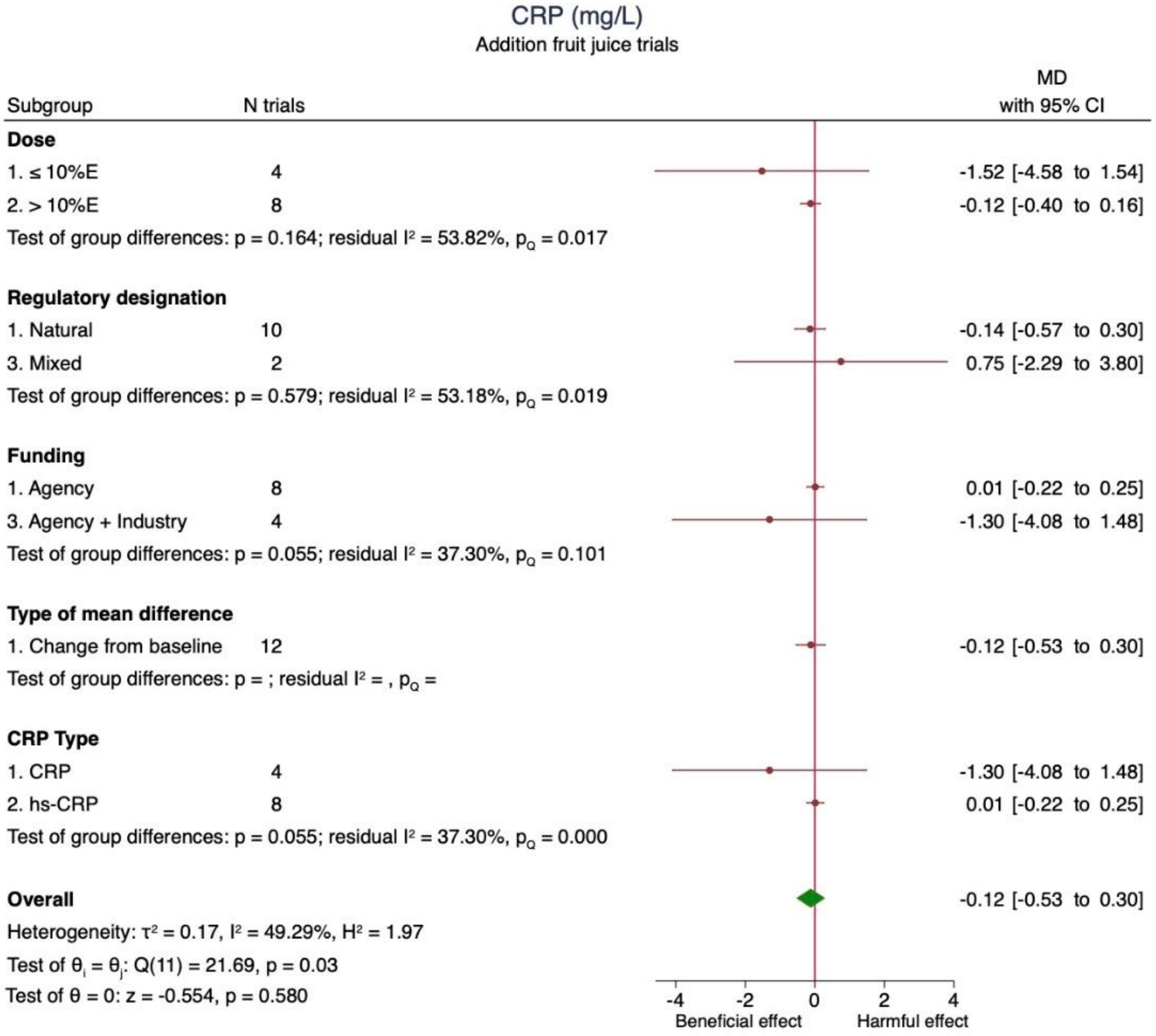

CI=confidence interval; CRP = C reactive protein; E= energy; MD= mean difference;
The green diamond represents the pooled estimate for the overall primary analysis of food sources of fructose-containing sugars and CRP. Within subgroup mean differences are the pooled effect estimates represented by a red circle. 95% confidence intervals are represented by the line through the circle. Data are expressed as mean differences with 95% confidence intervals using the generic inverse-variance method and random effects DerSimonian-Laird model. Inter-study heterogeneity was assessed using the Cochrane Q statistic and quantified using the I^2 statistic, with significance set at $P_Q<0{\cdot}100$ and $I^2\geq50\%$ considered to be evidence of substantial heterogeneity. P<0·050 indicates that the effect size differed between levels of the subgroup.

Supplemental Figure S57: Risk of bias (using The Cochrane Collaboration Tool) subgroup analysis for the effect of 100% fruit juice on CRP (mg/L) in addition trials

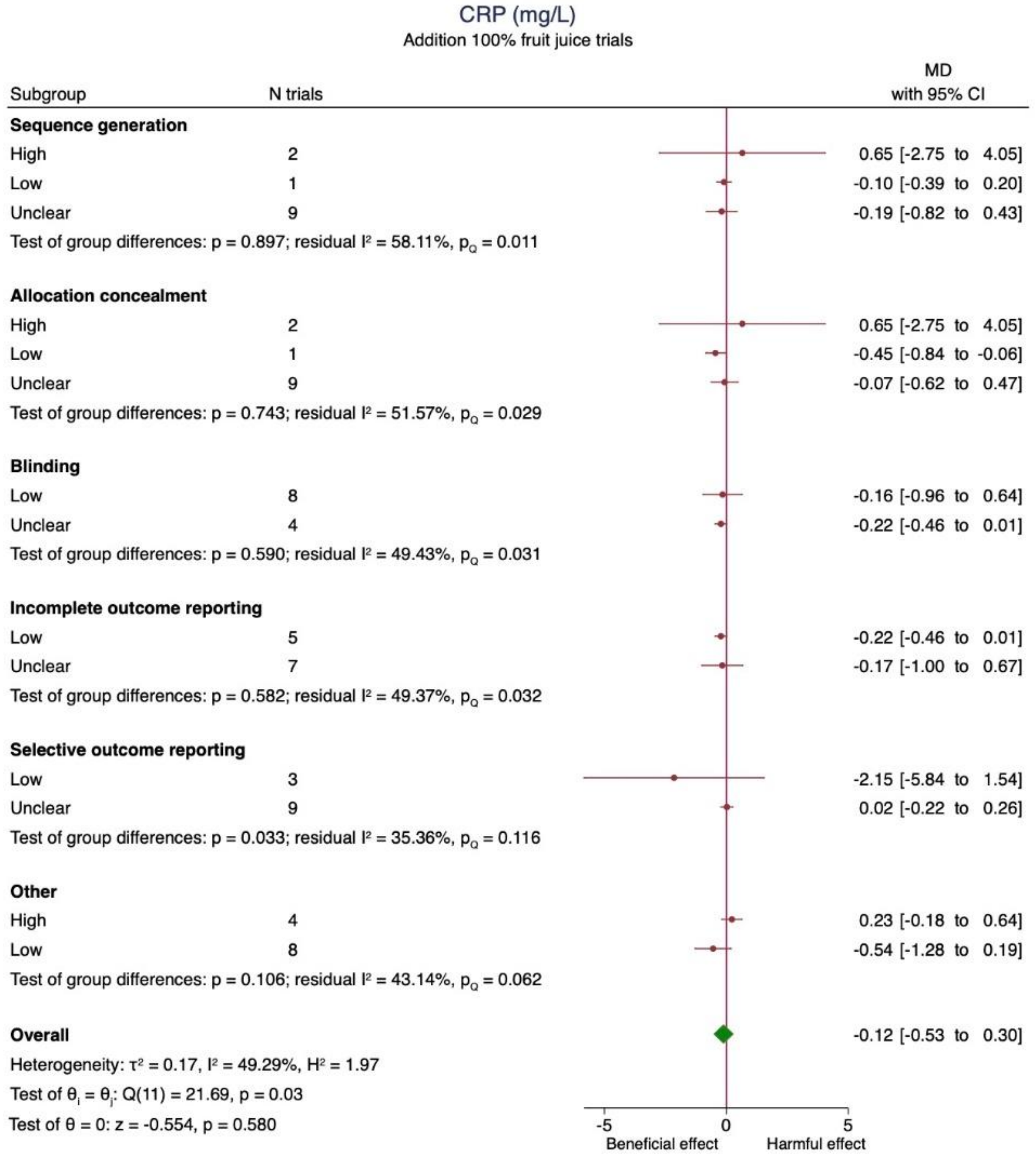

CI=confidence interval; CRP = C reactive protein; MD= mean difference;
The green diamond represents the pooled estimate for the overall primary analysis of food sources of fructose-containing sugars and CRP. Within subgroup mean differences are the pooled effect estimates represented by a red circle. 95% confidence intervals are represented by the line through the circle. Data are expressed as mean differences with 95% confidence intervals using the generic inverse-variance method and random effects DerSimonian-Laird model. Inter-study heterogeneity was assessed using the Cochrane Q statistic and quantified using the I^2 statistic, with significance set at $P_Q<0{\cdot}100$ and $I^2 \geq 50\%$ considered to be evidence of substantial heterogeneity. P<0·050 indicates that the effect size differed between levels of the subgroup.

Supplemental Figure S58: Continuous meta-regression analysis for the effect SSB on CRP (mg/L) in substitution comparisons

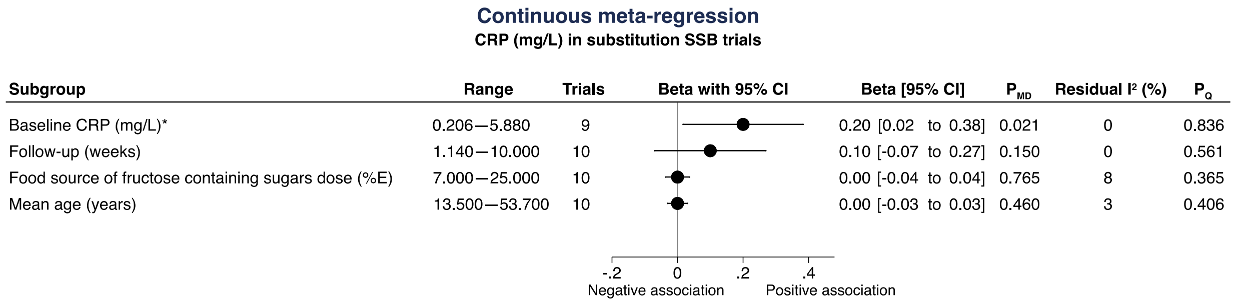

CI=confidence interval; CRP= C reactive protein; %E=percentage of total energy intake
Data is presented as between group mean difference (95% confidence intervals) for a 1-unit change in the predictor variable. ß–coefficients were estimated using continuous meta-regression analysis. A positive ß-coefficient implies an increase in CRP with the food source of fructose-containing sugars intervention as the subgroup variable increases, and a negative ß-coefficient implies a decrease in uric acid. Residual I^2 reports inter-study heterogeneity not explained by the subgroup and was estimated using the Cochrane Q statistic.
* N=1 trials missing data for baseline CRP

Supplemental Figure S59: Continuous meta-regression analysis for the effect of 100% fruit juice on CRP (mg/L) in addition comparisons

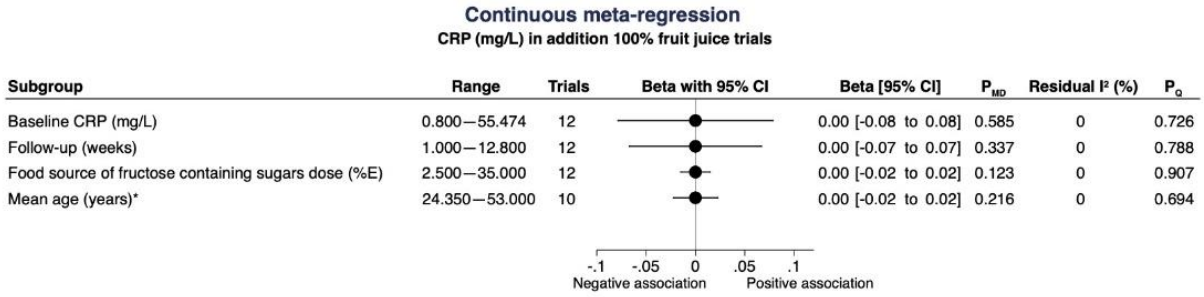

CI=confidence interval; CRP= C reactive protein; %E=percentage of total energy intake
Data is presented as between group mean difference (95% confidence intervals) for a 1-unit change in the predictor variable. ß–coefficients were estimated using continuous meta-regression analysis. A positive ß-coefficient implies an increase in CRP with the food source of fructose-containing sugars intervention as the subgroup variable increases, and a negative ß-coefficient implies a decrease in uric acid. Residual I^2 reports inter-study heterogeneity not explained by the subgroup and was estimated using the Cochrane Q statistic.
*N=2 trials missing data for age in addition 100% fruit juice trials

Supplemental Figure S60 (part 1 of 3): Subgroup analyses for the effect of important food sources of fructose-containing sugars on TNF-α (pg/mL) in substitution trials

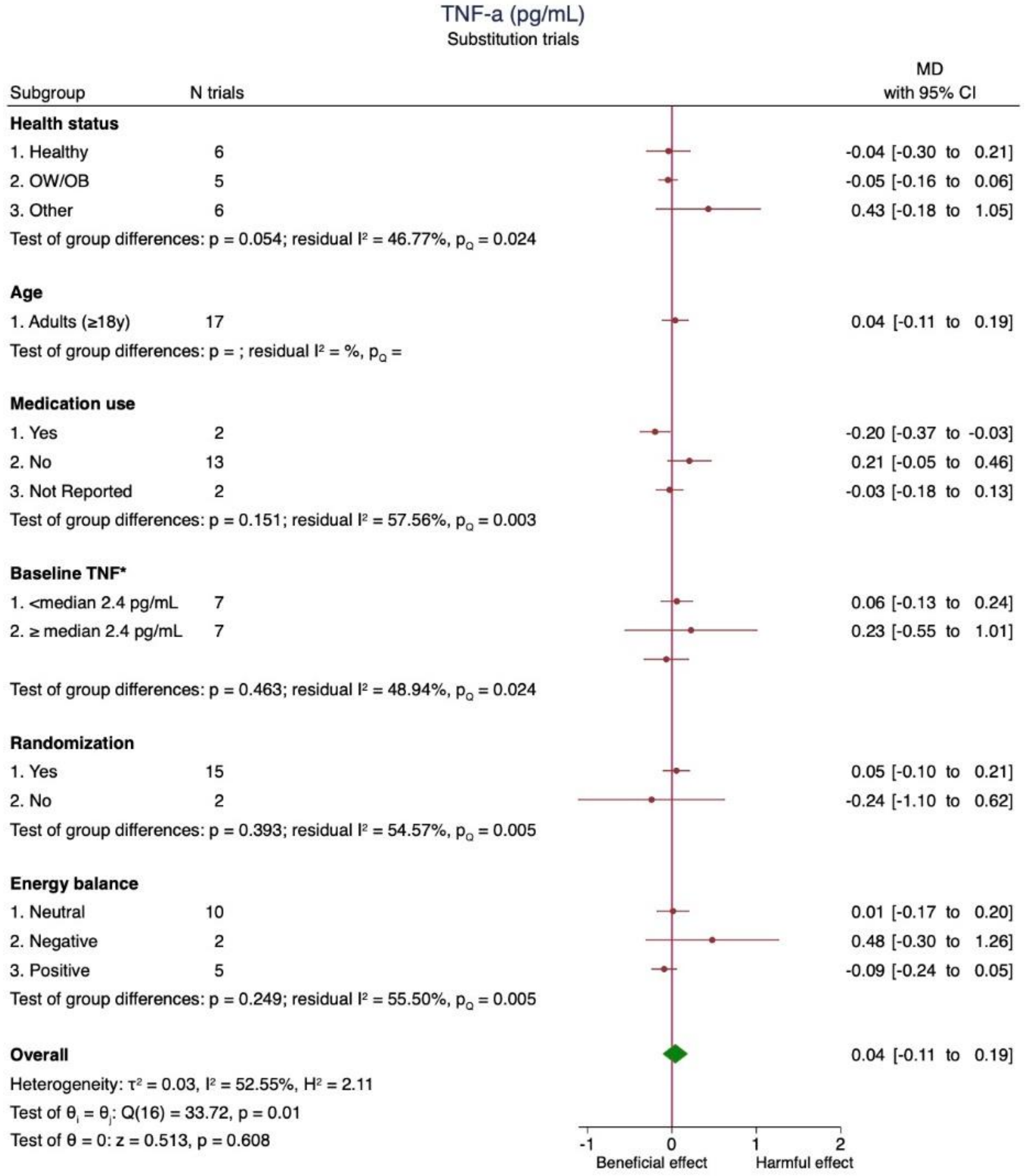

CI=confidence interval; MD= mean difference; OW/OB=overweight or obese; TNF-α = tumour necrosis factor alpha; y=years

*N=3 trials missing for baseline TNF

The green diamond represents the pooled estimate for the overall primary analysis of food sources of fructose-containing sugars and CRP. Within subgroup mean differences are the pooled effect estimates represented by a red circle. 95% confidence intervals are represented by the line through the circle. Data are expressed as mean differences with 95% confidence intervals using the generic inverse-variance method and random effects DerSimonian-Laird model. Inter-study heterogeneity was assessed using the Cochrane Q statistic and quantified using the I^2 statistic, with significance set at P_Q<0·100 and I^2≥50% considered to be evidence of substantial heterogeneity. P<0·050 indicates that the effect size differed between levels of the subgroup.

Supplemental Figure S60 (part 2 of 3): Subgroup analyses for the effect of important food sources of fructose-containing sugars on TNF-α (pg/mL) in substitution trials

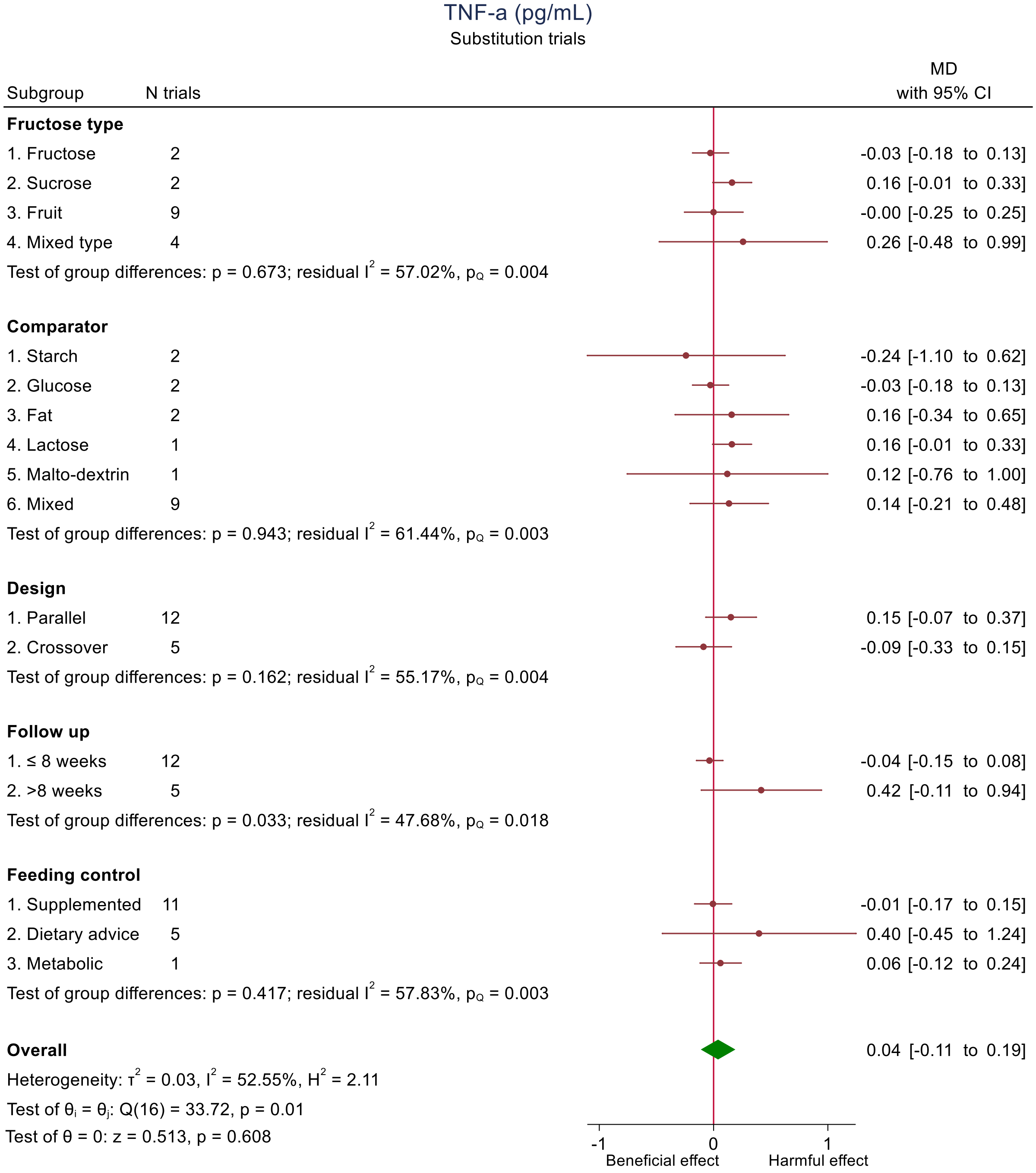

CI=confidence interval; MD= mean difference; TNF-α = tumour necrosis factor-alpha
The green diamond represents the pooled estimate for the overall primary analysis of food sources of fructose-containing sugars and CRP. Within subgroup mean differences are the pooled effect estimates represented by a red circle. 95% confidence intervals are represented by the line through the circle. Data are expressed as mean differences with 95% confidence intervals using the generic inverse-variance method and random effects DerSimonian-Laird model. Inter-study heterogeneity was assessed using the Cochrane Q statistic and quantified using the I^2 statistic, with significance set at $P_Q<0{\cdot}100$ and $I^2 \geq 50\%$ considered to be evidence of substantial heterogeneity. P<0·050 indicates that the effect size differed between levels of the subgroup.

Supplemental Figure S60 (part 3 of 3): Subgroup analyses for the effect of important food sources of fructose-containing sugars on TNF-α (pg/mL) in substitution trials

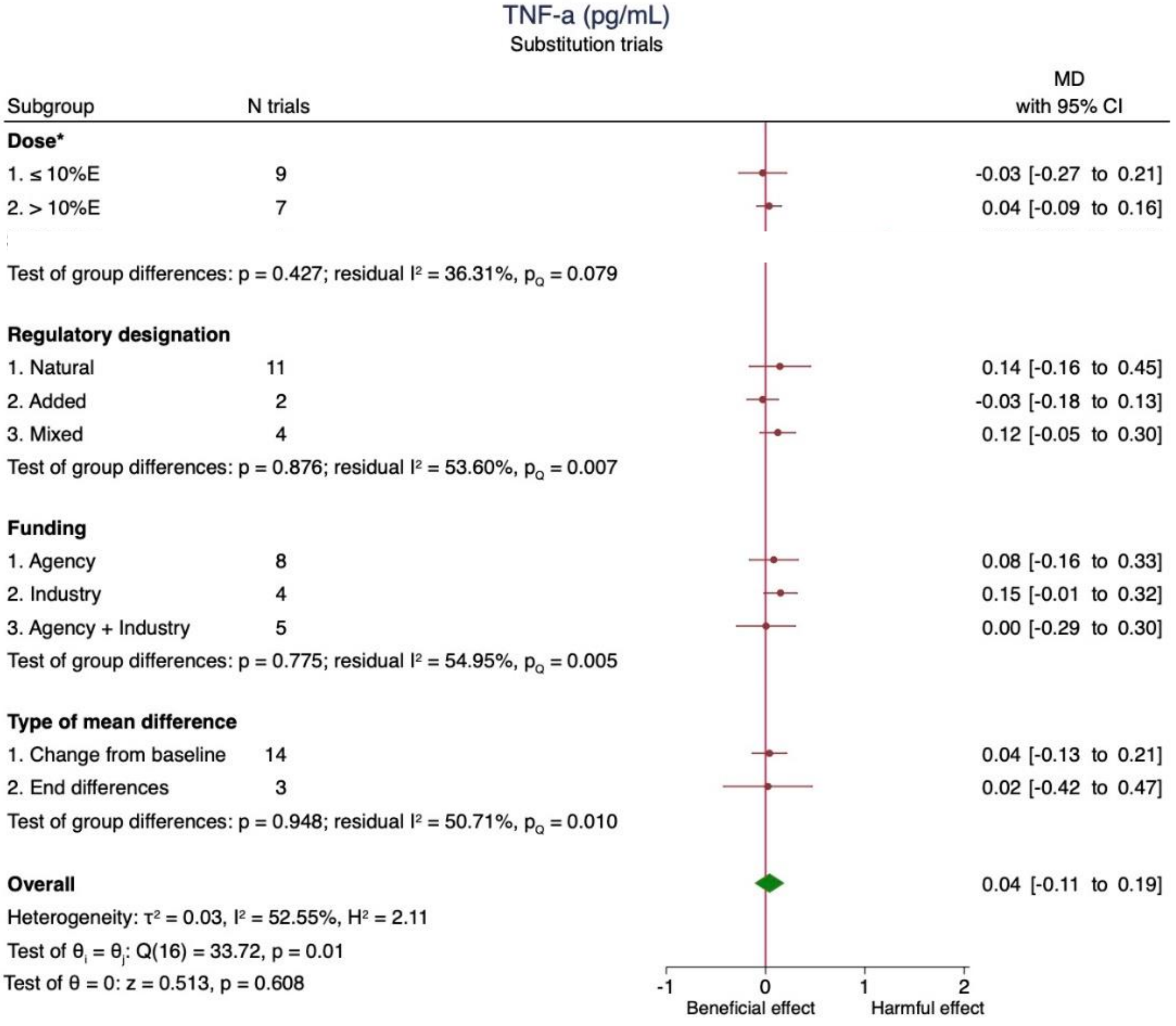

CI=confidence interval; E= Energy; MD= mean difference; TNF-α = tumour necrosis factor alpha;
The green diamond represents the pooled estimate for the overall primary analysis of food sources of fructose-containing sugars and CRP. Within subgroup mean differences are the pooled effect estimates represented by a red circle. 95% confidence intervals are represented by the line through the circle. Data are expressed as mean differences with 95% confidence intervals using the generic inverse-variance method and random effects DerSimonian-Laird model. Inter-study heterogeneity was assessed using the Cochrane Q statistic and quantified using the I^2 statistic, with significance set at P_Q<0·100 and I^2≥50% considered to be evidence of substantial heterogeneity. P<0·050 indicates that the effect size differed between levels of the subgroup.

Supplemental Figure S61: Risk of bias (using The Cochrane Collaboration Tool) subgroup analysis for the effect of important food sources of fructose-containing sugars on TNF-α (pg/mL) in substitution trials

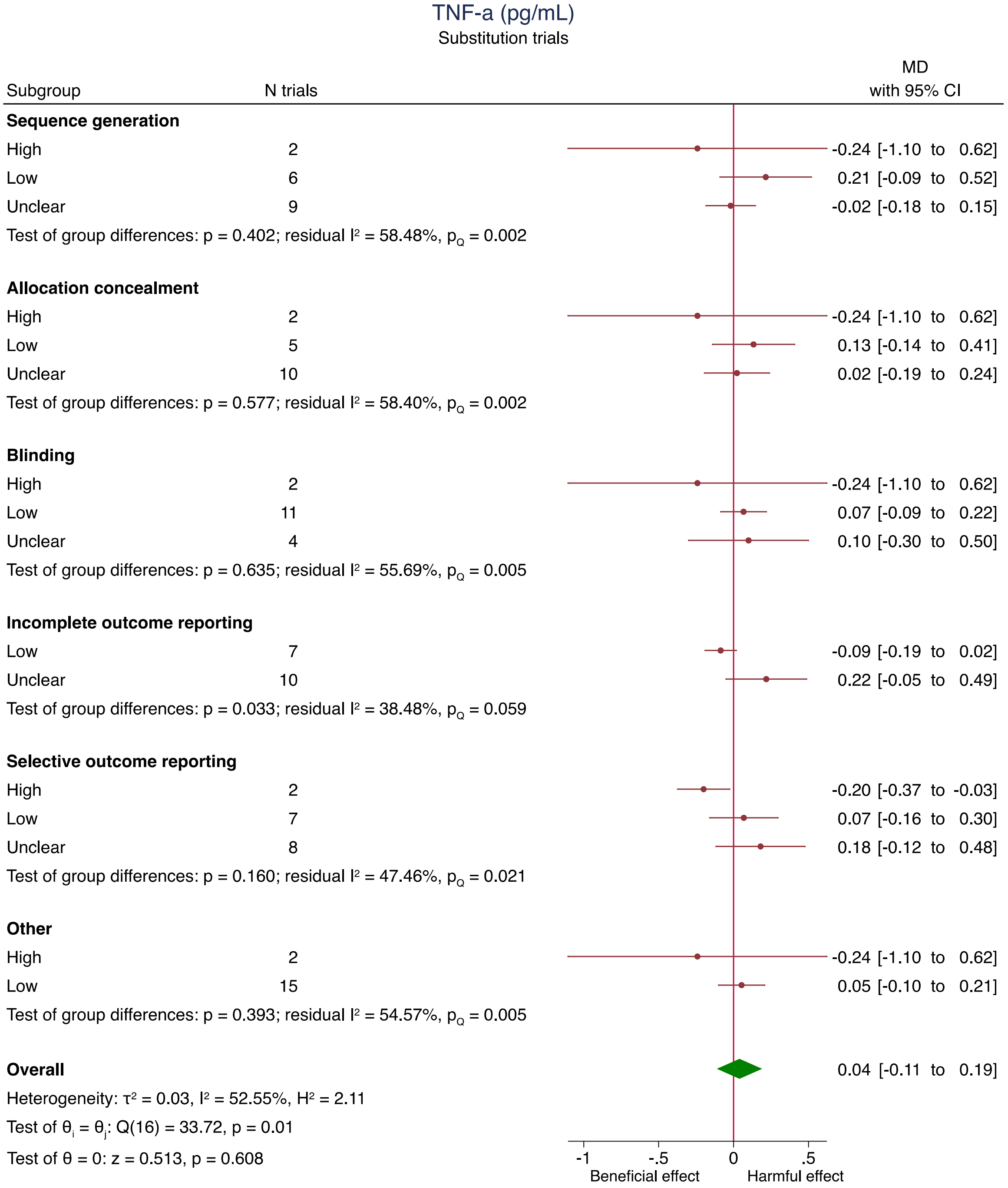

CI=confidence interval; MD= mean difference; TNF-α = tumour necrosis factor-alpha

The green diamond represents the pooled estimate for the overall primary analysis of food sources of fructose-containing sugars and CRP. Within subgroup mean differences are the pooled effect estimates represented by a red circle. 95% confidence intervals are represented by the line through the circle. Data are expressed as mean differences with 95% confidence intervals using the generic inverse-variance method and random effects DerSimonian-Laird model. Inter-study heterogeneity was assessed using the Cochrane Q statistic and quantified using the I^2 statistic, with significance set at P_Q<0·100 and I^2≥50% considered to be evidence of substantial heterogeneity. P<0·050 indicates that the effect size differed between levels of the subgroup.

Supplemental Figure S62 (part 1 of 3): Subgroup analyses for the effect of important food sources of fructose-containing sugars on TNF-α (pg/mL) in addition trials

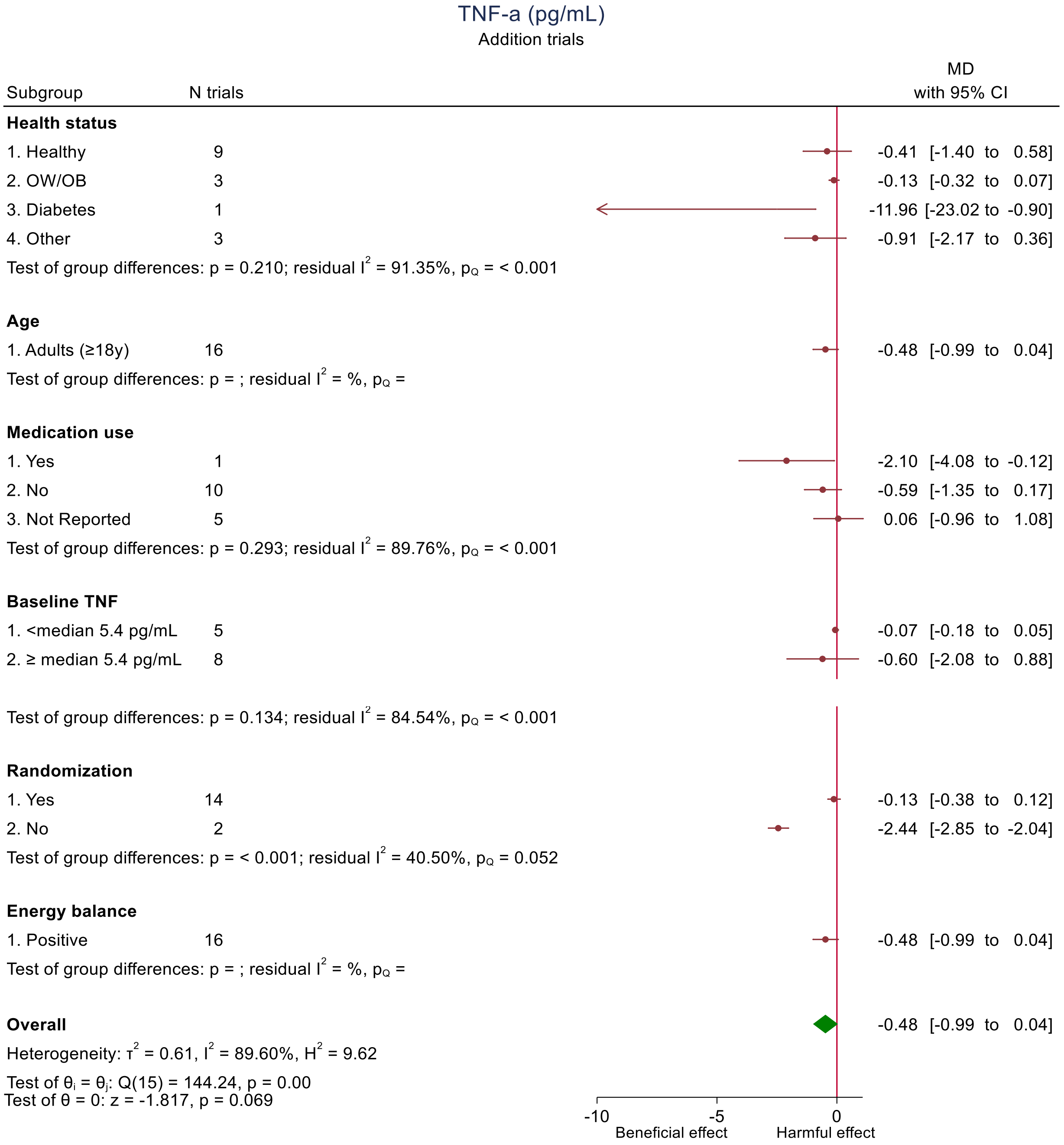

CI=confidence interval; MD=mean difference; OW/OB=overweight or obese; TNF-α = tumour necrosis factor-alpha
*N=4 trials missing in baseline TNF-α
The green diamond represents the pooled estimate for the overall primary analysis of food sources of fructose-containing sugars and CRP. Within subgroup mean differences are the pooled effect estimates represented by a red circle. 95% confidence intervals are represented by the line through the circle. Data are expressed as mean differences with 95% confidence intervals using the generic inverse-variance method and random effects DerSimonian-Laird model. Inter-study heterogeneity was assessed using the Cochrane Q statistic and quantified using the I^2 statistic, with significance set at P_Q<0·100 and I^2≥50% considered to be evidence of substantial heterogeneity. P<0·050 indicates that the effect size differed between levels of the subgroup.

Supplemental Figure S62 (part 2 of 3): Subgroup analyses for the effect of important food sources of fructose-containing sugars on TNF-α (pg/mL) in addition trials

TNF-a (pg/mL)

Addition trials

Subgroup	N trials	MD with 95% CI
**Fructose type**		
1. Sucrose	7	-0.06 [-0.28 to 0.16]
2. Honey	3	-0.91 [-2.17 to 0.36]
3. Fruit	6	-0.89 [-2.07 to 0.29]
Test of group differences: p = 0.119; residual I^2 = 83.83%, p_Q = < 0.001		
**Comparator**		
1. NNS	3	0.79 [-1.23 to 2.80]
2. Diet alone	12	-0.71 [-1.42 to -0.01]
3. Water	1	0.10 [-1.99 to 2.19]
Test of group differences: p = 0.258; residual I^2 = 90.98%, p_Q = < 0.001		
**Design**		
1. Parallel	12	-0.09 [-0.33 to 0.16]
2. Crossover	4	-1.58 [-2.69 to -0.46]
Test of group differences: p = < 0.001; residual I^2 = 53.97%, p_Q = 0.007		
**Follow up**		
1. ≤ 8 weeks	15	-0.51 [-1.18 to 0.15]
2. > 8 weeks	1	-0.04 [-0.18 to 0.10]
Test of group differences: p = 0.659; residual I^2 = 88.93%, p_Q = < 0.001		
**Feeding control**		
1. Supplemented	12	-0.64 [-1.26 to -0.02]
2. Metabolic	3	-0.14 [-1.17 to 0.89]
3. Metabolic and Supplemented	1	1.37 [-2.19 to 4.93]
Test of group differences: p = 0.454; residual I^2 = 90.94%, p_Q = < 0.001		
**Overall**		-0.48 [-0.99 to 0.04]
Heterogeneity: τ^2 = 0.61, I^2 = 89.60%, H^2 = 9.62		
Test of $\theta_i = \theta_j$: Q(15) = 144.24, p = 0.00		
Test of θ = 0: z = -1.817, p = 0.069		

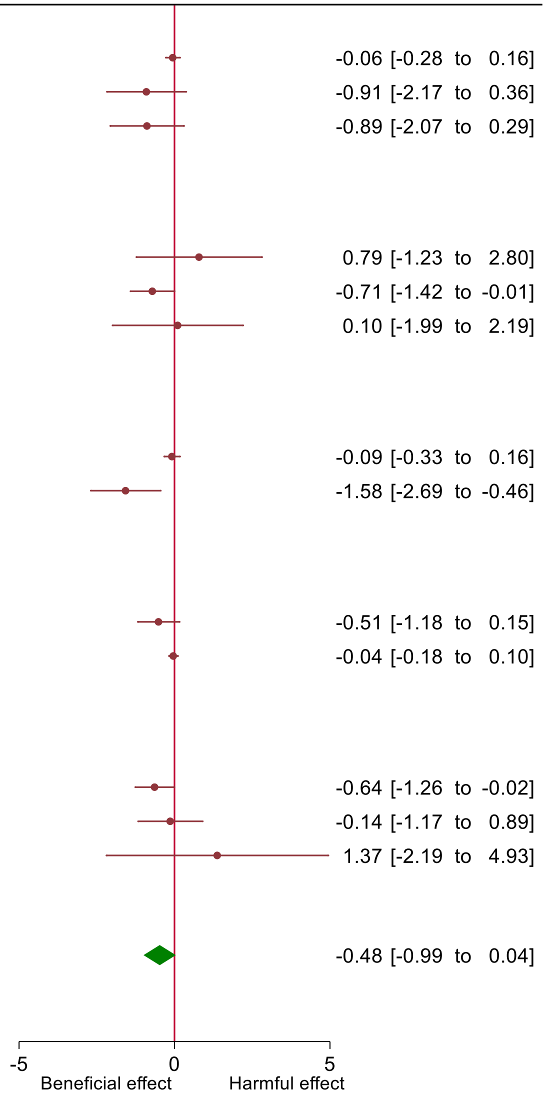

CI=confidence interval; HFCS= high fructose corn syrup; MD= mean difference; NNS= non-nutritive sweetener; TNF-α = tumour necrosis factor-alpha

The green diamond represents the pooled estimate for the overall primary analysis of food sources of fructose-containing sugars and CRP. Within subgroup mean differences are the pooled effect estimates represented by a red circle. 95% confidence intervals are represented by the line through the circle. Data are expressed as mean differences with 95% confidence intervals using the generic inverse-variance method and random effects DerSimonian-Laird model. Inter-study heterogeneity was assessed using the Cochrane Q statistic and quantified using the I^2 statistic, with significance set at $P_Q<0{\cdot}100$ and $I^2 \geq 50\%$ considered to be evidence of substantial heterogeneity. P<0·050 indicates that the effect size differed between levels of the subgroup.

Supplemental Figure S62 (part 3 of 3): Subgroup analyses for the effect of important food sources of fructose-containing sugars on TNF-α (pg/mL) in addition trials

TNF-a (pg/mL)

Addition trials

Subgroup	N trials	MD with 95% CI
**Dose**		
1. ≤ 10%E	10	-0.61 [-1.49 to 0.27]
2. > 10%E	6	-0.19 [-0.76 to 0.37]
Test of group differences: p = 0.562; residual I^2 = 88.79%, p_Q = < 0.001		
**Regulatory designation**		
1. Natural	8	-0.62 [-1.46 to 0.23]
2. Added	7	-0.19 [-1.29 to 0.91]
3. Mixed	1	-0.34 [-0.99 to 0.32]
Test of group differences: p = 0.768; residual I^2 = 90.98%, p_Q = < 0.001		
**Funding**		
1. Agency	7	-1.64 [-2.62 to -0.66]
2. Industry	1	0.10 [-1.99 to 2.19]
3. Agency + Industry	6	-0.07 [-0.23 to 0.09]
4. NR	2	1.76 [-1.48 to 5.00]
Test of group differences: p = < 0.001; residual I^2 = 57.20%, p_Q = 0.005		
**Type of mean difference**		
1. Change from baseline	11	-0.35 [-1.29 to 0.59]
2. End differences	5	-0.23 [-0.53 to 0.08]
Test of group differences: p = 0.738; residual I^2 = 90.24%, p_Q = < 0.001		
**Overall**		-0.48 [-0.99 to 0.04]
Heterogeneity: τ^2 = 0.61, I^2 = 89.60%, H^2 = 9.62		
Test of $\theta_i = \theta_j$: Q(15) = 144.24, p = 0.00		
Test of θ = 0: z = -1.817, p = 0.069		

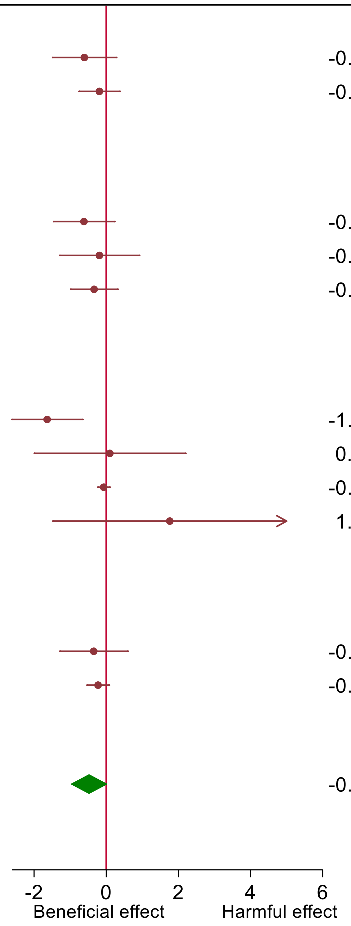

CI=confidence interval; E=energy; MD= mean difference; NR= not reported; TNF-α = tumour necrosis factor alpha; The green diamond represents the pooled estimate for the overall primary analysis of food sources of fructose-containing sugars and CRP. Within subgroup mean differences are the pooled effect estimates represented by a red circle. 95% confidence intervals are represented by the line through the circle. Data are expressed as mean differences with 95% confidence intervals using the generic inverse-variance method and random effects DerSimonian-Laird model. Inter-study heterogeneity was assessed using the Cochrane Q statistic and quantified using the I^2 statistic, with significance set at P_Q<0·100 and I^2≥50% considered to be evidence of substantial heterogeneity. P<0·050 indicates that the effect size differed between levels of the subgroup.

Supplemental Figure S63: Risk of bias (using The Cochrane Collaboration Tool) subgroup analysis for the effect of important food sources of fructose-containing sugars on TNF-α (pg/mL) in addition trials

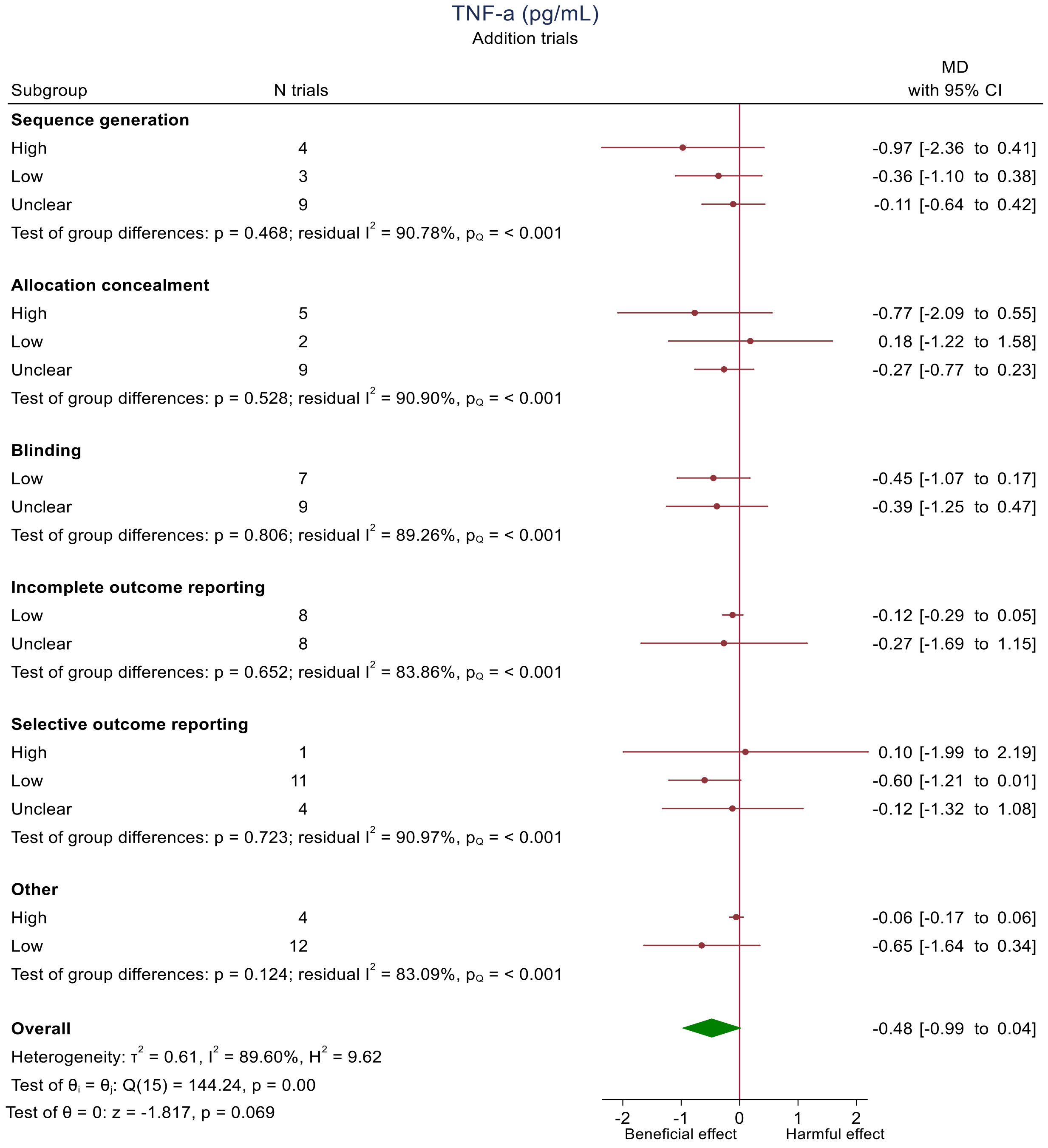

CI=confidence interval; MD= mean difference; TNF-α = tumour necrosis factor-alpha

The green diamond represents the pooled estimate for the overall primary analysis of food sources of fructose-containing sugars and CRP. Within subgroup mean differences are the pooled effect estimates represented by a red circle. 95% confidence intervals are represented by the line through the circle. Data are expressed as mean differences with 95% confidence intervals using the generic inverse-variance method and random effects DerSimonian-Laird model. Inter-study heterogeneity was assessed using the Cochrane Q statistic and quantified using the I^2 statistic, with significance set at $P_Q<0{\cdot}100$ and $I^2\geq50\%$ considered to be evidence of substantial heterogeneity. P<0·050 indicates that the effect size differed between levels of the subgroup.

Supplemental Figure S64 (part 1 of 3): Subgroup analyses for the effect of important food sources of fructose-containing sugars on IL-6 (pg/mL) in substitution trials

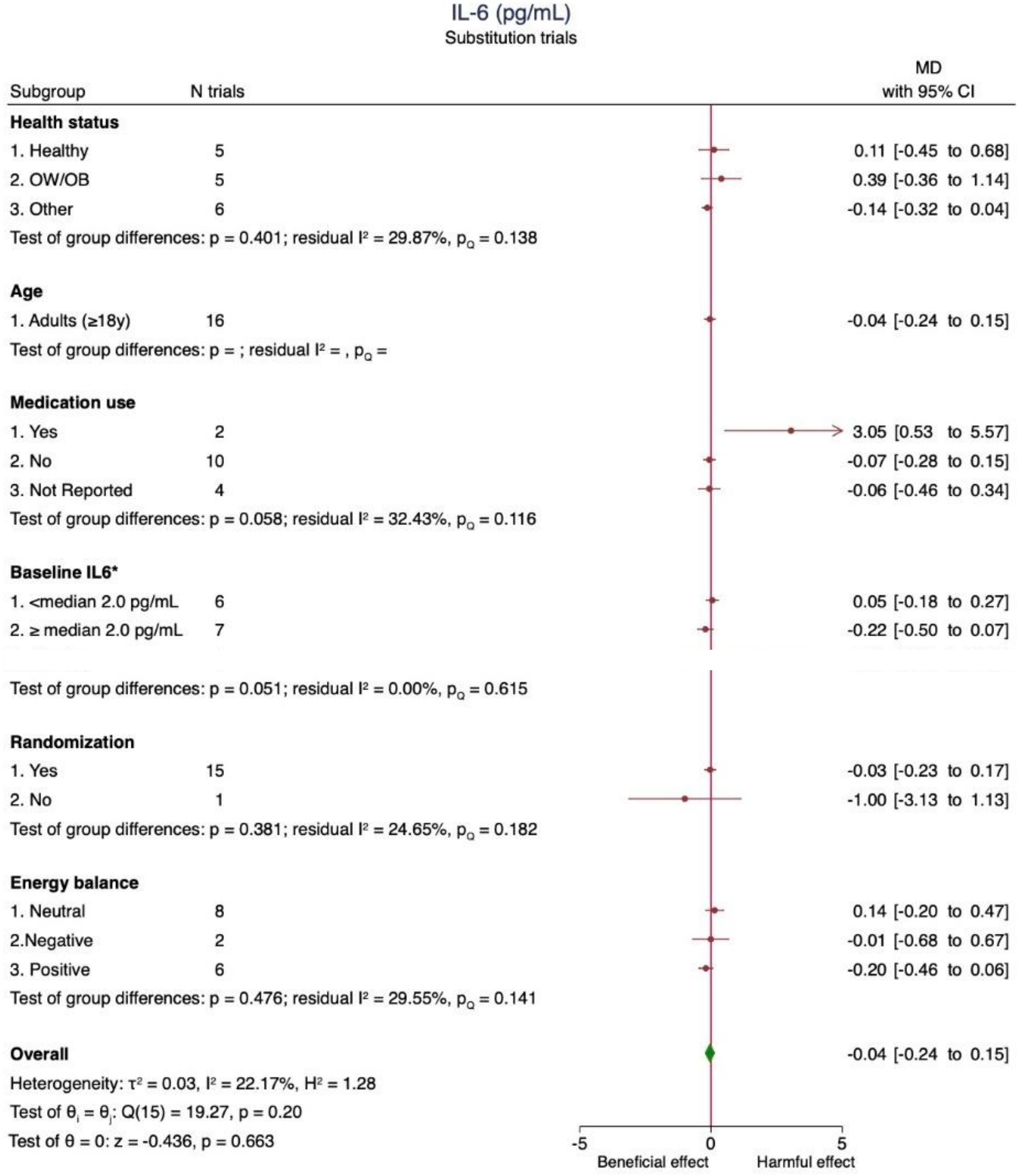

CI=confidence interval; MD= mean difference; OW/OB=overweight or obese; IL-6=interleukin-6; y=years

*N=3 trials missing in baseline IL-6

The green diamond represents the pooled estimate for the overall primary analysis of food sources of fructose-containing sugars and CRP. Within subgroup mean differences are the pooled effect estimates represented by a red circle. 95% confidence intervals are represented by the line through the circle. Data are expressed as mean differences with 95% confidence intervals using the generic inverse-variance method and random effects DerSimonian-Laird model. Inter-study heterogeneity was assessed using the Cochrane Q statistic and quantified using the I^2 statistic, with significance set at $P_Q<0·100$ and $I^2≥50\%$ considered to be evidence of substantial heterogeneity. P<0·050 indicates that the effect size differed between levels of the subgroup.

Supplemental Figure S64 (part 2 of 3): Subgroup analyses for the effect of important food sources of fructose-containing sugars on IL-6 (pg/mL) in substitution trials

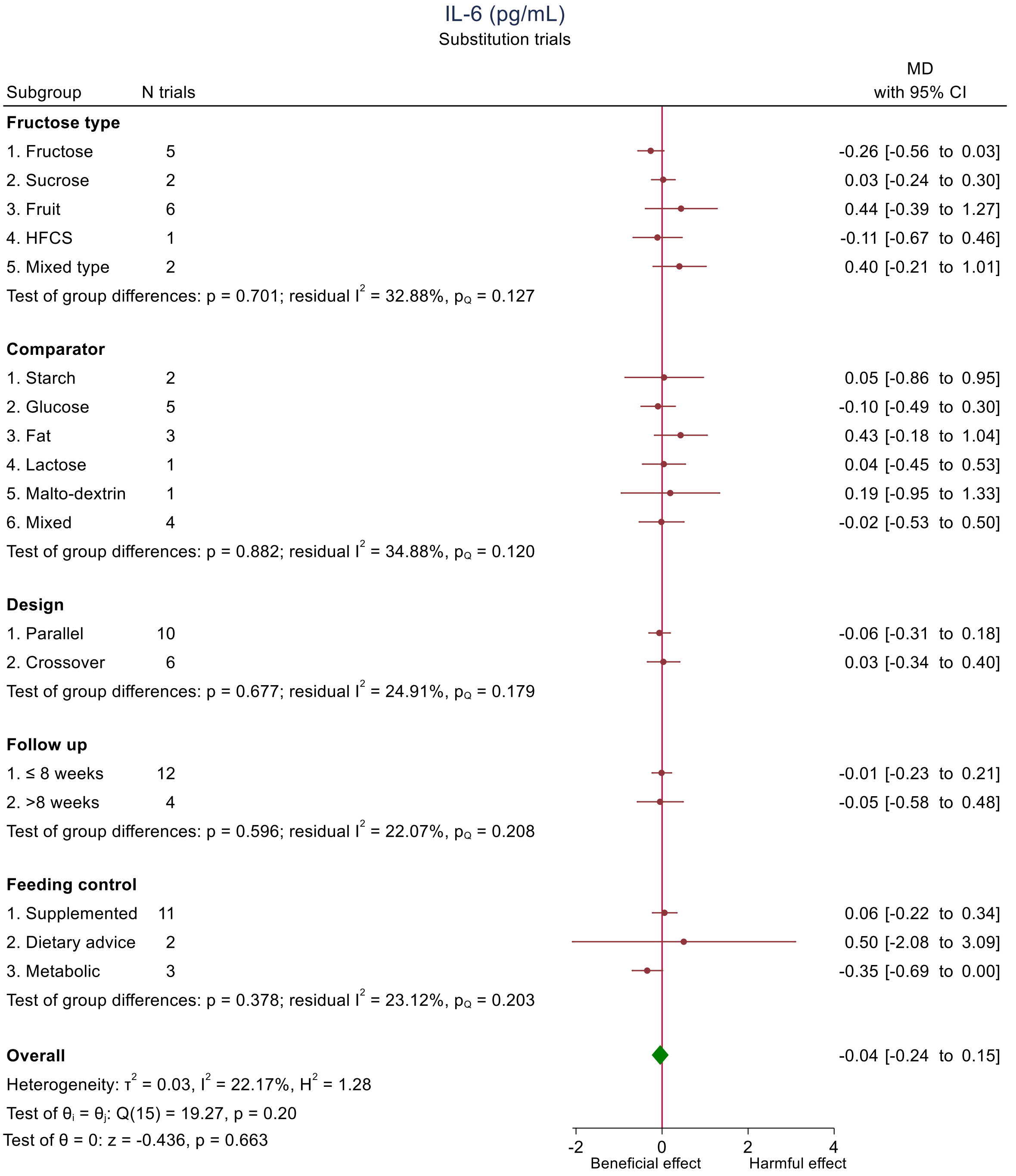

CI=confidence interval; MD= mean difference; HFCS= high fructose corn syrup; IL-6=interleukin-6
The green diamond represents the pooled estimate for the overall primary analysis of food sources of fructose-containing sugars and CRP. Within subgroup mean differences are the pooled effect estimates represented by a red circle. 95% confidence intervals are represented by the line through the circle. Data are expressed as mean differences with 95% confidence intervals using the generic inverse-variance method and random effects DerSimonian-Laird model. Inter-study heterogeneity was assessed using the Cochrane Q statistic and quantified using the I^2 statistic, with significance set at $P_Q<0{\cdot}100$ and $I^2\geq50\%$ considered to be evidence of substantial heterogeneity. $P<0{\cdot}050$ indicates that the effect size differed between levels of the subgroup.

Supplemental Figure S64 (part 3 of 3): Subgroup analyses for the effect of important food sources of fructose-containing sugars on IL-6 (pg/mL) in substitution trials

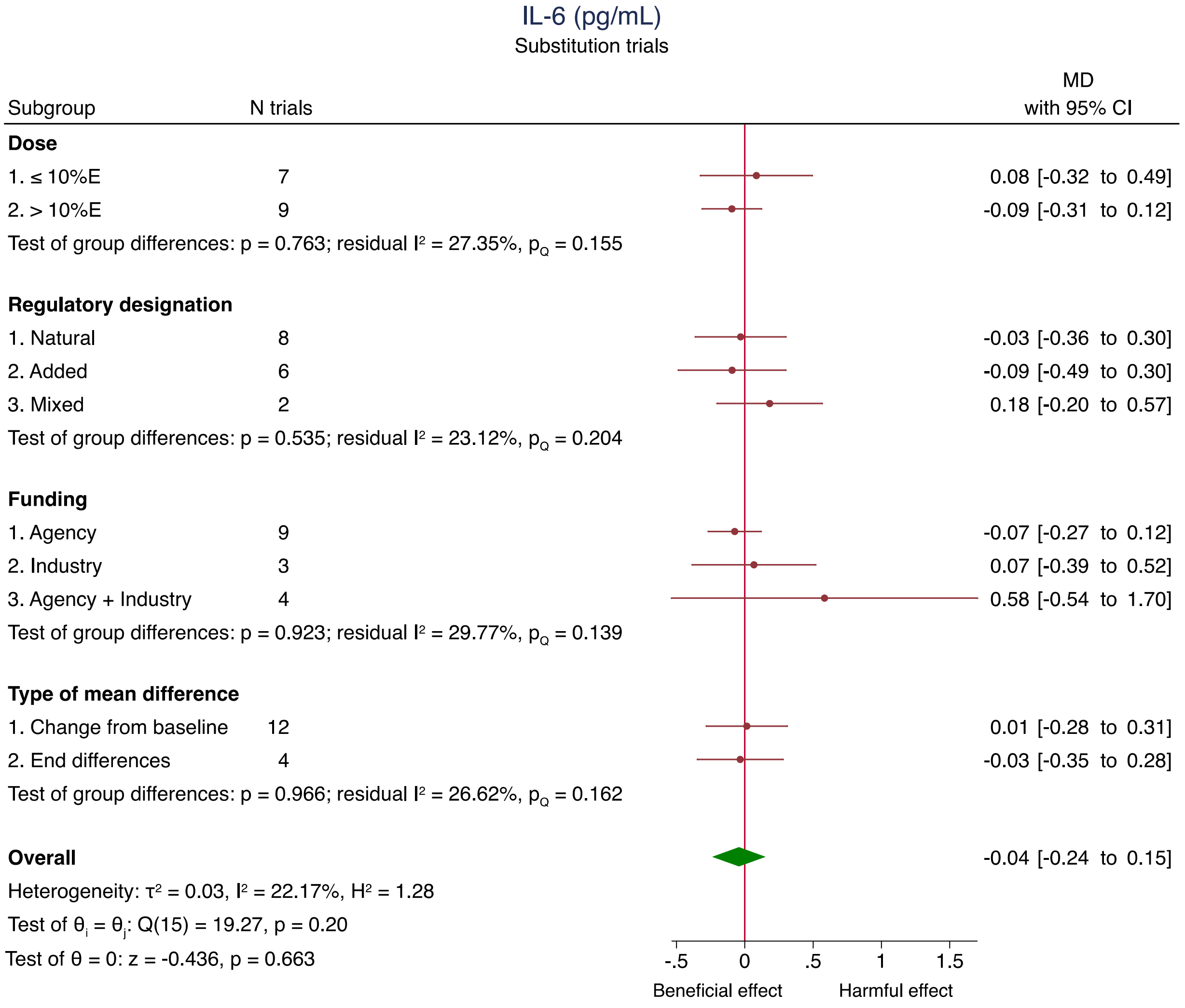

CI=confidence interval; E= energy; MD= mean difference; IL-6=interleukin-6

The green diamond represents the pooled estimate for the overall primary analysis of food sources of fructose-containing sugars and CRP. Within subgroup mean differences are the pooled effect estimates represented by a red circle. 95% confidence intervals are represented by the line through the circle. Data are expressed as mean differences with 95% confidence intervals using the generic inverse-variance method and random effects DerSimonian-Laird model. Inter-study heterogeneity was assessed using the Cochrane Q statistic and quantified using the I^2 statistic, with significance set at P_Q<0·100 and I^2≥50% considered to be evidence of substantial heterogeneity. P<0·050 indicates that the effect size differed between levels of the subgroup.

Supplemental Figure S65: Risk of bias (using The Cochrane Collaboration Tool) subgroup analysis for the effect of important food sources of fructose-containing sugars on IL-6 (pg/mL) in substitution trials

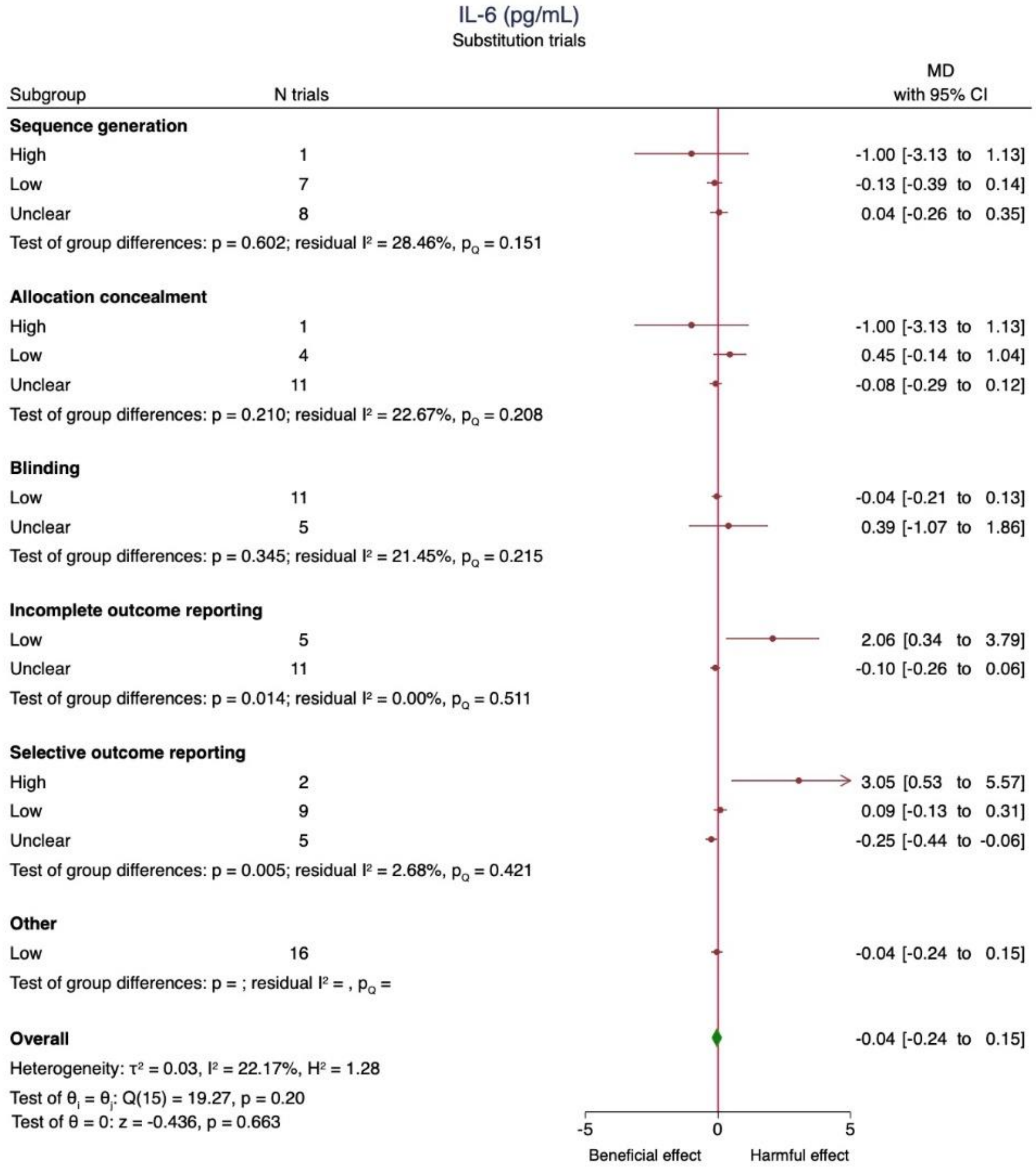

CI=confidence interval; MD= mean difference; HFCS= high fructose corn syrup; IL-6=interleukin-6
The green diamond represents the pooled estimate for the overall primary analysis of food sources of fructose-containing sugars and CRP. Within subgroup mean differences are the pooled effect estimates represented by a red circle. 95% confidence intervals are represented by the line through the circle. Data are expressed as mean differences with 95% confidence intervals using the generic inverse-variance method and random effects DerSimonian-Laird model. Inter-study heterogeneity was assessed using the Cochrane Q statistic and quantified using the I^2 statistic, with significance set at $P_Q<0{\cdot}100$ and $I^2 \geq 50\%$ considered to be evidence of substantial heterogeneity. P<0·050 indicates that the effect size differed between levels of the subgroup.

Supplemental Figure S66 (part 1 of 3): Subgroup analyses for the effect of important food sources of fructose-containing sugars on IL-6 (pg/mL) in addition trials

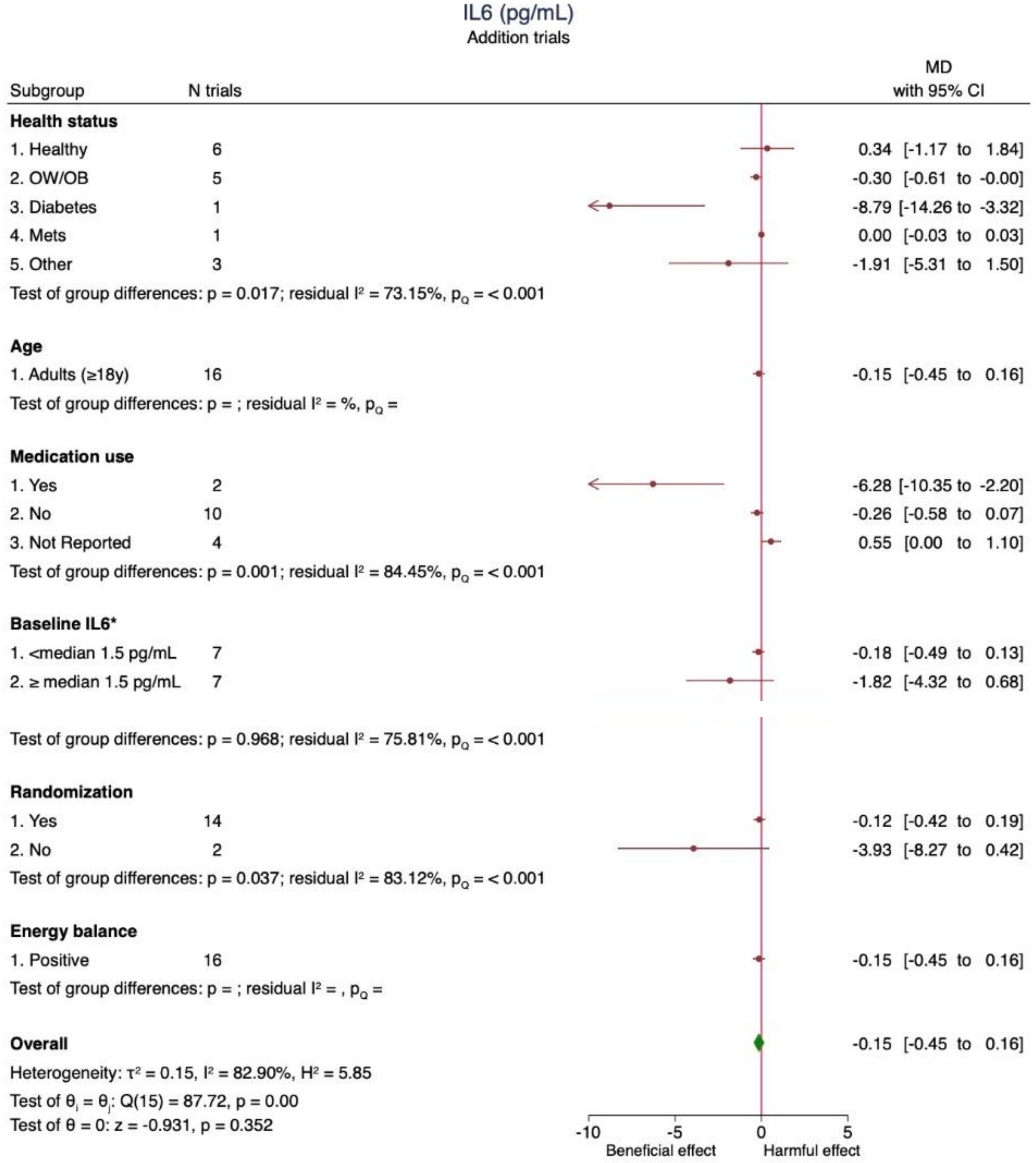

CI=confidence interval; MD= mean difference; Mets= Metabolic syndrome; OW/OB=overweight or obese; IL-6=interleukin-6

*N=2 trials missing from baseline IL-6

The green diamond represents the pooled estimate for the overall primary analysis of food sources of fructose-containing sugars and CRP. Within subgroup mean differences are the pooled effect estimates represented by a red circle. 95% confidence intervals are represented by the line through the circle. Data are expressed as mean differences with 95% confidence intervals using the generic inverse-variance method and random effects DerSimonian-Laird model. Inter-study heterogeneity was assessed using the Cochrane Q statistic and quantified using the I^2 statistic, with significance set at $P_Q<0·100$ and $I^2≥50\%$ considered to be evidence of substantial heterogeneity. P<0·050 indicates that the effect size differed between levels of the subgroup.

Supplemental Figure S66 (part 2 of 3): Subgroup analyses for the effect of important food sources of fructose-containing sugars on IL-6 (pg/mL) in addition trials

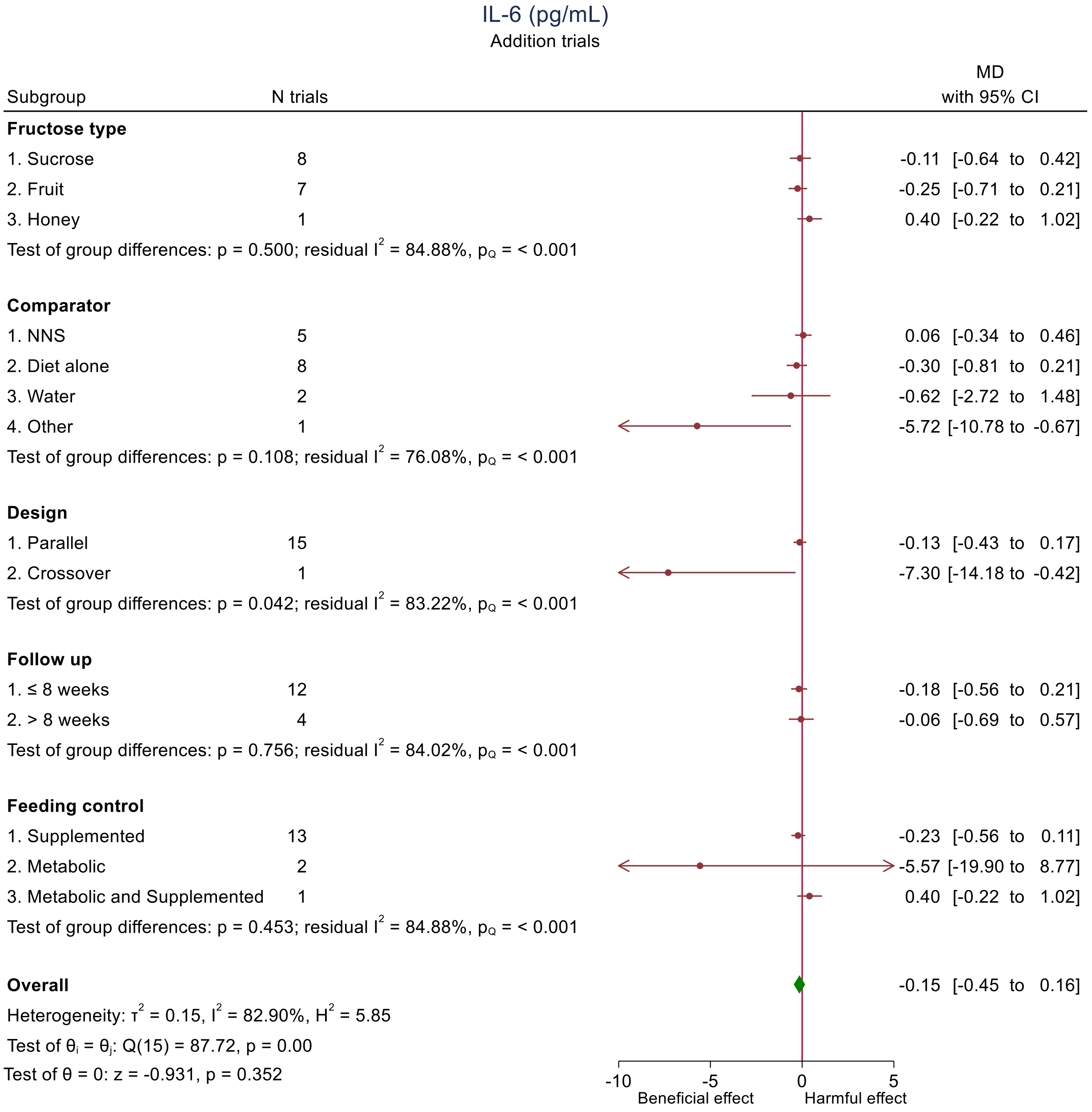

CI=confidence interval; MD= mean difference; HFCS= high fructose corn syrup; NNS= non-nutritive sweetener; IL-6=interleukin-6

The green diamond represents the pooled estimate for the overall primary analysis of food sources of fructose-containing sugars and CRP. Within subgroup mean differences are the pooled effect estimates represented by a red circle. 95% confidence intervals are represented by the line through the circle. Data are expressed as mean differences with 95% confidence intervals using the generic inverse-variance method and random effects DerSimonian-Laird model. Inter-study heterogeneity was assessed using the Cochrane Q statistic and quantified using the I^2 statistic, with significance set at P_Q<0·100 and I^2≥50% considered to be evidence of substantial heterogeneity. P<0·050 indicates that the effect size differed between levels of the subgroup.

Supplemental Figure S66 (part 3 of 3): Subgroup analyses for the effect of important food sources of fructose-containing sugars on IL-6 (pg/mL) in addition trials

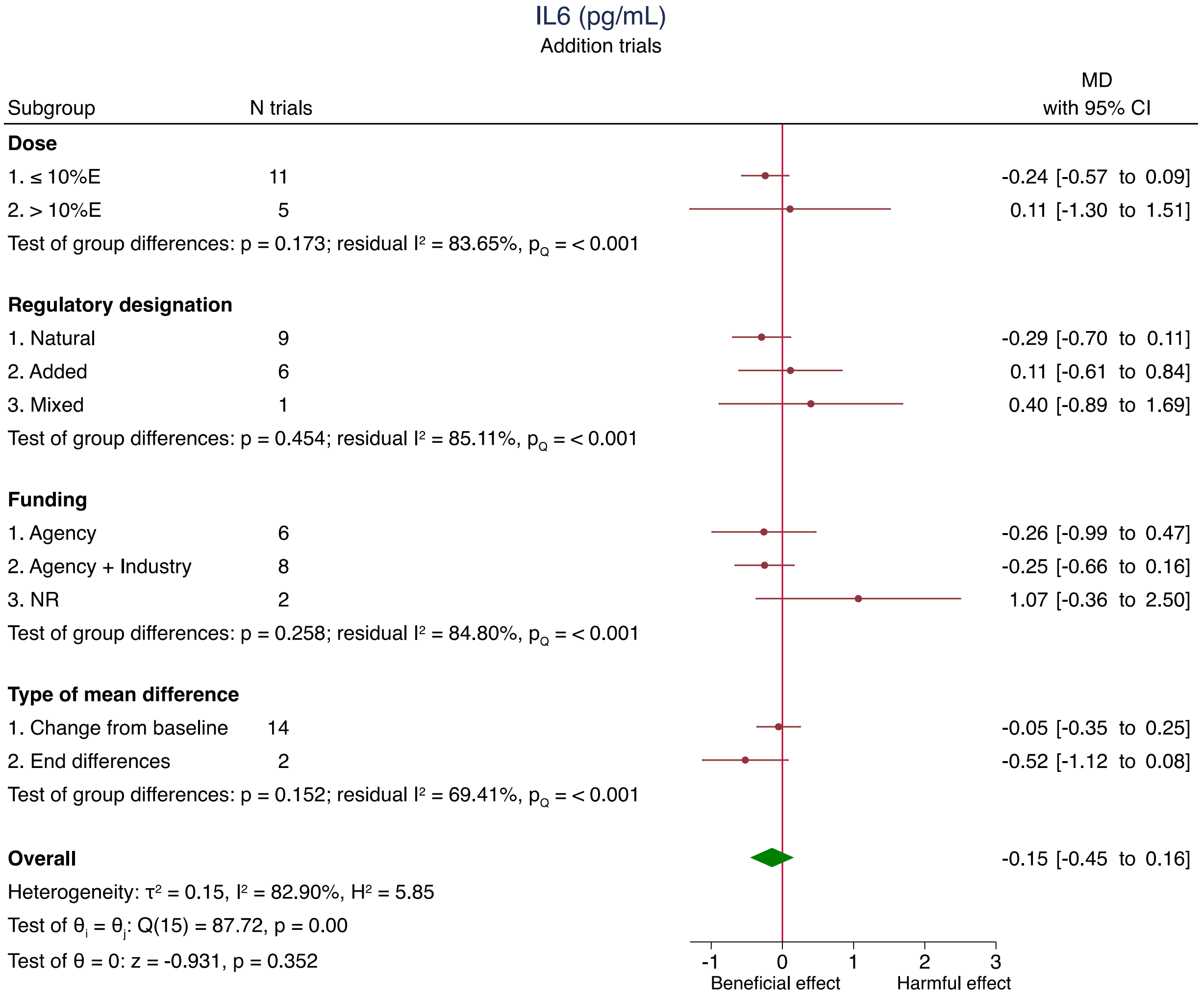

CI=confidence interval; E=energy; MD= mean difference; NR= not reported; IL-6=interleukin-6
The green diamond represents the pooled estimate for the overall primary analysis of food sources of fructose-containing sugars and CRP. Within subgroup mean differences are the pooled effect estimates represented by a red circle. 95% confidence intervals are represented by the line through the circle. Data are expressed as mean differences with 95% confidence intervals using the generic inverse-variance method and random effects DerSimonian-Laird model. Inter-study heterogeneity was assessed using the Cochrane Q statistic and quantified using the I^2 statistic, with significance set at P_Q<0·100 and I^2≥50% considered to be evidence of substantial heterogeneity. P<0·050 indicates that the effect size differed between levels of the subgroup.

Supplemental Figure S67: Risk of bias (using The Cochrane Collaboration Tool) subgroup analysis for the effect of important food sources of fructose-containing sugars on IL-6 (pg/mL) in addition trials

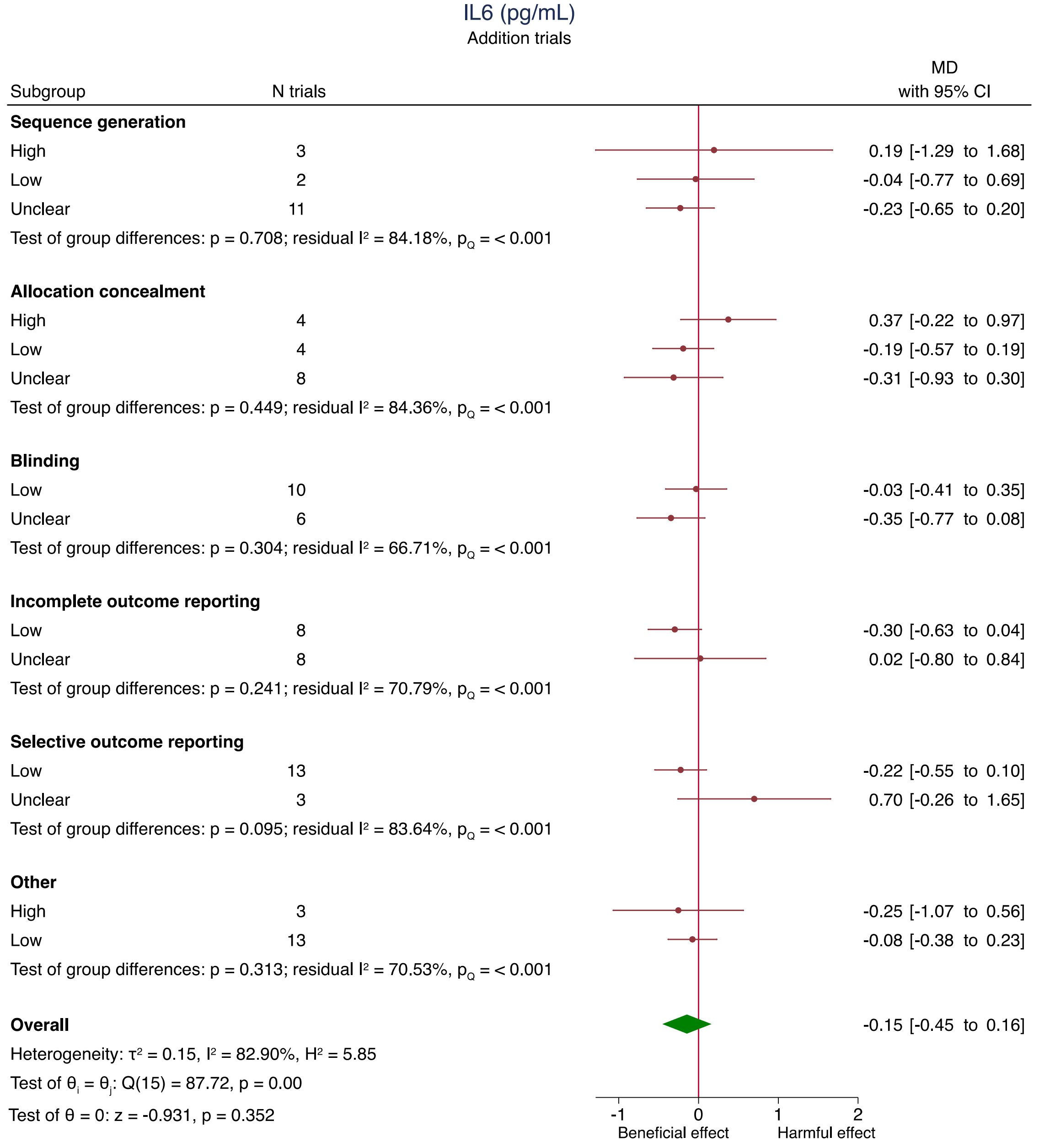

CI=confidence interval; MD= mean difference; IL-6=interleukin-6

The green diamond represents the pooled estimate for the overall primary analysis of food sources of fructose-containing sugars and CRP. Within subgroup mean differences are the pooled effect estimates represented by a red circle. 95% confidence intervals are represented by the line through the circle. Data are expressed as mean differences with 95% confidence intervals using the generic inverse-variance method and random effects DerSimonian-Laird model. Inter-study heterogeneity was assessed using the Cochrane Q statistic and quantified using the I^2 statistic, with significance set at $P_Q<0{\cdot}100$ and $I^2\geq50\%$ considered to be evidence of substantial heterogeneity. P<0·050 indicates that the effect size differed between levels of the subgroup.

Supplemental Figure S68: Continuous meta-regression analysis for the effect of important food sources of fructose-containing sugars on TNF-α (pg/mL) in substitution trials.

Continuous meta-regression
TNF-a (pg/mL) in substitution trials

Subgroup	Range	Trials	Beta [95% CI]	P_{MD}	Residual I^2 (%)	P_Q
Baseline TNF (pg/mL)*	1.000—6.800	14	-0.10 [-0.33 to 0.13]	0.515	28	0.174
Follow-up (weeks)	2.000—24.000	17	0.00 [-0.02 to 0.02]	0.113	31	0.117
Food source of fructose containing sugars dose (%E)	1.700—45.000	17	0.00 [-0.01 to 0.01]	0.162	26	0.167
Mean age (years)	23.500—70.212	17	0.00 [-0.01 to 0.01]	0.943	41	0.048

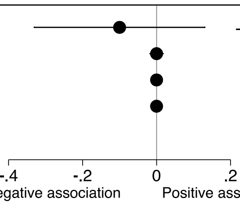

CI=confidence interval; %E=percentage of total energy intake; TNF-α= Tumour necrosis factor-alpha
Data is presented as between group mean difference (95% confidence intervals) for a 1-unit change in the predictor variable. ß–coefficients were estimated using continuous meta-regression analysis. A positive ß-coefficient implies an increase in TNF-α with the food source of fructose-containing sugars intervention as the subgroup variable increases, and a negative ß-coefficient implies a decrease in uric acid. Residual I^2 reports inter-study heterogeneity not explained by the subgroup and was estimated using the Cochrane Q statistic.
* N=3 trials missing data for baseline TNF-α

Supplemental Figure S69: Continuous meta-regression analysis for the effect of important food sources of fructose-containing sugars on TNF-α (pg/mL) in addition trials

Continuous meta-regression
TNF-a (pg/mL) in addition trials

Subgroup	Range	Trials	Beta [95% CI]	P_{MD}	Residual I^2 (%)	P_Q
Baseline TNF (pg/mL)*	1.2—29.2	13	-0.22 [-0.37 to -0.07]	0.004	88	<0.001
Follow-up (weeks)	1.0—24.0	16	0.00 [-0.10 to 0.10]	0.960	89	<0.001
Food source of fructose containing sugars dose (%E)	1.1—35.0	16	0.03 [-0.01 to 0.06]	0.089	86	<0.001
Mean age (years)	22.0—72.4	16	-0.04 [-0.06 to -0.01]	0.009	85	<0.001

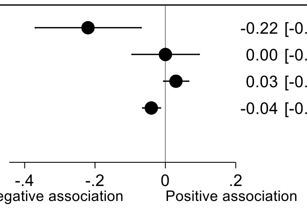

CI=confidence interval; %E=percentage of total energy intake; TNF-a= Tumour necrosis factor-alpha
Data is presented as between group mean difference (95% confidence intervals) for a 1-unit change in the predictor variable. ß–coefficients were estimated using continuous meta-regression analysis. A positive ß-coefficient implies an increase in TNF-α with the food source of fructose-containing sugars intervention as the subgroup variable increases, and a negative ß-coefficient implies a decrease in uric acid. Residual I^2 reports inter-study heterogeneity not explained by the subgroup and was estimated using the Cochrane Q statistic.
* N=4 trials missing data for baseline TNF-α

Supplemental Figure S70: Continuous meta-regression analysis for the effect of important food sources of fructose-containing sugars on IL-6 (pg/mL) in substitution trials

Continuous meta-regression
IL-6 (pg/mL) in substitution trials

Subgroup	Range	Trials	Beta [95% CI]	P_{MD}	Residual I^2 (%)	P_Q
Baseline IL6 (pg/mL)*	0.830—27.400	13	0.00 [-0.13 to 0.13]	0.558	9	0.354
Follow-up (weeks)	1.000—24.000	16	0.00 [-0.02 to 0.02]	0.970	25	0.173
Food source of fructose containing sugars dose (%E)	1.700—45.000	16	0.00 [-0.01 to 0.01]	0.540	27	0.158
Mean age (years)	23.500—70.212	16	0.00 [-0.01 to 0.01]	0.099	15	0.290

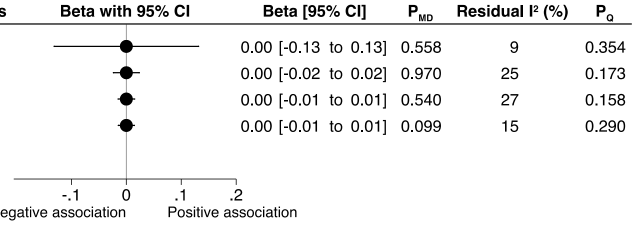

CI=confidence interval; %E=percentage of total energy intake; IL-6= interleukin-6
Data is presented as between group mean difference (95% confidence intervals) for a 1-unit change in the predictor variable. ß–coefficients were estimated using continuous meta-regression analysis. A positive ß-coefficient implies an increase in IL6 with the food source of fructose-containing sugars intervention as the subgroup variable increases, and a negative ß-coefficient implies a decrease in uric acid. Residual I^2 reports inter-study heterogeneity not explained by the subgroup and was estimated using the Cochrane Q statistic.
* N=3 trials missing data for baseline IL-6

Supplemental Figure S71: Continuous meta-regression analysis for the effect of important food sources of fructose-containing sugars on IL-6 (pg/mL) in addition comparisons

Continuous meta-regression
IL6 (pg/mL) in addition trials

Subgroup	Range	Trials	Beta [95% CI]	P_{MD}	Residual I^2 (%)	P_Q
Baseline IL6 (pg/mL)*	0.6—16.4	14	-0.23 [-0.55 to 0.09]	0.158	74	<0.001
Follow-up (weeks)	1.0—24.0	16	0.02 [-0.07 to 0.11]	0.662	80	<0.001
Food source of fructose containing sugars dose (%E)	1.1—35.0	16	0.03 [-0.00 to 0.06]	0.116	82	<0.001
Mean age (years)	22.0—53.2	16	-0.04 [-0.09 to 0.01]	0.058	84	<0.001

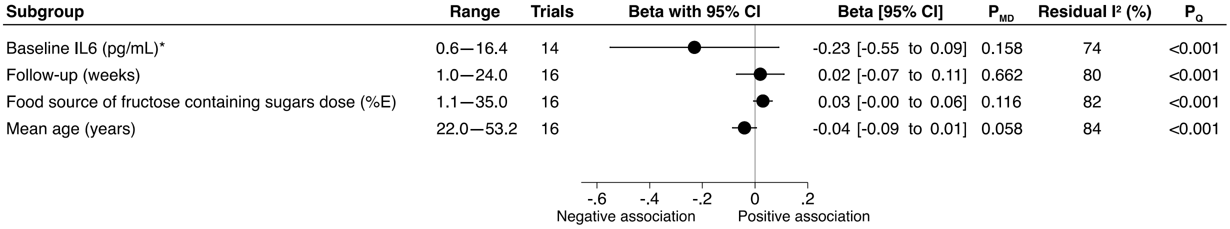

CI=confidence interval; %E=percentage of total energy intake; IL-6= interleukin-6
Data is presented as between group mean difference (95% confidence intervals) for a 1-unit change in the predictor variable. ß–coefficients were estimated using continuous meta-regression analysis. A positive ß-coefficient implies an increase in IL-6 with the food source of fructose-containing sugars intervention as the subgroup variable increases, and a negative ß-coefficient implies a decrease in uric acid. Residual I^2 reports inter-study heterogeneity not explained by the subgroup and was estimated using the Cochrane Q statistic.
* N=2 trials missing data for baseline IL-6

Supplemental Figure S72: Linear and non-linear meta-regression analyses for effect of important food sources of fructose-containing sugars on CRP (mg/L) in substitution trials

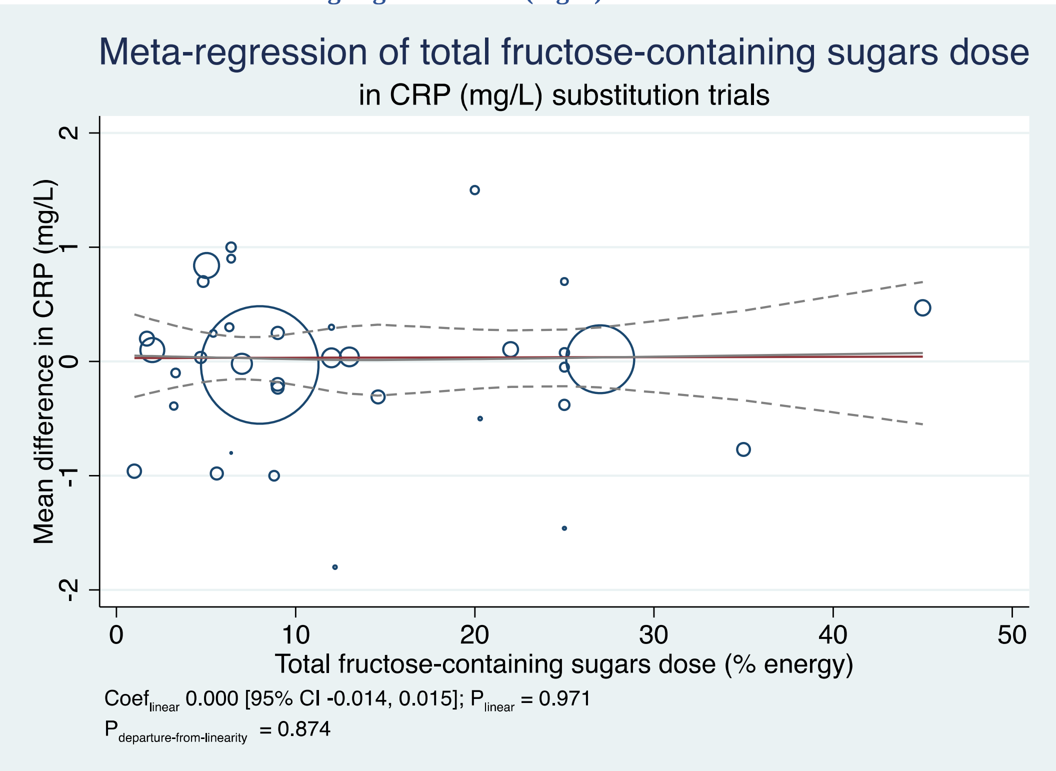

CI=confidence interval; coef=coefficient; E=energy; CRP= C reactive protein
Individual trials are represented by the circles, with their weight in the overall analysis represented by the size of the circles. The straight line represents the estimate dose response for amount of fructose-containing sugars consumed (% of total energy intake) and the dashed lines represent the upper and lower 95% confidence intervals.

Supplemental Figure S73: Linear and non-linear meta-regression analysis for the effect of important food sources of fructose-containing sugars on CRP (mg/L) in addition comparisons

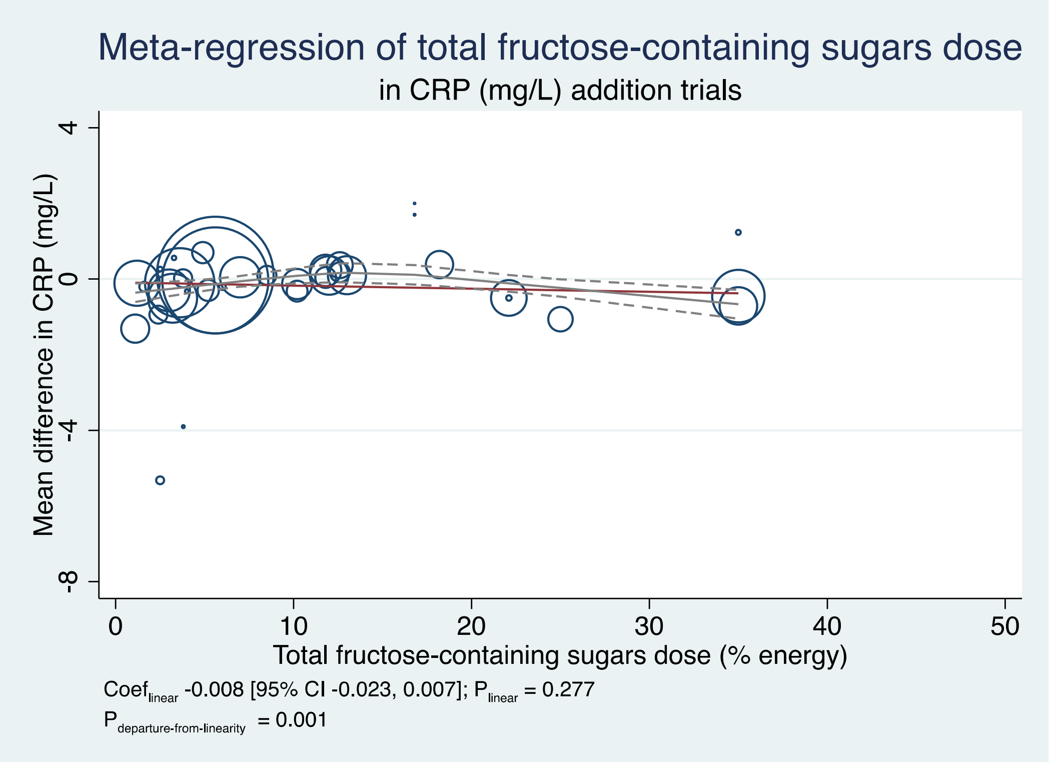

CI=confidence interval; coef=coefficient; E=energy; CRP= C reactive protein
Individual trials are represented by the circles, with their weight in the overall analysis represented by the size of the circles. The straight line represents the estimate dose response for amount of fructose-containing sugars consumed (% of total energy intake) and the dashed lines represent the upper and lower 95% confidence intervals.

Supplemental Figure S74: Linear and non-linear meta-regression analyses for the effect of individual food sources of fructose-containing sugars dose on CRP (mg/L) in substitution trials

A:

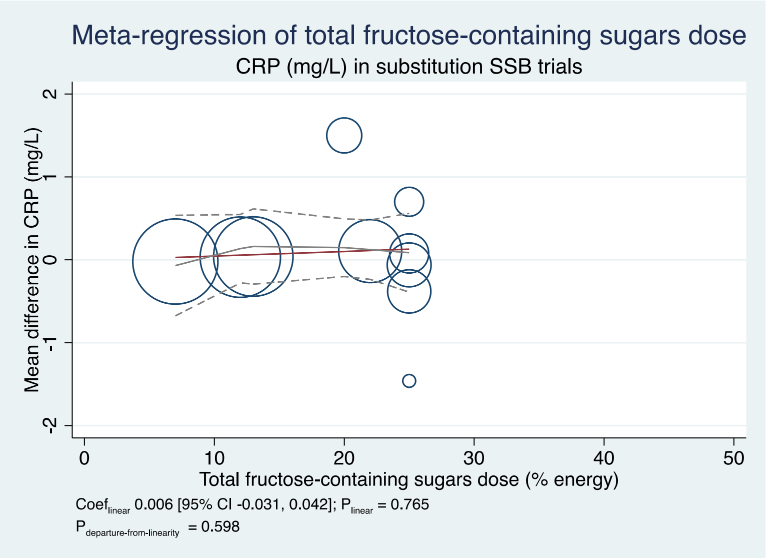

CI=confidence interval; coef=coefficient; CRP = C reactive protein; SSB= sugar sweetened beverage
Individual trials are represented by the circles, with their weight in the overall analysis represented by the size of the circles. The straight line represents the estimate dose response for amount of fructose-containing sugars consumed (% of total energy intake) and the dashed lines represent the upper and lower 95% confidence intervals.
Panel A= SSB
Linear and non-linear dose response analyses were not possible for sweetened dairy; sweetened dairy alternatives (soy); 100% fruit juice; fruit; dried fruit; mixed fruit forms; added nutritive (caloric) sweetener; mixed sources (with SSBs); and mixed sources (no SSBs) as there were fewer than six trial comparisons with dose data available.

Supplemental Figure S75: Linear and non-linear meta-regression analyses for the effect of individual food sources of fructose-containing sugars dose on CRP (mg/L) in addition trials

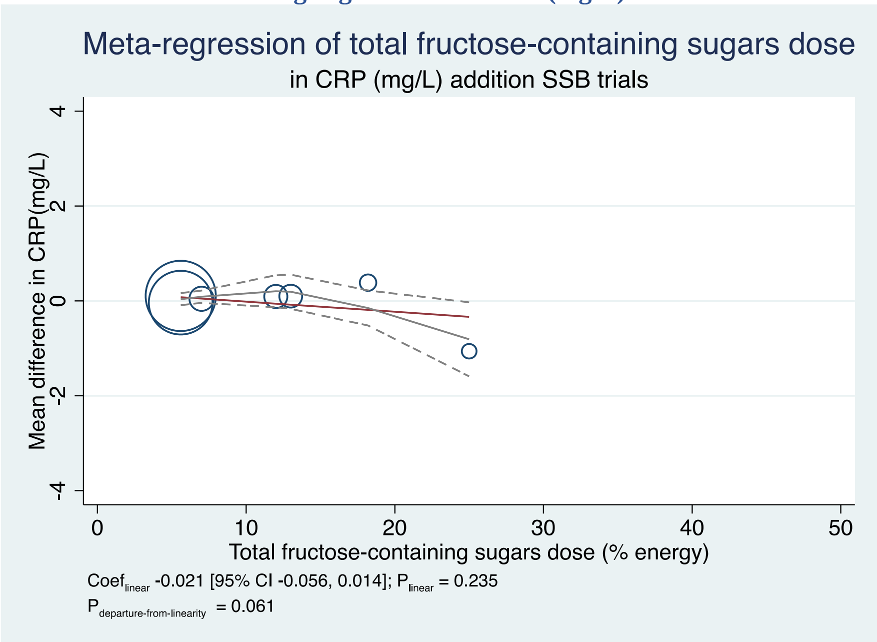

A:

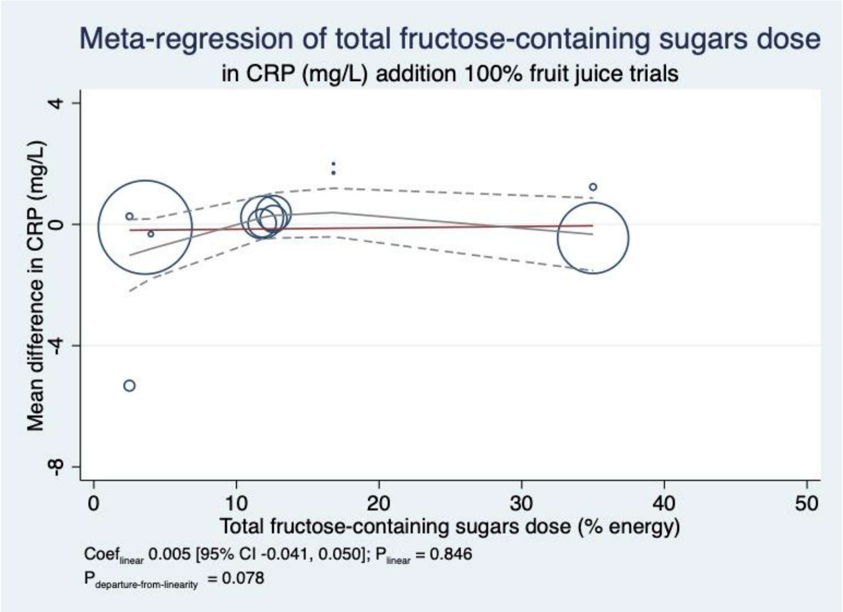

B:

C: 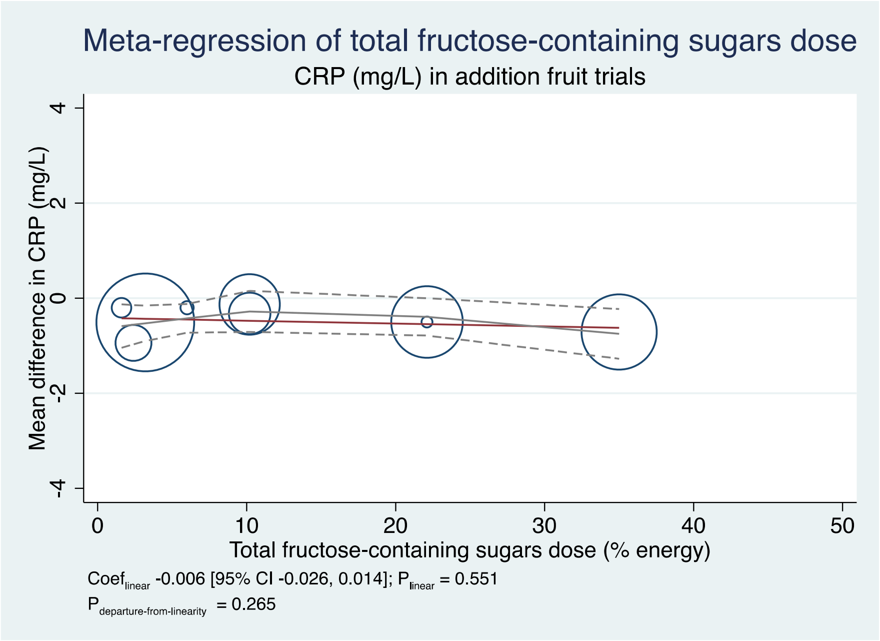

CI=confidence interval; coef=coefficient; CRP = C reactive protein; SSB= sugar sweetened beverage
Individual trials are represented by the circles, with their weight in the overall analysis represented by the size of the circles. The straight line represents the estimate dose response for amount of fructose-containing sugars consumed (% of total energy intake) and the dashed lines represent the upper and lower 95% confidence intervals.
Panel A= SSB; B= 100% fruit juice; C= fruit
Linear and non-linear dose response analyses were not possible for sweetened dairy alternatives (soy); dried fruit; mixed fruit forms; sweetened cereal grains and bars; sweets and desserts; added nutritive (caloric) sweetener as there were fewer than six trial comparisons with dose data available.

Supplemental Figure S76: Non-linear dose-response analysis using public thresholds of 5% (panel A), 10% (panel B), and 25% (panel C) of energy for the effect of total food sources of fructose-containing sugars on CRP (mg/L) in substitution trials

A:

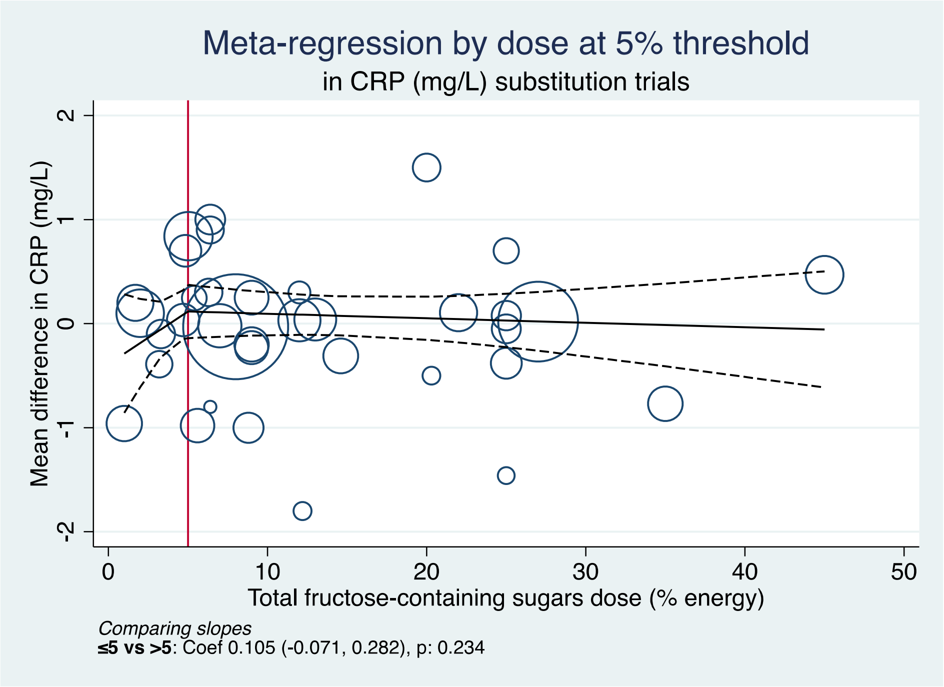

B:

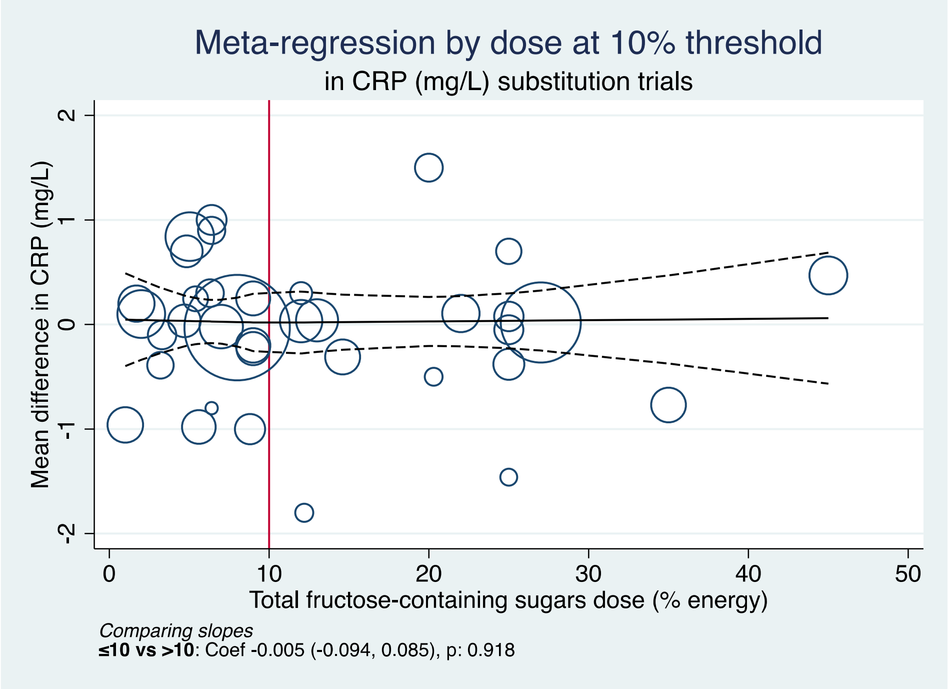

C:

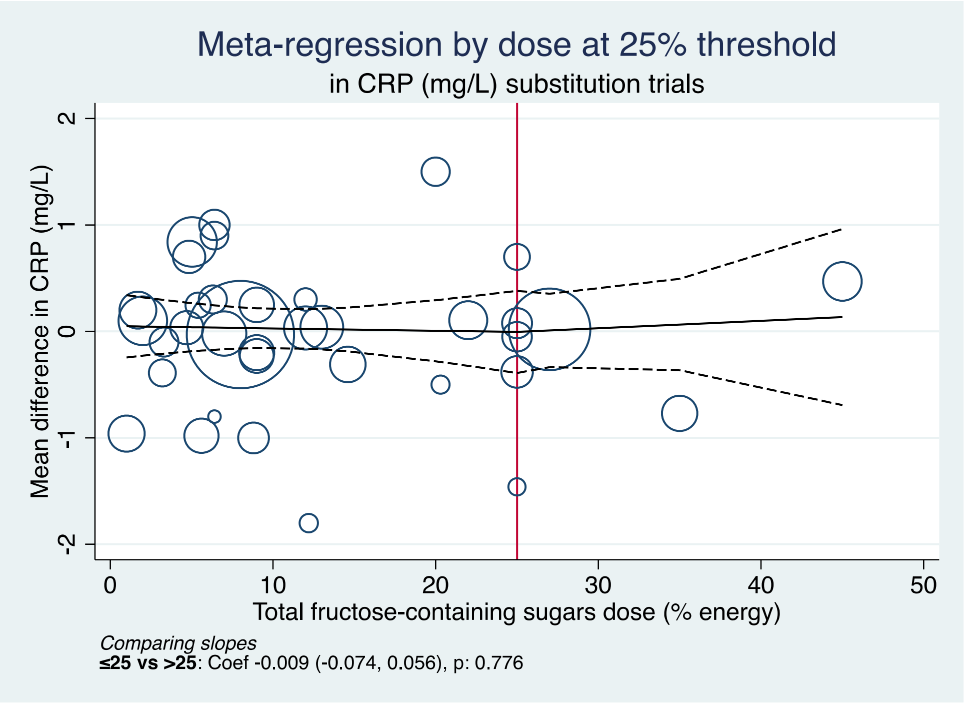

Coef=coefficient; %E=percentage of total energy intake; CRP= C reactive protein; p-val= p-value
Individual trials are represented by the circles, with their weight in the overall analysis represented by the size of the circles. The straight line represents the estimate dose response for amount of fructose-containing sugars consumed (% of total energy intake) and the dashed lines represent the upper and lower 95% confidence intervals.
Panel A: 5% threshold; B: 10% threshold; C: 25% threshold.

Supplemental Figure S77: Non-linear dose-response analysis using public threshold of of 5% (panel A), 10% (panel B), and 25% (panel C) energy for the effect of important food sources of fructose-containing sugars on CRP (mg/L) in addition trials

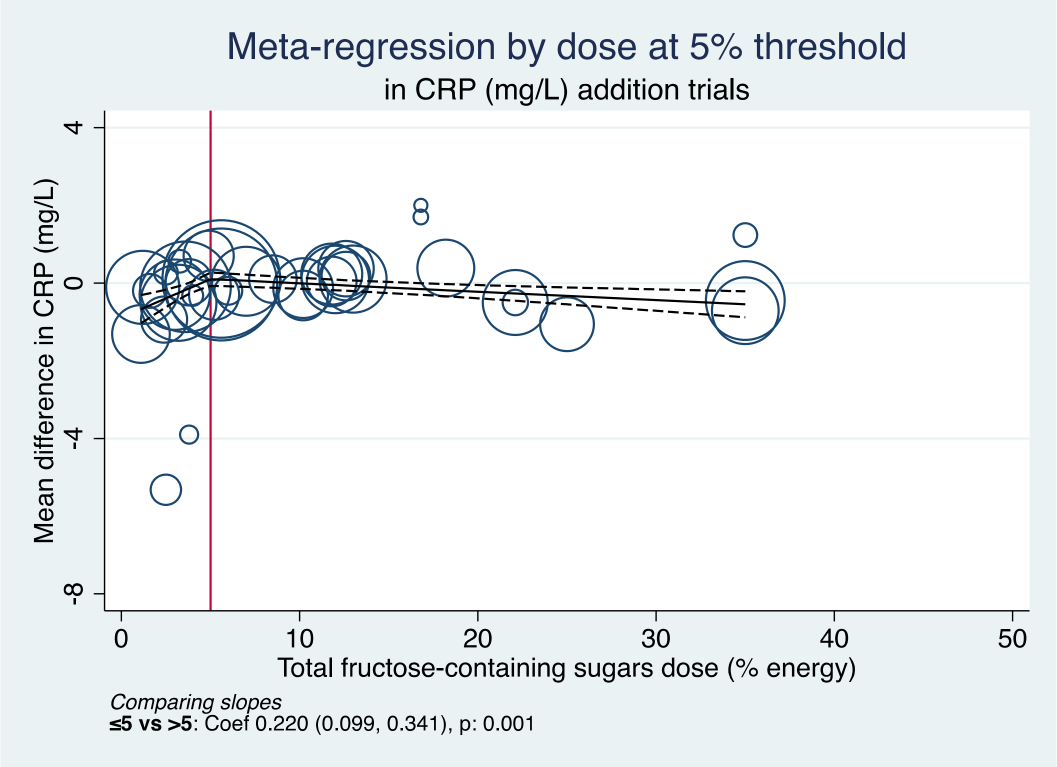

A:

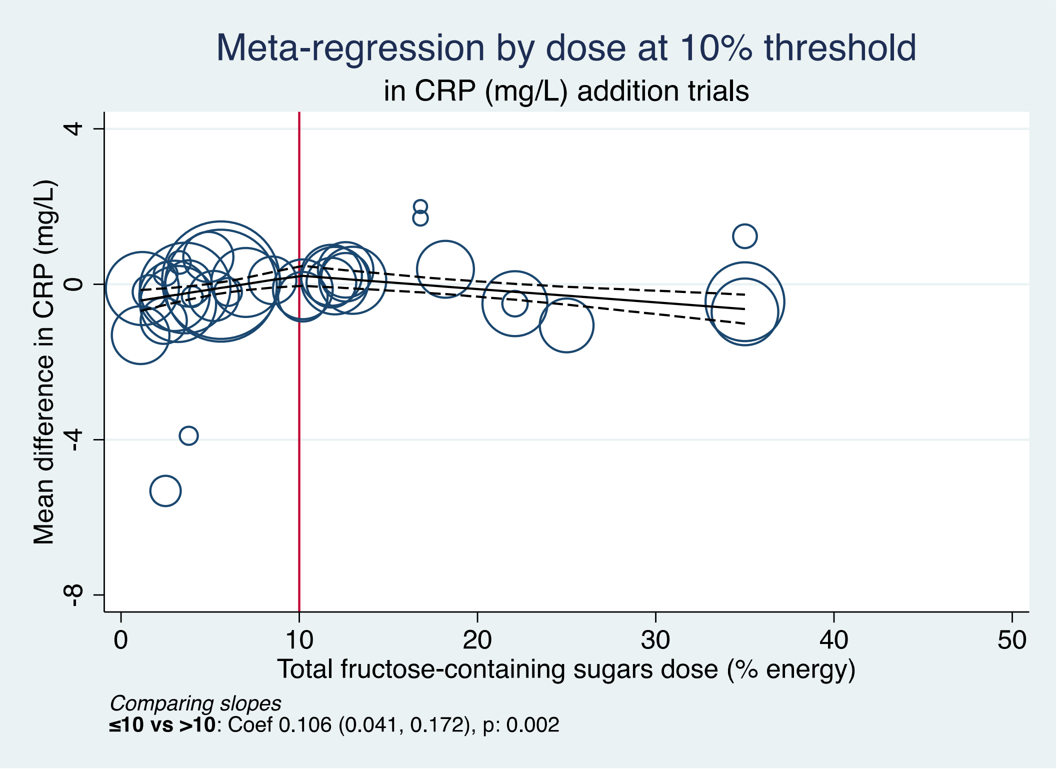

B:

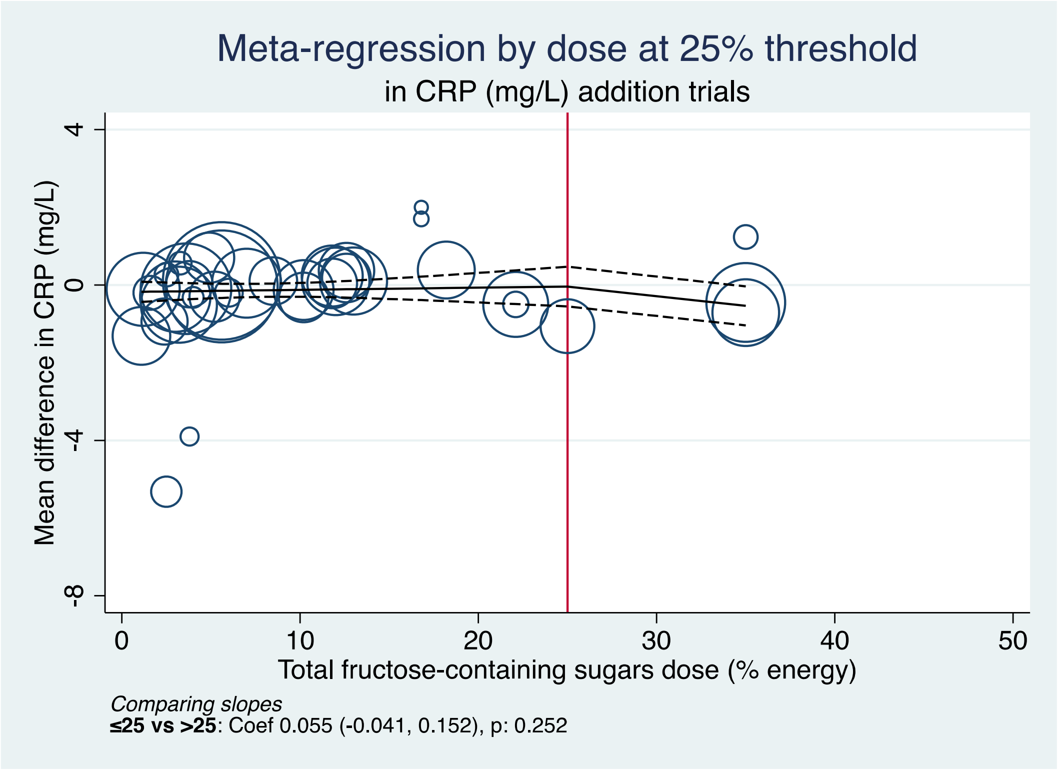

C:

Coef=coefficient; %E=percentage of total energy intake; CRP= C reactive protein; p-val= p-value
Individual trials are represented by the circles, with their weight in the overall analysis represented by the size of the circles. The straight line represents the estimate dose response for amount of fructose-containing sugars consumed (% of total energy intake) and the dashed lines represent the upper and lower 95% confidence intervals.
Panel A: 5% threshold; B: 10% threshold; C: 25% threshold.

Supplemental Figure S78: Non-linear dose-response analysis using public thresholds of 10% (panel A) of energy for the effect of the effect of SSBs on CRP (mg/L) in substitution trials

A: 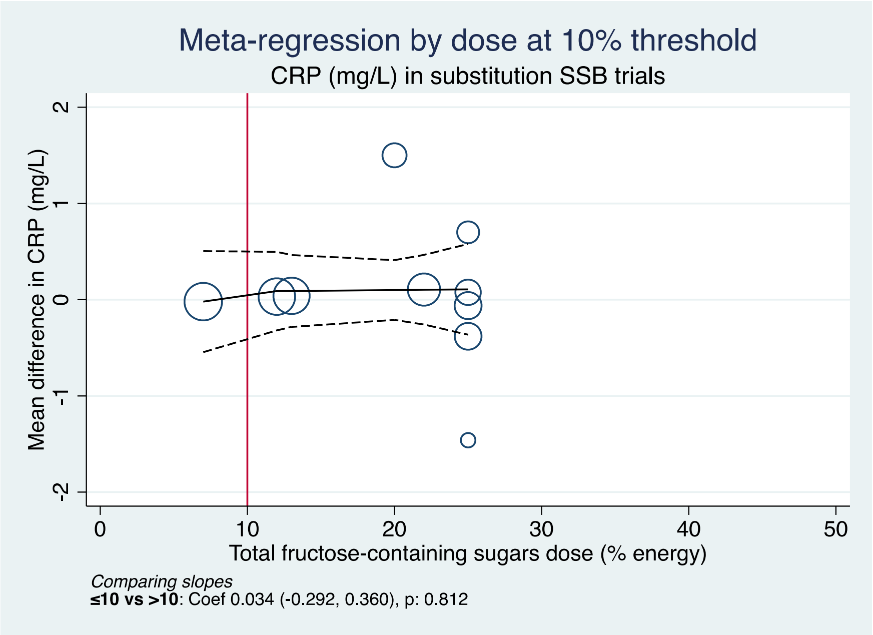

Coef=coefficient; CRP= C reactive protein; %E=percentage of total energy intake; p-val=p-value; SSB=sugar-sweetened beverage
Individual trials are represented by the circles, with their weight in the overall analysis represented by the size of the circles. The straight line represents the estimate dose response for amount of fructose-containing sugars consumed (% of total energy intake) and the dashed lines represent the upper and lower 95% confidence intervals.
Panel A: 10% threshold
No trial comparisons with a dose for less than 5% nor greater than 25% were available

Supplemental Figure S79: Non-linear dose-response analysis using public thresholds of 5%, 10% , and 25% of energy for the effect of the effect of SSBs on CRP (mg/L) in addition trials

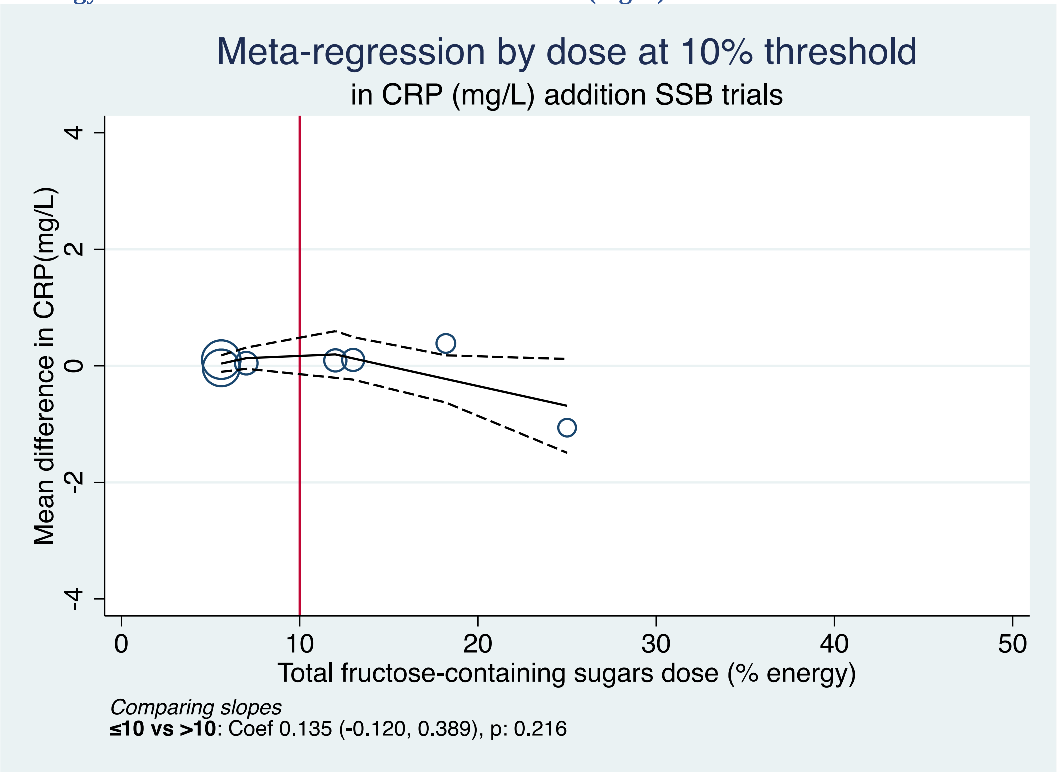

Coef=coefficient; CRP= C reactive protein; %E=percentage of total energy intake; p-val=p-value; SSB=sugar-sweetened beverage

Individual trials are represented by the circles, with their weight in the overall analysis represented by the size of the circles. The straight line represents the estimate dose response for amount of fructose-containing sugars consumed (% of total energy intake) and the dashed lines represent the upper and lower 95% confidence intervals.

Panel A: 10% threshold

No trial comparisons with a dose for less than 5% nor greater than 25% were available

Supplemental Figure S80: Non-linear dose-response analysis using public thresholds of 5%, 10% , and 25% of energy for the effect of 100% fruit juice on CRP (mg/L) in addition trials

A:

B:

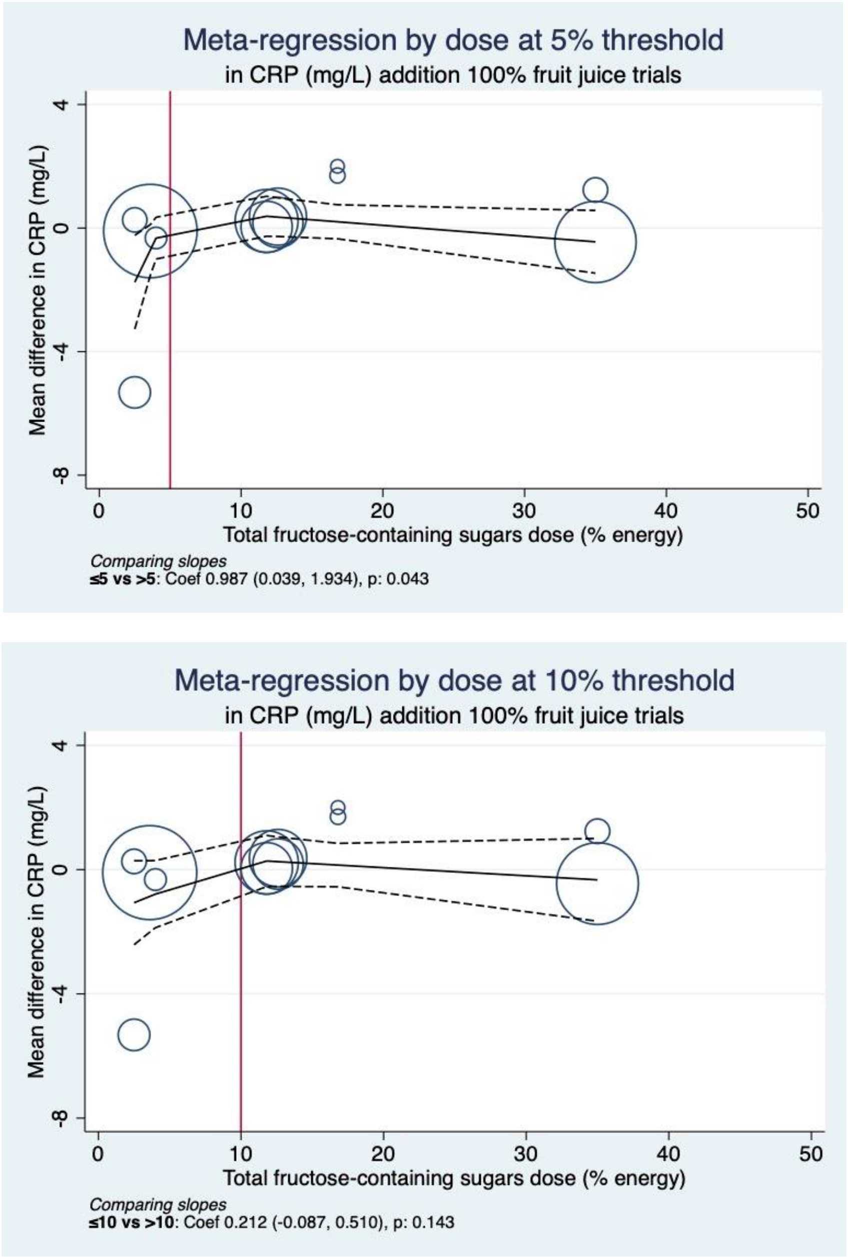

C: 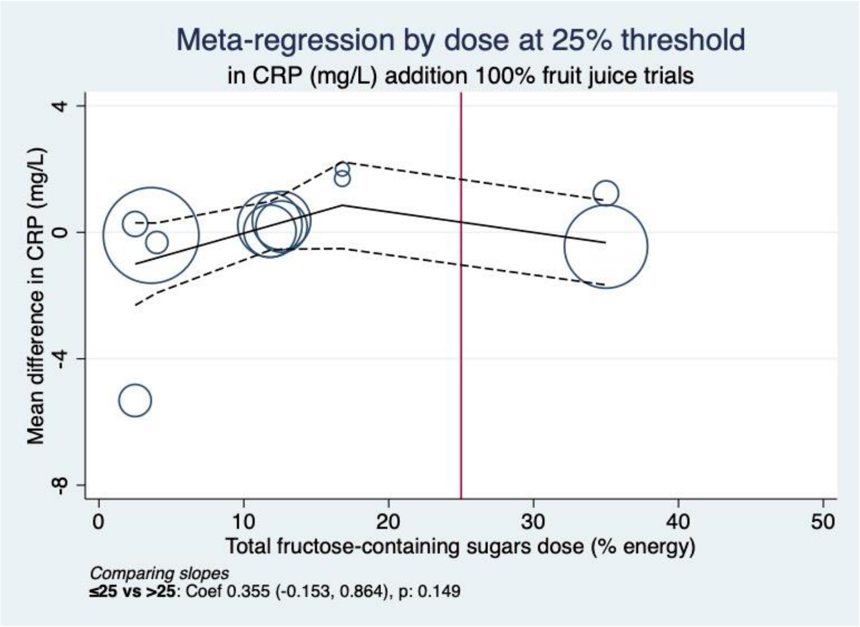

Coef=coefficient; CRP= C reactive protein; %E=percentage of total energy intake; p-val=p-value
Individual trials are represented by the circles, with their weight in the overall analysis represented by the size of the circles. The straight line represents the estimate dose response for amount of fructose-containing sugars consumed (% of total energy intake) and the dashed lines represent the upper and lower 95% confidence intervals.
Panel A: A= 5% threshold for 100% fruit juice trials; B = 10% threshold for 100% fruit juice trials; C= 25% threshold for 100% fruit juice trials

Supplemental Figure S81: Non-linear dose-response analysis using public thresholds of 5%, 10%, and 25% of energy for the effect of fruit on CRP (mg/L) in addition trials

A: 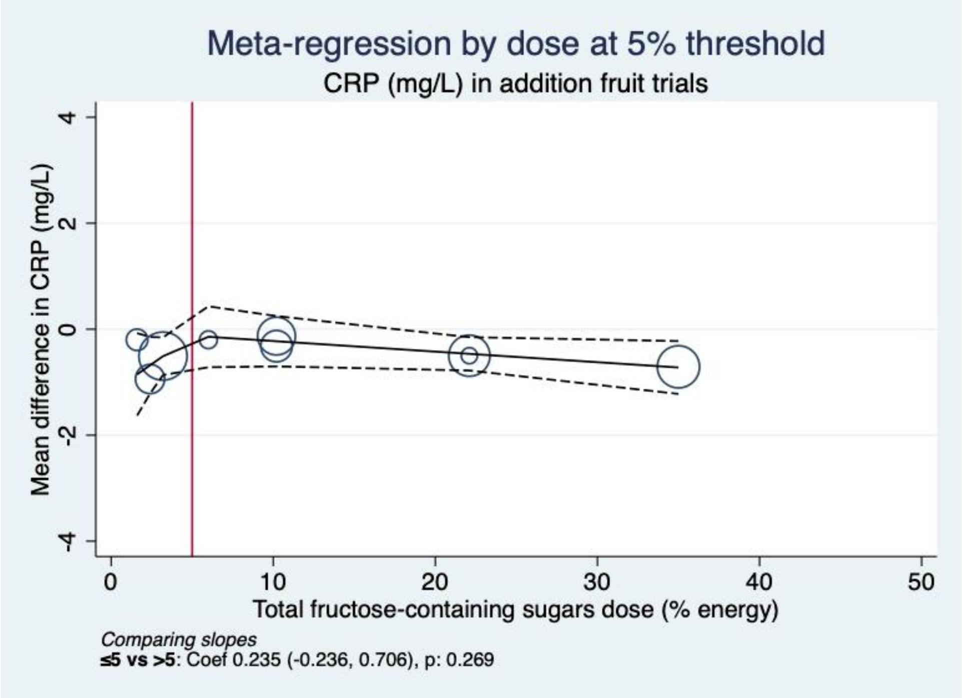

B: 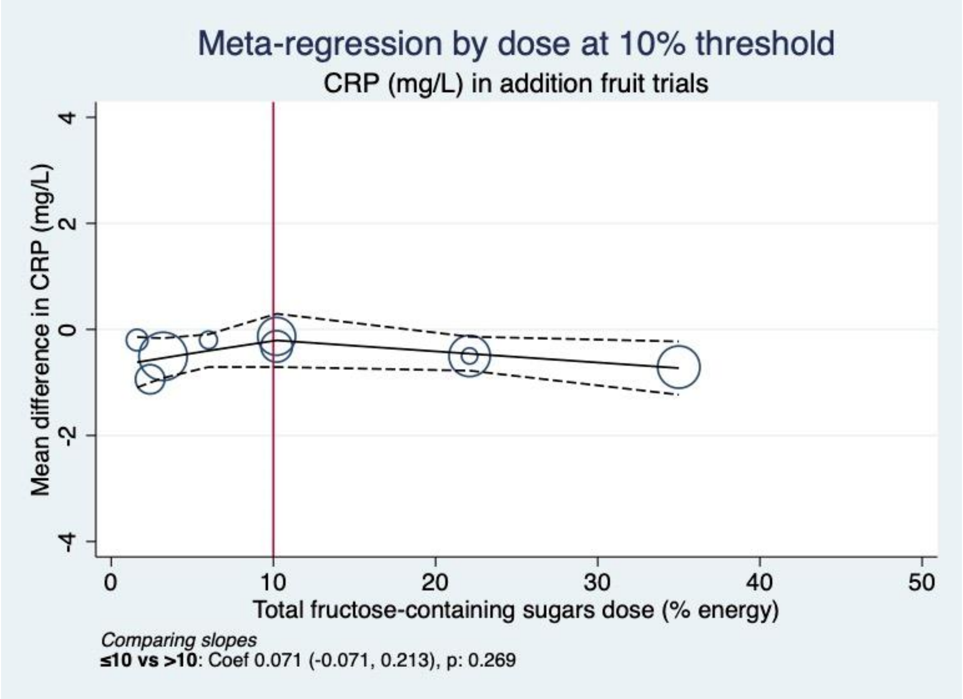

C: 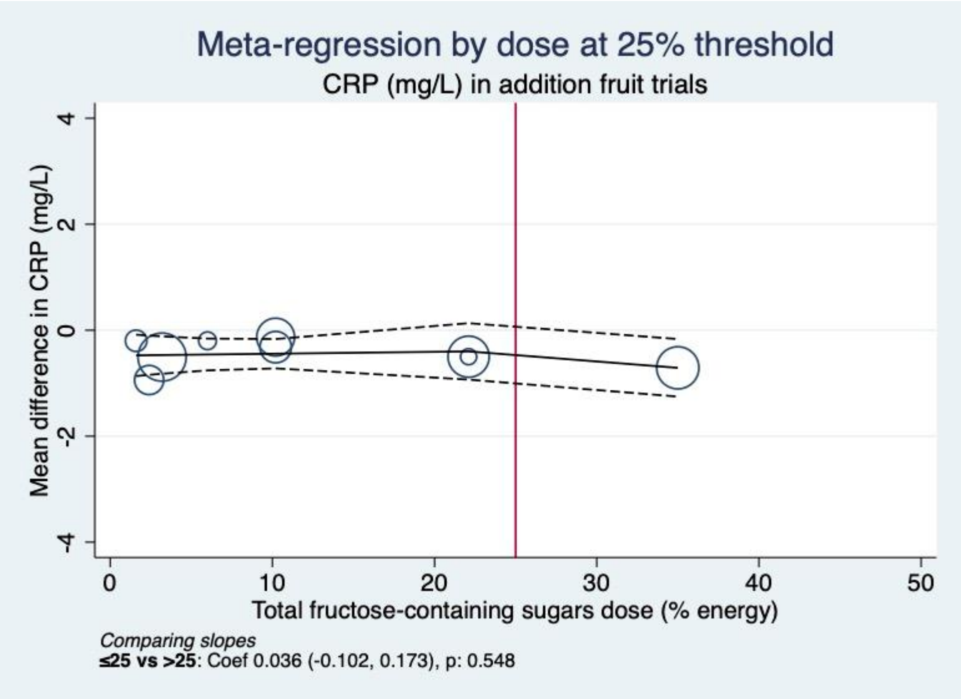

Coef=coefficient; CRP= C reactive protein; %E=percentage of total energy intake; p-val=p-value
Individual trials are represented by the circles, with their weight in the overall analysis represented by the size of the circles. The straight line represents the estimate dose response for amount of fructose-containing sugars consumed (% of total energy intake) and the dashed lines represent the upper and lower 95% confidence intervals.
Panel A: 5% threshold for fruit trials; B= 10% threshold for fruit trials; C = 25% threshold for fruit trials

Supplemental Figure S82: Linear and non-linear meta-regression analyses for the effect of important food sources of fructose-containing sugars on TNF-α (pg/mL) in substitution trials.

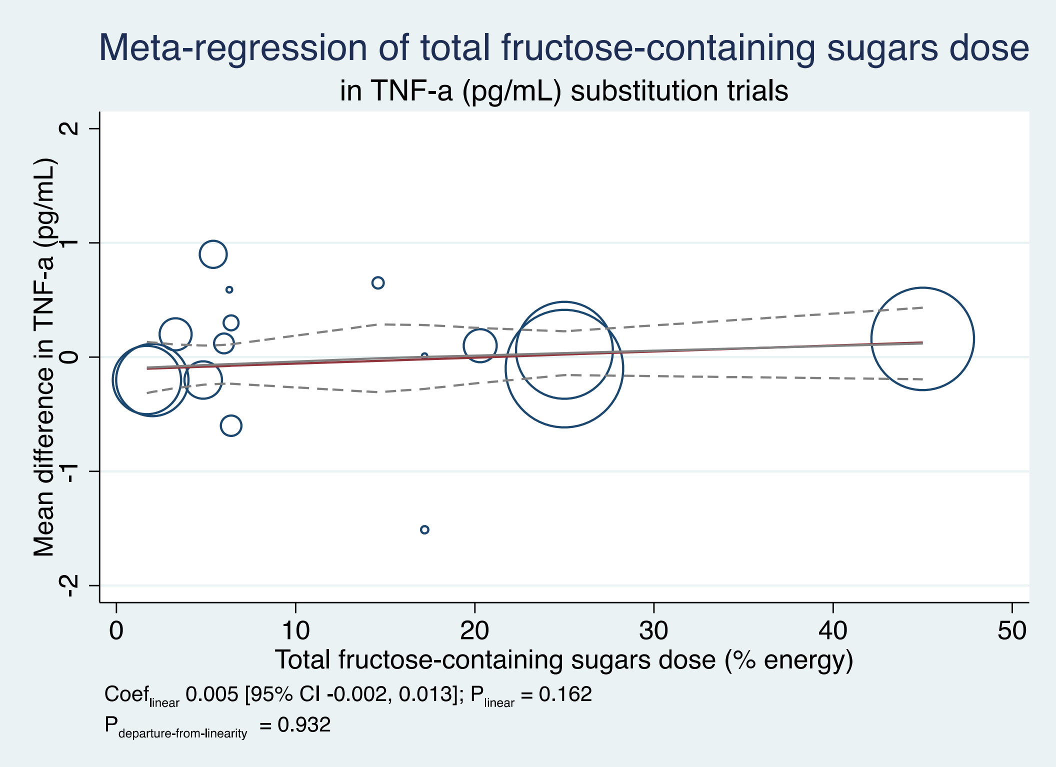

CI=confidence interval; coef=coefficient; E=energy; TNF-a= tumour necrosis factor-alpha
Individual trials are represented by the circles, with their weight in the overall analysis represented by the size of the circles. The straight line represents the estimate dose response for amount of fructose-containing sugars consumed (% of total energy intake) and the dashed lines represent the upper and lower 95% confidence intervals.

Supplemental Figure S83: Linear and non-linear meta-regression analyses for the effect of important food sources of fructose-containing sugars on TNF-α (pg/mL) in addition trials

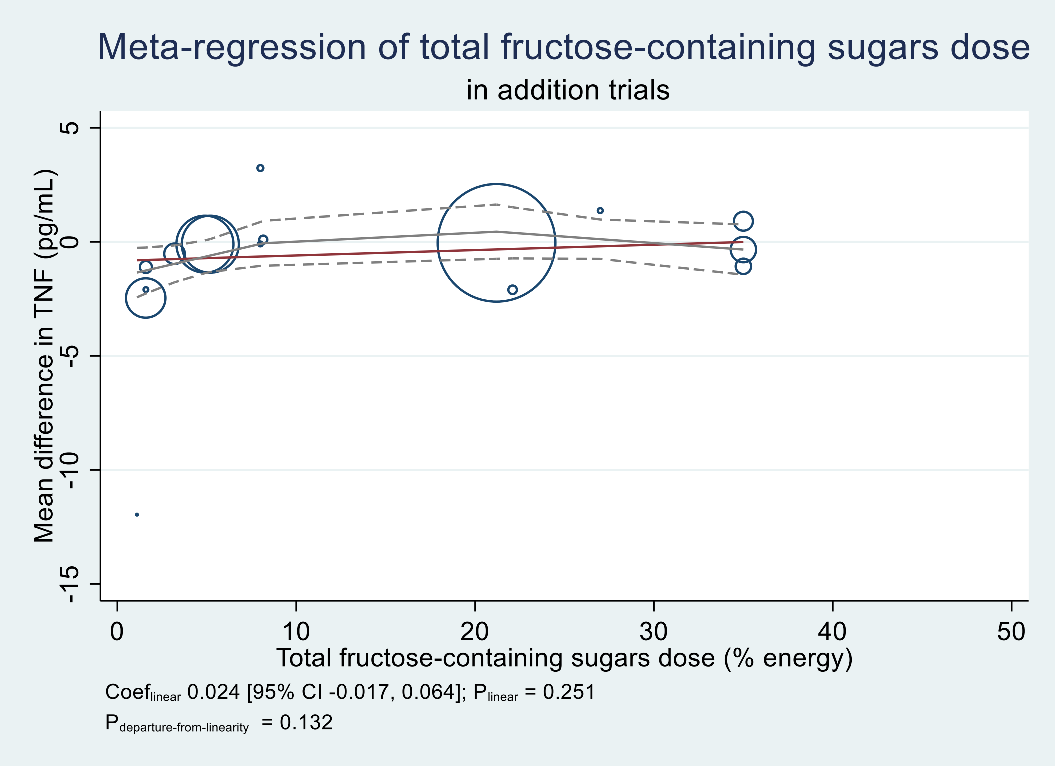

CI=confidence interval; coef=coefficient; E=energy; TNF-α= tumour necrosis factor-alpha
Individual trials are represented by the circles, with their weight in the overall analysis represented by the size of the circles. The straight line represents the estimate dose response for amount of fructose-containing sugars consumed (% of total energy intake) and the dashed lines represent the upper and lower 95% confidence intervals.

Supplemental Figure S84: Linear and non-linear meta-regression analyses for the effect of important food sources of fructose-containing sugars on IL-6 (pg/mL) in substitution trials.

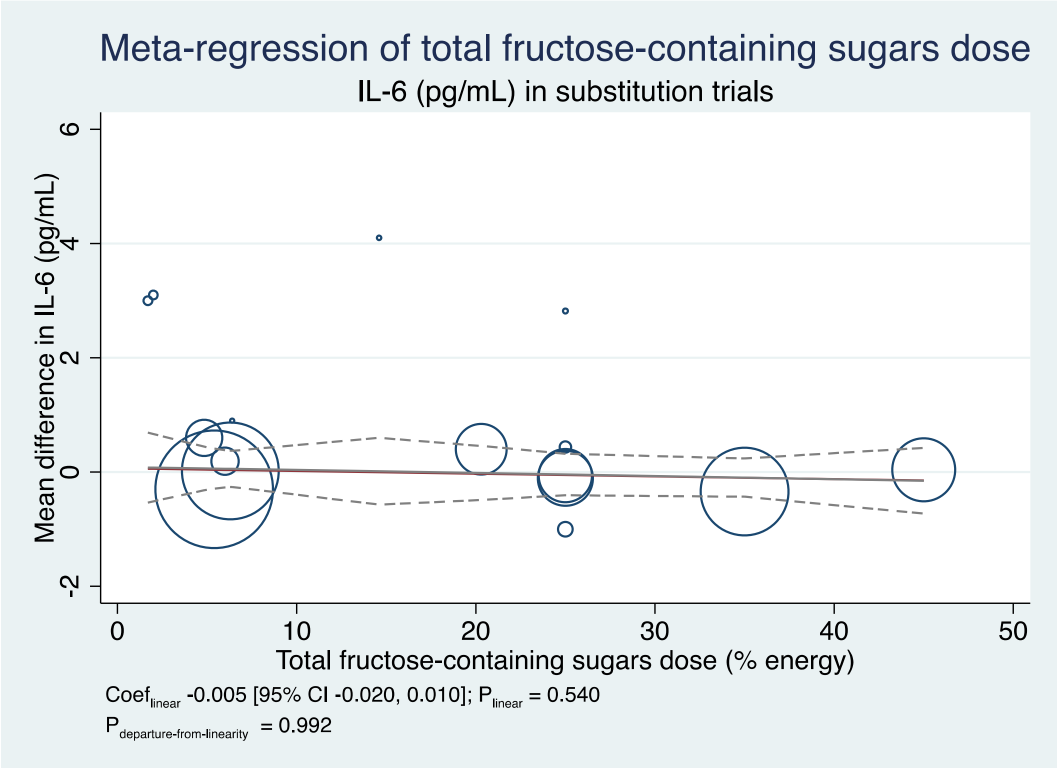

CI=confidence interval; coef=coefficient; E=energy; IL-6= interleukin-6
Individual trials are represented by the circles, with their weight in the overall analysis represented by the size of the circles. The straight line represents the estimate dose response for amount of fructose-containing sugars consumed (% of total energy intake) and the dashed lines represent the upper and lower 95% confidence intervals.

Supplemental Figure S85: Linear and non-linear meta-regression analyses for the effect of important food sources of fructose-containing sugars on IL-6 (pg/mL) in addition trials

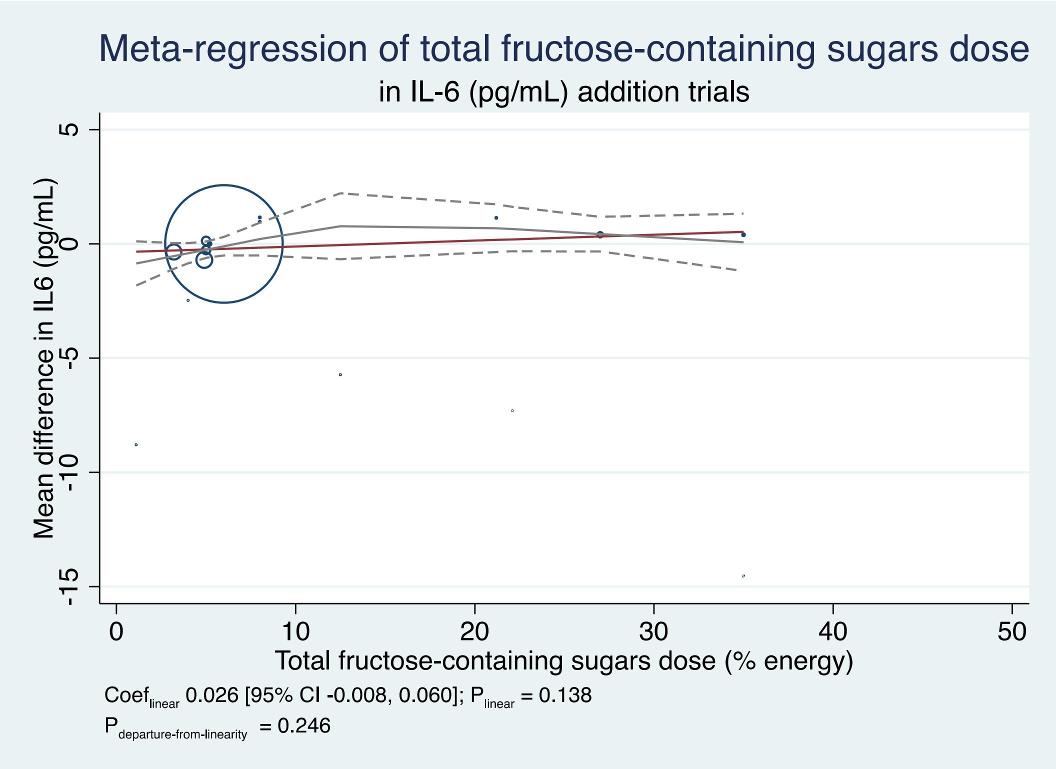

CI=confidence interval; coef=coefficient; E=energy; IL-6=interleukin-6
Individual trials are represented by the circles, with their weight in the overall analysis represented by the size of the circles. The straight line represents the estimate dose response for amount of fructose-containing sugars consumed (% of total energy intake) and the dashed lines represent the upper and lower 95% confidence intervals.

Supplemental Figure S86: Non-linear dose-response analysis using public thresholds of 5% (panel A), 10% (panel B), and 25% (panel C) of energy for the effect of important food sources of fructose-containing sugars on TNF-α (pg/mL) in substitution trials

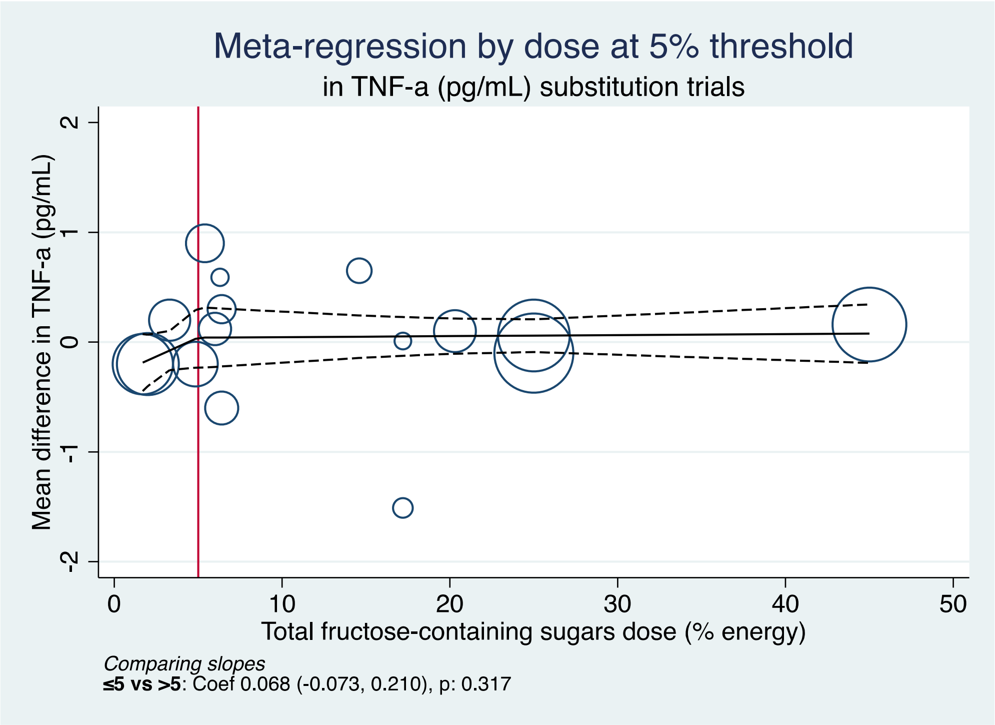

A:

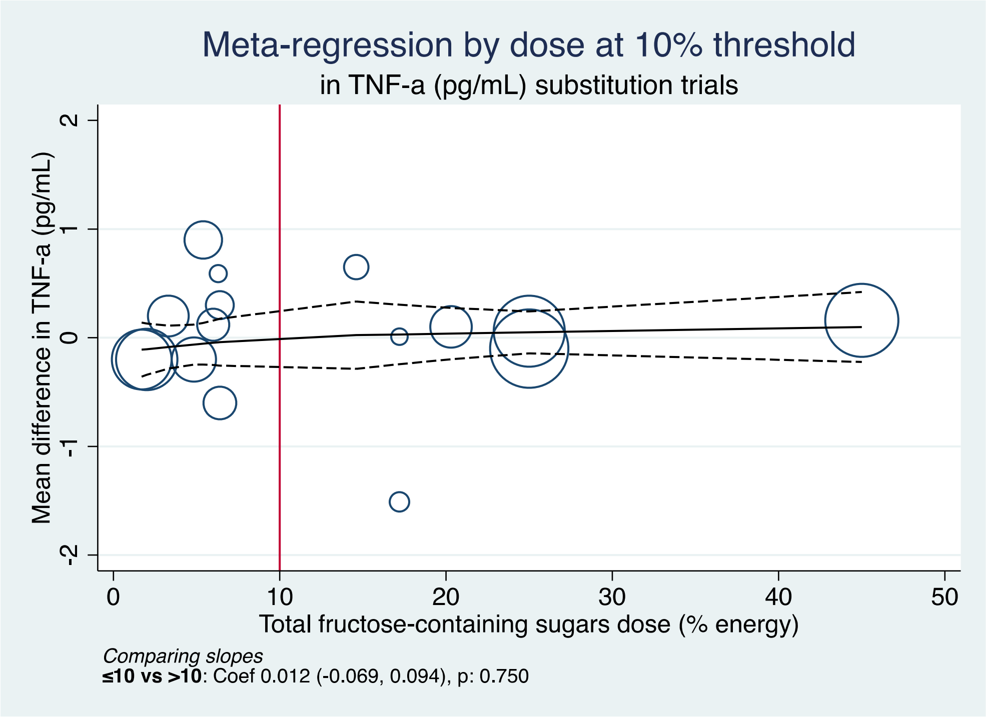

B:

C:

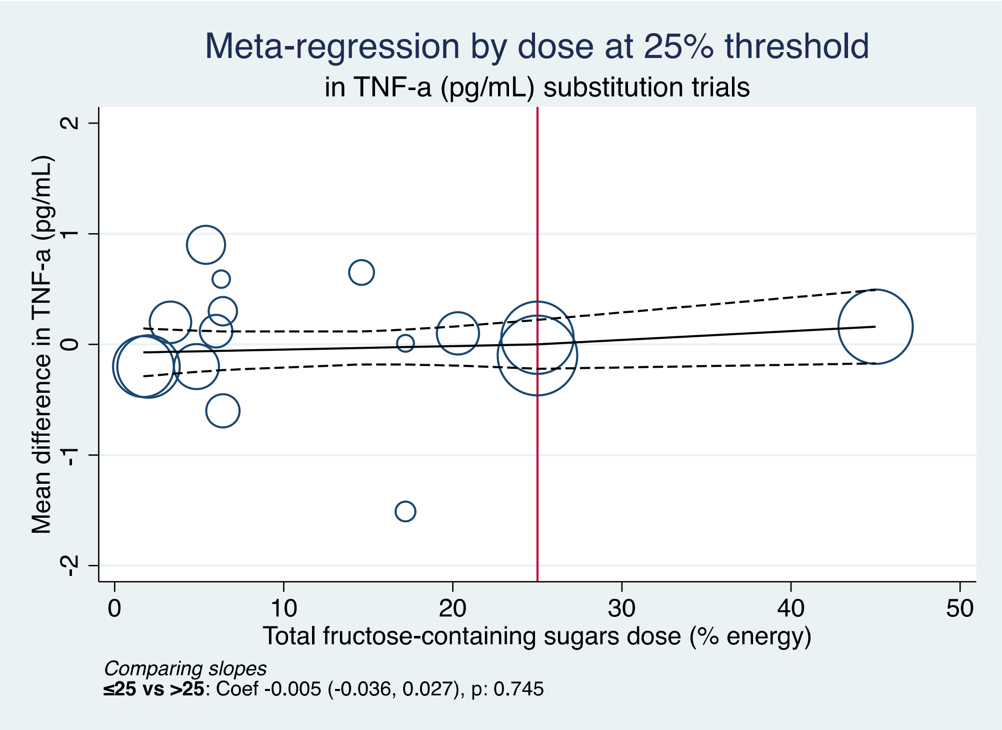

Coef=coefficient; %E=percentage of total energy intake; TNF-a= tumour necrosis factor-alpha; p-val= p-value
Individual trials are represented by the circles, with their weight in the overall analysis represented by the size of the circles. The straight line represents the estimate dose response for amount of fructose-containing sugars consumed (% of total energy intake) and the dashed lines represent the upper and lower 95% confidence intervals.
Panel A: 5% threshold; B: 10% threshold; C: 25% threshold.

Supplemental Figure S87: Non-linear dose-response analysis using public thresholds of 5% (panel A), 10% (panel B), and 25% (panel C) of energy for the effect of important food sources of fructose-containing sugars on TNF-α (pg/mL) in addition trials

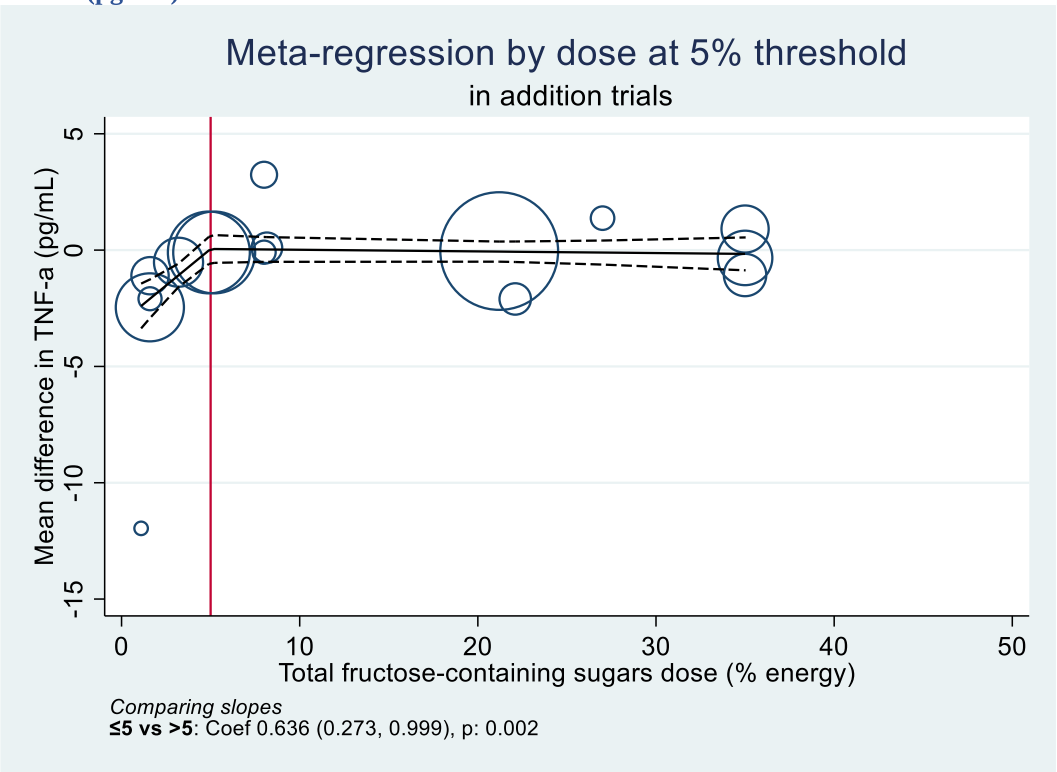

A:

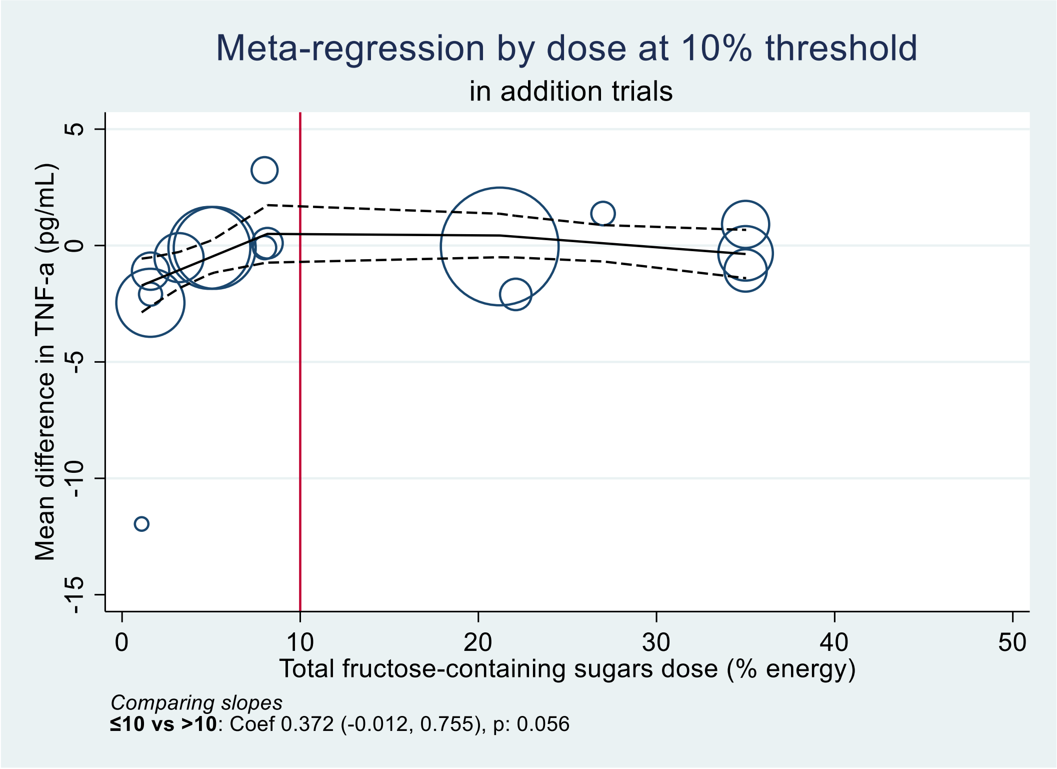

B:

C: 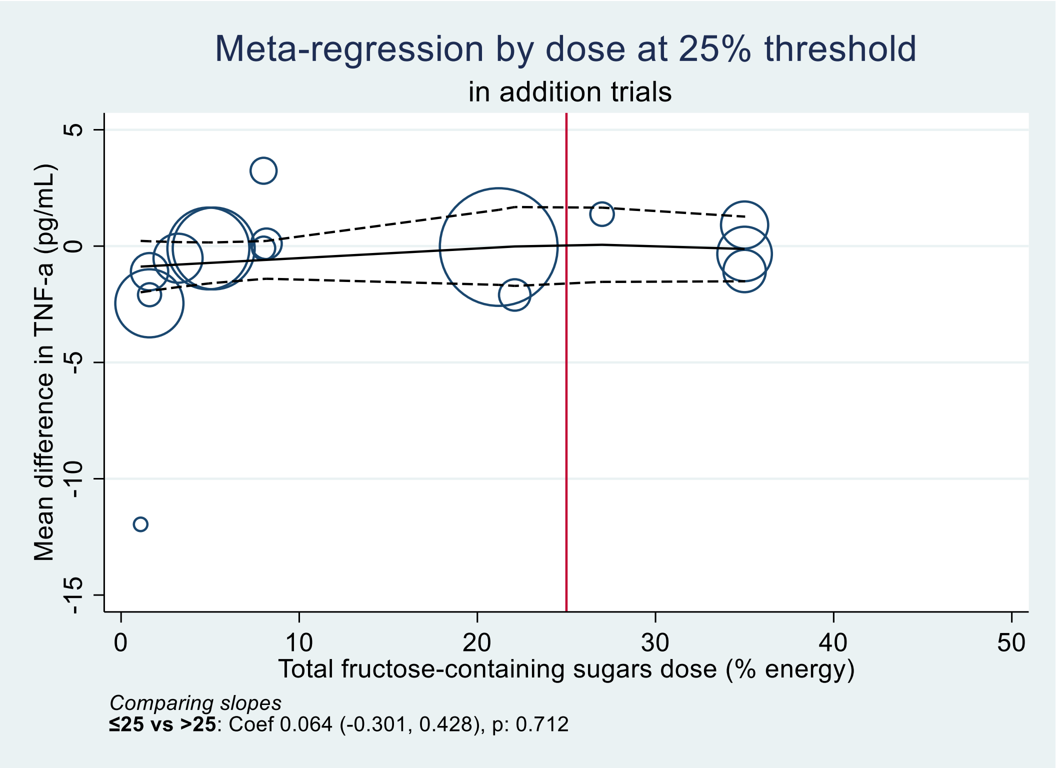

Coef=coefficient; %E=percentage of total energy intake; TNF-a= tumour necrosis factor-alpha; p-val= p-value
Individual trials are represented by the circles, with their weight in the overall analysis represented by the size of the circles. The straight line represents the estimate dose response for amount of fructose-containing sugars consumed (% of total energy intake) and the dashed lines represent the upper and lower 95% confidence intervals.
Panel A: 5% threshold; B: 10% threshold; C: 25% threshold.

Supplemental Figure S88: Non-linear dose-response analysis using public thresholds of 5% (panel A), 10% (panel B), and 25% (panel C) of energy for the effect of important food sources of fructose-containing sugars on IL-6 (pg/mL) in substitution trials

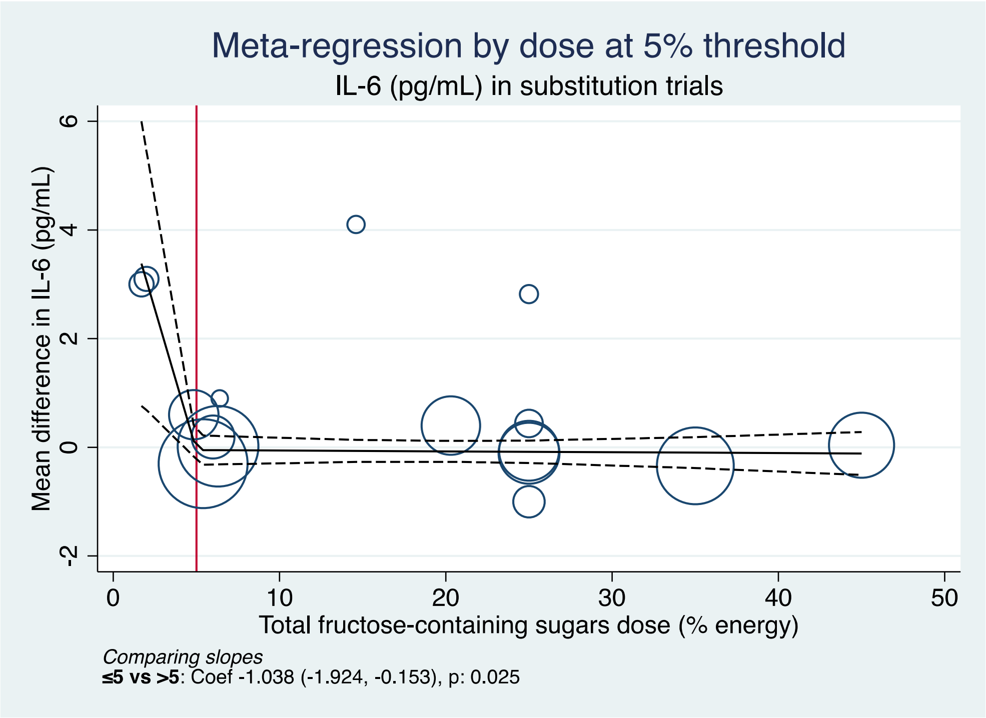

A:

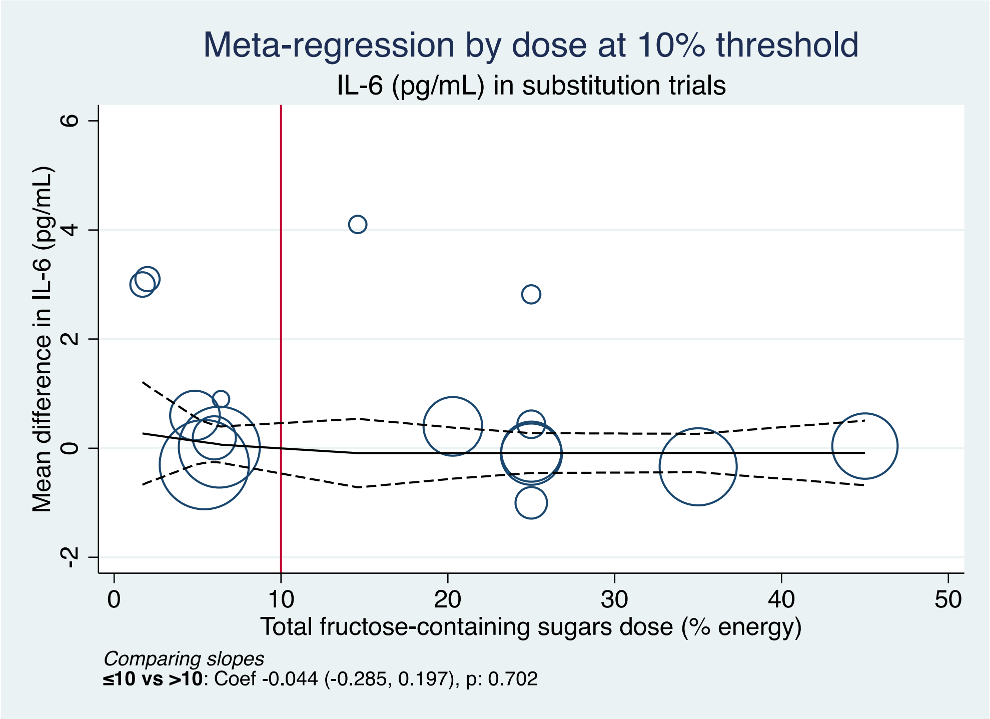

B:

C: 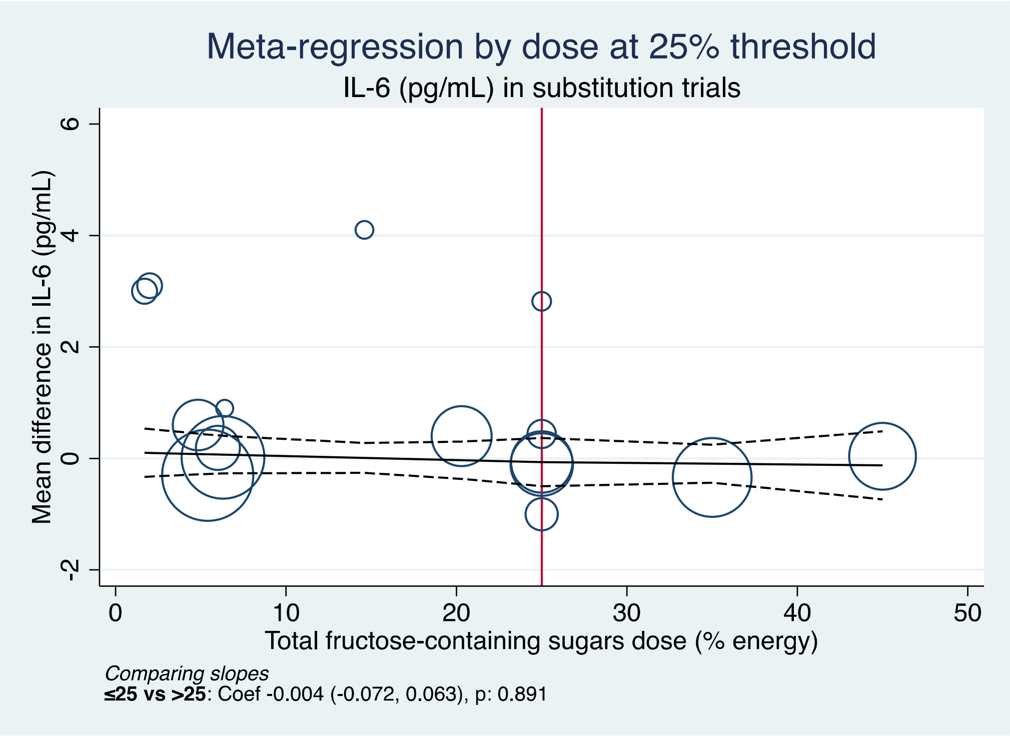

Coef=coefficient; %E=percentage of total energy intake; IL-6= interleukin-6; p-val= p-value
Individual trials are represented by the circles, with their weight in the overall analysis represented by the size of the circles. The straight line represents the estimate dose response for amount of fructose-containing sugars consumed (% of total energy intake) and the dashed lines represent the upper and lower 95% confidence intervals.
Panel A: 5% threshold; B: 10% threshold; C: 25% threshold.

Supplemental Figure S89: Non-linear dose-response analysis using public thresholds of 5% (panel A), 10% (panel B), and 25% (panel C) of energy for the effect of important food sources of fructose-containing sugars on IL-6 (pg/mL) addition trials

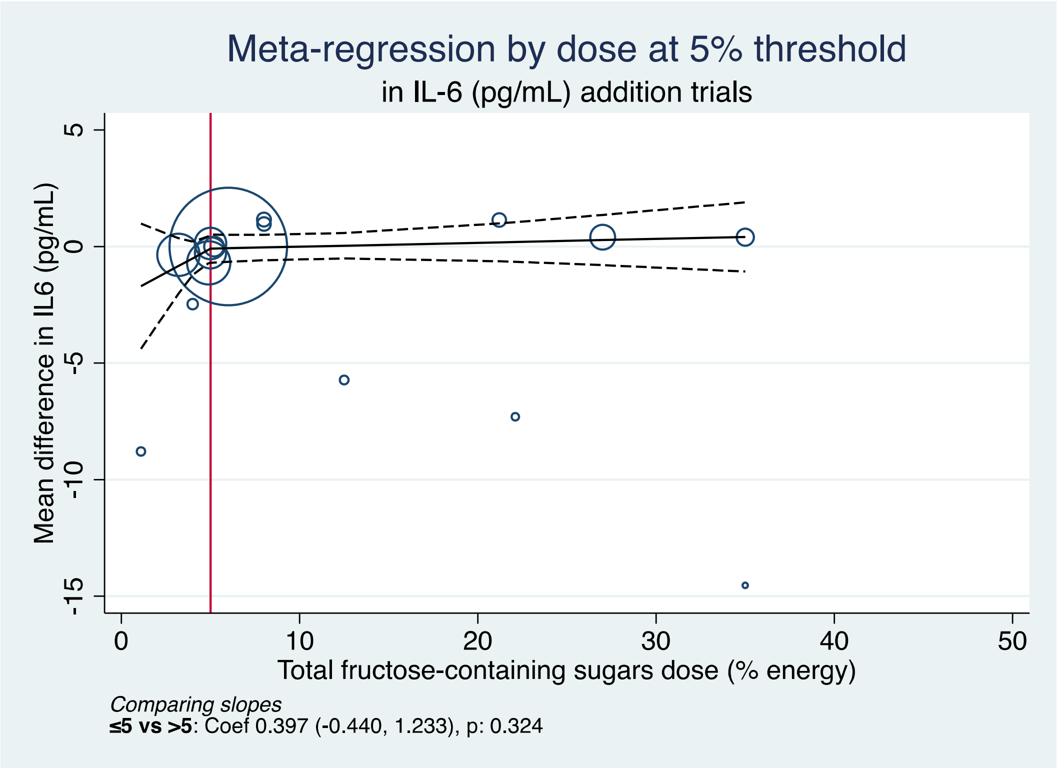

A:

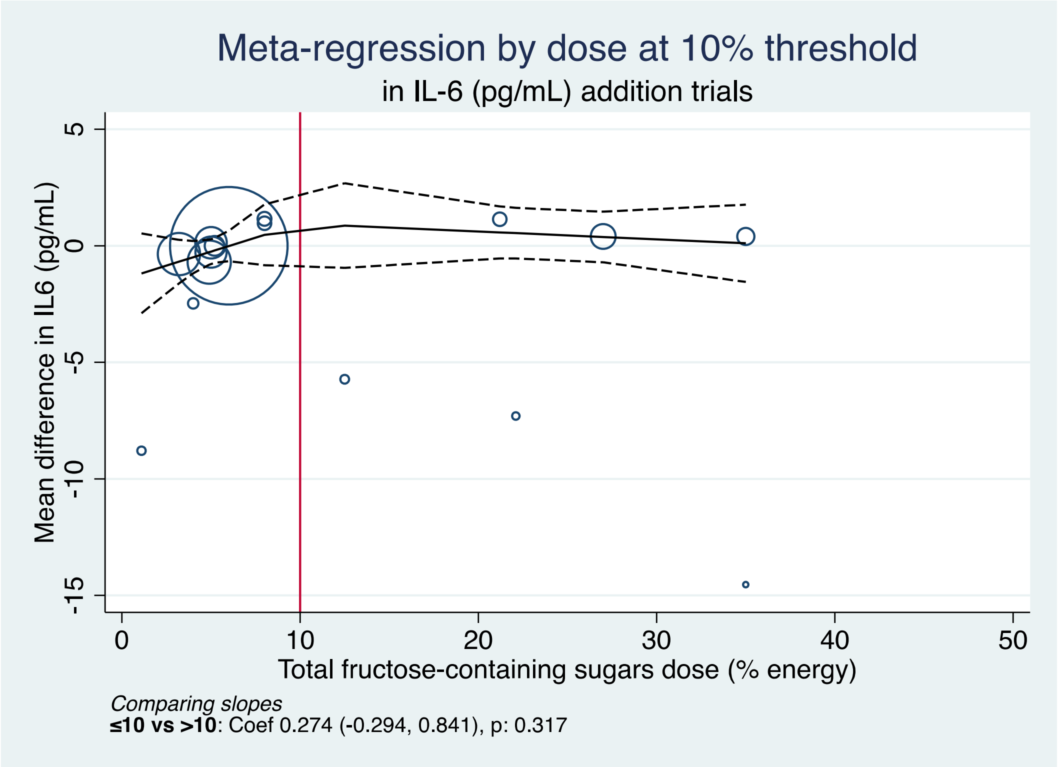

B:

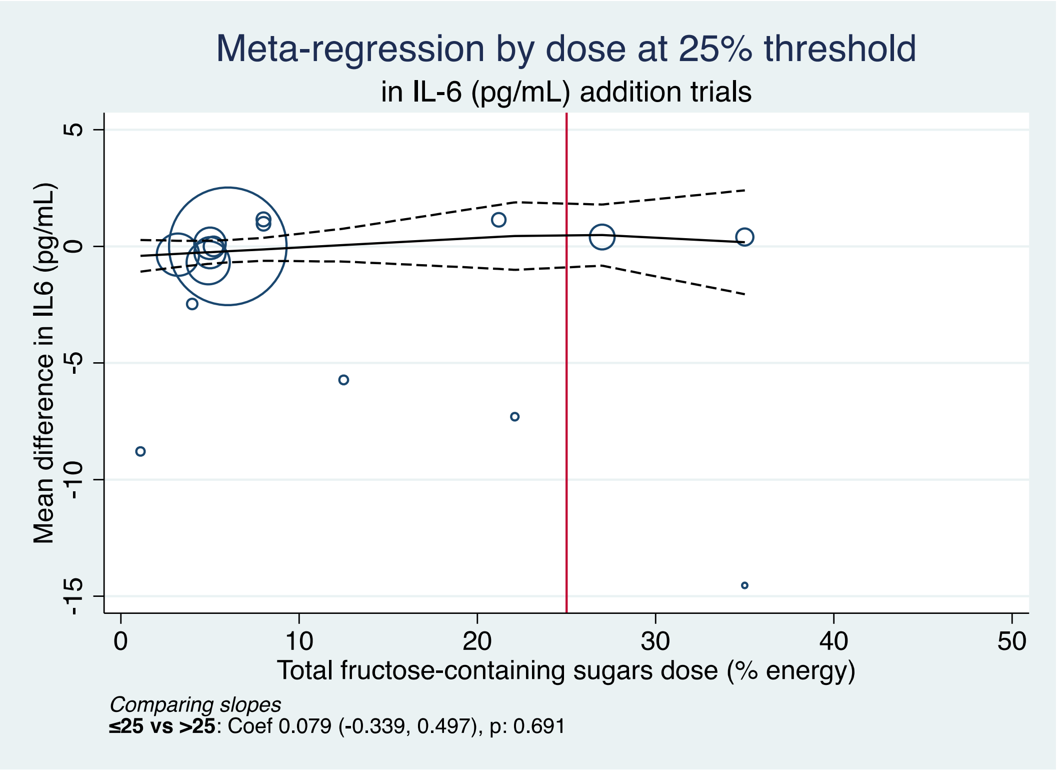

C:

Coef=coefficient; %E=percentage of total energy intake; IL-6= interleukin-6; p-val= p-value
Individual trials are represented by the circles, with their weight in the overall analysis represented by the size of the circles. The straight line represents the estimate dose response for amount of fructose-containing sugars consumed (% of total energy intake) and the dashed lines represent the upper and lower 95% confidence intervals.
Panel A: 5% threshold; B: 10% threshold; C: 25% threshold.

Supplemental Figure S90: Publication bias funnel plots for the effect of important food sources of fructose-containing sugars on CRP (mg/L) in substitution trials

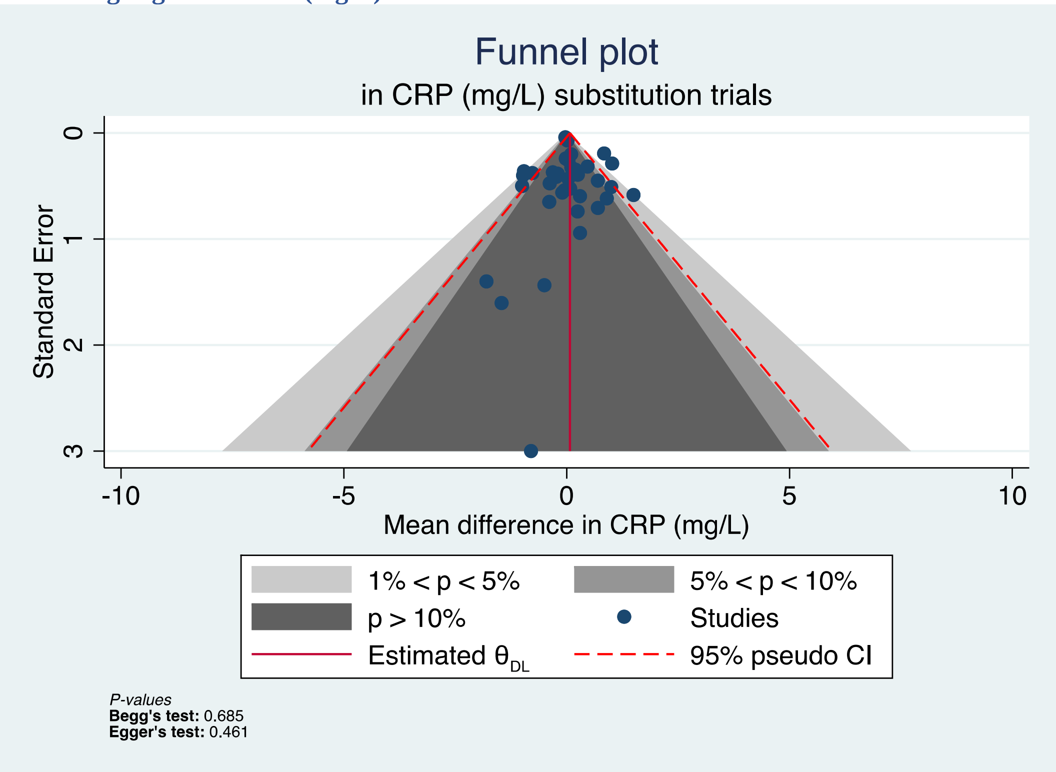

CI=confidence interval; CRP= C reactive protein

Contour-enhanced funnel plot is a scatterplot of each trial weighted mean difference on the x-axis with the standard error representing precision on the y-axis. The vertical solid red line represents the pooled effect estimate and the dashed red lines represent the pseudo-95% confidence limits. The blue dots represent individual trials. The contour regions define the regions for the test of significance of individual trial effect size for a given p-value range >0.100 (dark grey), 0.500 to <0.100 (medium grey), 0.010 to <0.500 (light grey), <0.0100 (white)]. The contour-enhanced funnel plots may suggest funnel-plot asymmetry is due to publication bias when less precise (smaller) trials are missing in the non-significant regions. Quantitative assessment of publication bias was also performed using Egger's and Begg's tests set at a significance level of P<0.100.

Supplemental Figure S91: Publication bias funnel plots for the effect of important food sources of fructose-containing sugars on CRP (mg/L) in addition trials

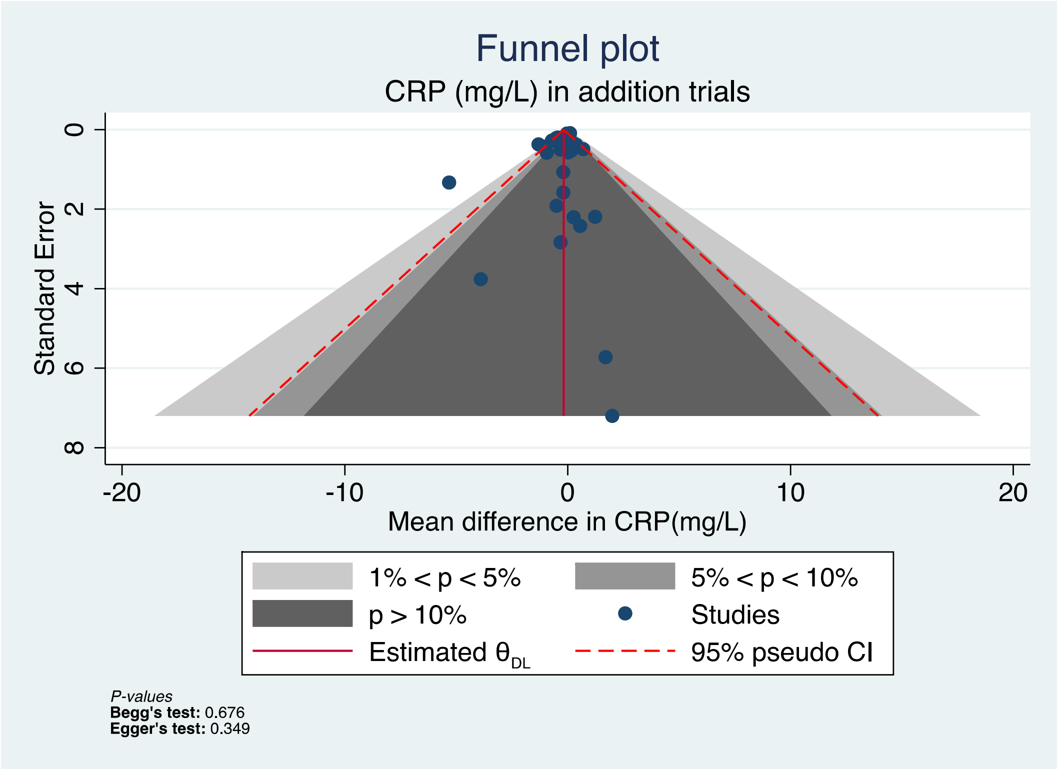

CI=confidence interval; CRP= C reactive protein

Contour-enhanced funnel plot is a scatterplot of each trial weighted mean difference on the x-axis with the standard error representing precision on the y-axis. The vertical solid red line represents the pooled effect estimate and the dashed red lines represent the pseudo-95% confidence limits. The blue dots represent individual trials. The contour regions define the regions for the test of significance of individual trial effect size for a given p-value range >0.100 (dark grey), 0.500 to <0.100 (medium grey), 0.010 to <0.500 (light grey), <0.0100 (white)]. The contour-enhanced funnel plots may suggest funnel-plot asymmetry is due to publication bias when less precise (smaller) trials are missing in the non-significant regions. Quantitative assessment of publication bias was also performed using Egger's and Begg's tests set at a significance level of P<0.100.

Supplemental Figure S92: Publication bias funnel plots for the effect of SSBs on CRP (mg/L) in substitution trials

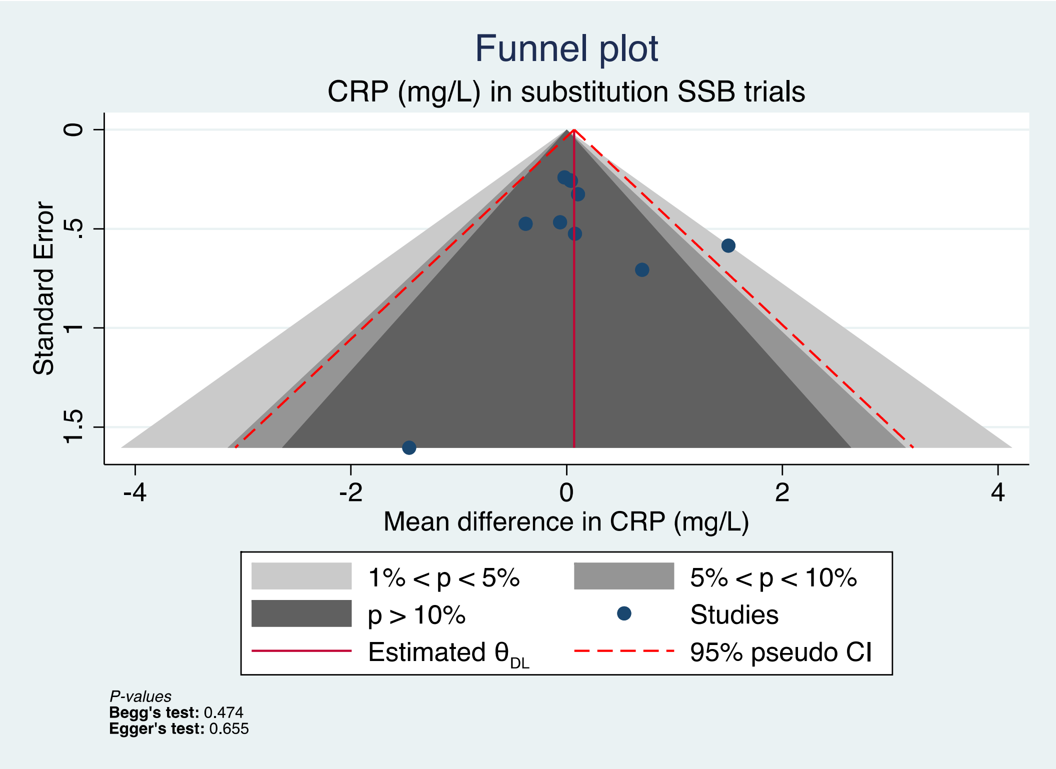

CI=confidence interval; CRP= C reactive protein; SSB= sugar sweetened beverages

Contour-enhanced funnel plot is a scatterplot of each trial weighted mean difference on the x-axis with the standard error representing precision on the y-axis. The vertical solid red line represents the pooled effect estimate and the dashed red lines represent the pseudo-95% confidence limits. The blue dots represent individual trials. The contour regions define the regions for the test of significance of individual trial effect size for a given p-value range >0.100 (dark grey), 0.500 to <0.100 (medium grey), 0.010 to <0.500 (light grey), <0.0100 (white)]. The contour-enhanced funnel plots may suggest funnel-plot asymmetry is due to publication bias when less precise (smaller) trials are missing in the non-significant regions. Quantitative assessment of publication bias was also performed using Egger's and Begg's tests set at a significance level of P<0.100.

Supplemental Figure S93: Publication bias funnel plots for the effect of 100% fruit juice on CRP (mg/L) in addition trials

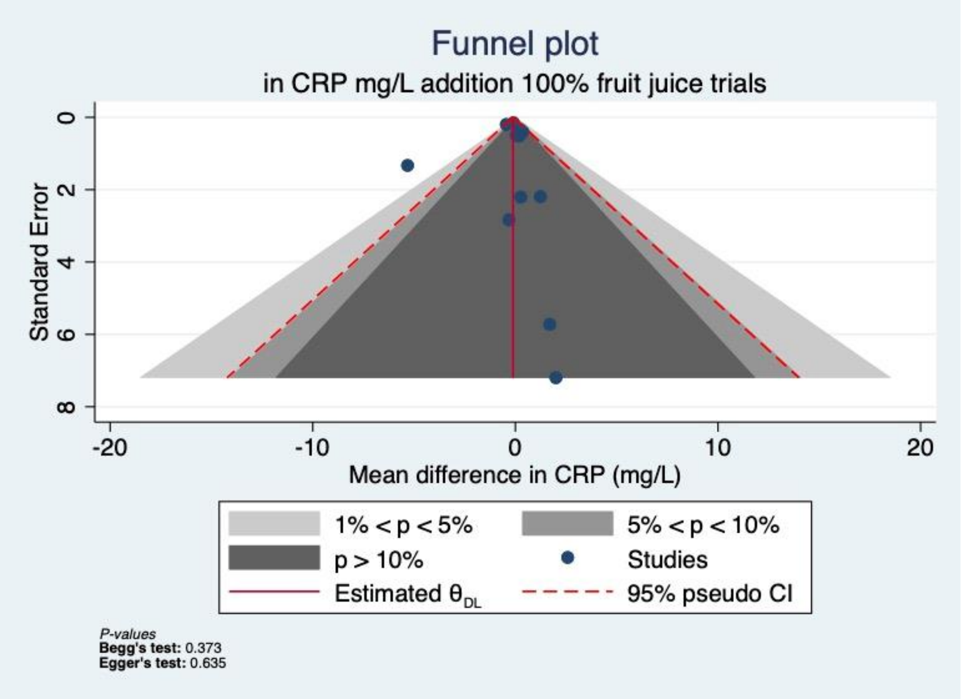

CI=confidence interval; CRP= C reactive protein

Contour-enhanced funnel plot is a scatterplot of each trial weighted mean difference on the x-axis with the standard error representing precision on the y-axis. The vertical solid red line represents the pooled effect estimate and the dashed red lines represent the pseudo-95% confidence limits. The blue dots represent individual trials. The contour regions define the regions for the test of significance of individual trial effect size for a given p-value range >0.100 (dark grey), 0.500 to <0.100 (medium grey), 0.010 to <0.500 (light grey), <0.0100 (white)]. The contour-enhanced funnel plots may suggest funnel-plot asymmetry is due to publication bias when less precise (smaller) trials are missing in the non-significant regions. Quantitative assessment of publication bias was also performed using Egger's and Begg's tests set at a significance level of P<0.100.

Supplemental Figure S94: Publication bias funnel plots for the effect of important food sources of fructose-containing sugars on TNF-α (pg/mL) in substitution trials

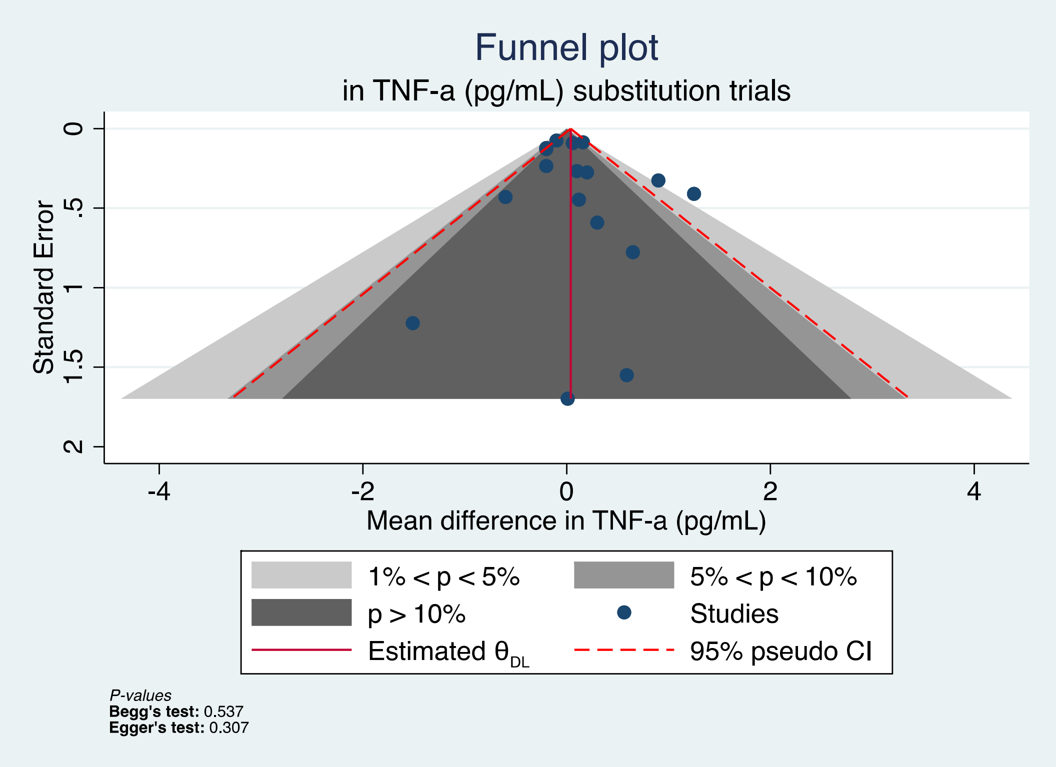

CI=confidence interval; TNF-a= tumour necrosis factor-alpha

Contour-enhanced funnel plot is a scatterplot of each trial weighted mean difference on the x-axis with the standard error representing precision on the y-axis. The vertical solid red line represents the pooled effect estimate and the dashed red lines represent the pseudo-95% confidence limits. The blue dots represent individual trials. The contour regions define the regions for the test of significance of individual trial effect size for a given p-value range >0.100 (dark grey), 0.500 to <0.100 (medium grey), 0.010 to <0.500 (light grey), <0.0100 (white)]. The contour-enhanced funnel plots may suggest funnel-plot asymmetry is due to publication bias when less precise (smaller) trials are missing in the non-significant regions. Quantitative assessment of publication bias was also performed using Egger's and Begg's tests set at a significance level of P<0.100.

Supplemental Figure S95: Publication bias funnel plots for the effect of important food sources of fructose-containing sugars on TNF-α (pg/mL) in addition trials

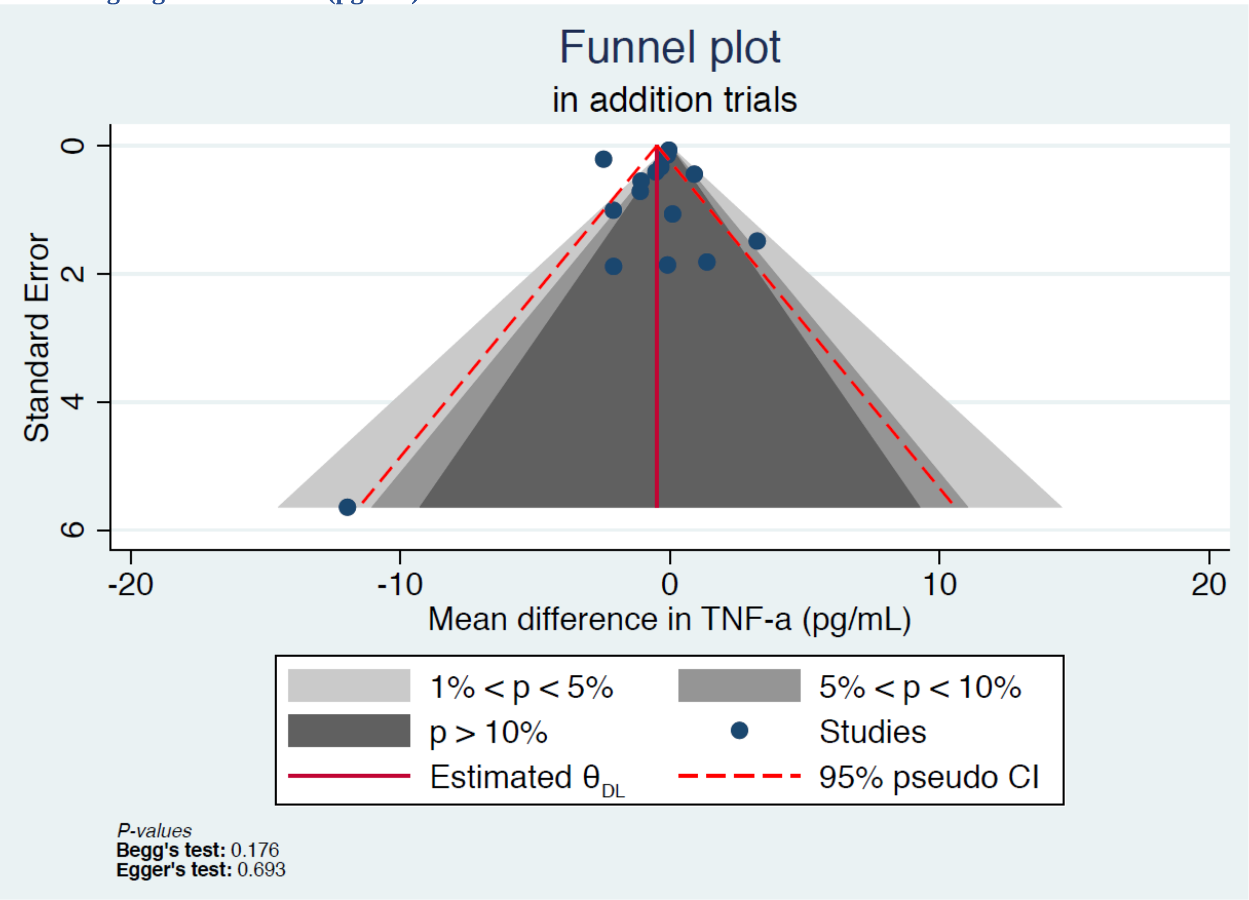

CI=confidence interval; TNF-a= tumour necrosis factor-alpha

Contour-enhanced funnel plot is a scatterplot of each trial weighted mean difference on the x-axis with the standard error representing precision on the y-axis. The vertical solid red line represents the pooled effect estimate and the dashed red lines represent the pseudo-95% confidence limits. The blue dots represent individual trials. The contour regions define the regions for the test of significance of individual trial effect size for a given p-value range >0.100 (dark grey), 0.500 to <0.100 (medium grey), 0.010 to <0.500 (light grey), <0.0100 (white)]. The contour-enhanced funnel plots may suggest funnel-plot asymmetry is due to publication bias when less precise (smaller) trials are missing in the non-significant regions. Quantitative assessment of publication bias was also performed using Egger's and Begg's tests set at a significance level of P<0.100.

Supplemental Figure S96: Publication bias funnel plots for the effect of important food sources of fructose-containing sugars on IL-6 (pg/mL) in substitution trials

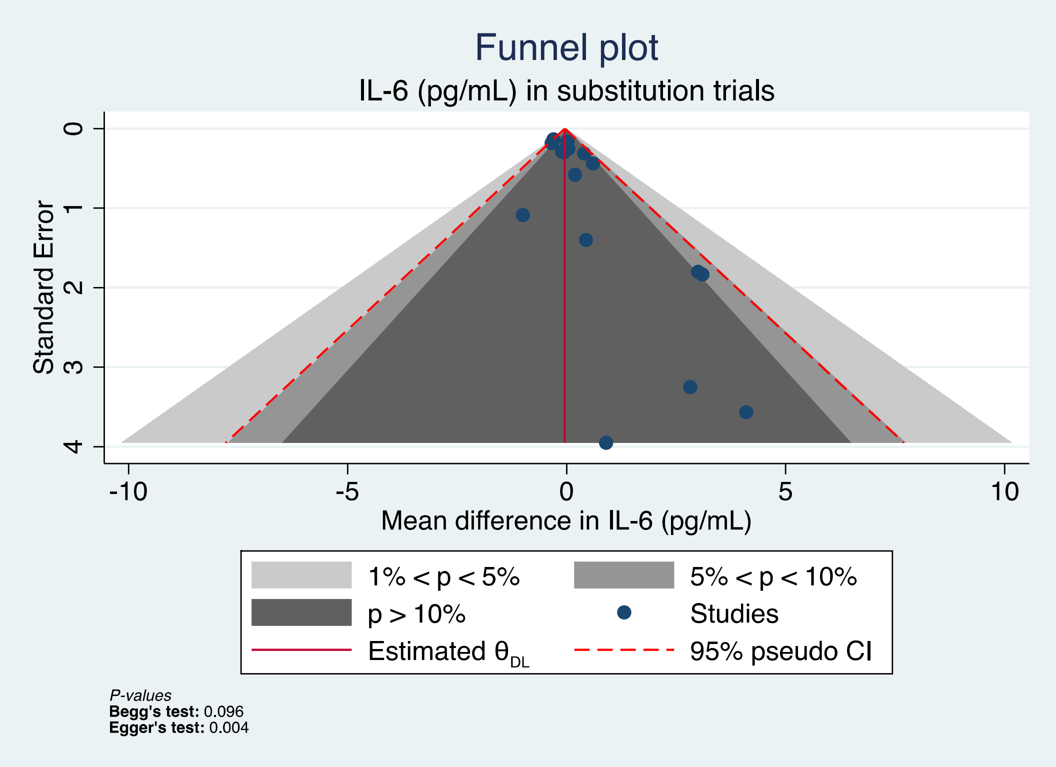

CI=confidence interval; IL-6 = interleukin-6

Contour-enhanced funnel plot is a scatterplot of each trial weighted mean difference on the x-axis with the standard error representing precision on the y-axis. The vertical solid red line represents the pooled effect estimate and the dashed red lines represent the pseudo-95% confidence limits. The blue dots represent individual trials. The contour regions define the regions for the test of significance of individual trial effect size for a given p-value range >0.100 (dark grey), 0.500 to <0.100 (medium grey), 0.010 to <0.500 (light grey), <0.0100 (white)]. The contour-enhanced funnel plots may suggest funnel-plot asymmetry is due to publication bias when less precise (smaller) trials are missing in the non-significant regions. Quantitative assessment of publication bias was also performed using Egger's and Begg's tests set at a significance level of P<0.100.

Supplemental Figure S97: Publication bias funnel plots for the effect of important food sources of fructose-containing sugars on IL-6 (pg/mL) in addition trials

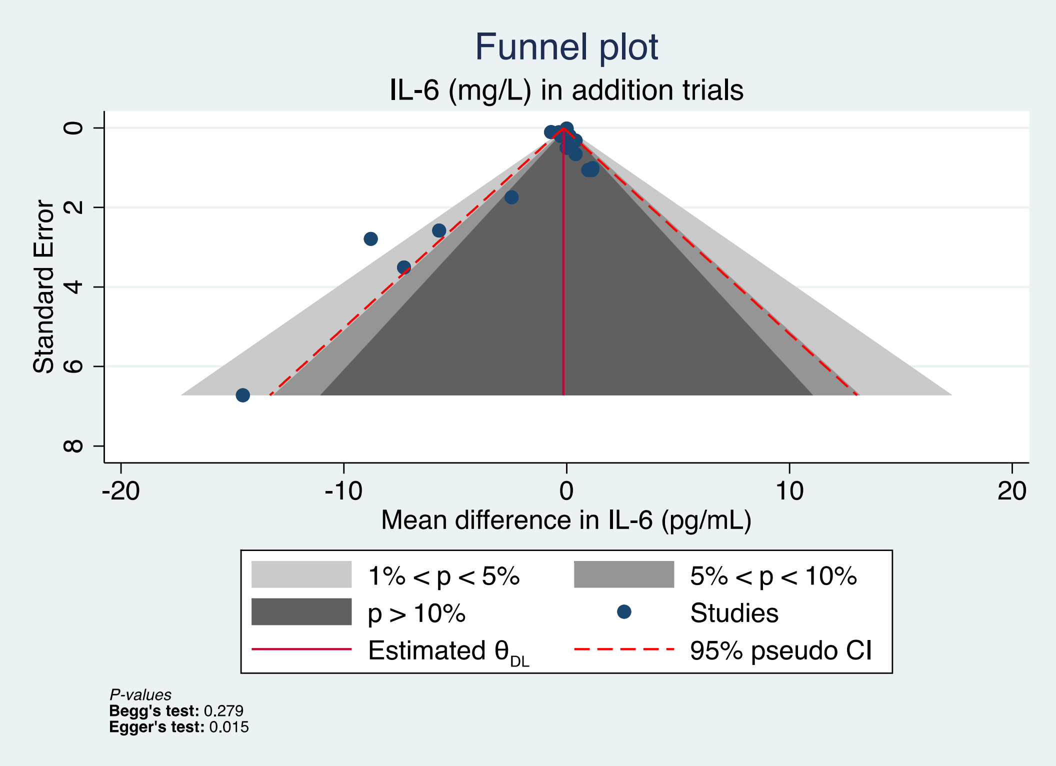

CI=confidence interval; IL-6= interleukin-6

Contour-enhanced funnel plot is a scatterplot of each trial weighted mean difference on the x-axis with the standard error representing precision on the y-axis. The vertical solid red line represents the pooled effect estimate and the dashed red lines represent the pseudo-95% confidence limits. The blue dots represent individual trials. The contour regions define the regions for the test of significance of individual trial effect size for a given p-value range >0.100 (dark grey), 0.500 to <0.100 (medium grey), 0.010 to <0.500 (light grey), <0.0100 (white)]. The contour-enhanced funnel plots may suggest funnel-plot asymmetry is due to publication bias when less precise (smaller) trials are missing in the non-significant regions. Quantitative assessment of publication bias was also performed using Egger's and Begg's tests set at a significance level of P<0.100.

Supplemental Figure S98: Trim and Fill funnel plot for the effect of important food sources of fructose-containing sugars IL-6 (pg/mL) in substitution trials

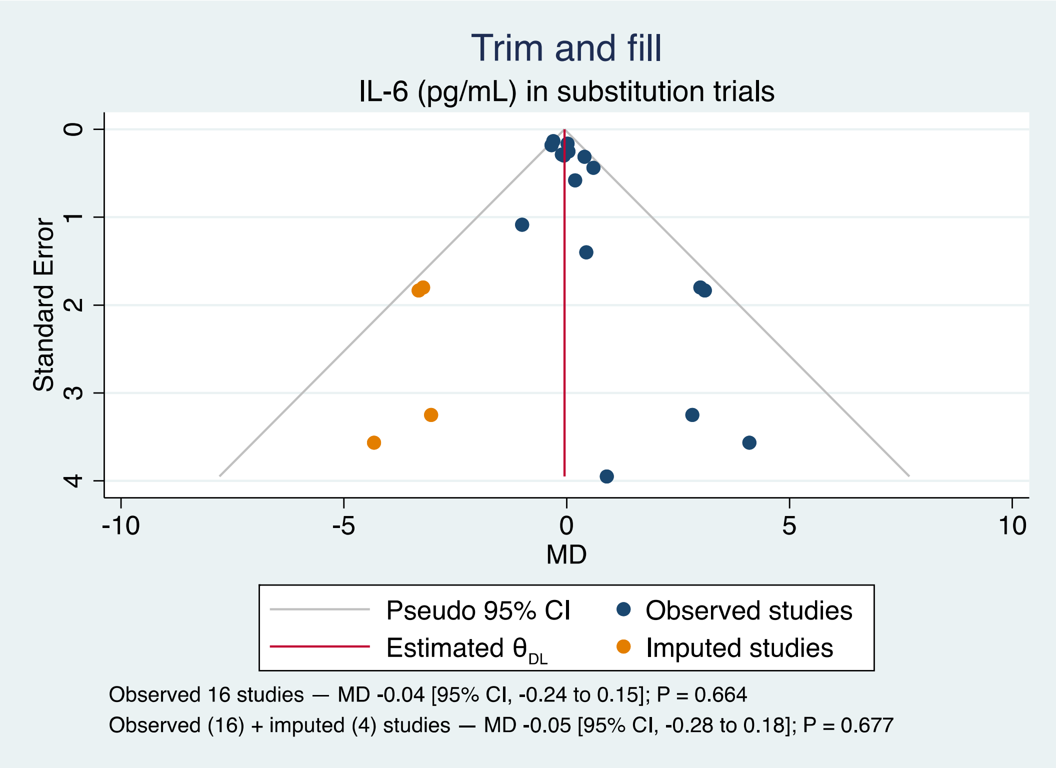

CI=confidence interval; IL-6= interleukin-6

The vertical line represents the pooled effect estimate expressed as standardized mean difference. The diagonal lines represent the pseudo-95% confidence limits, the blue circles represent the effect estimate for each included study, and orange circles represent the effect estimate for each imputed "missed" study. Imputed random standardized mean difference is provided; when the imputed result differs from the primary result in either significance or magnitude (>1 MID =0.18 pg/mL for IL-6), this is considered evidence of small-study effects.

Supplemental Figure S99: Trim and Fill funnel plot for the effect of important food sources of fructose-containing sugars IL-6 (pg/mL) in addition trials

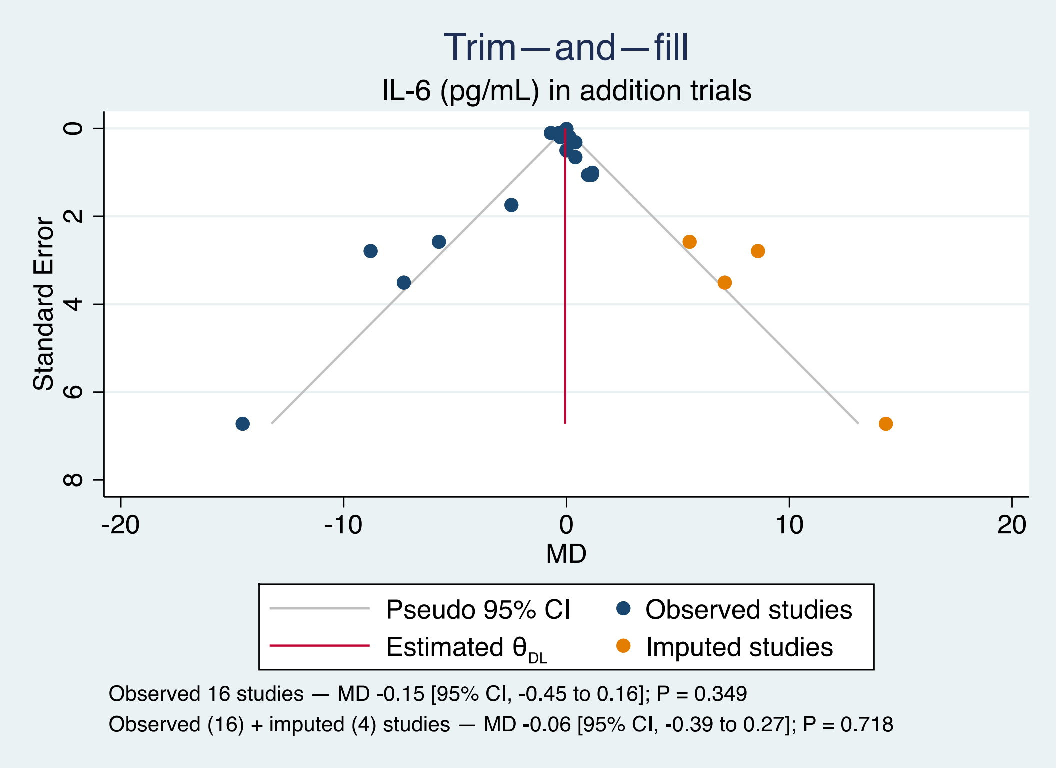

CI=confidence interval; IL-6= interleukin-6

The vertical line represents the pooled effect estimate expressed as standardized mean difference. The diagonal lines represent the pseudo-95% confidence limits, the blue circles represent the effect estimate for each included study, and orange circles represent the effect estimate for each imputed "missed" study. Imputed random standardized mean difference is provided; when the imputed result differs from the primary result in either significance or magnitude (>1 MID =0.18 pg/mL for IL-6), this is considered evidence of small-study effects.
